# Supplementary material for: NRF3 suppresses squamous carcinogenesis, involving the unfolded protein response regulator HSPA5
Source: EMBO Mol Med. 2023 Oct 9;15(11):e17761. doi: 10.15252/emmm.202317761 (PMC10630885; doi:10.15252/emmm.202317761)
Supplement: Supplementary file 1 — Appendix S1 [file EMMM-15-e17761-s011.pdf]

Table of Content

Appendix Figure S1	Page 2
Appendix Figure S2	Page 3
Appendix Figure S3	Page 4
Appendix Figure S4	Page 5
Appendix Figure S5	Page 6
Appendix Figure S6	Page 7

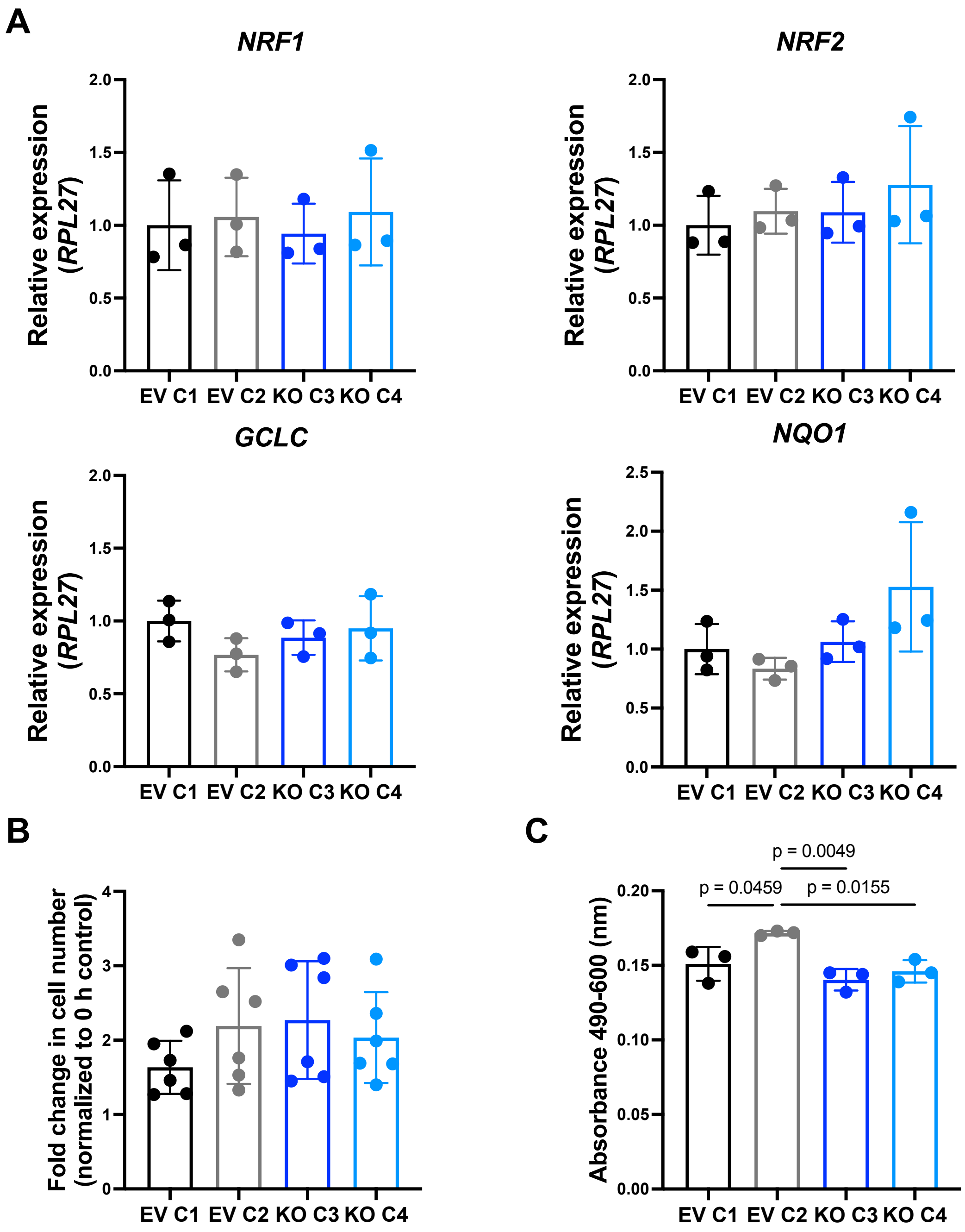

Appendix Figure S1 - Loss of NRF3 neither affects the viability of SCC13 cells nor the expression of *NRF1* and *NRF2* and of major NRF2 target genes under normal culture conditions

SCC13 EV and *NRF3*-KO cells were cultivated under normal culture conditions for 24 h.

A qRT-PCR using RNA from SCC13 EV and *NRF3*-KO cells for *NRF1, NRF2, GCLC* and *NQO1* relative to *RPL27*. N=3. Mean expression in EV C1 cells was set to 1.

B Fold change in cell number based on MTT assay (N = 6)

C Cytotoxicity based on LDH Assay (N = 3).

Data information: Bar graphs show mean +/- S.D. P-values were determined using Kruskal-Wallis test.

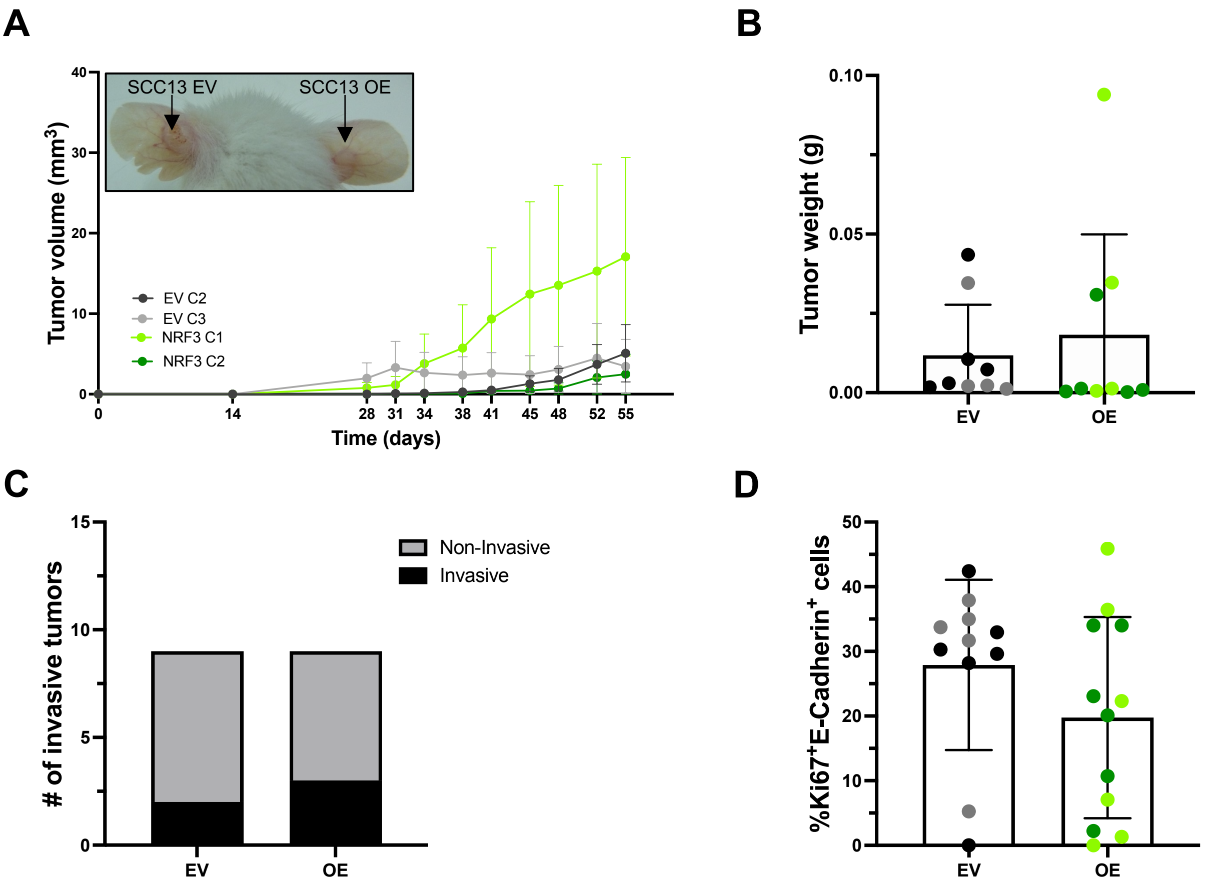

Appendix Figure S2 - Overexpression of NRF3 does not affect tumorigenesis and invasiveness of SCC13 cells in a xenograft mouse model

SCC13 cells transduced with EV or an NRF3 expression vector were intradermally injected into the ear of NOD/SCID mice to induce tumor formation.

A Representative pictures of ~8-week-old tumors (indicated by arrows) and tumor volume at different time points of tumor development. N = 4-5 tumors per cell line. Graph shows mean +/- S.E.M.

B Tumor weight at endpoint. N = 9 tumors.

C Percentage of tumors that had invaded through the basement membrane based on H&E-stained tumor sections. N = 9.

D Percentage of Ki67/E-cadherin-positive cells among all E-cadherin positive cells in the tumors. N = 4 tumors, n = 9-15 sections.

Data information: Bar graphs show absolute values (C), mean +/- S.E.M. (A) or mean +/- S.D. (B,D). P-values were determined using Mann-Whitney U test (B, D) or Fisher's exact test (C). Black or grey dots indicate data points from different EV cell lines; dark or light green dots indicate data points from different OE cell lines.

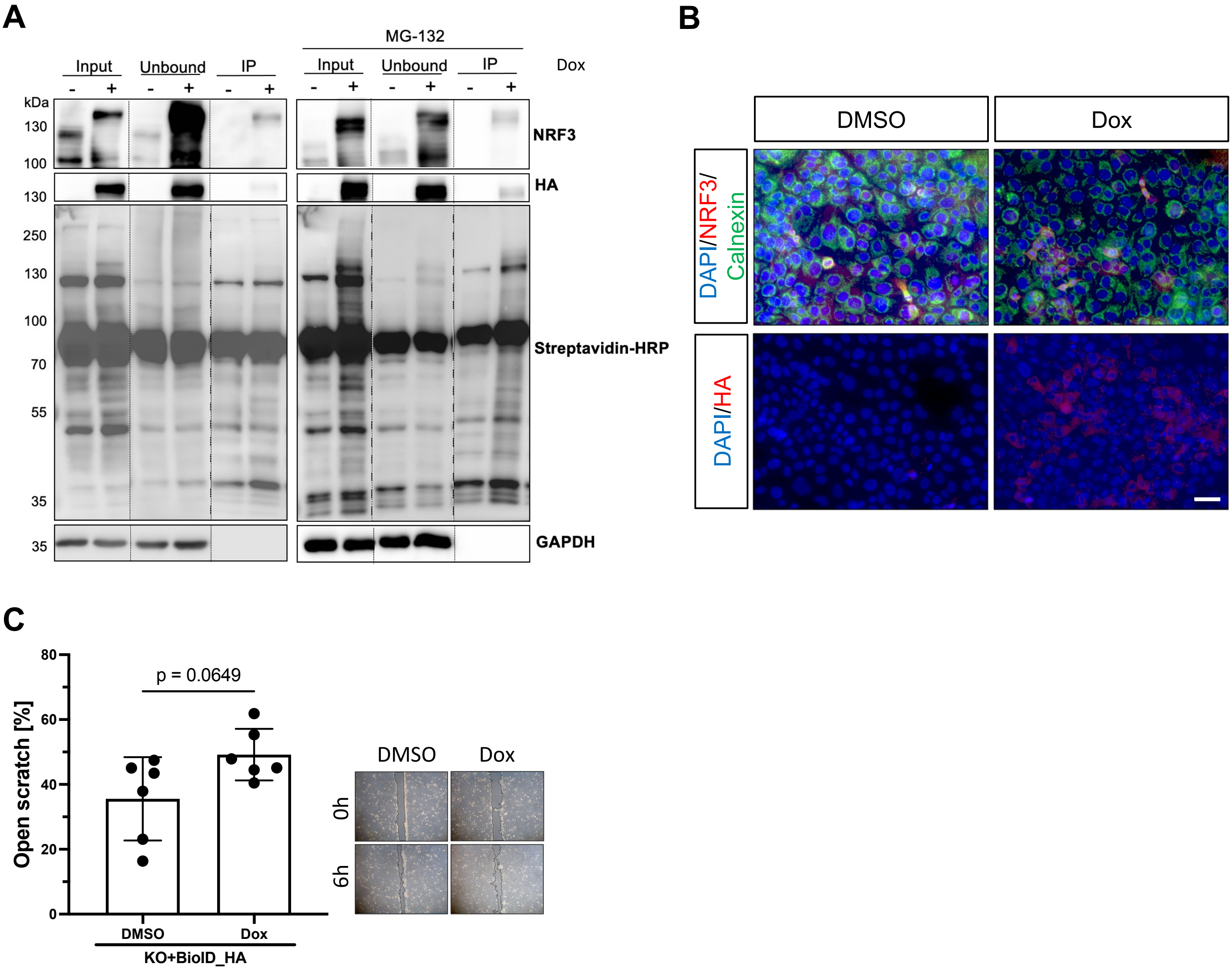

Appendix Figure S3 - Validation of NRF3-BioID fusions proteins in SCC13 cells

A Western blot for NRF3, HA fusion proteins and GAPDH and detection of biotinylated proteins using horseradish peroxidase (HRP)-coupled streptavidin in lysates from SCC13 cells with inducible expression of NRF3-BioID fusion proteins in the presence of Dox and the proteasome inhibitor MG-132 or vehicle. The dashed lines indicate where the membrane was cropped.

B Representative immunofluorescence stainings of SCC13 cells with Dox-inducible expression of HA-tagged NRF3-BioID fusion proteins cultured in the presence or absence of Dox. Cells were stained with antibodies against the ER marker calnexin (green), NRF3 (red) or the HA epitope (red) and counterstained with Hoechst (blue). Scale bar: 50 µm.

C Confluent SCC13 cells with Dox-inducible expression of HA-tagged NRF3-BioID fusion proteins were subjected to scratch wounding in the presence or absence of Dox (1 µg/ml). N = 6. The area of open scratch at 6 h is shown as percentage of the initial open scratch.

Data information: Bar graphs show mean +/- S.D.. P-values were determined using Mann-Whitney U test.

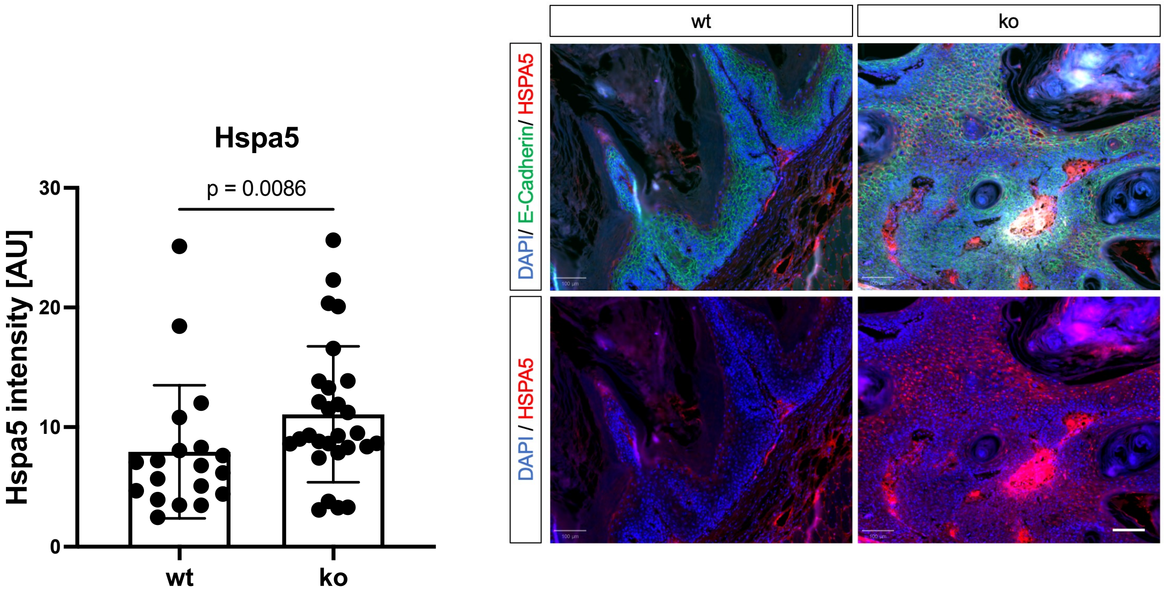

Appendix Figure S4 - HSPA5 levels are enhanced in the DMBA/TPA-induced tumors in *Nrf3*-ko mice

A Quantification of the HSPA5 staining intensity and representative images of sections from DMBA/TPA-induced tumors in wt and *Nrf3*-ko mice stained for HSPA5 (red), E-Cadherin (green) combined with Hoechst staining (blue). N = 8-9 tumors, n = 1-4 histological sections. Scale bar: 100 µm.

Data information: Graphs show mean +/- S.D.. P-value was determined using Mann-Whitney U test.

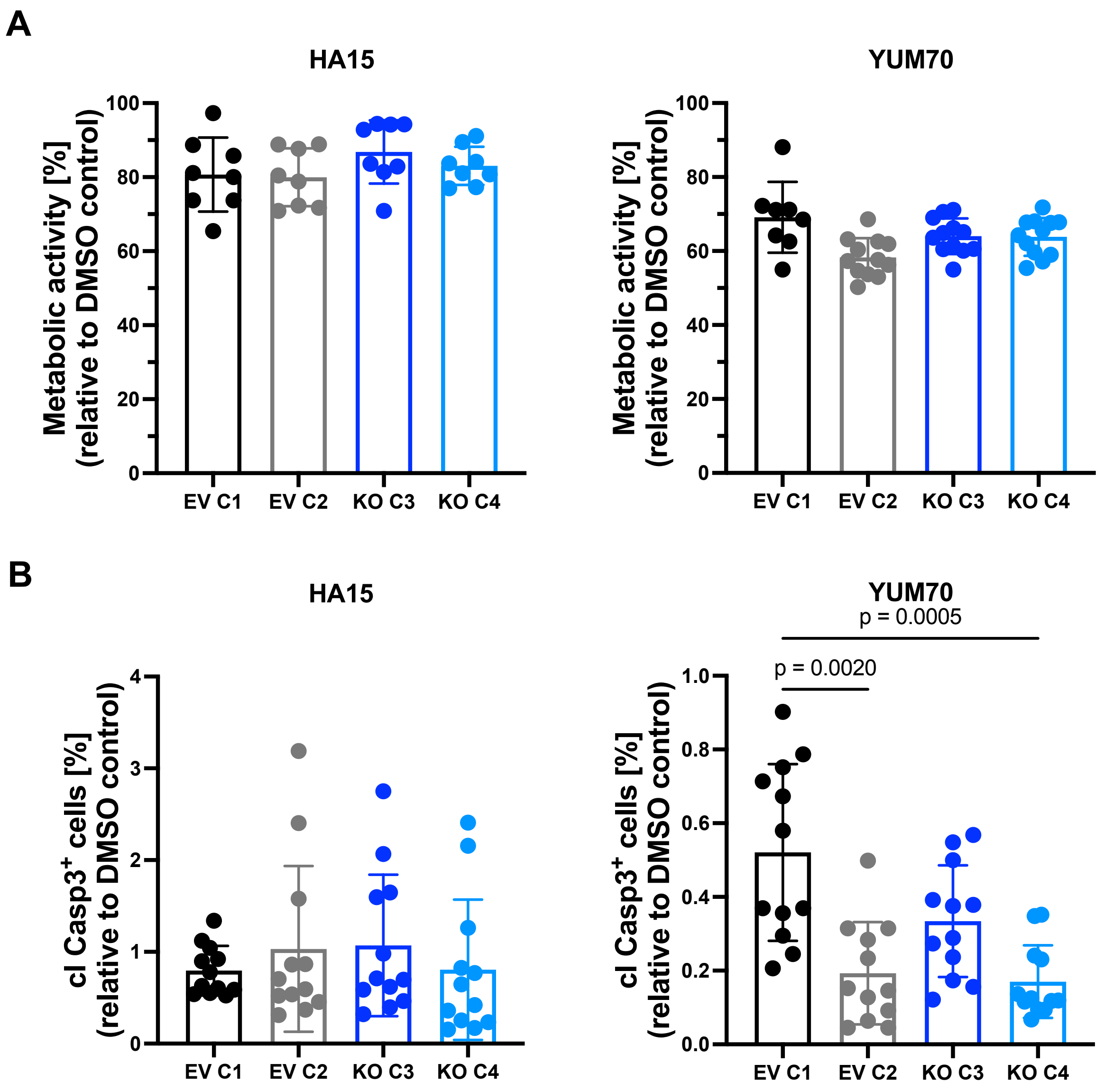

Appendix Figure S5 - HSPA5 inhibitors do not affect survival of SCC13 EV and *NRF3*-KO cells under non-challenged conditions

SCC13 EV and *NRF3*-KO cells were treated with DMSO, 25 μM HA15 (left) or 5 μM YUM70 (right) for 24 h.

A Metabolic activity of inhibitor-treated cells based on MTT assay relative to DMSO-treated control cells (N = 8).

B Percentage of cleaved caspase 3 positive inhibitor-treated cells relative to DMSO-treated control cells (N = 12).

Data information: Bar graphs show mean +/- S.D.. P-values were determined using Kruskal-Wallis test.

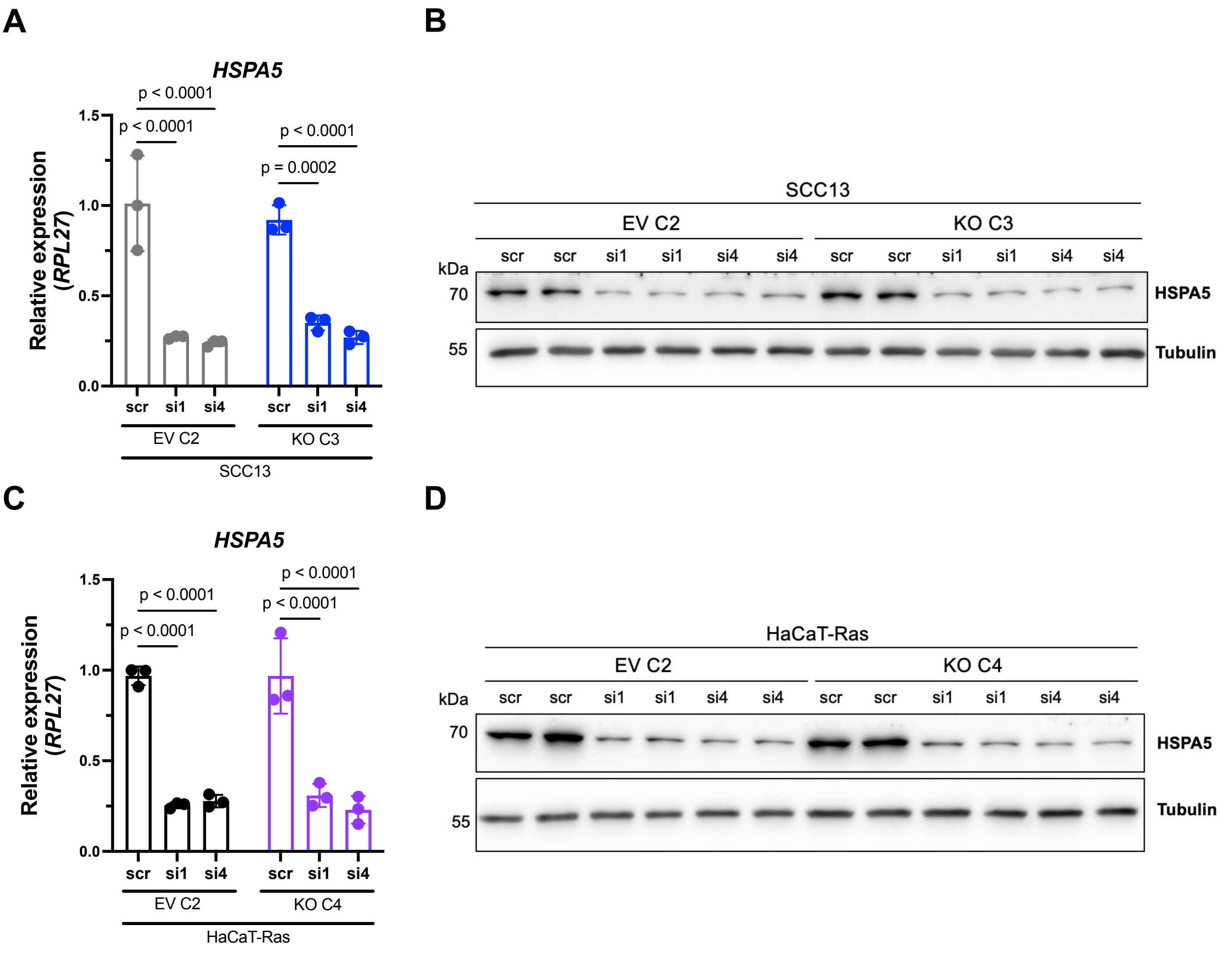

Appendix Figure S6 - Validation of siRNA-mediated HSPA5 knock-down

A-D SCC13 EV or *NRF*3-KO cells (A, B) or HaCaT-Ras EV or *NRF3*-KO cells (C, D) were transfected with scrambled (scr) or HSPA5 siRNAs and analyzed 24 h (RNA) or 48 h (protein) after transfection.

(A, C) qRT-PCR using RNA from the knock-down cells for *HSPA5* relative to *RPL27*.

(B, D) Western blot of total cell lysates of the knock-down cells using antibodies against HSPA5 or tubulin (loading control).

Data information: Bar graphs show mean +/- S.D.. P-values were determined using 2-way ANOVA.
