# Supplementary material for: NRF3 suppresses squamous carcinogenesis, involving the unfolded protein response regulator HSPA5
Source: EMBO Mol Med. 2023 Oct 9;15(11):e17761. doi: 10.15252/emmm.202317761 (PMC10630885; doi:10.15252/emmm.202317761)
Supplement: Supplementary file 2 — Expanded View Figures PDF [file EMMM-15-e17761-s010.pdf]

Expanded View Figures

Figure EV1. Loss of Nrf3 has no significant effect on the immune cell composition of chemically induced papillomas.

A Representative immunohistochemistry stainings of DMBA/TPA-induced acanthopapillomas (AP) and SCCs for $CD3^+$ T-cells (upper panel) or $Ly6G^+$ neutrophils (middle panel) and toluidine blue staining for mast cells (lower panel). Scale bar: 500 μm.

B–D Quantification of $CD3^+$ cells (B) $Ly6G^+$ cells (C) toluidine $blue^+$ (TB) (D) cells per mm^2 tumor tissue. $N_{AP\text{-}wt} = 22$, $N_{AP\text{-}ko} = 15$, $N_{SCC\text{-}wt} = 3$, $N_{SCC\text{-}ko} = 10$ tumors.

Data information: Graphs show mean ± SD.

© 2023 The Authors

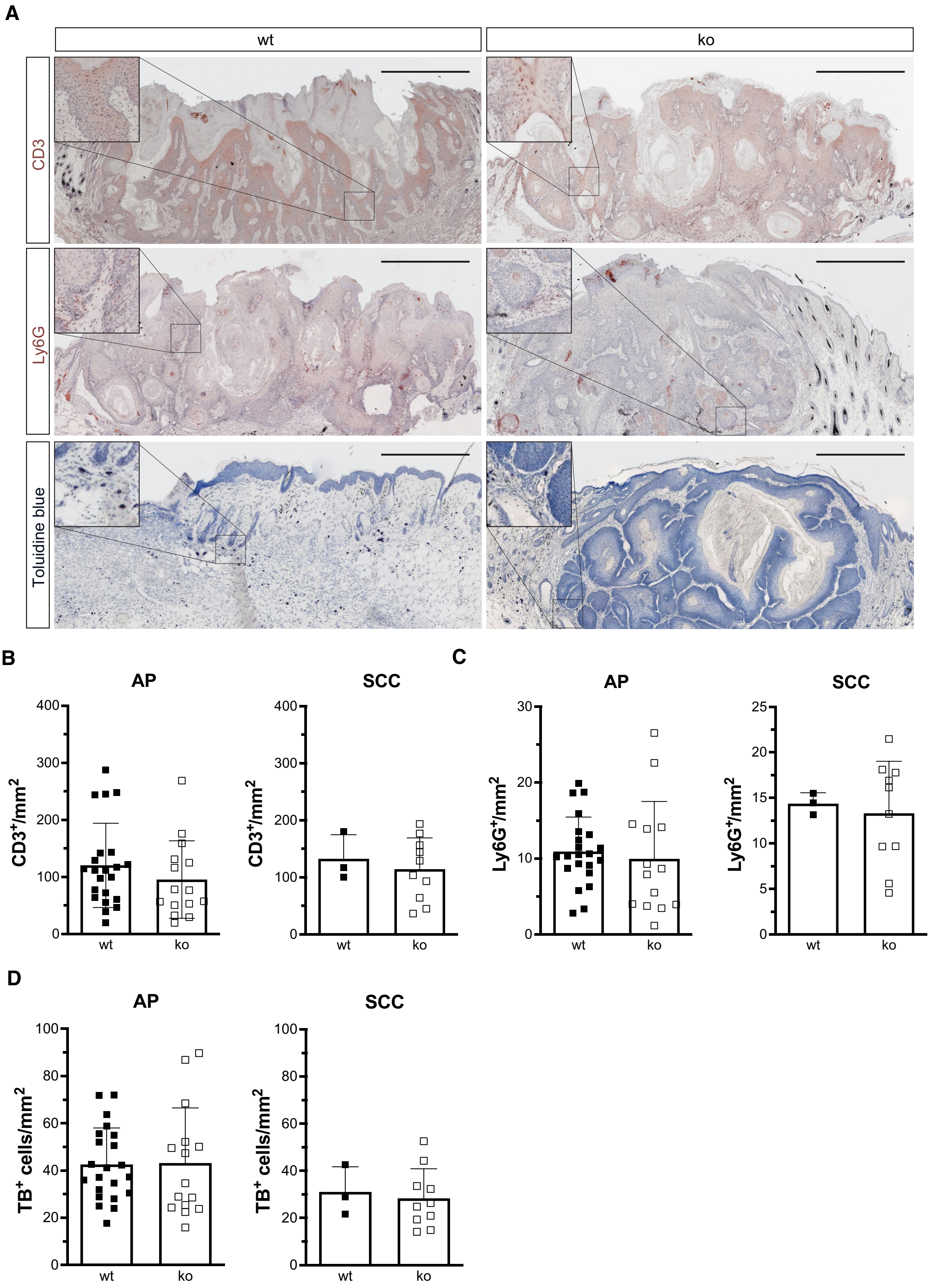

Figure EV1.

© 2023 The Authors

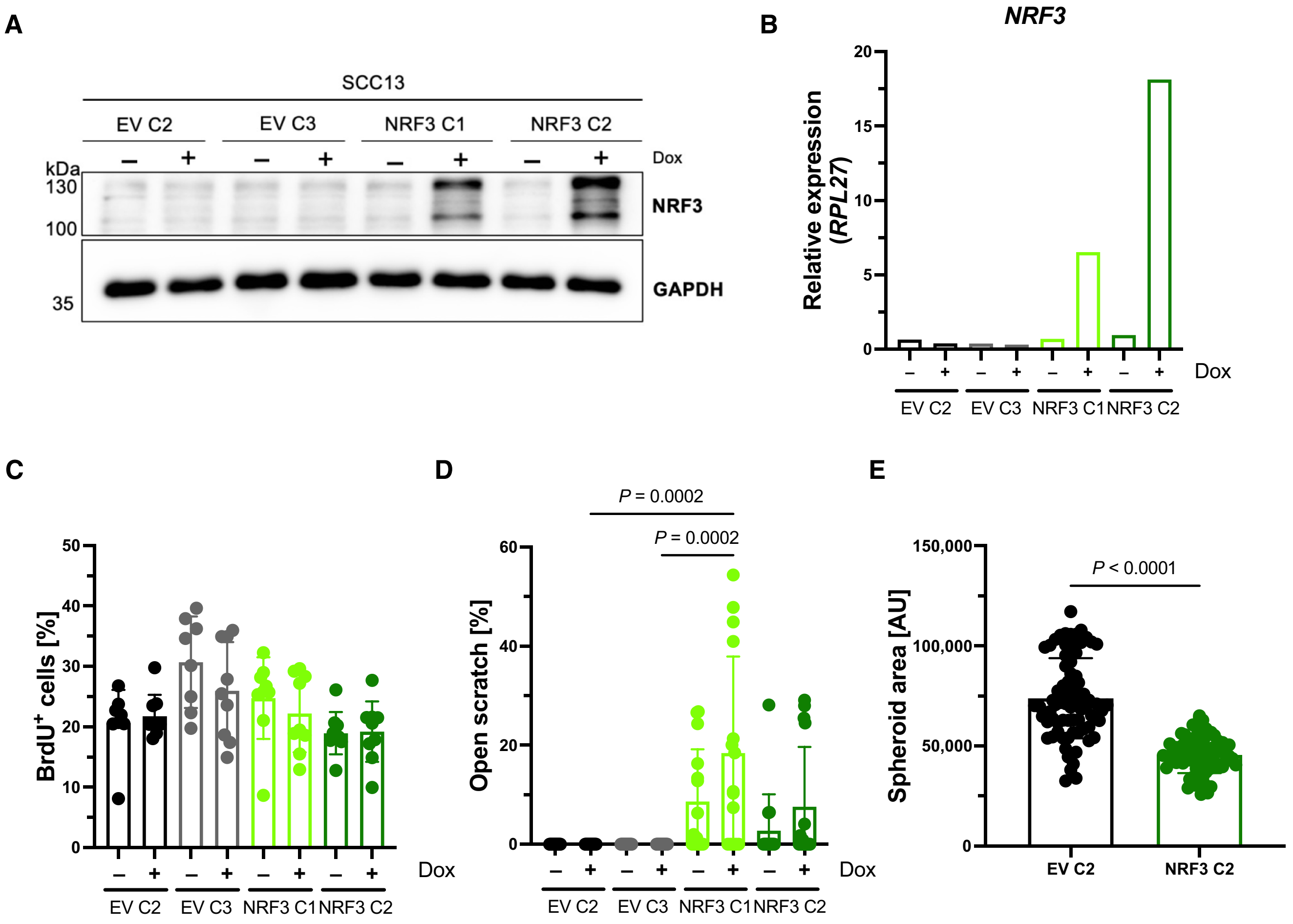

Figure EV2. Overexpression of NRF3 mildly reduces malignant features of SCC13 cells in 2D and 3D cell cultures.

A Western blot of total lysates of SCC13 cells with Dox-inducible overexpression of NRF3, treated with Dox (100 ng/ml) or vehicle, using antibodies against NRF3 and GAPDH.

B qRT-PCR for *NRF3* relative to *RPL27* using RNA from SCC13 cells with Dox-inducible overexpression of NRF3, treated with Dox (100 ng/ml) or vehicle. $N = 1$ per cell line, genotype and treatment group.

C Percentage of cells that had incorporated BrdU in cultures of SCC13 cells transduced with EV or NRF3 expression vectors and treated with Dox (100 ng/ml) or vehicle. $N = 8$–9 cultures.

D Quantification of the area of open scratch at 24 h post scratch wounding in percentage to the original scratch area in confluent SCC13 cells transduced with EV or NRF3 expression vectors and treated with Dox (100 ng/ml) or vehicle. $N = 12$.

E Area of spheroids formed by SCC13 cells transduced with EV or NRF3 expression vectors and treated with Dox (100 ng/ml) in single hanging drops. $N = 75$–80 hanging drops.

Data information: Graphs show mean ± SD. *P*-values were determined using Kruskal–Wallis test (C, D) or Mann–Whitney *U* test (E).

© 2023 The Authors

Figure EV3. Loss of NRF3 promotes malignancy of HaCaT-Ras cells in 2D and 3D cell cultures and enhances tumorigenesis and invasiveness in a xenograft mouse model.

A Western blot of cell lysates from transduced and clonally expanded HaCaT-Ras EV and *NRF3*-KO cells, treated with MG-132 or vehicle, using antibodies against NRF3, NRF1, NRF2, and tubulin.
B qRT-PCR using RNA from HaCaT-Ras EV and *NRF3*-KO cells for *NRF1*, *NRF2*, *GCLC*, and *NQO1* relative to *RPL27*. *N* = 3. Mean expression in EV C2 cells was set to 1.
C Area of colonies formed by HaCaT-Ras EV and *NRF3*-KO cells relative to the whole area of the well. *N* = 18–23.
D Percentage of HaCaT-Ras EV and *NRF*3-KO cells that had incorporated BrdU. *N* = 9.
E Quantification of the area of open scratch at 9 h post scratch wounding in percentage of the original scratch area in confluent HaCaT-Ras EV and *NRF*3-KO cells. *N* = 9.
F Area of spheroids formed by HaCaT-Ras EV and *NRF*3-KO cells in single hanging drops. *N* = 14 hanging drops.
G Representative pictures of ~ 6.5-week-old tumors (indicated by arrows) formed in the ear of NOD/SCID mice upon intradermal injection of HaCaT-Ras EV and *NRF3*-KO cells and tumor volume at different time points of tumor development. *N* = 6 tumors.
H Representative images of H&E-stained sections from tumors formed by HaCaT-Ras EV and *NRF3*-KO cells at day 45. Scale bar: 500 μm. Graph shows tumor weight at end point. *N* = 6 tumors.
I Percentage of tumors that had invaded through the basement membrane based on H&E-stained tumor sections. *N* = 6.
J Percentage of Ki67-positive cells among all E-cadherin-positive cells in tumors formed by HaCaT-Ras EV and *NRF3*-KO cells. *N* = 3 tumors, *n* = 15 sections.

Data information: Bar graph in (G) shows mean ± SEM, and the other graphs show mean ± SD. *P*-values were determined using Kruskal–Wallis (B–E), Mann–Whitney *U* (F, H, J), 2-way ANOVA (G), or Fisher's exact test (I).

© 2023 The Authors

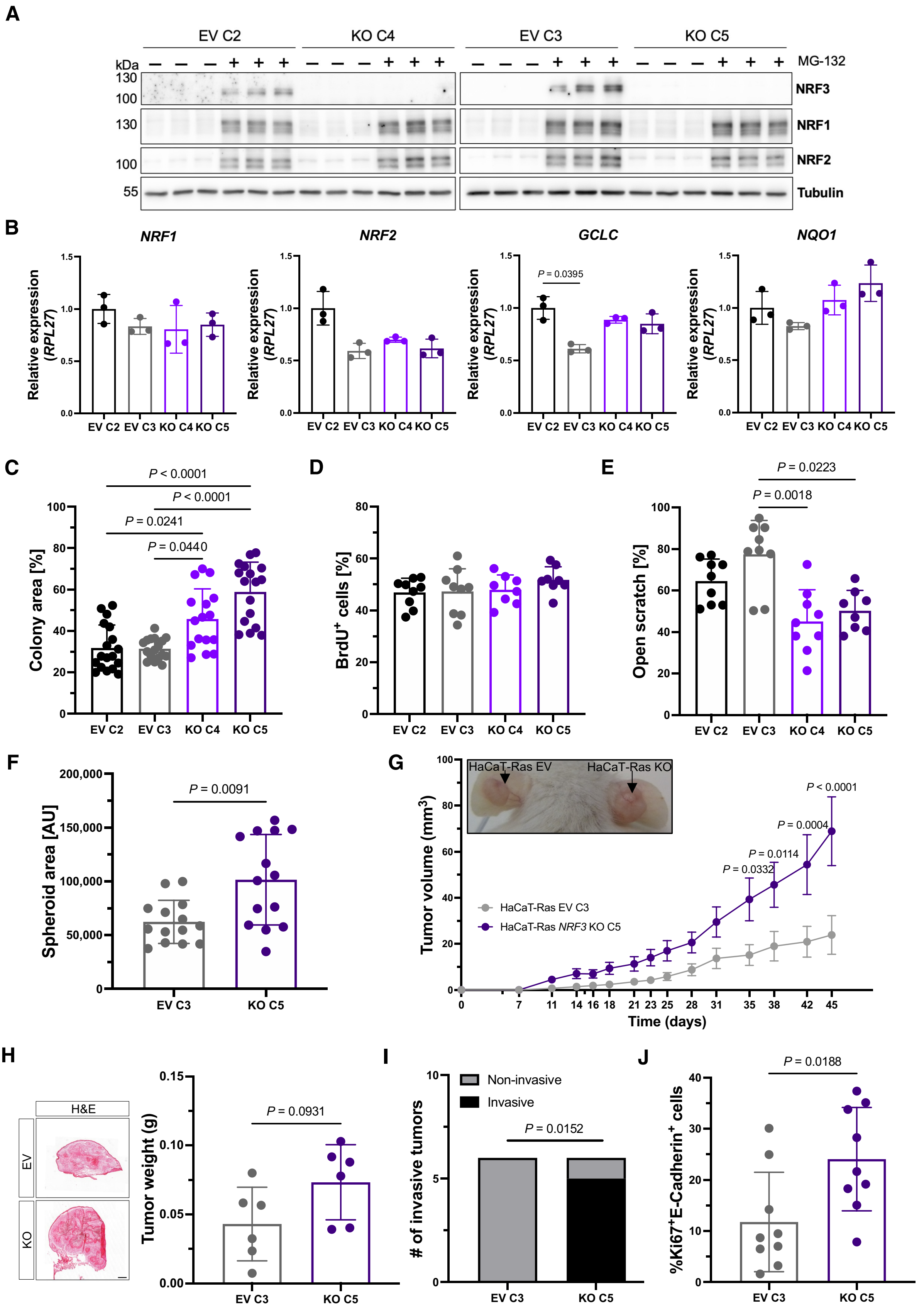
A
EV C2
KO C4
EV C3
KO C5
kDa
MG-132
130
100
NRF3
130
NRF1
100
NRF2
55
Tubulin
B
NRF1
NRF2
GCLC
NQO1
Relative expression (RPL27)
P = 0.0395
EV C2 EV C3 KO C4 KO C5
C
P < 0.0001
P < 0.0001
P = 0.0241
P = 0.0440
Colony area [%]
D
BrdU+ cells [%]
E
P = 0.0223
P = 0.0018
Open scratch [%]
F
P = 0.0091
Spheroid area [AU]
EV C3
KO C5
G
HaCaT-Ras EV
HaCaT-Ras KO
P < 0.0001
P = 0.0004
P = 0.0114
P = 0.0332
HaCaT-Ras EV C3
HaCaT-Ras NRF3 KO C5
Tumor volume (mm3)
Time (days)
H
H&E
EV
KO
P = 0.0931
Tumor weight (g)
I
Non-invasive
Invasive
P = 0.0152
# of invasive tumors
J
P = 0.0188
%Ki67+E-Cadherin+ cells

Figure EV3.

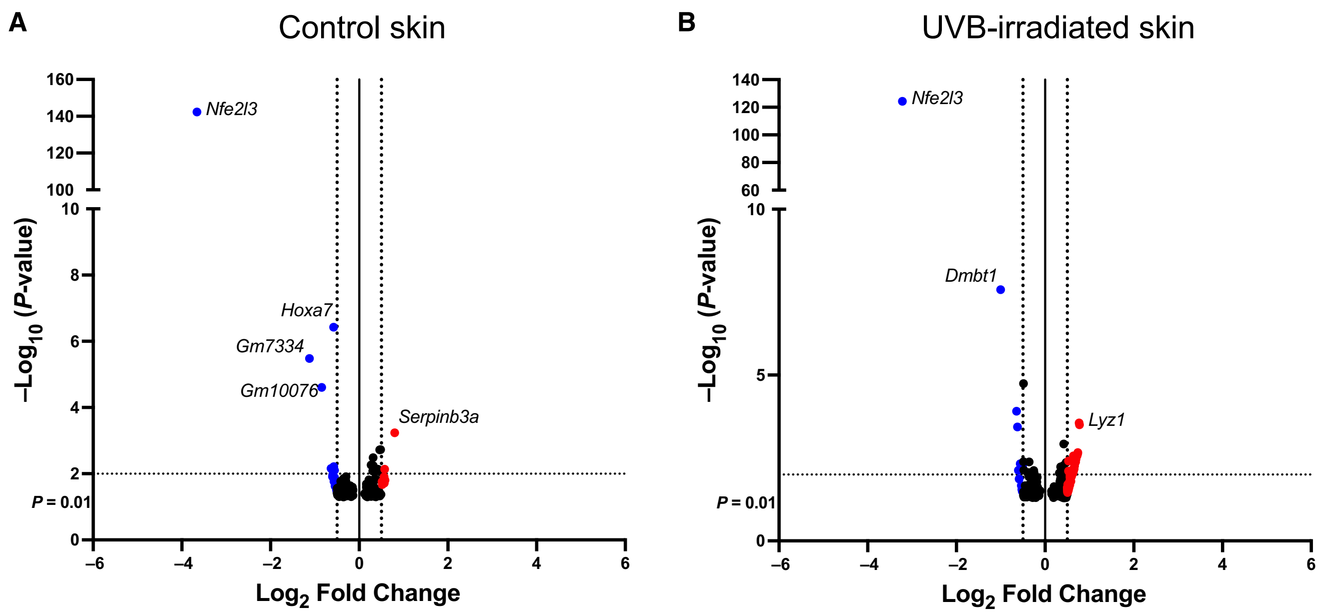

Figure EV4. Very few genes are differentially expressed in normal and UVB-irradiated epidermis of Nrf3-ko mice vs. wt littermates.

A, B Volcano plot of RNA-Seq data of isolated epidermis from untreated *Nrf3*-ko and wt mice (A) and at 24 h after irradiation with 100 mJ/cm^2 UVB (B). Significantly up- or downregulated transcripts are shown in red, respectively blue. A *P*-value < 0.05 (dotted black line at $-\log_{10}$(*P*-value) = 2) and a fold change > 0.5 (dotted black line at $\log_2$(FC) = $\pm$0.5) were used as a cutoff.

Figure EV5. HSPA5 is important for the tumor-suppressive effect of NRF3 in HaCaT-Ras cells.

A Top: Western blot using total cell lysates of SCC13 RescueNRF3 cells treated with DMSO, 20 ng/ml Dox or 1,000 ng/ml Dox for 24 h. The membrane was probed with antibodies against NRF3, HSPA5, and tubulin. Bottom: qRT-PCR using RNA from SCC13 RescueNRF3 cells treated with DMSO, 20 ng/ml Dox or 1,000 ng/ml Dox for 10 h for *NRF3* and *HSPA5* relative to *RPL27*. Mean expression in DMSO-treated cells was set to 1. *N* = 3 cultures per treatment group.

B Top: Western blot using total cell lysates of HaCaT-Ras EV and *NRF3*-KO cells treated with 10 µM MG-132 or vehicle (DMSO) for 6 h. The membrane was probed with antibodies against NRF3 and tubulin. Bottom: Quantification of the HSPA5/tubulin ratio (left) and qRT-PCR using RNA from HaCaT-Ras EV and *NRF3*-KO cells for *HSPA5* (right). Mean expression in EV cells was set to 1. Black or gray dots indicate data points from different EV cell lines; dark or light purple dots indicate data points from different KO cell lines. *N* = 6 cultures per genotype and treatment group (3 per cell line).

C, D Area of spheroids formed by HaCaT-Ras EV and *NRF3*-KO cells in single hanging drops cultured in the presence of 25 µM HA15 (C) or 5 µM YUM70 (D) or DMSO (vehicle). *N* = 23–28 hanging drops.

E Area of spheroids formed by HaCaT-Ras EV and *NRF3*-KO cells, which had been transfected with scrambled (scr) or HSPA5 siRNAs. *N* = 24–28 hanging drops.

F Quantification of the area of open scratch at 6 h in percentage of the original scratch area in confluent HaCaT-Ras control and *NRF3*-KO cells after transfection with scrambled (scr) or HSPA5 siRNAs. *N* = 6.

G Left: Bar graph showing volume of tumors formed by HaCaT-Ras EV and *NRF3*-KO cells in NOD/SCID mice at different time points after intratumoral injection of vehicle or HA15. Right: Three-way ANOVA output table. The relevant parameter is shown in bold. *N* = 6 tumors per genotype and treatment group, pooled from two independent experiments.

Data information: Bar graphs indicate mean ± SD. *P*-values were determined using Kruskal–Wallis test (A, B), 2-way ANOVA test (C–F), or 3-way ANOVA test (G).

 © 2023 The Authors

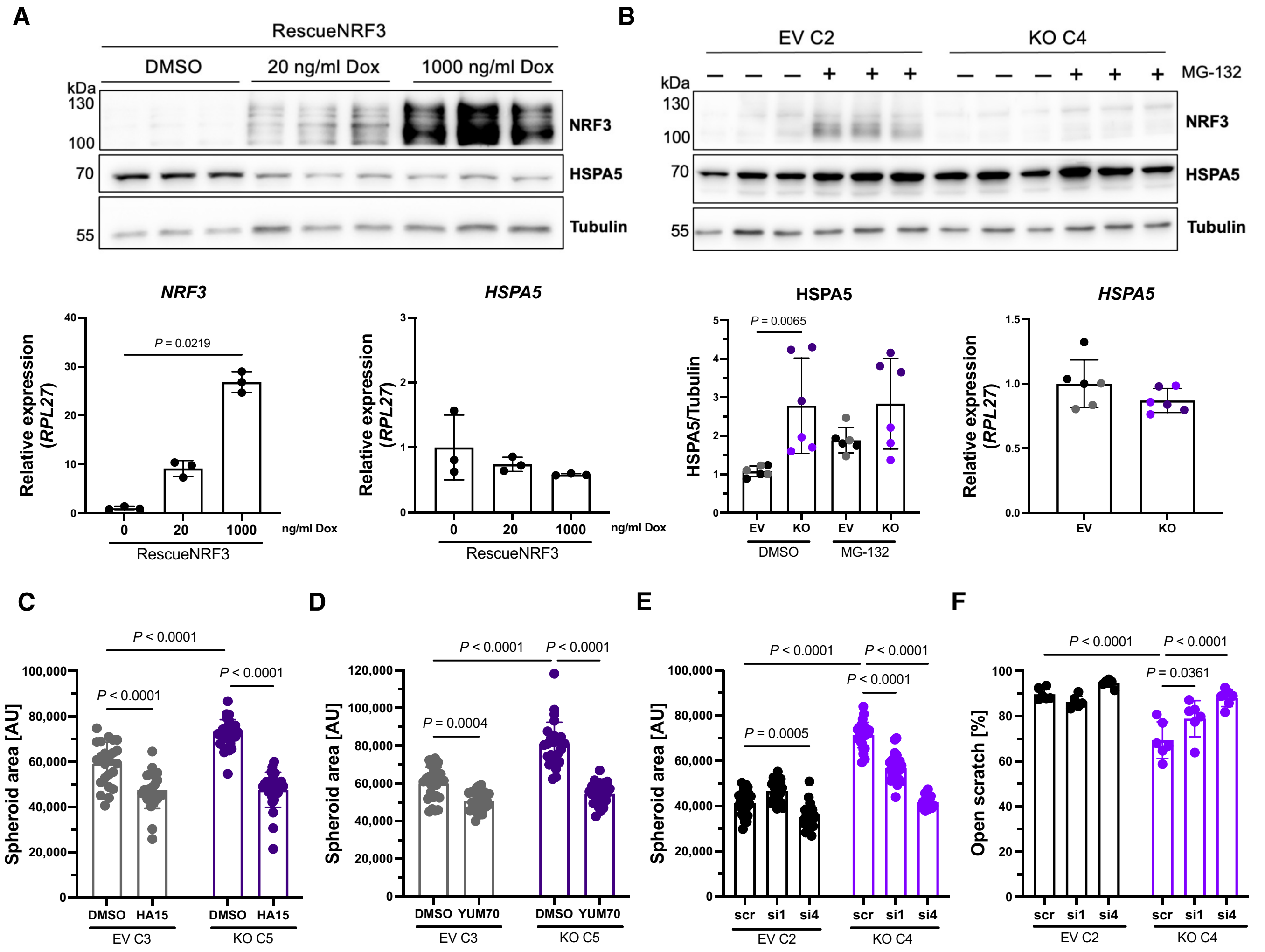

G

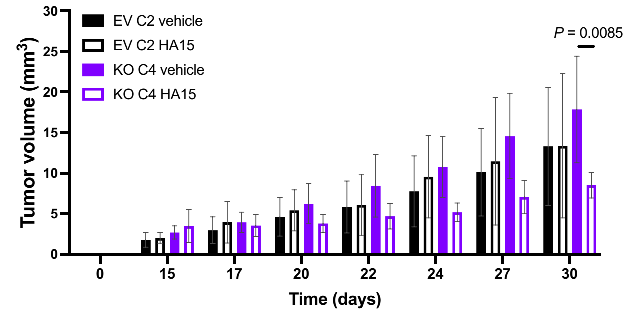

Source of Variation	% of total variation	P value	P value summary	Significant?
Time	53.63	<0.0001	****	Yes
Genotype	0.01958	0.7689	ns	No
Treatment	1.587	0.0089	**	Yes
Time x Genotype	0.2170	0.9953	ns	No
Time x Treatment	2.218	0.2081	ns	No
**Genotype x Treatment**	**3.501**	**0.0001**	***	**Yes**
Time x Genotype x Treatment	2.653	0.1183	ns	No

Figure EV5.
