# Supplementary material for: NRF3 suppresses squamous carcinogenesis, involving the unfolded protein response regulator HSPA5
Source: EMBO Mol Med. 2023 Oct 9;15(11):e17761. doi: 10.15252/emmm.202317761 (PMC10630885; doi:10.15252/emmm.202317761)
Supplement: Supplementary file 5 — PDF+ [file EMMM-15-e17761-s004.pdf]

Article

SOURCE DATA TRANSPARENT PROCESS OPEN ACCESS

EMBO Molecular Medicine

NRF3 suppresses squamous carcinogenesis, involving the unfolded protein response regulator HSPA5

Selina Gurri[1], Beat Siegenthaler[1], Michael Cangkrama[1], Gaetana Restivo[2], Marcel Huber[3], James Saliba[4], Reinhard Dummer[2], Volker Blank[4], Daniel Hohl[3] & Sabine Werner[1,*]

Abstract

Epithelial skin cancers are extremely common, but the mechanisms underlying their malignant progression are still poorly defined. Here, we identify the NRF3 transcription factor as a tumor suppressor in the skin. NRF3 protein expression is strongly downregulated or even absent in invasively growing cancer cells of patients with basal and squamous cell carcinomas (BCC and SCC). NRF3 deficiency promoted malignant conversion of chemically induced skin tumors in immunocompetent mice, clonogenic growth and migration of human SCC cells, their invasiveness in 3D cultures, and xenograft tumor formation. Mechanistically, the tumor-suppressive effect of NRF3 involves HSPA5, a key regulator of the unfolded protein response, which we identified as a potential NRF3 interactor. HSPA5 levels increased in the absence of NRF3, thereby promoting cancer cell survival and migration. Pharmacological inhibition or knockdown of HSPA5 rescued the malignant features of NRF3-deficient SCC cells *in vitro* and in preclinical mouse models. Together with the strong expression of HSPA5 in NRF3-deficient cancer cells of SCC patients, these results suggest HSPA5 inhibition as a treatment strategy for these malignancies in stratified cancer patients.

Keywords HSPA5; malignancy; NRF3; skin carcinogenesis; unfolded protein response
Subject Categories Cancer; Skin
DOI 10.15252/emmm.202317761 | Received 28 March 2023 | Revised 5 September 2023 | Accepted 15 September 2023 | Published online 9 October 2023
EMBO Mol Med (2023) 15: e17761

Introduction

Skin cancers are the most frequently diagnosed malignancies in humans, with one in three cancers diagnosed as a malignant skin tumor. The two major types of epithelial skin cancers, basal and squamous cell carcinomas (BCC and SCC), are diagnosed in 2–3 million people world-wide every year (Ciążyńska *et al*, 2021; Sung *et al*, 2021). They develop in a complex multistage process, which ends up with cells that have acquired an altered proliferative capacity, invasiveness, and possibly metastatic potential (Rundhaug & Fischer, 2010). SCCs are often preceded by precursor lesions, of which actinic keratosis (AK) is the most frequent one that develops at multiple sites, in particular in chronically sun-exposed skin of aged individuals (Werner *et al*, 2013).

The development and progression of epithelial skin cancers is controlled by various transcriptional regulators, which orchestrate the expression of downstream targets that affect proliferation, survival, and/or migration of tumor cells. A key player in cancer development is nuclear factor (erythroid-derived 2)-like 2 (NFE2L2; NRF2; Sporn & Liby, 2012; Rojo de la Vega *et al*, 2018). Loss of Nrf2 predisposes mice to different types of tumors, including skin SCCs, because of its important role in the detoxification of reactive oxygen species (ROS) and toxic chemicals (auf dem Keller *et al*, 2006; Xu *et al*, 2006; Sporn & Liby, 2012; Schmidlin *et al*, 2021). On the other hand, NRF2 is frequently hyperactivated in cancer cells, including SCC cells (Cescon *et al*, 2015; Oshimori *et al*, 2015), which promotes their malignancy and chemo−/radioresistance (Sporn & Liby, 2012; Schmidlin *et al*, 2021).

In contrast to the well-characterized NRF2, little is known about the target genes, mechanisms of action, and roles in cancer development and progression of the related NRF3 (NFE2L3) protein. NRF3 mRNA levels are strongly elevated in various types of cancers, and *NRF3* was identified as one of the most significantly mutated genes across 12 cancer types (Kandoth *et al*, 2013). The few published functional studies suggest a pro-tumorigenic function of NRF3 in most tissues, in particular in colorectal cancer, by controlling cancer cell growth and transcriptional regulation of proteasome genes (Aono *et al*, 2019; Bury *et al*, 2019; Waku *et al*, 2020). However, positive or negative correlation of high NRF3 mRNA levels with patient survival was described depending on the type of

1 Department of Biology, Institute of Molecular Health Sciences, ETH Zurich, Zurich, Switzerland
2 Department of Dermatology, University Hospital Zurich, Zurich, Switzerland
3 Service of Dermatology, Lausanne University Hospital and University of Lausanne, Lausanne, Switzerland
4 Lady Davis Institute for Medical Research, McGill University, Montreal, Canada
*Corresponding author. Tel: +41 44 633 3941; E-mail: sabine.werner@biol.ethz.ch

© 2023 The Authors. Published under the terms of the CC BY 4.0 license

cancer (see https://www.proteinatlas.org/ENSG00000050344-NFE2L3/pathology).

In the skin, NRF3 protein is highly expressed in basal keratinocytes (Braun *et al*, 2006; Siegenthaler *et al*, 2018). We previously showed that its loss protects keratinocytes *in vitro* and *in vivo* from apoptosis induced by UVB and other insults in an NRF2-independent manner (Siegenthaler *et al*, 2018), suggesting that the high levels of NRF3 in basal keratinocytes promote the elimination of mutated/damaged cells. Hence, we speculated about a possible role of NRF3 in skin carcinogenesis, as UVB radiation is the primary cause for skin cancer (Zambrano-Román *et al*, 2022).

Here we show that NRF3 protein expression is downregulated in invasively growing skin cancer cells of BCCs and SCCs in humans, suggesting a tumor-suppressive function in the skin. This was confirmed in functional 2D and 3D culture studies as well as in xenograft and chemically induced skin cancer models. Surprisingly, this activity is not mediated at the level of transcription, but results at least in part from NRF3-mediated destabilization of heat shock protein family A (HSP70 Member 5; HSPA5), a key regulator of the unfolded protein response (UPR) in the endoplasmic reticulum (ER). Upon loss of NRF3, HSPA5 levels increased, thereby promoting cancer cell survival and migration. These findings offer promising therapeutic opportunities for the treatment of epithelial skin tumors with HSPA5 inhibitors as demonstrated in this study using preclinical mouse models.

Results

NRF3 protein expression is downregulated in human non-melanoma skin cancer (NMSC)

To determine a potential role of NRF3 in NMSC, we analyzed its expression in BCCs and SCCs and in AK precursor lesions. NRF3 mRNA levels in whole skin/tumor samples did not differ between AK, BCC, and normal human skin, while an increase was seen in 3 out of 9 SCCs (Fig 1A).

A strong perinuclear staining of NRF3 was observed in basal keratinocytes of normal human epidermis and in the outer root sheath of hair follicles, while NRF3 was not or only hardly detectable in the dermis (Fig 1B). In contrast to the mRNA data, NRF3 staining was reduced in AK lesions, and a strong downregulation or even complete loss of NRF3 was observed in the invasively growing tumor cells of BCCs and SCCs (Fig 1B and C). This was confirmed by analysis of data presented in the Human Protein Atlas, which revealed that 8 out of 12 epithelial skin cancers had lost NRF3 in the tumor cells. Three tumors showed weak staining for NRF3, and only one tumor had medium expression similar to keratinocytes of normal skin (Fig 1D; https://www.proteinatlas.org/ENSG00000050344-NFE2L3/pathology/skin+cancer).

Together, these results identify NRF3 downregulation at the protein level as a hallmark of invasively growing skin cancer cells *in vivo*. Consistently, analysis of publicly available data revealed that amplification of the *NRF3* gene, which is frequent in other tumors, does not occur in NMSCs. Rather, they exclusively show mutations, which were, however, not further defined (Fig 1E).

Loss of Nrf3 promotes growth and malignant conversion of chemically induced skin tumors

To determine if the loss of NRF3 in skin cancers is functionally relevant, we performed a two-stage skin carcinogenesis study in mice with a global *Nrf3* knockout (*Nrf3*-ko mice), which do not show phenotypic abnormalities under homeostatic conditions (Derjuga *et al*, 2004). Tumors were induced by a single topical treatment with the mutagen 7,12-dimethylbenzo(a)anthracene (DMBA), followed by weekly topical treatment with the tumor promoter 12-O-tetradecanoylphorbol-13-acetate (TPA). Fifty percent of the mice were sacrificed at the end of the 26-week TPA treatment period, while the other 50% were only sacrificed 20 weeks later, allowing time for tumor progression (Abel *et al*, 2009). Although the overall number of tumors per mouse was low due to the general resistance of C57BL/6 mice to skin tumorigenesis (DiGiovanni *et al*, 1993), *Nrf3*-ko mice developed tumors earlier after DMBA treatment compared to their wild-type (wt) controls (Fig 2A–C). There was no more difference in tumor incidence and multiplicity after week 24 (Fig 2B and C). We noticed, however, that *Nrf3*-ko mice developed more mid- and in particular large-size tumors, while wt mice had mainly small tumors. This was confirmed by histomorphological analysis (Fig 2D). The larger tumor size was at least in part the result of increased keratinocyte proliferation in the papillomas (Fig 2E).

Most of the tumors in mice of both genotypes were histologically classified as acanthopapillomas. Importantly, however, the malignant conversion rate was strongly and significantly increased at week 30 and week 46 after DMBA treatment in *Nrf3*-ko compared to wt mice (Fig 2F). Almost 20% of the tumors in Nrf3-deficient mice were classified as malignant SCCs at week 30 and 30% at week 46, while the malignant conversion rate in wt mice did not exceed 5%.

There were no alterations in the number of dermal and epidermal T cells ($CD3^+$), neutrophils ($Ly6G^+$), and mast cells (toluidine blue [TB^+]; Fig EV1A–D), but functional differences in immune cells cannot be excluded. Although the experiment was performed in mice with a global *Nrf3* knockout, the highly predominant expression of NRF3 in keratinocytes of human skin (Fig 1B) and of mouse skin (Siegenthaler *et al*, 2018) and the low or even undetectable expression of *Nrf3/Nfe2l3* in different types of immune cells of healthy mice (http://rstats.immgen.org/Skyline/skyline.html) suggest that Nrf3 acts mainly in a cell autonomous manner in keratinocytes during tumor progression. Therefore, we next analyzed the functional consequences of NRF3 deficiency in SCC cells.

Loss of NRF3 promotes malignancy of SCC13 cells *in vitro* and in 3D organotypic skin cultures

To study the consequences of NRF3 deficiency in human SCC cells, we performed CRISPR/Cas9-mediated knockout of *NRF3* in the weakly malignant human SCC13 cell line (Rheinwald & Beckett, 1981). These cells express NRF3, which was stabilized by the proteasome inhibitor MG-132 (Fig 3A). Following clonal expansion, the knockout was confirmed by Western blot analysis (Fig 3A). SCC13 cells, which had been transduced with empty vector (EV) lacking a single guide (sg)RNA and which were also clonally expanded, were used as controls. There was no compensatory increase in NRF1 or NRF2 mRNA or protein levels as shown by qRT-PCR (Appendix Fig S1A) or Western blot analysis of lysates

 © 2023 The Authors

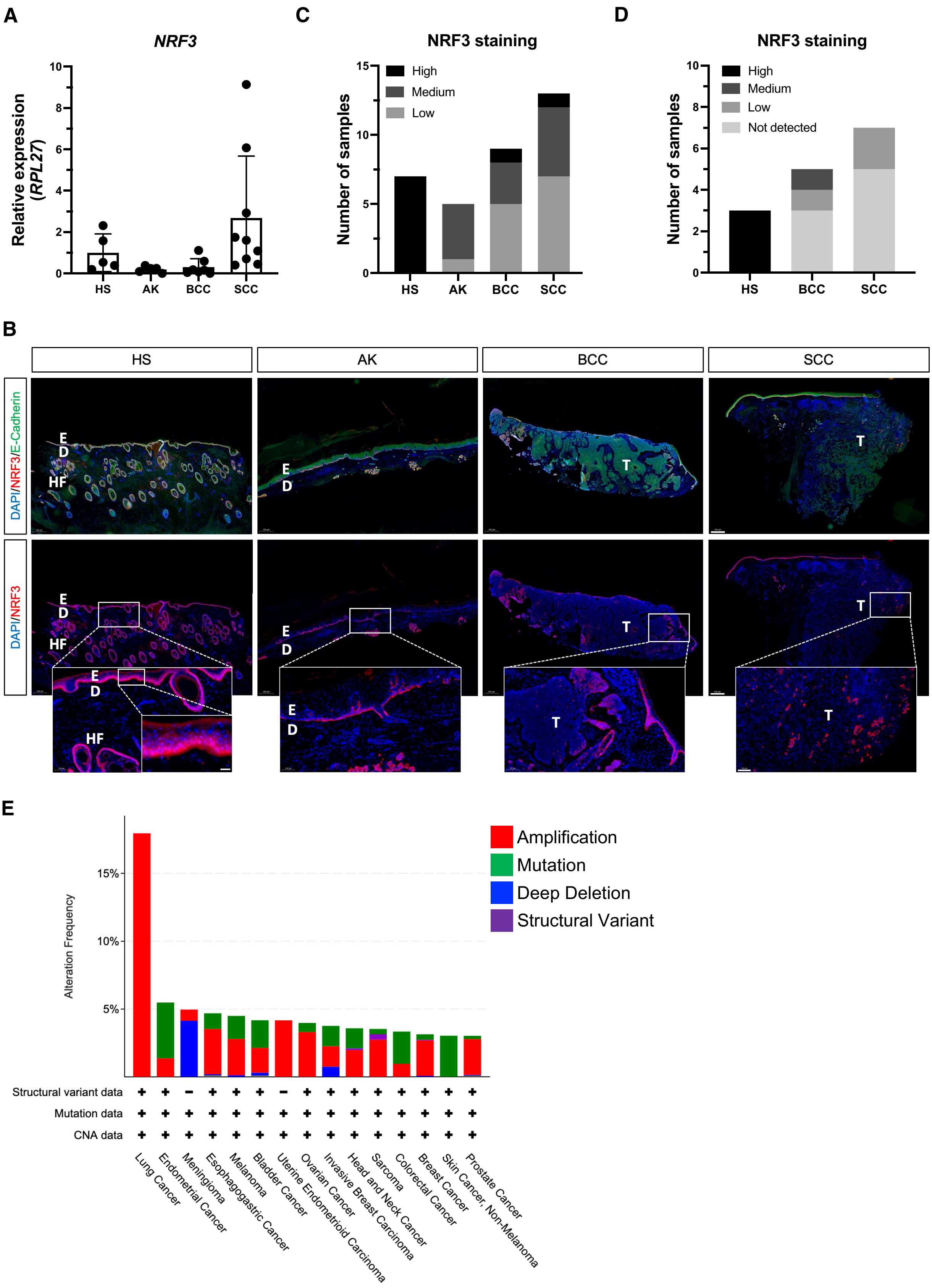
A
NRF3
Relative expression (RPL27)
HS
AK
BCC
SCC
C
NRF3 staining
Number of samples
High
Medium
Low
D
NRF3 staining
Not detected
B
DAPI/NRF3/E-Cadherin
DAPI/NRF3
E
D
HF
T
E
Amplification
Mutation
Deep Deletion
Structural Variant
Alteration Frequency
Structural variant data
Mutation data
CNA data
Lung Cancer
Endometrial Cancer
Meningioma
Esophagogastric Cancer
Melanoma
Bladder Cancer
Uterine Endometrioid Carcinoma
Ovarian Cancer
Invasive Breast Carcinoma
Head and Neck Cancer
Sarcoma
Colorectal Cancer
Breast Cancer
Skin Cancer, Non-Melanoma
Prostate Cancer

Figure 1.

◀ **Figure 1. Downregulation of NRF3 protein in epithelial human skin cancer.**

A qRT-PCR for *NRF3* relative to *RPL27* using RNA from normal human skin (HS), Actinic keratosis (AK), basal cell carcinoma (BCC), and squamous cell carcinoma (SCC). *N* = 5 (NS and AK), *N* = 6 (BCC), and *N* = 9 (SCC) biopsies from different individuals. Mean expression in HS was set to 1.

B Representative NRF3 immunofluorescence stainings of sections from HS, AK, BCC, or SCC using antibodies against NRF3 (red) and E-Cadherin (green). Nuclei were counterstained with Hoechst (blue). Scale bar: 500 μm. The areas indicated with a rectangle are shown at higher magnification in the indent. Scale bar: 100 μm, respectively, 20 μm. D, dermis; E, epidermis; HF, hair follicle; T, tumor.

C Summary of the expression of NRF3 in HS (respectively normal epidermis) and skin cancers. Samples were classified into those with high, medium, or low NRF3 staining intensity. *N* = 7 (HS), *N* = 5 (AK); *N* = 8 (BCC), and *N* = 13 (SCC) biopsies from different individuals.

D NRF3 staining intensity of sections from 12 NMSC samples in comparison to HS (respectively normal epidermis) according to data from the Human Protein Atlas (https://www.proteinatlas.org/ENSG00000050344-NFE2L3/pathology/skin+cancer).

E Graph showing alteration frequency of *NRF3* across different tumor types. Data from https://www.cbioportal.org by querying for *NFE2L3* and including curated non-redundant data sets. Min. # of total cases was set to 10 and min. % of altered cases to 3%. For datasets see https://www.cbioportal.org/datasets.

Data information: Bar represents mean ± standard deviation (SD).
Source data are available online for this figure.

from EV and *NRF3*-KO cells cultured in the presence or absence of MG-132, which stabilizes the NRF transcription factors (Fig 3A). Consistently, expression of the NRF2 target genes *GCLC* (encoding glutamate cysteine ligase catalytic submit) and *NQO1* (encoding NAD(P)H dehydrogenase (quinone) 1) was also not affected by the *NRF3* knockout (Appendix Fig S1A). Loss of NRF3 had no or only a very minor effect on the viability of SCC13 cells under normal culture conditions (Appendix Fig S1B and C). However, it strongly promoted clonogenicity and migration of the cancer cells, whereas their proliferation was not significantly affected (Fig 3B–D). We also generated SCC13 *NRF3*-KO cells with doxycycline (Dox)-inducible re-expression of NRF3 (designated RescueNRF3 cells). The recombinant NRF3 protein was efficiently expressed in these cells when 20 ng/ml Dox or more were used (Fig 3E). Under these conditions, the small variant (C-form) was detectable in the nucleus, even without MG-132 treatment (Fig 3F). Similar to the endogenous NRF3, three isoforms of the recombinant NRF3 were detected, including the N-glycosylated A variant, the ER-resident B variant, and the smallest (C) variant, which is present in the nucleus (Nouhi *et al*, 2007; Figs 3E and EV2A).The high cell migration rate was reduced by Dox-inducible expression of recombinant NRF3 (Fig 3G). When grown in hanging drops, the *NRF3*-KO cells developed much larger spheroids (Fig 3H). *Vice versa*, Dox-inducible overexpression of NRF3 in wt SCC13 cells reduced the migration rate and spheroid size (Fig EV2A–E). The latter was also reduced by Dox-inducible re-expression of recombinant NRF3 in the *NRF3*-KO cells (Fig 3I). We also generated skin equivalents in which keratinocytes are seeded on top of primary human foreskin fibroblasts, which had previously deposited their own extracellular matrix (Berning *et al*, 2015). NRF3-deficient SCC13 cells invaded into the dermal equivalent, while this was not observed with the parental cells (Fig 3J and K).

The positive effect of loss of NRF3 on cell migration and spheroid formation without alterations in NRF1 and NRF2 expression and activation was reproduced with the malignantly transformed HaCaT cell derivative, HaCaT-Ras (Ryle *et al*, 1989; Fig EV3A–F).

Loss of NRF3 enhances tumorigenesis and invasiveness in a xenograft mouse model

We next determined the tumorigenic potential of the SCC13 *NRF3*-KO cells in a xenograft mouse model. Control (EV C1 and EV C2) cells were injected into the left ear and the KO cells (KO C3 and KO C4) into the right ear of immunodeficient NOD/SCID mice. The tumors formed by *NRF3*-KO cells were larger compared to those formed by control cells as reflected by the larger tumor volume over time and the higher tumor weight at the day of sacrifice (Fig 4A and B). Histologically, the NRF3-deficient tumors showed high malignancy and invasive growth, resulting in penetration of the cancer cells through the ear cartilage (Fig 4B and C). Ki67 staining revealed increased tumor cell proliferation in NRF3-deficient tumors (Fig 4D). Similar results were obtained with NRF3-deficient HaCaT-Ras cells (Fig EV3G–J). Overexpression of NRF3 in SCC13 cells did not affect tumor growth in the xenograft model (Appendix Fig S2A–D).

NRF3 mainly acts in the endoplasmic reticulum of keratinocytes

To identify genes that are controlled by NRF3 in skin cancer cells, we performed RNA sequencing (RNA-seq) of RescueNRF3 cells, introduced in Fig 3. Cells were treated with DMSO or Dox for 10 h to allow expression of recombinant NRF3 and translocation of the shorter C variant, which lacks the ER targeting domain (Nouhi *et al*, 2007), into the nucleus (Fig 3F). Surprisingly, very few differentially expressed genes with a *P*-value of at least 0.05 and $\log_2$ fold change of at least 0.5 were identified upon re-expression of NRF3 in the *NRF3*-KO cells (Fig 5A), and the FPKM (fragments per kilobase per million mapped fragments) values were extremely low. The only exception was *NRF3* (*NFE2L3*) itself, demonstrating the validity of the data. We were also not able to validate the few candidate genes, including glutathione S-transferase A4 (*GSTA4*) and proline rich 7, synaptic (*PRR7*), by qRT-PCR (Fig 5B). These findings suggest that NRF3 does not have a major role as a transcription factor in SCC cells under these experimental conditions. Consistently, RNA-seq of isolated epidermis from *Nrf3*-ko mice and wt littermates also identified very few genes that were differentially expressed—both in untreated mice and at 24 h after irradiation with 100 mJ/cm^2 UVB (Fig EV4A and B), when the UV-protective effect of Nrf3 deficiency was clearly visible (Siegenthaler *et al*, 2018).

Because of the minimal effect of NRF3 on gene transcription in keratinocytes and the predominant localization of NRF3 outside the nucleus in basal keratinocytes *in vivo* (Fig 1B), we speculated that NRF3 may affect skin tumorigenesis by different mechanisms. Therefore, we searched for putative NRF3-binding partners using the proximity-dependent biotin identification (BioID) technique, which allows the labeling of proteins in close proximity to the protein of interest (Hesketh *et al*, 2017). For this purpose, we generated HaCaT and SCC13 cell lines with Dox-inducible expression of

 © 2023 The Authors

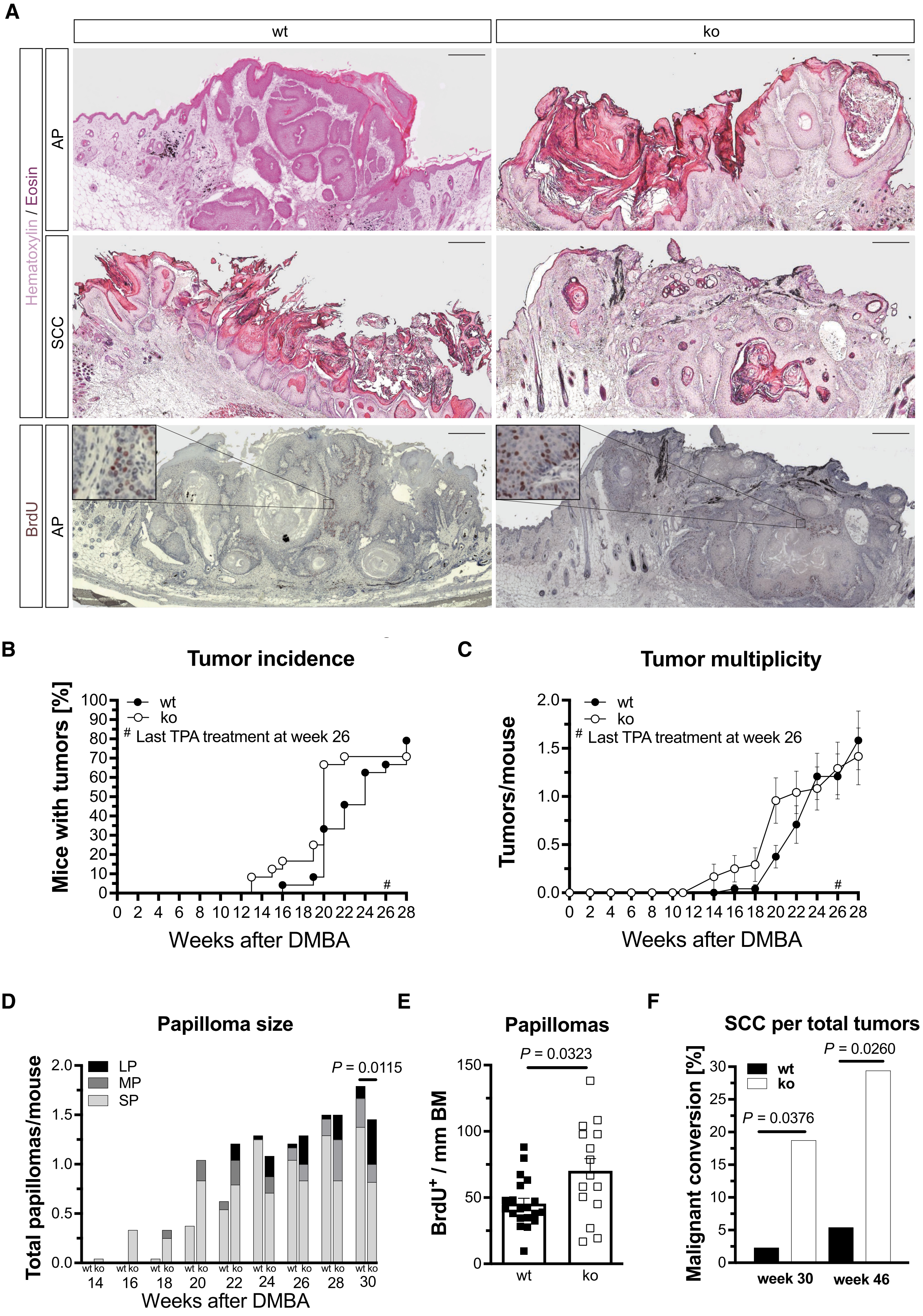
A
wt
ko
Hematoxylin / Eosin
AP
SCC
BrdU
AP
B
Tumor incidence
Mice with tumors [%]
wt
ko
Last TPA treatment at week 26
Weeks after DMBA
C
Tumor multiplicity
Tumors/mouse
wt
ko
Last TPA treatment at week 26
Weeks after DMBA
D
Papilloma size
Total papillomas/mouse
LP
MP
SP
P = 0.0115
Weeks after DMBA
E
Papillomas
BrdU+ / mm BM
P = 0.0323
wt
ko
F
SCC per total tumors
Malignant conversion [%]
wt
ko
P = 0.0376
P = 0.0260
week 30
week 46

Figure 2.

Figure 2. Loss of Nrf3 in keratinocytes promotes malignant progression of chemically induced skin tumors.

Nrf3-ko and wild-type (wt) mice were treated once topically with the mutagen DMBA, followed by weekly treatment with TPA for 25 weeks.

- A Histological analysis of tumors stained with hematoxylin/eosin (H&E; upper two panels) or analyzed by immunohistochemistry for BrdU incorporation (lower panel). Scale bars: 500 μm. The areas indicated by squares are shown at higher magnification in the indent. AP, acanthopapilloma; SCC, squamous cell carcinoma.
- B, C Kaplan–Meier plots showing tumor incidence (B) and multiplicity (C). N = 24 wt and *Nrf3*-ko mice.
- D Size of papillomas; LP, large papilloma (bigger than 8 mm diameter); MP, medium papilloma (between 3 mm and 8 mm diameter); SP, small papilloma (smaller than 3 mm diameter). N = 24 wt and N = 22 *Nrf3*-ko mice.
- E BrdU-positive tumor cells/mm of basement membrane (BM) in papillomas. N = 20 tumors from 16 mice; N = 15 tumors from 14 *Nrf3*-ko mice.
- F Malignant conversion rate of tumors in DMBA/TPA-treated mice. N = 43 tumors from 24 wt mice and N = 32 tumors from 22 *Nrf3*-ko mice (week 30); N = 37 tumors from 16 wt mice and N = 17 tumors from 14 *Nrf3*-ko mice (week 46).

Data information: Graphs show absolute values (B, D, F) or mean and standard error of the mean (SEM) (C, E). *P*-values were determined by Fisher's exact test (D for comparison of large papillomas, F) or Mann–Whitney *U* test for middle and large tumors (E).
Source data are available online for this figure.

a BioID2-NRF3 fusion protein with an in-frame influenza virus hemagglutinin (HA)-tagged BioID2 moiety at the C-terminus. A C-terminal tag was chosen to prevent interference of a tag at the N-terminus with the integration of the protein into the ER membrane. The transduced cells efficiently expressed the fusion protein upon Dox treatment (Appendix Fig S3A). Biotinylated proteins were detected using horseradish peroxidase-coupled streptavidin (Appendix Fig S3A, left panel). Since NRF3 has a very short half-life (Chevillard & Blank, 2011), we expected to identify only very few interactors. Therefore, we also performed the BioID experiment with cells that had been treated with the proteasome inhibitor MG-132, and we indeed observed increased biotin signals under these conditions (Appendix Fig S3A, right panel). We confirmed the ER and nuclear localization of NRF3-BioID2 fusion proteins by immunofluorescence staining (Appendix Fig S3B). Expression of the HA-tagged fusion protein reduced the migration rate of *NRF3*-KO cells (Appendix Fig S3C), demonstrating its proper function. Next, we performed the BioID screen in SCC13 cells and in the non-malignant HaCaT cell line upon treatment of the cells with Dox or vehicle (Fig 5C) and in the presence or absence of MG-132. All hits are listed in Dataset EV1 and EV2. The putative interaction partners were analyzed using "Significance Analysis of INTeractome" (SAINT) and filtered for proteins with a Saint Probability (SP) score of ≥ 0.95. To further increase the stringency, only proteins detected in all three replicates and in both SCC13 and HaCaT cells were considered for validation. Using these criteria, we identified 34 potential NRF3 interactors (Fig 5D and E), which were present in the MG-132-treated group as well as in the non-treated group. These included all three small MAF proteins, which are known NRF3 interaction partners in the nucleus (Kobayashi *et al*, 1999; Fig 5E, marked in orange). Surprisingly, however, Ingenuity Pathway Analysis identified many proteins of the unfolded protein response (UPR) as putative NRF3 binding partners based on the BioID data (Fig 5F; upper panel). String analysis also showed that the UPR-associated proteins interact with each other (Fig 5F; lower panel).

Using co-immunoprecipitation experiments, we showed that the NRF3 fusion protein interacts with dishevelled 2 (Dvl2), heat shock protein A5 (HSPA5), and protein disulfide-isomerase A3 (PDIA3; Fig 5G). Additionally, proximity ligation assays verified the close proximity of the NRF3 fusion protein with PDIA3 (Fig 5H, upper panel) and of endogenous NRF3 with PDIA3 (Fig 5H, middle panel) and HSPA5 (Fig 5H, lower panel).

HSPA5 levels are enhanced in the *NRF3*-KO cells and in invasively growing skin cancer cells

The close proximity of NRF3 with proteins involved in ER stress and the UPR pointed to a functional role of NRF3 in this pathway. To test this possibility, we treated wt and *NRF3*-KO cells with the ER stress inducer tunicamycin. Loss of NRF3 strongly protected from tunicamycin-induced apoptosis as shown by cleaved caspase-3 staining (Fig 6A). This is consistent with the protection of Nrf3-deficient normal mouse keratinocytes from apoptosis induced by UVB irradiation (Siegenthaler *et al*, 2018), which also induces ER stress and activates the UPR in keratinocytes (Farrukh *et al*, 2014; Seo *et al*, 2020). The protection of NRF3-deficient SCC13 cells from tunicamycin-induced apoptosis was associated with a less pronounced increase in the apoptotic markers CHOP and cleaved PARP (Fig 6B). In control cells, the amounts of NRF3 increased in response to tunicamycin treatment. Concomitantly, processing of NRF1 occurred, but there was no difference in the amounts of NRF1 or its processing between control and *NRF3*-KO cells (Fig 6B). This finding suggests that the anti-apoptotic effect of NRF3 deficiency in SCC cells is not mediated by alterations of the related NRF1 protein. Remarkably, however, *NRF3*-KO cells showed higher levels of HSPA5, also called glucose-regulated protein 78 (GRP78) or binding immunoglobulin protein (BiP; Fig 6B)—one of the proteins found in the BioID screen with the NRF3-HA fusion protein. We focused the further functional analysis on this protein, because (i) it is the master regulator of the UPR, which is activated in many different cancers (Wang *et al*, 2009), (ii) it was found in the BioID screen with an SP of 1, (iii) its close proximity with NRF3 was verified for the endogenous proteins (Fig 5H); and (iv) overexpression of HSPA5 promoted malignancy in other types of cancer (Wang *et al*, 2009; Wang & Kaufman, 2014). Loss of NRF3 seems to stabilize HSPA5, because HSPA5 protein levels were also higher in *NRF3*-KO cells under homeostatic conditions, while HSPA5 mRNA levels were not significantly affected (Fig 6C). As expected, Dox-induced expression of NRF3 in the RescueNRF3 cells reduced the amounts of HSPA5, but a strong NRF3 overexpression had no additional effect (Fig EV5A).

HSPA5 levels were also increased in the 3D organotypic cultures generated with *NRF3*-KO cells, especially at the invasive front (Fig 6D, areas indicated with white arrows), as well as in the non-hyperkeratotic epithelial area of DMBA/TPA-induced tumors of *Nrf3*-ko vs. wt mice (Appendix Fig S4). Most importantly, HSPA5 was strongly expressed in human BCCs and SCCs, in particular in

© 2023 The Authors

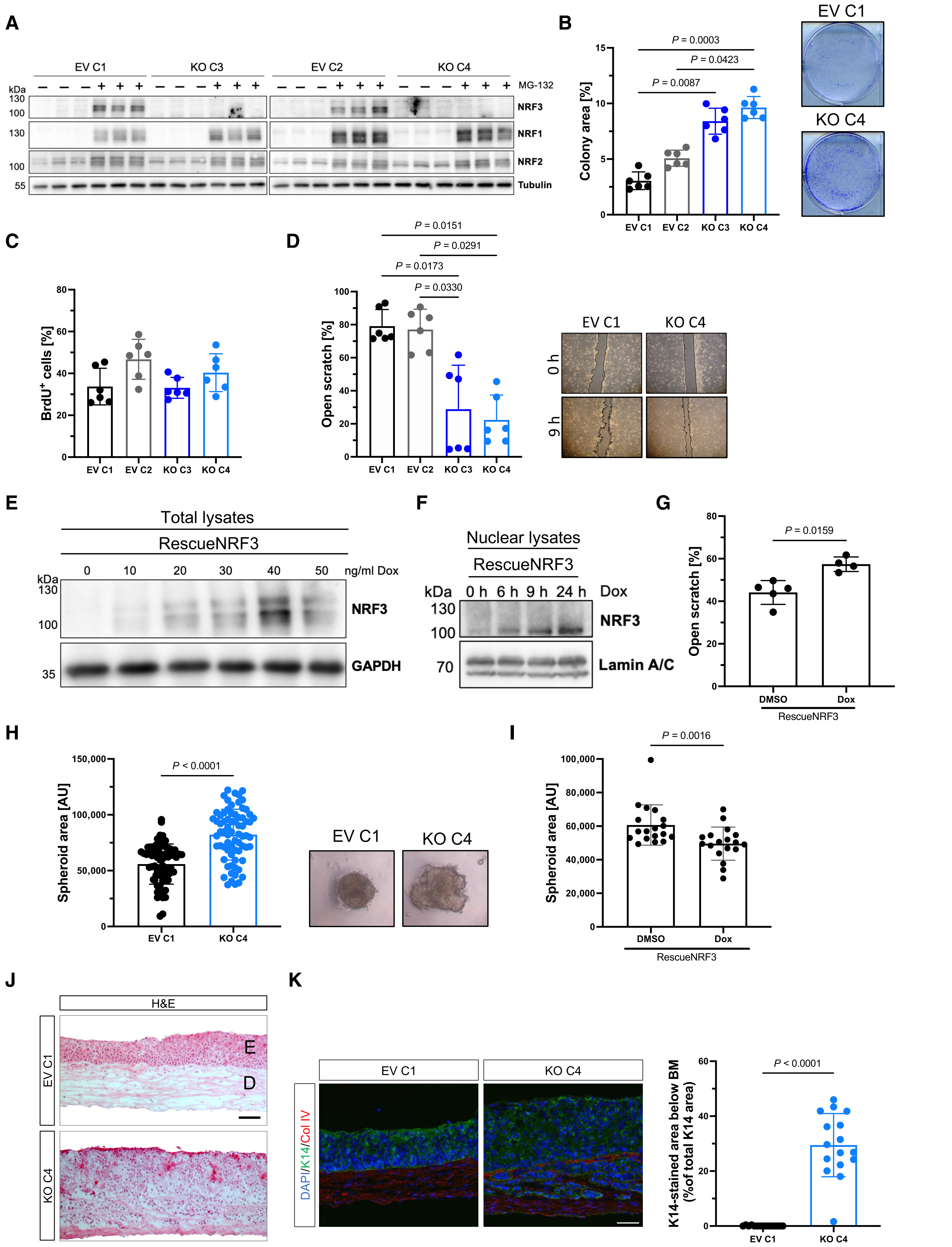
A
EV C1
KO C3
EV C2
KO C4
MG-132
kDa
130
100
NRF3
NRF1
NRF2
55
Tubulin
B
Colony area [%]
P = 0.0003
P = 0.0423
P = 0.0087
EV C1
EV C2
KO C3
KO C4
C
BrdU+ cells [%]
D
Open scratch [%]
P = 0.0151
P = 0.0291
P = 0.0173
P = 0.0330
0 h
9 h
E
Total lysates
RescueNRF3
0
10
20
30
40
50
ng/ml Dox
GAPDH
35
F
Nuclear lysates
RescueNRF3
0 h
6 h
9 h
24 h
Dox
70
Lamin A/C
G
P = 0.0159
DMSO
Dox
H
Spheroid area [AU]
P < 0.0001
I
P = 0.0016
J
H&E
E
D
K
DAPI/K14/Col IV
K14-stained area below BM (%of total K14 area)
P < 0.0001

Figure 3.

Figure 3. Loss of NRF3 promotes malignancy of SCC13 cells.

A Western blot of lysates of transduced SCC13 cells (two clones of empty-vector [EV]-transduced cells [EV C1; EV C2] and of *NRF3*-KO cells [KO C3; KO C4]), which had been treated with the proteasome inhibitor MG-132 or vehicle, using antibodies against NRF3, NRF1, NRF2, and tubulin (loading control).

B Clonogenicity of SCC13 EV and *NRF3*-KO cells. *N* = 6 cultures per cell line. Left: Quantification of the area covered by colonies relative to the whole area of the well. Right: Representative pictures of colonies after 7 days.

C BrdU incorporation into SCC13 EV and *NRF3*-KO cells. *N* = 6.

D Left: Quantification of the area of open scratch at 9 h post scratch wounding in percentage of the original scratch area (left). Right: Representative pictures of scratch-wounded SCC13 EV and *NRF3*-KO cells at 0 or 9 h (right). *N* = 6.

E Western blot of total lysates from RescueNRF3 cells, cultured in the presence of vehicle or different concentrations of Dox to induce NRF3 expression, using antibodies against NRF3 and GAPDH (loading control). A 6 h treatment with 10 μM MG-132 was performed to increase the signal intensity.

F Western blot of nuclear lysates of SCC13 RescueNRF3 cells treated with vehicle or 20 ng/ml Dox for 6, 9, or 24 h, using antibodies against NRF3 and the nuclear marker lamin A/C.

G Quantification of the area of open scratch at 9 h post scratch wounding in percentage of the original scratch area in SCC13 RescueNRF3 cells treated with 20 ng/ml Dox or vehicle (DMSO). *N* = 4–5.

H Left: Area of spheroids formed by SCC13 EV and *NRF3*-KO cells in single hanging drops. Right: Representative pictures of the spheroids. *N* = 70–71 hanging drops.

I Area of spheroids formed by SCC13 RescueNRF3 cells treated with 20 ng/ml Dox or vehicle (DMSO). *N* = 18 hanging drops.

J Representative images of H&E-stained sections from 3D skin equivalents formed by primary human skin fibroblasts and either EV or *NRF3*-KO cells. Scale bar: 100 μm. D, dermal equivalent; E, epidermal equivalent.

K Representative images of sections from SCC13 EV and *NRF3*-KO organotypic cultures immunostained for keratin 14 (K14; green) and collagen IV (red) combined with Hoechst staining (blue), and quantification of the K14-positive area below the basement membrane (BM). *N* = 3 independent cultures, *n* = 5 sections. Scale bar: 100 μm.

Data information: Graphs show mean ± SD. *P*-values were determined using Mann–Whitney test (G, H, I, K) or Kruskal–Wallis (B–D).
Source data are available online for this figure.

the invasively growing skin cancer cells, which showed low or no expression of NRF3 (Fig 6E, areas indicated by white dotted lines). These results point to an important role of HSPA5 in the tumor-suppressing effect of NRF3 in the skin.

HSPA5 inhibition or knock-down reverts the high malignancy of NRF3-deficient cancer cells

To determine if the increased HSPA5 levels affect the tumorigenic features of SCC13 cells, we generated hanging drop spheroids in the presence of the HSPA5 inhibitors HA15 (Cerezo *et al*, 2016) or YUM70 (Samanta *et al*, 2021) or vehicle. Both inhibitors significantly reduced the spheroid size of *NRF3*-KO cells, while they had no or only a minimal effect on control cells (Fig 7A and B). This is consistent with findings obtained with melanoma cells, where HA15 was more effective when the concentration of HSPA5 was high (Cerezo *et al*, 2016). None of the inhibitors caused significant cell death at the concentration used for the spheroid assay, and there were no or only slight differences in survival between inhibitor-treated control and *NRF3*-KO cells (Appendix Fig S5A and B).

We also performed siRNA-mediated HSPA5 knock-down in control and NRF3-deficient SCC13 (Appendix Fig S6A and B) and HaCaT-Ras cells (Appendix Fig S6C and D). A 70–80% reduction of HSPA5 levels strongly reduced the spheroid size of *NRF3*-KO SCC13 cells, while it had no or only a slight effect on the control cells, which had been transduced with scrambled siRNA (Fig 7C). The enhanced migration of *NRF3*-KO SCC13 cells was also rescued by HSPA5 knock-down (Fig 7D). The effects of HSPA5 inhibition or knock-down were verified in HaCaT-Ras cells, where we also detected higher HSPA5 protein, but not mRNA levels in the *NRF3*-KO cells (Fig EV5B–F).

Finally, we performed a preclinical study and determined if treatment with HA15 affects tumor growth in the xenograft model (Fig 7E). HaCaT-Ras cells were used for this purpose because they form tumors more rapidly and efficiently compared to SCC13 cells. Indeed, injection of HA15 into growing tumors attenuated the growth of tumors formed by *NRF3*-KO HaCaT-Ras cells (Fig 7F), while it had no significant effect on the tumors formed by HaCaT-Ras control cells (Fig 7G). Three-way ANOVA analysis of the combined data from control and *NRF3*-KO cells confirmed that the effect of HA15 treatment differed significantly between genotypes (Fig EV5G; relevant parameter shown in bold). While there was a trend for the vehicle-treated *NRF3*-KO tumors to be larger than the vehicle-treated EV tumors during the treatment period, this difference was not significant, which is consistent with the data shown in Fig EV3G, where the difference between EV and *NRF3*-KO tumors only became significant after day 30. Ki67 staining revealed reduced proliferation in the HA15-treated tumors generated by *NRF3*-KO HaCaT-Ras cells, whereas HA15 even promoted proliferation in the tumors generated by HaCaT-Ras control cells (Fig 7H). Furthermore, it promoted apoptosis in the tumors formed by *NRF3*-KO HaCaT-Ras cells, but not in the tumors formed by control cells (Fig 7I). This difference demonstrates that HSPA5 inhibition or knock-down is mainly beneficial in NRF3-deficient cells.

In summary, pharmacological inhibition or knock-down of HSPA5 reverted the enhanced malignancy of *NRF3*-KO cells, demonstrating that the tumor-suppressive function of NRF3 depends on its binding partner HSPA5 and the reduction in the levels of this pro-tumorigenic UPR protein.

Discussion

We discovered a tumor-suppressive role of NRF3 in the skin, which involves its unexpected function as a regulator of the UPR. This is surprising, since several studies showed a pro-tumorigenic function of NRF3, which is consistent with the increased NRF3 mRNA levels in many types of human cancer. For example, NRF3 overexpression promoted tumorigenesis in the colon and in other tissues through enhancement of cell proliferation and proteasomal inactivation of tumor suppressors (Bury *et al*, 2019; Kobayashi, 2020). On the other hand, loss of Nrf3 predisposed mice to T cell lymphoblastic

 © 2023 The Authors

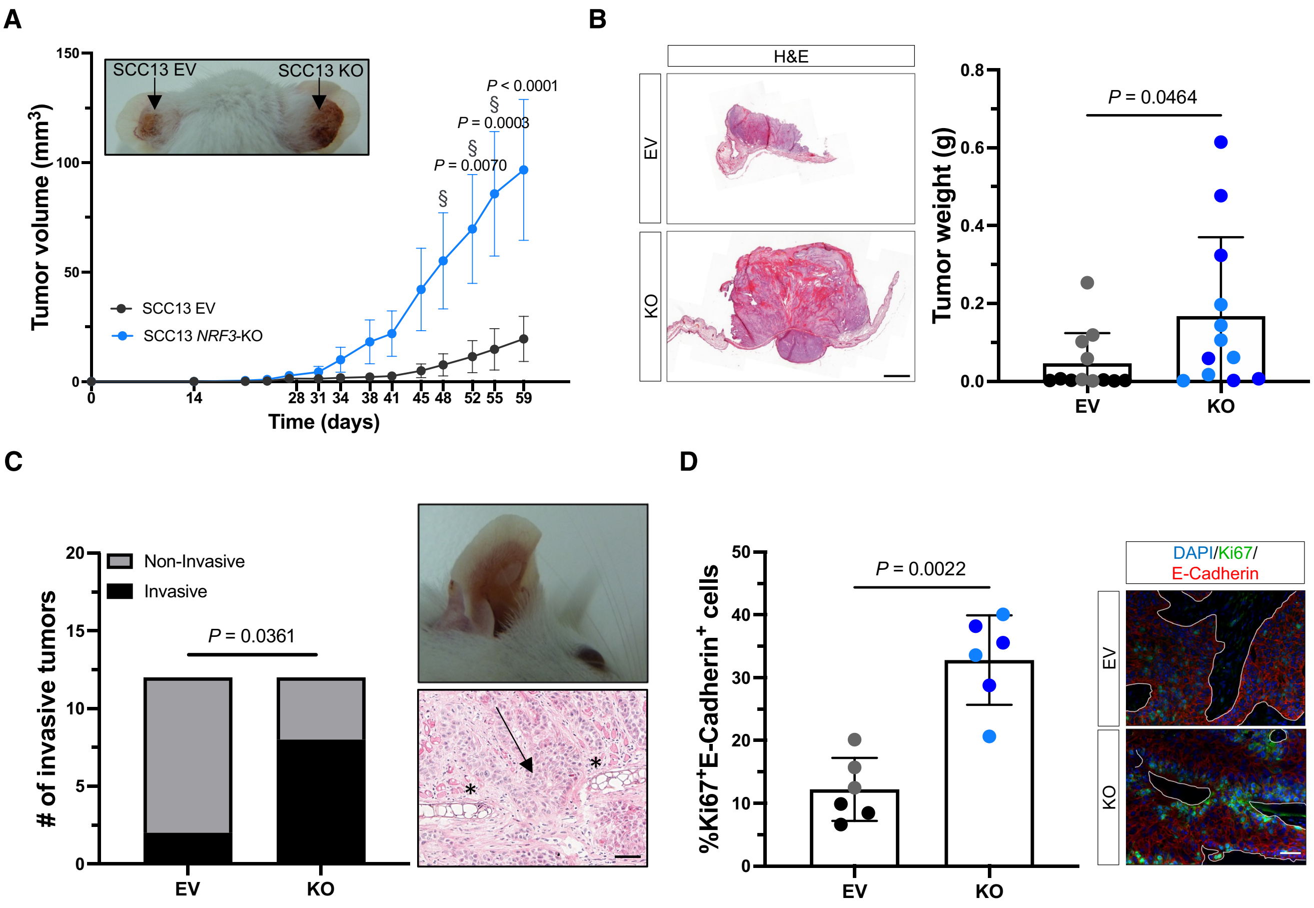

Figure 4. Loss of NRF3 enhances tumor formation and invasive growth of SCC13 cells in a xenograft mouse model.

A Representative photographs of ~ 8-week-old tumors (indicated by arrows) formed in NOD/SCID mice upon intradermal injection of SCC13 EV and *NRF3*-KO cells and tumor volume at different time points of tumor development. $N = 6$ tumors per cell line. Graph shows pooled results from two control (EV C1 and EV C2) and two KO clones (EV C3 and EV C4). § indicates time points when a mouse had to be sacrificed because the tumor had reached a size of almost 1 cm.

B Representative H&E stainings of sections from tumors formed by SCC13 EV or *NRF3*-KO cells at day 59, respectively day 55. Scale bar: 1,000 μm. Graph shows tumor weight at endpoint. $N = 6$ per cell line.

C Graph showing the percentage of tumors that had invaded through the basement membrane ($N = 6$) and a representative image of a mouse with an invasive tumor formed by the SCC13 *NRF3*-KO cells and histological staining of the invasive front (asterisk indicates cartilage; arrow indicates site of invasion). Scale bar: 50 μm.

D Quantification of Ki67/E-cadherin-positive cells in the xenograft tumors ($N = 6$ tumors [3 per cell line, $n = 5$–15 sections]) and representative images of sections from tumors formed by SCC13 EV and *NRF3*-KO cells stained for Ki67 (green), E-cadherin (red), and counterstained with Hoechst (blue). White lines indicate the borders to the stromal area. Scale bar: 50 μm.

Data information: Graph in (A) shows mean ± SEM. Graphs in (B) and (D) show mean ± SD. *P*-values were determined using 2-way ANOVA (A), Mann–Whitney *U* test (B, D) or Fisher's exact test (C). Black or gray dots indicate data points from different EV cell lines; dark or light blue dots indicate data points from different KO cell lines. Source data are available online for this figure.

lymphoma induced by benzo[a]pyrene (Chevillard *et al*, 2011) and suppressed breast cancer cell metastasis and proliferation (Sun *et al*, 2019). Hence, the function of NRF3 in cancer development and progression may depend on the tissue and possibly the tumor stage and the oncogenic stimulus.

We found a strong downregulation of NRF3 protein in NMSCs, while NRF3 mRNA levels were upregulated in some of the SCC samples. A decrease in NRF3 protein levels was also observed during the transition from benign melanocytic lesions to primary melanoma, while the mRNA levels concomitantly increased (Immonen *et al*, 2022). However, it is not clear if the transcripts detected in these tumors encode the wt or mutant proteins, as *NRF3* is one of 127 significantly mutated genes among 12 cancer types (Kandoth *et al*, 2013). In the future, it will be interesting to determine if the mutations in *NRF3* that occur in NMSC (see Fig 1E) result in reduced stability of the NRF3 protein. It is also possible that NRF3 mRNA is insufficiently translated in cancer cells or that the protein gets destabilized during tumor progression, e.g. as a consequence of hypoxia-induced ubiquitination and proteasomal degradation as previously shown for other tumor suppressor proteins, e.g. "deleted in breast cancer 1" (DBC1; Liu *et al*, 2022).

Independent of the mechanism underlying the downregulation of NRF3 protein, our results demonstrate that loss of NRF3 in SCC cells is functionally important and strongly increases their malignant features *in vitro* and in xenograft and chemically induced skin cancer models *in vivo*. However, we did not observe significant differences

© 2023 The Authors

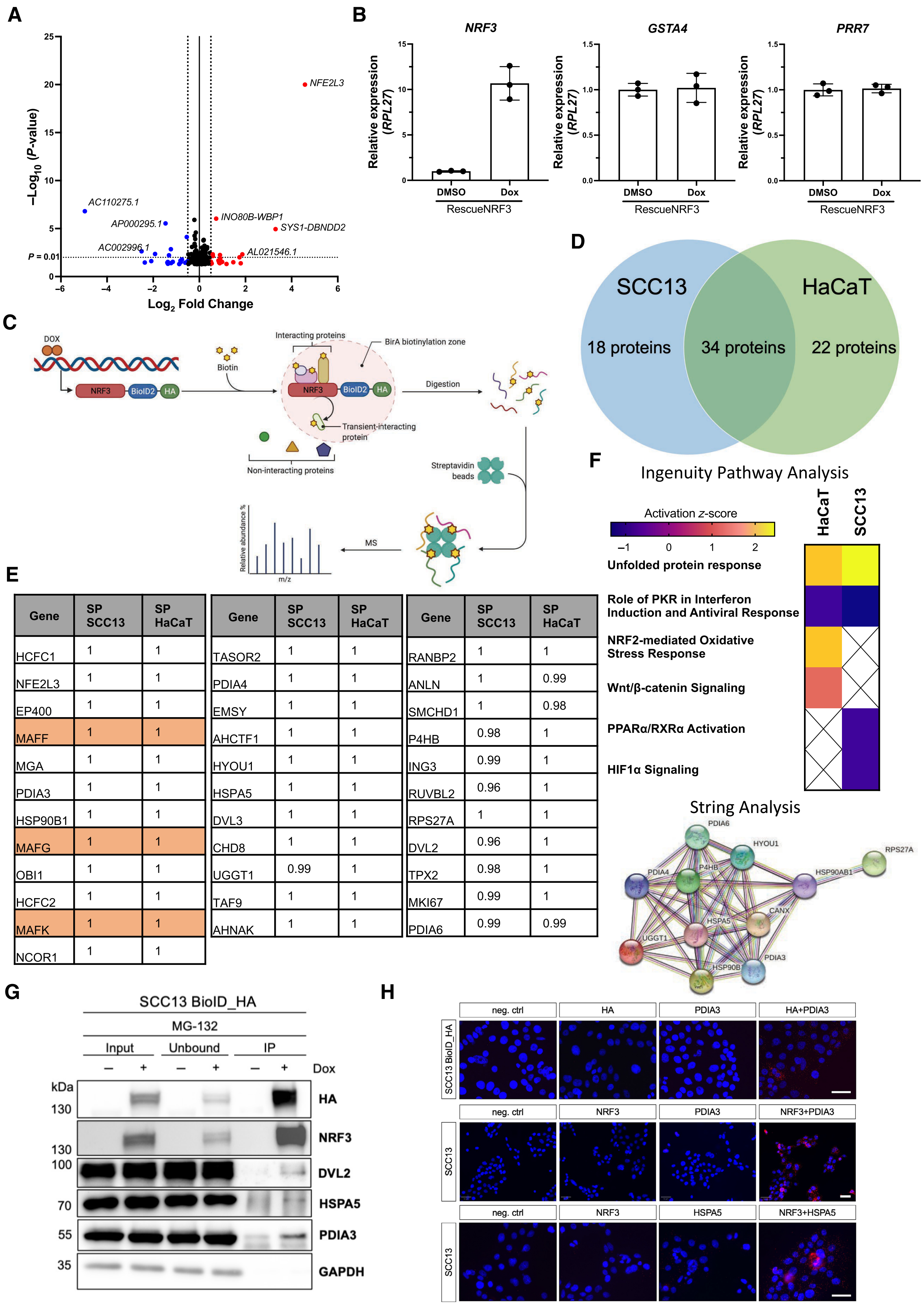

Gene	SP SCC13	SP HaCaT	Gene	SP SCC13	SP HaCaT	Gene	SP SCC13	SP HaCaT
HCFC1	1	1	TASOR2	1	1	RANBP2	1	1
NFE2L3	1	1	PDIA4	1	1	ANLN	1	0.99
EP400	1	1	EMSY	1	1	SMCHD1	1	0.98
MAFF	1	1	AHCTF1	1	1	P4HB	0.98	1
MGA	1	1	HYOU1	1	1	ING3	0.99	1
PDIA3	1	1	HSPA5	1	1	RUVBL2	0.96	1
HSP90B1	1	1	DVL3	1	1	RPS27A	1	1
MAFG	1	1	CHD8	1	1	DVL2	0.96	1
OBI1	1	1	UGGT1	0.99	1	TPX2	0.98	1
HCFC2	1	1	TAF9	1	1	MKI67	0.99	1
MAFK	1	1	AHNAK	1	1	PDIA6	0.99	0.99
NCOR1	1	1						

Figure 5.

© 2023 The Authors

Figure 5. NRF3 binds to proteins involved in the Unfolded Protein Response.

A Volcano plot of RNA-Seq data showing significantly up- (red) and downregulated (blue) transcripts in RescueNRF3 cells treated for 10 h with 20 ng/ml Dox vs. DMSO. A P-value < 0.05 (dotted black line at $-\log_{10}(P\text{-value}) = 2$) and a fold change > 0.5 (dotted black line at $\log_2(FC) = \pm 0.5$) were used as a cutoff.

B qRT-PCR using RNA from SCC13 RescueNRF3 cells treated for 10 h with 20 ng/ml Dox or vehicle (DMSO) for *NRF3*, *GSTA4*, and *PRR7* relative to *RPL27*. Graphs show mean ± SD. $N = 3$. Mean expression in DMSO-treated cells was set to 1.

C BioID proximity labelling strategy to identify potential NRF3 binding partners in SCC13 and HaCaT cells with Dox-inducible expression of HA-tagged NRF3-BioID fusion proteins. Created with BioRender.com.

D Venn diagram showing common putative NRF3 interactors in SCC13 and HaCaT cells.

E Table showing the 34 proteins that were identified as putative NRF3 interactors in SCC13 and HaCaT cells in the BioID screen and their respective Saint Probability (SP) scores. The small MAF proteins, which are known interactors of NRF3 in the nucleus, are highlighted in orange.

F Ingenuity pathway analysis showing the pathways in which the putative interaction partners are involved (top) and String analysis showing that many of these proteins also interact with each other (bottom).

G Co-immunoprecipitation of the HA-tagged NRF3-BioID fusion protein with Dvl2, HSPA5 and PDIA3 in transduced SCC13 cells cultured in the presence or absence of Dox (1 μg/ml) and in the presence of MG-132 (10 μM). GAPDH was used as loading control for the input and the unbound fraction.

H Top panel: Proximity ligation assay using transduced SCC13 cells expressing the HA-tagged NRF3-BioID fusion protein and antibodies against the HA epitope and PDIA3. Scale bar: 100 μm. Middle panel: Proximity ligation assay using SCC13 wt cells and antibodies against NRF3 and PDIA3. Scale bar: 50 μm. Lower panel: Proximity ligation assay using SCC13 wt cells and antibodies against NRF3 and HSPA5. Scale bar: 100 μm.

Source data are available online for this figure.

between control and NRF3-overexpressing SCC cells in the xenograft tumorigenesis studies. To achieve NRF3 overexpression, mice were systemically treated with Dox during the tumor formation phase via intraperitoneal injection, and it is unclear if sufficient amounts of Dox reached the ears and for how long it persisted to achieve a stable overexpression. Alternatively, a basal level of NRF3 may be sufficient to suppress tumorigenic features in SCC cells.

NRF3 has a known function as transcription factor and was reported to regulate some of the NRF2 target genes, but also genes that are not controlled by NRF2 (Pepe *et al*, 2010; Liu *et al*, 2019; Ibrahim *et al*, 2020; Waku *et al*, 2021; Saliba *et al*, 2022). Surprisingly, however, we could not identify significantly regulated genes in NRF3-deficient SCC13 cells after inducible expression of recombinant NRF3 or in the epidermis of UVB-irradiated wild-type vs. *Nrf3* knockout mice, although NRF3 was present in the nucleus under both experimental conditions. Although we cannot exclude that NRF3 regulates transcription under other experimental conditions or at other time points after its re-expression in *NRF3*-KO cells, these findings suggest that NRF3-mediated gene expression is suppressed in keratinocytes through yet unknown mechanisms. They also point to another mechanism of action of NRF3 in this cell type, which does not involve alterations in the expression or activity of NRF2 or NRF1. Indeed, we found that NRF3 is in close proximity and may even directly interact with many proteins involved in the UPR—both in non-tumorigenic keratinocytes and in SCC cells. The UPR is an adaptive signaling network, which consists of three main signal transducers that are activated upon accumulation of unfolded proteins, thereby restoring protein homeostasis (Hetz, 2012). However, if the ER stress cannot be resolved and if homeostasis is not restored, damaged cells undergo UPR-induced apoptosis (Fribley *et al*, 2009). Cancer cells frequently experience ER stress because of the harsh conditions in the tumor, and hence the UPR is often hijacked to enhance the protein folding capacity. This favors tumor cell survival and tumor growth and progression (Wang *et al*, 2009; Urra *et al*, 2016; Madden *et al*, 2019). It also involves inactivation of proapoptotic UPR pathways, often as a result of mutations and/or enhanced expression of ER chaperones, like HSPA5 (Wang *et al*, 2009). HSPA5 senses ER stress and thereby dissociates from the UPR sensors, leading to UPR activation. HSPA5 directly affects cell survival by interacting with apoptotic pathway intermediates and by blocking caspase activation (Wang *et al*, 2009; Madden *et al*, 2019). It is overexpressed in many tumor types, and its expression correlates with cancer cell survival and malignancy (Wang *et al*, 2009; Wang & Kaufman, 2014). It was shown to regulate proliferation, invasion, and metastasis in head and neck SCC (Cole *et al*, 2019) and was crucial for mutant Kras-driven lung tumorigenesis (Rangel *et al*, 2021). We identified HSPA5 as a putative interaction partner of NRF3. While this may simply reflect the assistance of HSPA5 in the folding of NRF3, our follow-up data revealed a functional role of HSPA5 in the tumor-suppressive effect of NRF3 in keratinocytes, and HSPA5 protein levels increased upon loss of NRF3. HSPA5 is normally retained in the ER by its KDEL motif, but

Figure 6. HSPA5 levels are enhanced in *NRF3*-KO cells and in invasively growing skin cancer cells.

A Percentage of cleaved caspase 3 (cl Casp3) positive cells after treatment of SCC13 EV and *NRF3*-KO cells with 1 μg/ml tunicamycin for 24 h. $N = 6$ cultures.

B Western blot analysis of total lysates from SCC13 EV and *NRF3*-KO cells treated with DMSO or 1 μg/ml tunicamycin for 24 h, using antibodies against NRF3, NRF1, HSPA5, cl PARP, CHOP, and tubulin (loading control) and quantification of the signal intensity for HSPA5, cl PARP, and CHOP. $N = 6$ (3 per cell line).

C Top: Western blot of total lysates from SCC13 EV and *NRF3*-KO cells for HSPA5 and tubulin. Bottom: Quantification of the Western blot signal intensity (left) and qRT-PCR of RNA from SCC13 EV and *NRF3*-KO cells for *HSPA5* relative to *RPL27* (right). Mean expression in EV cells was set to 1. $N = 6$ (3 per cell line).

D Representative images of sections from organotypic skin cultures with SCC13 EV and *NRF3*-KO cells stained for HSPA5 (red) combined with Hoechst staining (blue) and quantification of the HSPA5 staining intensity. $N = 3$ independent cultures, $n = 3$–5 sections. Scale bar: 50 μm.

E Representative confocal images of sections from human BCC (upper two panels) and SCC (lower two panels) stained for NRF3 (green) and HSPA5 (red). Nuclei were counterstained with Hoechst (blue). Scale bar: 50 μm.

Data information: Graphs show mean ± SD. P-values were determined using Kruskal–Wallis test (A, B) or Mann–Whitney U test (C, D). Black or gray dots indicate data points from different EV cell lines; dark or light blue dots indicate data points from different KO cell lines.
Source data are available online for this figure.

© 2023 The Authors

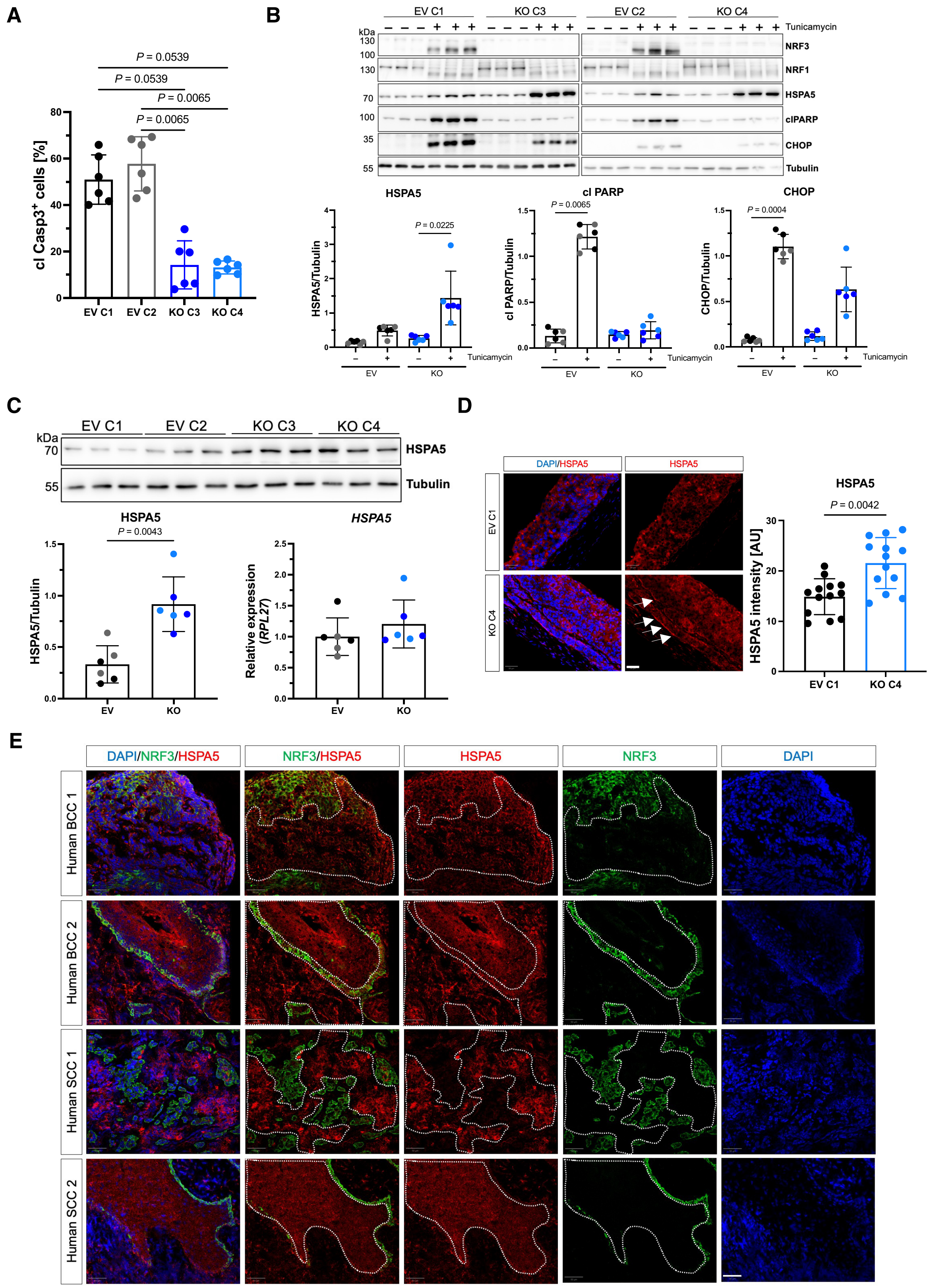

Figure 6.

 © 2023 The Authors

when its expression levels exceed a certain threshold, it can "escape" to the cell surface, in particular in stressed cells (Li & Lee, 2006). The presence of HSPA5 at the cell surface is therefore a hallmark of some cancer cells, including melanoma cells, and cell surface HSPA5 regulates various oncogenic processes, including stemness, proliferation, migration, angiogenesis, and invasiveness (Hernandez & Cohen, 2022). In NRF3-deficient SCC cells, HSPA5 levels were strongly increased, and our preliminary data also point

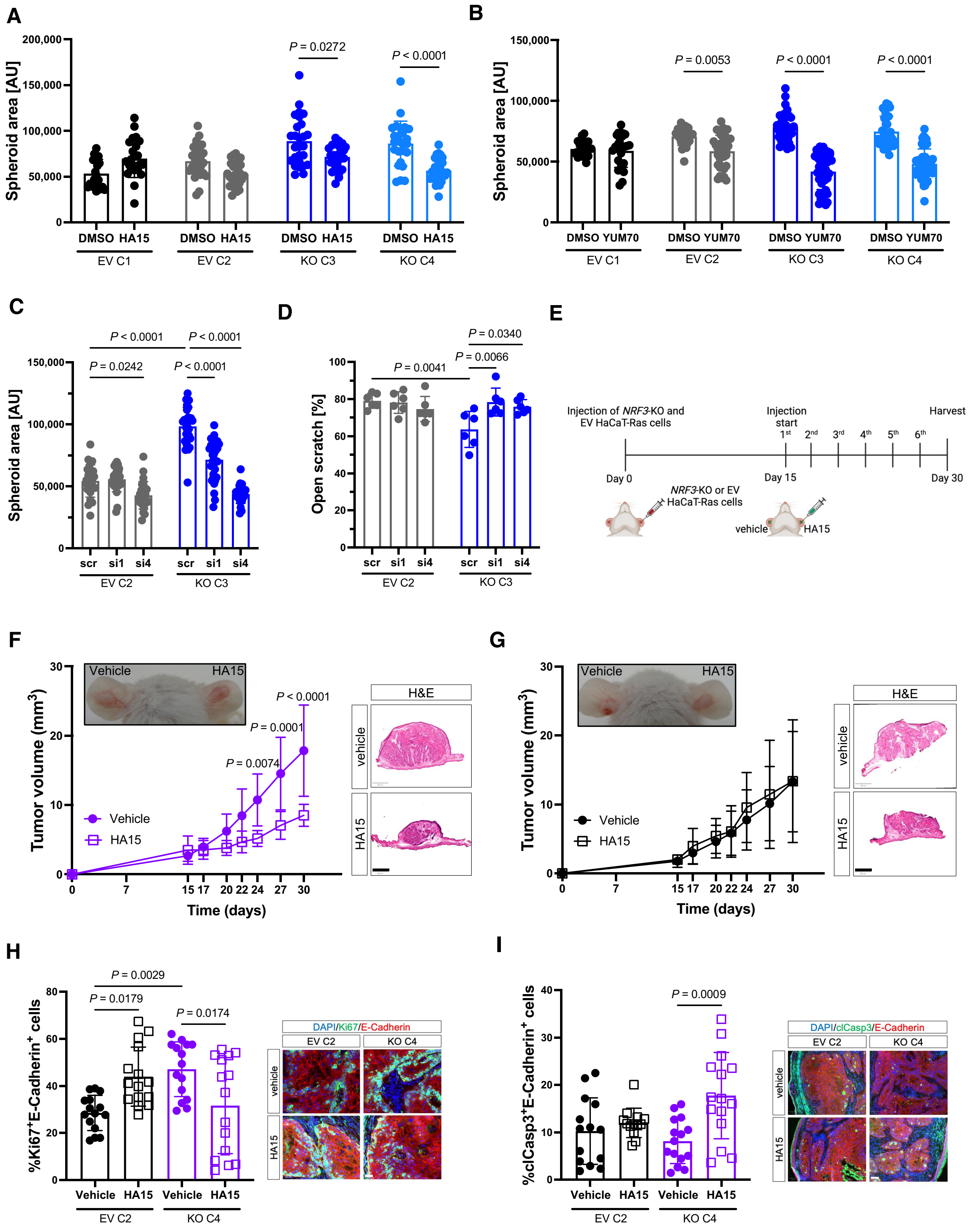

Figure 7.

Figure 7. HSPA5 is important for the tumor-suppressive effect of NRF3.

A, B Area of spheroids formed by SCC13 EV and *NRF3*-KO cells in the presence of 25 μM HA15 (A), 5 μM YUM70 (B) or vehicle (DMSO). *N* = 24–30 hanging drops.
C Area of spheroids formed by SCC13 EV and *NRF3*-KO cells transfected with scrambled (scr) or HSPA5 (si1 and si4) siRNAs. *N* = 24–31 hanging drops.
D Quantification of the area of open scratch at 9 h post scratch wounding in percentage of the original scratch area in confluent SCC13 EV and *NRF3*-KO cells transfected with scrambled or HSPA5 siRNAs. *N* = 6.
E Schematic representation of the HA15 treatment experiment. *NRF3*-KO and EV HaCaT-Ras cells were injected into the ears of NOD/SCID mice at day 0. Starting from day 15, vehicle (DMSO) or HA15 (1.69×10^{-3} mg/ear) were directly injected into the tumors every 2–3 days for a total of 6 times. Tumors were harvested at day 30. Created with BioRender.com.
F, G Left: Representative pictures of ~ 4-week-old tumors (arrows) formed in NOD/SCID mice by HaCaT-Ras *NRF3*-KO (F) or EV (G) cells after intratumoral injection of vehicle (left) or HA15 (right), and tumor volume at different time points. *N* = 6 tumors per condition pooled from two independent experiments. Right: Representative H&E stainings of sections from tumors formed by HaCaT-Ras *NRF3*-KO (F) or EV (G) cells treated with vehicle or HA15 at day 30. Scale bar: 800 μm.
H Left: Quantification of Ki67/E-cadherin-positive cells in the xenograft tumors. *N* = 3 tumors per condition, *n* = 5 sections. Right: Representative images of sections from tumors formed by HaCaT-Ras *NRF3*-KO or control cells treated with vehicle or HA15 stained for Ki67 (green), E-cadherin (red), and counterstained with Hoechst (blue). Scale bar: 50 μm.
I Left: Quantification of cleaved caspase 3 (cl Casp3)/E-cadherin-positive cells in the xenograft tumors. *N* = 3 tumors per condition, *n* = 3–5 sections. Right: Representative images of sections from tumors formed by HaCaT-Ras *NRF3*-KO or control cells treated with vehicle or HA15 stained for cl Casp3 (green), E-cadherin (red), and counterstained with Hoechst (blue). Scale bar: 50 μm.

Data information: Graphs show mean ± SD. *P*-values were determined using 2-way ANOVA (A–D and F, G) or Kruskal–Wallis test (H, I).
Source data are available online for this figure.

to a higher abundance of this protein at the plasma membrane. We do not know if the regulation of HSPA5 by NRF3 involves the help of further UPR proteins that we identified in the BioID screen. Independent of this open question, NRF3 seems to play a role in the proapoptotic arm of the UPR as suggested by the reduced apoptosis of NRF3-deficient SCC cells upon ER-stress induced by tunicamycin (this study) and of UVB-irradiated murine keratinocytes *in vitro* and *in vivo* (Siegenthaler *et al*, 2018). In the future, it will be important to determine if NRF3 and HSPA5 directly bind to each other, how the interaction of NRF3 with HSPA5 regulates the amount and localization of this ER chaperone, and if additional mechanisms contribute to the tumor-suppressive effect of NRF3 in keratinocytes.

Independent of these open questions, the findings obtained in this study are of potential therapeutic relevance because pharmacological inhibition or knock-down of HSPA5 rescued the malignant *NRF3*-KO phenotype. The HSPA5 inhibitor HA15 has previously been shown to inhibit melanoma development in mice by enhancing cancer cell death, in particular in cells with high HSPA5 levels (Cerezo *et al*, 2016). Furthermore, inhibition of HSPA5 by HA15 induced apoptosis in lung cancer cells (Wu *et al*, 2020). YUM70, another selective HSPA5 inhibitor, attenuated tumor growth in a pancreatic cancer xenograft model (Samanta *et al*, 2021). Therefore, our preclinical *in vivo* studies strongly suggest HSPA5 inhibition as a promising treatment for NMSC. This hypothesis is supported by the high expression of HSPA5 in most NMSCs (https://www.proteinatlas.org/ENSG00000044574-HSPA5/pathology/skin+cancer), in particular in areas where strong downregulation of NRF3 is observed (this study).

In summary, we identified a tumor-suppressive function of NRF3 in the skin, which involves its interaction with the UPR regulator HSPA5. This offers promising therapeutic opportunities in stratified cancer patients.

Materials and Methods

Human skin and skin cancer samples

Surplus biopsies from normal human skin, AK, BCC, and SCC were obtained from the biobank of the Department of Dermatology, University Hospital Zurich (KEK Nr. 647/800). The diagnosis of AK and tumor samples was confirmed by a board-certified dermatopathologist (RD). Only samples from patients, who had signed an informed consent, were used in this study. The experiments were conducted in conformity to the principles set out in the WMA Declaration of Helsinki and the Department of Health and Human Services Belmont Report. The use of samples for research purpose was approved by the local ethics commission (KEK Nr. 2017-00684).

Genetically modified mice

Nrf3-ko mice (in C57BL/6 background) were previously described (Derjuga *et al*, 2004). NOD-SCID mice (NOD.CB17-Prkdcscid/NCrCrl) were originally purchased from Jackson Laboratories (Bar Harbor, ME) and bred in the EPIC mouse facility at ETH Zurich. Mice were housed and fed according to federal guidelines, and all animal experiments had been approved by the local veterinary authorities of Zurich or Lausanne, Switzerland. Animals in tumor experiments were checked twice per week for the development of skin tumors. They were euthanized according to animal welfare regulations when a single tumor had a diameter of more than 1 cm. Mice were randomly assigned to the different treatment groups.

UVB irradiation of mice

Seven- to eight-week-old mice were irradiated with 100 mJ/cm^2 UVB exactly as previously described (Schäfer *et al*, 2010) and sacrificed 24 h post UV irradiation. Irradiation was monitored with a UV dosimeter.

DMBA/TPA treatment of mice

8–10 week-old female *Nrf3*-ko and wt mice received a single topical subcarcinogenic dose of 7,12-dimethylbenz(a)anthracene (DMBA; 100 μg in 300 μl acetone), followed by 25 weekly topical treatments with the tumor promoter 12-O-tetradecanoylphorbol-13-acetate (TPA; 15 μg in 200 μl acetone). Mice were sacrificed 30 or 46 weeks after DMBA treatment or when a tumor reached a size of 1 cm diameter or when more than 1 tumor with a size of more than 0.5 mm appeared, according to animal welfare regulations.

 © 2023 The Authors

Ear tumorigenicity assay

For xenograft tumor experiments, 2×10^5 cancer cells in 3 µl PBS were injected intradermally into the ear skin of 10–15 weeks-old NOD-SCID mice using a 33-gauge Hamilton micro-syringe. The control cells were injected into one ear and the cells under investigation into the other ear of the same mouse. Cells with inducible expression of a protein were pretreated for 24 h with 100 ng/ml Dox (Sigma-Aldrich; St. Louis, MO; D9891-5G) or vehicle (DMSO). Mice were then monitored for 3–4 weeks, and the size of the developing tumors was measured during the following 3–4 weeks. During the monitoring and measuring period, 20 mg/kg Dox was injected intraperitoneally (i.p.) every 3–4 days when cells with inducible overexpression were used. For the rescue experiment with the HSPA5 inhibitor (HA15), vehicle (DMSO) or HA15 (1.69×10^{-3} mg/ear) was directly injected into the ear tumors every 2–3 days, for a total of 6 times. Tumor length, width, and thickness were recorded every 2–3 days. Mitutoyo 7301 dial gauge (Mitutoyo, Urdorf, Switzerland) was used for thickness measurements; length and width were determined with a regular ruler. Before the tumors reached > 1 cm, the mice were sacrificed and the tumors harvested, bisected, and embedded in tissue freezing medium (Leica Microsystems, Wetzlar, Germany).

Cell culture

We used the SCC13 cancer cell line (Rheinwald & Beckett, 1981), human immortalized keratinocytes (HaCaT cells, Boukamp *et al*, 1988), HaCaT cells harboring the c-Ha-*ras* (EJ) oncogene (HaCaT-Ras, Ryle *et al*, 1989), and human primary foreskin fibroblasts. Human embryonic kidney cells (HEK 293T) were used for virus production. Furthermore, different *NRF3*-KO and overexpression cell lines were generated as well as cell lines with BioID fusion proteins. All cells were cultured in Dulbecco's modified Eagle's medium (DMEM; Sigma-Aldrich, MO; D6429) supplemented with 10% fetal bovine serum (FBS) and 1% penicillin/streptomycin (P/S) at 37°C and 5% CO_2. Cells were tested monthly for mycoplasma using the PCR Mycoplasma Test Kit I/C (Vitaris AG, Baar, Switzerland; PK-CA91-1096) according to the manufacturer's protocol. Authentication of the cancer cell lines was performed by the "Deutsche Sammlung von Mikroorganismen und Zellkulturen" (DSMZ) GmbH, Braunschweig, Germany.

CRISPR/Cas9-mediated knockout of *NRF3*

The lentiCRISPRv2 vector (Addgene, Watertown, MA; #52961) was used to generate *NRF3*-KO cells. Using the Benchling platform, single guide RNAs (sgRNA) against NRF3 were selected, which showed the highest efficiency and lowest off-target activity (see Table 1).

Table 1. List of sgRNAs cloned into the lentiCRISPRv2 vector.

Name	Sequence 5′ to 3′
sgRNA1	Forward: CAC CGG GTA AAG ATC TAG GTC TAC G<br>Reverse: AAA CCG TAG ACC TAG ATC TTT ACC C
sgRNA2	Forward: CAC CGA AAC TTG AGT CAT CTA GGT G<br>Reverse: AAA CCA CCT AGA TGA CTC AAG TTT C
sgRNA4	Forward: CAC CGC ACC GGT TGA CAA TCA TAT G<br>Reverse: AAA CCA TAT GAT TGT CAA CCG GTG C

Table 2. Primers used for TOPO cloning.

Name	Sequence 5′ to 3′
NRF3flag_fw	AAA CCA TAT GAT TGT CAA CCG GTG C
NRF3flag_rv	TCA CTT ATC GTC GTC ATC CTT GTA ATC CTT

The sgRNAs were cloned into the lentiCRISPRv2 vector by restriction enzyme cloning as previously described (Sanjana *et al*, 2014).

Generation of NRF3 overexpression cells

The *NRF3* cDNA was amplified and cloned into the pENTR/D-TOPO vector (Thermo Fisher Scientific, Waltham, MA; K240020) using the primers listed in Table 2. Subsequently, the LR Clonase II Enzyme mix (Thermo Fisher Scientific; 11791019) was used for cloning into the pInducer20 vector (kindly provided by Dr. S. Elledge, Harvard Medical School, Boston, MA) according to the manufacturer's protocol.

Cloning of an NRF3-BioID2 fusion protein

The Gibson Assembly® Cloning Kit (New England Biolabs [NEB]; Ipswich, MA; E5510S) was used to insert the *NRF3* cDNA into the pENTR/D-TOPO vector containing a C-terminally HA-tagged BioID ligase using primers listed in Table 3. Subsequently, the cDNA encoding the NRF3-BioID fusion protein was inserted into the pInducer20 vector using the LR Clonase II Enzyme mix (Thermo Fisher Scientific; 11791019).

Lentivirus production

HEK 293T cells were grown to 50% confluency in 10 cm petri dishes in DMEM/10% FBS. Cells were transfected using Lipofectamine 2000 (Thermo Fisher Scientific; 11668019) according to the manufacturer's protocol using the psPax2 (Addgene; 12260) and pCMV-VSV-G (Addgene; 8454) packaging plasmids and the plasmid of interest. The next day, transfection media were removed, and fresh DMEM/10% FBS/1% P/S was added. After 48 h, the conditioned media containing the produced lentiviruses were collected, centrifuged at 1,200 rpm for 5 min to remove cell debris, and filtered through a 0.45 µm filter (Sarstedt, Nürnbrecht. Germany; 83.1826.001).

Lentiviral transduction of cells

Cells were seeded into 6-well plates, incubated overnight at 37°C, 5% CO_2, and transduced the next day. For lentiviral transduction, the virus-containing media were added to the cells in a 1:3 ratio with fresh DMEM/10% FBS/1% P/S. 48 h after transduction and incubation at 37°C and 5% CO_2, the virus-containing medium was removed and replaced by the selection medium, which contained either 5 mg/ml G418 (Thermo Fisher Scientific; 11811-031) or 1 µg/ml puromycin (P8833, Sigma-Aldrich) in DMEM/10% FBS. The medium was changed every second day, and the cells were assessed under the microscope. A well with non-transduced cells and cultured with the same selection medium served as control. Once all cells in the control wells had died and the transduced cells had

© 2023 The Authors

Table 3. Primers used for Gibson Assembly.

Name	Sequence 5′ to 3′
BioID-HA_Vector.REV	AGG TGC TTC ATG GTG AAG GGG GCG G
BioID-HA_Vector.FOR	GGA AAG AGA AAG TTC GAA TTC GGA TCC TTC AAG AAC C
BioID-HA_Fragment.FOR	CCC CTT CAC CAT GAA GCA CCT GAA GCG GT
BioID-HA_Fragment.REV	ATC CGA ATT CGA ACT TTC TCT TTC CCT TTT GGG TTT CCT

recovered, the surviving colonies were slowly expanded and maintained in selection medium to prevent overgrowth by cells, which had lost expression of the transgene.

Single cell clone expansion

For clonal expansion, cells were resuspended in 1 ml of 1% BSA in PBS containing propidium iodide (PI; Sigma-Aldrich; P4170; 1:1,000 diluted). A negative control without PI was also prepared. Cells were sorted by fluorescence activated cell sorting (FACS), and 1 cell per well was added into a 96-well plate with 200 μl selection medium. The 96-well plates were incubated at 37°C and 5% CO_2, and the cells were slowly expanded. The purity of the different single cell clones was assessed by qRT-PCR and Western blot.

Cell treatment

Cells were seeded and treated on the following day. For inducible gene expression, the medium (DMEM/10% FBS/1% P/S) was supplemented with 100 ng/ml Dox and left on the cells for 24 h unless indicated differently. To induce ER-stress, cells were treated with 1 μg/ml tunicamycin (Sigma-Aldrich; T7765) for 24 h. To inhibit HSPA5, 25 μM HA15 (MedChemExpress, Princeton, NJ; HY-100437) or 5 μM YUM70 (MedChemExpress; HY-138364) were added to the cells for 24 h. As control, cells were treated with the same volume of vehicle (DMSO) for the same time period.

siRNA-mediated knock-down of HSPA5

The day before transfection, 500,000 cells per well were seeded into 6-well plates. For siRNA transfection (25 nM), Lipofectamine™ RNAi MAX (Invitrogen; 56532) diluted in Opti-MEM®-I reduced serum medium (Thermo Fisher Scientific; 31985-047) was used according to the manufacturer's instructions. The siRNAs were purchased from Dharmacon Inc., Lafayette, CA (J-008198-06-0002; J-008198-09-0002). Scrambled siRNA (Sigma-Aldrich, #SIC001) was used as control.

Preparation of protein lysates and Western blot (WB) analysis

Cells were washed with PBS, lysed in 240 mM Tris/HCl pH 6.8/ 280 mM SDS/40% (vol/vol) glycerol, and heated to 95°C for 10 min. After short centrifugation, the protein concentration was determined using the BCA Protein assay kit (Thermo Fisher Scientific; 23225) and a GloMax Microplate Reader (Promega, Madison, WI). The samples were diluted to 1 μg/μl in lysis buffer, and 10 mM dithiothreitol and bromophenol blue were added. Proteins (20 μg protein in total) were separated by SDS–PAGE and transferred to Protran nitrocellulose membranes (GE Healthcare; Chicago, IL; 10600001). Membranes were incubated for 1 h with 5% (wt/vol) skim milk powder (Rapilait; Migros, Switzerland; 150141000000) in Tris-buffered saline with Tween 20 (TBS-T) containing 25 mM Tris pH 8, 137 mM NaCl, 2.7 mM KCl, and 0.1% (vol/vol) Tween 20, to block unspecific binding sites. Cells were incubated with the primary antibody (see Table 4) diluted in 5% (wt/vol) BSA in TBS-T overnight at 4°C. The next day, the membranes were washed 3× for 10 min with TBS-T, incubated for 1 h with the secondary antibody (see Table 5) diluted in 5% (wt/vol) skim milk powder in TBS-T, and washed 3× for 10 min with TBS-T. Signals were developed using WesternBright Sirius HRP substrate (Advansta, San Jose, CA).

Table 4. List of primary antibodies used for WB.

Antigen	Dilution	Catalog #	Manufacturer
NRF3	1:1,000	/	Volker Blank
HSPA5	1:1,000	MA5-27686	Thermo Fisher Scientific
Tubulin	1:10,000	T5168	Sigma-Aldrich
GAPDH	1:10,000	5G4	HyTest, Turku, Finland
HA-tag	1:1,000	3724S	Cell Signaling Technology, Danvers, MA
Dvl2	1:1,000	3216	Cell Signaling Technology
PDIA3	1:1,000	AMAB90988	Sigma-Aldrich
CHOP	1:1,000	2895S	Cell Signaling Technology
Cleaved PARP	1:1,000	5625	Cell Signaling Technology
Lamin A/C	1:1,000	4777	Cell Signaling Technology
NRF2	1:1,000	MBS176387	MyBioSource, San Diego, CA
NRF1	1:1,000	8052	Cell Signaling Technology

Co-Immunoprecipitation (co-IP)

Lysate from one 10 cm dish was used per precipitation. Cells were washed once with PBS, scraped off with 500 μl ice-cold PBS, and transferred to an Eppendorf tube. The plate was re-rinsed with 500 μl ice-cold PBS. Cells were centrifuged for 5 min at 500 *g* and 4°C. The supernatant was removed, and the cells were lysed with 500 μl IP buffer containing 0.05% NP-40, 10% glycerol, 150 mM NaCl, 5 mM HEPES, pH 7.4, 5 mM EDTA, and 0.25% deoxycholate, supplemented with PhosSTOP™ Phosphatase Inhibitor Cocktail (Sigma-Aldrich) and cOmplete™ EDTA-free Protease Inhibitor Cocktail (Sigma-Aldrich). Samples were incubated on ice for 10 min and centrifuged for 10 min at 16,000 *g*, 4°C. The supernatant was transferred to a new tube. 50 μl were put aside as input fraction. 25 μl

Table 5. List of secondary antibodies and other reagents used for WB.

Antibody or reagent	Dilution	Catalog #	Manufacturer
Anti-mouse-IgG-HRP	1:5,000	W4021	Promega
Anti-rabbit-IgG-HRP	1:5,000	W4011	Promega
Streptavidin-HRP	1:40,000	Ab7403	Abcam

© 2023 The Authors

Table 6. Primers used for qRT-PCR.

Gene	Sequence 5′ to 3′
hRPL27	Forward: TCA CCT AAT GCC CAC AAG GTA<br>Reverse: CCA CTT GTT CTT GCC TGT CTT
hNRF3	Forward: GAG CGA GGA GAA TGG GGT AC<br>Reverse: CAA TGA GAT GCC CTC CAG TGA
hHSPA5	Forward: CAT CAC GCC GTC CTA TGT CG<br>Reverse: CGT CAA AGA CCG TGT TCT C
hGSTA4	Forward: TCT GCA AGG CTC AGC TGA TTA<br>Reverse: AAC ATG GCC CAG AAG GCT AT
hPRR7	Forward: ACC ACC GTG TTA CGA AGA GG<br>Reverse: GGA AGG GCG TGA CGA TCT TG
hNRF1	Forward: CAA CCA CTG AGG TAA ATG CCT<br>Reverse: CCT GCT CCG TTT CGC TTT CT
hNRF2	Forward: AGG TTG CCC ACA TTC CCA AA<br>Reverse: AAT GTC TGC GCC AAA AGC TG
hGCLC	Forward: GGA AGG AAG GTG TGT TTC CTG G<br>Reverse: ACT CCC TCA TCC ATC TGG CAA
hNQO1	Forward: GTG ATA TTC CAG TTC CCC CTG C<br>Reverse: AAG CAC TGC CTT CTT ACT CCG G

anti-HA magnetic beads (Thermo Fisher Scientific; 88836) per sample were washed 4× with 500 µl IP buffer without PhosSTOP and protease inhibitors and once with 500 µl complete IP buffer and resuspended in 160 µl complete IP buffer. 50 µl washed bead suspension was added to each lysate and incubated overnight at 4°C under rotation. 50 µl of the supernatant were put aside as unbound fraction. Beads were washed 3× with 500 µl TBS/0.05% Tween-20 and once with 500 µl ddH_2O, resuspended in 100 µl Western blot lysis buffer +1:10 dithiothreitol and boiled for 10 min at 95°C.

RNA isolation and qRT-PCR

Cells were washed with PBS, and RNA was extracted using the Mini Total RNA Kit (IBI Scientific, Dubuque, IA; IB47300) according to the manufacturer's protocol. 1 µg RNA was reverse transcribed with the iScript cDNA synthesis kit (Bio-Rad, Hercules, CA; 1708890). The cDNA was diluted 1:10 in water. For the qPCR reaction, 5 µl diluted cDNA were mixed with 5.5 µl LightCycler SYBR green (Roche, Rotkreuz, Switzerland; 04887352001) and 0.5 µl primer mix (10 µM; Table 6). qRT-PCR signals were detected with a Light Cycler 480 II (Roche), and the data were analyzed using the double δ cycle threshold (CT) method.

5-Bromo-2′-deoxyuridine (BrdU) assay

Cells were seeded into 12-well plates containing one coverslip per well. The next day, BrdU (10 µM final concentration) was added to the cells, which were then incubated for 6 h at 37°C and 5% CO_2. Afterward, they were washed once with 1× PBS and fixed with 4% PFA for 20 min. The DNA was denatured with 2 M HCl/0.1% Triton X-100 for 30 min. After addition of BrdU neutralization solution containing 0.1 M sodium borate pH 8.5 and incubation for 5 min, unspecific binding sites were blocked with 1% BSA in PBS for 1 h. Cells were incubated with the primary antibody (α-BrdU-FITC, Roche; 1:500 diluted) overnight at 4°C. Nuclei were then counterstained with Hoechst 33342 (Thermo Fisher Scientific; H3570).

Clonogenic assay

Totally, 10,000 SCC or HaCaT-Ras cells were seeded into each well of a 6-well plate and cultured at 37°C, 5% CO_2 for 7 days. Cells were then washed once with PBS, fixed and stained with 6% glutaraldehyde and 0.5% crystal violet in water for 30 min at room temperature (RT). After gently rinsing the wells with tap water, they were left to dry at RT. Plates were scanned with an Epson Perfection V700 Photo Scanner (Epson Deutschland GmbH, Meerbusch, Germany) and analyzed with ImageJ using the plugin "Colony Area" and the macro "Colony Measurer" (Fiji/ImageJ, Open Source).

Scratch assay

Cells were seeded into 6-well plates and cultured until they reached full confluency. To inhibit proliferation, 2 µg/ml mitomycin C (Sigma-Aldrich; M4287) was added for 2 h. Afterward, one scratch per well was made with a 200 µl pipette tip. The mitomycin C-containing medium was removed, and the wells were washed once with PBS before fresh medium was added. Pictures of the scratch were taken at different time points always at the same spot. They were analyzed manually or with the MRI wound healing tool (https://github.com/MontpellierRessourcesImagerie/imagej_macros_and_scripts/wiki/Wound-Healing-Tool).

Thiazolyl blue tetrazolium bromide (MTT) assay

Totally, 200,000 cells per well were seeded into a 12-well plate. The next day, cells were treated with DMSO (vehicle), HA15 (25 µM), or YUM70 (5 µM) for 24 h. Afterwards, 150 µl MTT (Abcam; ab146345) solution (5 mg/ml MTT in PBS) was added to each well and incubated for another 30 min. The supernatant was aspirated, and the cells were lysed with 225 µl MTT lysis buffer (49.5 µl HCl in 15 ml isopropanol) for 10 min at RT. 125 µl lysate was transferred to a 96-well plate, and the absorption was measured at 600 nm using the GloMax® Microplate reader (Promega).

Lactate dehydrogenase (LDH) cytotoxicity assay

Cells were seeded into 96-well plates. The LDH assay was performed 24 h after seeding using the Pierce™ LDH Cytotoxicity Assay Kit (Thermo Fisher Scientific) according to the manufacturer's protocol. Colorimetric measurements at 490 and 600 nm were obtained using a GloMax® Microplate reader (Promega).

Spheroid formation assay

Drops of 20 µl containing 2,000 cells were placed on the inner part of the lid of a 6 cm dish, which was then turned upside down. To prevent dehydration, 2 ml PBS was added to the bottom of the plate. Cells were incubated in the hanging drops with different cell culture media at 37°C and 5% CO_2 for 72 h. Pictures were taken, and the size of the spheroids was measured using ImageJ.

3D organotypic skin cultures

Scaffold-free organotypic 3D cultures were established as previously described (Berning *et al*, 2015). For this purpose, 1.86×10^6 human

primary foreskin fibroblasts were seeded into 6-well inserts with a 0.4 μm transparent polyethylene terephthalate (PET) membrane in 1.5 ml of sterile-filtered Medium A (DMEM with high glucose/L-glutamine and DMEM/F-12 GlutaMAX™ in a 1:1 ratio), supplemented with 0.2 mg/ml 2-phospho-L-ascorbic acid (Sigma-Aldrich; 49752), 1 ng/ml transforming growth factor β1 (Sigma-Aldrich;-T7039), 2.5 ng/ml epidermal growth factor (Sigma-Aldrich; E4127), 5 ng/ml fibroblast growth factor 2 (PeproTech, Cranbusy, NJ; 100-18B), and 5 μg/ml insulin (Sigma-Aldrich; I5500). Outside of the insert, another 12 ml of Medium A was added to Falcon® 6-well Deep Well tissue culture-treated polystyrene plates (Corning, New York, NY; 355467). Two additional rounds of fibroblast seeding as described above were performed after 2 and 4 days. Medium A inside and outside the 6-well insert was replaced every second day until day 31. On day 32, 10^6 SCC13 cells (*NRF3*-KO cells and their corresponding controls) were seeded on top of the dermal layer with 1.5 ml sterile-filtered Medium B (DMEM with high glucose/L-glutamine and DMEM-F12 GlutaMAX™ in a 1:1 ratio, supplemented with 0.2 mg/ml 2-phospho-L-ascorbic acid, 0.0084 μg/ml cholera toxin [Sigma-Aldrich; C8052], and 0.4 μg/ml hydrocortisone [Sigma-Aldrich; H0888]). Additionally, 12 ml of Medium B was added to the well. After 3 days, the insert was air-lifted by removing the media on the insert and only replacing the outside medium with 12 ml of fresh Medium B. Cultures on the inserts were then incubated at the air–liquid interface for 4 weeks. The outside medium was replaced every second day with 12 ml of Medium B until collection (day 60). Mature cultures were harvested, bisected, and embedded in tissue freezing medium (Leica Microsystems).

Immunofluorescence and immunohistochemistry stainings

Sections were deparaffinized and rehydrated or fixed with ice-cold acetone (in case of frozen sections). If needed, antigen retrieval was performed prior to the blocking procedure by incubation in hot citrate buffer (1 h at 95°C). For immunohistochemistry, endogenous peroxidase activity was quenched with 3% H_2O_2 for 10 min prior to blocking. Unspecific binding sites were blocked with 12% BSA for 1 h at 4°C, and sections were then incubated overnight at 4°C with the primary antibodies (see Table 7) diluted in the same buffer.

After three washes with PBS, slides were incubated for 1 h with the secondary antibody (see Table 8) and 1 μg/ml Hoechst 33342 (Sigma-Aldrich; 14533). Samples were washed with PBS and mounted with N-propyl gallate mounting medium. Photomicrographs were taken using a light microscope (Carl Zeiss AG, Oberkochen, Germany), a fluorescence microscope (Carl Zeiss AG), an LSM880 Airyscan confocal microscope (Carl Zeiss AG), a 3DHistech Pannoramic 250 slide scanner (3DHistech, Budapest, Hungary), or an Axioscan 7 slide scanner (Carl Zeiss AG).

Table 7. List of primary antibodies used for immunostaining.

Antigen	Dilution	Catalog #	Manufacturer
NRF3	1:200	HPA055889	Sigma-Aldrich
BrdU	1:30	11585860001	Roche
CD3	1:100	A0452	Agilent, Santa Clara, CA
Ly6G	1:100	01211A	BD Pharmingen, San Diego, CA
Ki67	1:100	ab15580	Abcam
Keratin 14	1:5,000	PRB-155P	BioLegend, San Diego, CA
Collagen IV	1:500	10710	Progen, Heidelberg, Germany
E-Cadherin	1:200	610,181	BD Pharmingen
HSPA5	1:200	MA5-27686	Thermo Fisher Scientific
HA-tag	1:100	3724S	Cell Signaling Technology
Calnexin	1:200	NB300-518	Novus Biologicals, Centennial, CO
Cleaved caspase 3	1:100	9661	Cell Signaling Technology

Hematoxylin and eosin (H&E) staining

Samples were stained with hematoxylin and eosin according to the protocol described in Table 9. Sections were mounted with Eukitt® (Merck, 03989) mounting medium.

Toluidine blue staining

Cells were stained with toluidine blue staining solution (Sigma-Aldrich; 198161) for 30 min at RT. Afterwards, they were washed twice for 5 s with ddH_2O and counterstained with hematoxylin.

Proximity ligation assay (PLA)

SCC13 or SCC13 cells harboring the HA-tagged NRF3-BioID fusion protein were seeded onto 30 mm coverslips in 12-well plates. The next day, expression of the fusion protein was induced by treatment with 1 μg/ml Dox for 24 h. Plates were washed once with PBS, fixed in 4% PFA for 20 min at RT, and permeabilized in 0.5% Triton X-100 (Carl Roth, Karlsruhe, Germany) dissolved in PBS for 5 min. PLA was performed using the Duolink™ In Situ Red kit (Sigma-Aldrich; DUO92101-1KT) according to the manufacturer's protocol. Duolink™ In Situ PLA© anti-mouse MINUS (Sigma-Aldrich; DUO92004-100RXN) and anti-rabbit PLUS probes (Sigma-Aldrich; DUO92002-100RXN) were used for ligation. The following antibodies were used for PLA: mouse anti-PDIA3 (Sigma-Aldrich, AMAB90988; 1:1,000 diluted); mouse anti-HSPA5 (Thermo Fisher Scientific, MA5-27686; 1:500 diluted); rabbit anti-HA (Cell Signaling Technology, 3724S; 1:200 diluted); and rabbit anti-NRF3 (Sigma-Aldrich, HPA055889; 1:200 diluted).

BioID labeling and sample preparation for mass spectrometry analysis

BioID interactome identification was adapted from Hesketh *et al* (2017). SCC13 and HaCaT cells with Dox-inducible expression of the NRF3-BioID-HA fusion protein were seeded into 15 cm dishes. The next day, plates were treated with 50 μM biotin (Sigma-Aldrich; B4501) and 1 μg/ml Dox or DMSO for 24 h. The following day, half of the samples were additionally treated with 10 μM MG-132 (Merck; 474790) for 6 h. Cells were scraped off with PBS, pelleted, and stored at −80°C until further usage. Cell pellets were thawed and lysed in a 4:1 volume:weight ratio in BioID lysis buffer (50 mM Tris pH 7.5, 150 mM NaCl, 0.4% SDS, 1% NP-40,

 © 2023 The Authors

Table 8. List of secondary antibodies used for staining.

Antibody	Dilution	Catalog #	Manufacturer
Anti-mouse-IgG-Cy2	1:200	115-225-003	Jackson ImmunoResearch, West Grove, PA
Anti-rabbit-IgG-Cy2	1:200	111-225-144	Jackson ImmunoResearch
Anti-rabbit-IgG-Cy3	1:200	111-165-003	Jackson ImmunoResearch
Anti-mouse-IgG-Cy3	1:200	115-166-062	Jackson ImmunoResearch

Table 9. Protocol for H&E staining.

Reagent	Time
Mayer's hematoxylin solution	1 min
ddH_2O	3 × 10 s
Scott water	30 s
ddH_2O	10 s
70% EtOH	10 s
Eosin solution	1 min
80% EtOH	2 × 10 s
90% EtOH	2 × 10 s
100% EtOH	2 × 10 s
Xylene	2 × 10 min

1.5 mM $MgCl_2$, 1 mM EDTA) supplemented with protease inhibitors (Sigma-Aldrich; P8340) and 250 U/μl benzonase (EMD-Millipore, Billerica, MA; 71205). After incubation for 30 min under rotation at 4°C, the samples were centrifuged for 20 min at 16,000 *g* and 4°C, and the supernatant was collected in a new tube. 35 μl of Dynabeads™ MyOne™ Streptavidin C1 (Thermo Fisher Scientific; 65001) slurry was added to each sample after the beads had been washed 3× with BioID lysis buffer. Samples were incubated overnight under rotation at 4°C. The next day, beads were washed once with BioID washing buffer containing 2% SDS, 50 mM Tris pH 7.5, twice with BioID lysis buffer, and three times with 50 mM ammonium bicarbonate (ABC solution). Western blot was performed to confirm the efficiency of the pull-down of the biotinylated proteins. Precipitated proteins were analyzed by mass spectrometry (see below). 100 μl trypsin solution (1 μg trypsin in 100 μl ABC solution) was added to the beads and incubated under rotation overnight at 37°C. 100 μl of bead supernatant was collected, and the beads were vortexed in 100 μl Milli Q water. The 100 μl supernatants were pooled with the previously collected supernatants, and 50 μl formic acid was added. After evaporation on a SpeedVac, the dried peptides were dissolved in 20 μl 3% acetonitrile, 0.1% formic acid buffer and vortexed extensively. After sonication for 5 min, samples were placed on a thermomixer at 600 rpm for 10 min.

Mass spectrometry analysis of biotinylated proteins

Dissolved peptides were transferred to autosampler vials for liquid chromatography-mass spectrometry (LC–MS/MS) analysis. 1 μl was injected on a NanoAcquity HPLC coupled to a Q-Exactive HF mass spectrometer (Thermo Fisher Scientific). Raw files were converted to Mascot Generic File formats (.mgf files) from which they were further analyzed using the Mascot search engine (Matrix Science, London, UK). For visualization of the protein and peptide identification results, Mascot protein identification results were imported into the Scaffold software (Proteome Software, Portland, OR). The data was uploaded to the Reprint online tool and analyzed using the CRAPome database.

RNA-sequencing

SCC13 *NRF3*-KO cells, which had been transduced with the pINDUCER20 or NRF3-pINDUCER20 vector, were incubated with 20 ng/ml Dox or DMSO (vehicle) for 10 h. Cells were washed with PBS, and RNA was extracted using the Mini Total RNA Kit (IBI Scientific; IB47300), according to the manufacturer's protocol. Using a 4200 TapeStation (Agilent Technologies, Santa Clara, CA), RNA quality was assessed, and libraries were prepared according to the Illumina TruSeq Stranded mRNA protocol. Libraries were pooled equimolarly and sequenced (single-end 100 bp) using an Illumina NovaSeq6000 instrument with a depth of approximately 20 Mio reads per sample.

For sequencing of mouse epidermal RNA, wt and *Nrf3*-ko mice were mock-treated or UVB-irradiated and sacrificed 24 h later. The epidermis was isolated exactly as described previously (Siegenthaler *et al*, 2018) and the RNA extracted with Trizol followed by purification with the RNeasy Mini Kit, including on-column DNase treatment (Qiagen, Hilden, Germany). The sequencing platform was Illumina HiSeq2500 (Illumina Inc., San Diego, CA). Preparation of the library was performed with Illumina's TruSeq Stranded mRNA kit with 700 ng total RNA as input.

RNA-Seq data quantification and statistical analysis

The raw reads were first cleaned by removing adapter sequences and poly-x sequences (> 9 nt used for detection) using fastp (Version 0.20.0; Chen *et al*, 2018). Sequence pseudo alignment of the resulting high-quality reads to the human reference genome (build GRCh38.p13), and quantification of gene-level expression (gene model definition from GENCODE release 37) was carried out using Kallisto (Version 0.46.1; Bray *et al*, 2016). To identify differentially expressed genes, we used the glm approach implemented in the software package DESeq2 (R version: 4.2.0, DESeq2 version: 1.36.0; Love *et al*, 2014). Gene expression differences with a P-value ≤ 0.01 and $\log_2$ ratio ≥ 0.5 ($N = 3$) were considered significant.

For the mouse epidermis RNA-seq, the raw reads were first cleaned by removing adapter sequences, trimming low quality ends, and filtering reads with low quality (phred quality < 20) using Trimmomatic (Bolger *et al*, 2014). Sequence alignment of the resulting high-quality reads to the mouse reference genome (build GRCm38 with the gene annotations downloaded on 2014-02-25 from Ensembl), and quantification of gene level expression was carried out using RSEM (B. Li & Dewey, 2011). Differential expression was computed using the generalized linear model implemented in the Bioconductor package DESeq2 (Love *et al*, 2014). Genes showing altered expression with adjusted (Benjamini and Hochberg method) P-values < 0.05 ($N = 3$) were considered differentially expressed.

The paper explained

Problem

Basal and squamous cell carcinomas of the skin are by far the most frequent types of cancer in humans, and their incidence is continuously increasing. There is a strong need for the development of more efficient, noninvasive treatments, which requires a thorough understanding of the underlying mechanisms and the identification of new targets for pharmacological intervention.

Results

We identified NRF3 as a potent tumor-suppressive protein in the skin. The invasively growing cancer cells of skin cancer patients loose this protein, which promotes their invasive migration and survival in the harsh tumor environment. Mechanistically, NRF3 was shown to reduce the amounts of the chaperone heat shock protein A5 (HSPA5) and its activity in the unfolded protein response. Pharmacological inhibition of HSPA5 or reduction in its levels rescued the high malignancy of NRF3-deficient cancer cells in 2D and organotypic 3D cell cultures and in preclinical mouse models.

Impact

These results suggest pharmacological HSPA5 inhibition as a promising therapeutic strategy in skin cancer patients, whose skin tumor cells have lost the expression of NRF3.

Ingenuity pathway analysis

The list of putative interaction partners found in the BioID screen with a Saint Probability of > 0.95 was used for Ingenuity pathway analysis (Qiagen). Data tables associated with the canonical pathways identified in the SCC13 or HaCaT cell line were exported to identify pathways in which the partners of NRF3 seem to be involved depending on their activation *z*-scores.

Statistical analysis

For all experiments, we used the largest possible sample size, and there was no exclusion of any animal or data point. Quantifications were performed blinded by the investigators.

Statistical analyses were performed using the Prism 9 software (GraphPad Software, San Diego, CA). For comparison of two groups, non-parametric Mann–Whitney *U* test was performed; for comparison of more than two groups, Kruskal–Wallis test or two-way ANOVA and Bonferroni's multiple comparisons test were performed. Nonsignificant (ns): $P > 0.05$; $*P < 0.05$, $**P < 0.01$, $***P < 0.001$ $****P < 0.0001$.

Data availability

The BioID mass spectrometry data have been deposited to the ProteomeXchange Consortium via the PRIDE (Perez-Riverol *et al*, 2022) partner repository with the data set identifier PXD043305; project accession: PXD043305 and are also provided in Dataset EV1 and EV2. RNA-seq files have been deposited in the Gene Expression Omnibus (GSE226792 (for mouse RNA-seq) and GSE226798 (for RescueNRF3 RNA-seq)).

Expanded View for this article is available online.

Acknowledgments

We thank Till Wüstemann, Álvaro Menchaca Muñiz, Pascal Vonlanthen, Vanessa Wälchli, Drs. Michael Meyer, Kristin Seltmann, and Marko Sesartic (all ETH Zurich) and Daniel Bachmann (University of Lausanne) for invaluable experimental help and Dr. Tobias Kockmann, Maria Domenica Moccia, and Noé Falko (Functional Genomics Center Zurich) for help with the BioID or RNA-seq experiments, respectively. This work was supported by the Wilhelm-Sander Foundation (to SW), the Swiss Cancer League (KFS-5408-08-2021 to SW), and the Swiss National Science Foundation (31003B_189364 and 310030-212212/1 to SW). SG was a member of the Life Science Zurich Graduate School. SW, DH, and RD are members of the SKINTEGRITY.CH collaborative research program.

Author contributions

Selina Gurri: Conceptualization; data curation; formal analysis; validation; investigation; visualization; methodology; writing – original draft; writing – review and editing. **Beat Siegenthaler:** Data curation; formal analysis; validation; investigation; visualization; methodology; writing – review and editing. **Michael Cangkrama:** Formal analysis; methodology; writing – review and editing. **Gaetana Restivo:** Resources; formal analysis; writing – review and editing. **Marcel Huber:** Formal analysis; methodology; writing – review and editing. **James Saliba:** Resources; writing – review and editing. **Reinhard Dummer:** Resources; formal analysis; writing – review and editing. **Volker Blank:** Resources; writing – review and editing. **Daniel Hohl:** Resources; formal analysis; supervision; writing – review and editing. **Sabine Werner:** Conceptualization; resources; supervision; funding acquisition; writing – original draft; project administration; writing – review and editing.

Disclosure and competing interests statement

Sabine Werner is a member of the Advisory Editorial Board of EMBO Molecular Medicine. This has no bearing on the editorial consideration of this article for publication.

References

Abel E, Angel J, Kiguchi K, DiGiovanni J (2009) Multi-stage chemical carcinogenesis in mouse skin: fundamentals and applications. *Nat Protoc* 4: 1350 – 1362

Aono S, Hatanaka A, Hatanaka A, Gao Y, Hippo Y, Taketo M, Waku T, Kobayashi A (2019) β-catenin/TCF4 complex-mediated induction of the NRF3 (NFE2L3) gene in cancer cells. *Int J Mol Sci* 20: 3344

auf dem Keller U, Huber M, Beyer T, Kümin A, Siemes C, Braun S, Bugnon P, Mitropoulos V, Johnson D, Johnson J *et al* (2006) Nrf transcription factors in keratinocytes are essential for skin tumor prevention but not for wound healing. *Mol Cell Biol* 26: 3773 – 3784

Berning M, Prätzel-Wunder S, Bickenbach J, Boukamp P (2015) Three-dimensional *in vitro* skin and skin cancer models based on human fibroblast-derived matrix. *Tissue Eng Part C Methods* 21: 958 – 970

Bolger A, Lohse M, Usadel B (2014) Trimmomatic: a flexible trimmer for illumina sequence data. *Bioinformatics* 30: 2114 – 2120

Boukamp P, Petrussevska R, Breitkreutz D, Hornung J, Markham A, Fusenig N (1988) Normal keratinization in a spontaneously immortalized aneuoid human keratinocyte cell line. *J Cell Biol* 106: 761 – 771

Braun S, Krampert M, Bodo E, Kümin A, Born-Berclaz C, Paus R, Werner S (2006) Keratinocyte growth factor protects epidermis and hair follicles

© 2023 The Authors

from cell death induced by UV irradiation, chemotherapeutic or cytotoxic agents. *J Cell Sci* 119: 4841–4849

Bray N, Pimentel H, Melsted P, Pachter L (2016) Near-optimal probabilistic RNA-seq quantification. *Nat Biotechnol* 34: 525–527

Bury M, Le Calvé B, Lessard F, Dal Maso T, Saliba J, Michiels C, Ferbeyre G, Blank V (2019) NFE2L3 controls colon cancer cell growth through regulation of DUX4, a CDK1 inhibitor. *Cell Rep* 29: 1469–1481.e9

Cerezo M, Lehraiki A, Millet A, Rouaud F, Plaisant M, Jaune E, Botton T, Ronco C, Abbe P, Amdouni H *et al* (2016) Compounds triggering ER stress exert anti-melanoma effects and overcome BRAF inhibitor resistance. *Cancer Cell* 29: 805–819

Cescon D, She D, Sakashita S, Zhu C, Pintilie M, Shepherd F, Tsao M (2015) NRF2 pathway activation and adjuvant chemotherapy benefit in lung squamous cell carcinoma. *Clin Cancer Res* 21: 2499–2505

Chen S, Zhou Y, Chen Y, Gu J (2018) fastp: an ultra-fast all-in-one FASTQ preprocessor. *Bioinformatics* 34: i884–i890

Chevillard G, Blank V (2011) NFE2L3 (NRF3): the Cinderella of the Cap'n'Collar transcription factors. *Cell Mol Life Sci* 68: 3337–3348

Chevillard G, Paquet M, Blank V (2011) Nfe2l3 (Nrf3) deficiency predisposes mice to T-cell lymphoblastic lymphoma. *Blood* 117: 2005–2008

Ciążyńska M, Kamińska-Winciorek G, Lange D, Lewandowski B, Reich A, Sławińska M, Pabianek M, Szczepaniak K, Hankiewicz A, Ułańska M *et al* (2021) The incidence and clinical analysis of non-melanoma skin cancer. *Sci Rep* 11: 15705

Cole D, Svider P, Shenouda K, Lee P, Yoo N, McLeod T, Mutchnick S, Yoo G, Kaufman R, Callaghan M *et al* (2019) Targeting the unfolded protein response in head and neck and oral cavity cancers. *Exp Cell Res* 382: 111386

Derjuga A, Gourley T, Holm T, Heng H, Shivdasani R, Ahmed R, Andrews N, Blank V (2004) Complexity of CNC transcription factors as revealed by gene targeting of the Nrf3 locus. *Mol Cell Biol* 24: 3286–3294

DiGiovanni J, Bhatt T, Walker S (1993) C57bl/6 mice are resistant to tumor promotion by full thickness skin wounding. *Carcinogenesis* 14: 319–321

Farrukh M, Nissar U, Afnan Q, Rafiq R, Sharma L, Amin S, Kaiser P, Sharma P, Tasduq S (2014) Oxidative stress mediated Ca^{2+} release manifests endoplasmic reticulum stress leading to unfolded protein response in UV-B irradiated human skin cells. *J Dermatol Sci* 75: 24–35

Fribley A, Zhang K, Kaufman R (2009) Regulation of apoptosis by the unfolded protein response. *Methods Mol Biol* 559: 191–204

Hernandez I, Cohen M (2022) Linking cell-surface GRP78 to cancer: from basic research to clinical value of GRP78 antibodies. *Cancer Lett* 524: 1–14

Hesketh G, Youn J, Samavarchi-Tehrani P, Raught B, Gingras A (2017) Parallel exploration of interaction space by BioID and affinity purification coupled to mass spectrometry. *Methods Mol Biol* 1550: 115–136

Hetz C (2012) The unfolded protein response: controlling cell fate decisions under ER stress and beyond. *Nat Rev Mol Cell Biol* 13: 89–102

Ibrahim L, Mesgarzadeh J, Xu I, Powers E, Wiseman R, Bollong M (2020) Defining the functional targets of Cap'n'collar transcription factors NRF1, NRF2, and NRF3. *Antioxidants* 9: 1025

Immonen A, Haapasaari K, Skarp S, Karihtala P, Teppo H (2022) NRF3 decreases during melanoma carcinogenesis and is an independent prognostic marker in melanoma. *Oxid Med Cell Longev Mar* 26: 2240223

Kandoth C, McLellan M, Vandin F, Ye K, Niu B, Lu C, Xie M, Zhang Q, McMichael J, Wyczalkowski M *et al* (2013) Mutational landscape and significance across 12 major cancer types. *Nature* 502: 333–339

Kobayashi A (2020) Roles of nrf3 in the hallmarks of cancer: proteasomal inactivation of tumor suppressors. *Cancer* 12: 2681

Kobayashi A, Ito E, Toki T, Kogame K, Takahashi S, Igarashi K, Hayashi N, Yamamoto M (1999) Molecular cloning and functional characterization of a new Cap'n'Collar family transcription factor Nrf3. *J Biol Chem* 274: 6443–6452

Li B, Dewey C (2011) RSEM: accurate transcript quantification from RNA-Seq data with or without a reference genome. *BMC Bioinformatics* 12: 323

Li J, Lee A (2006) Stress induction of GRP78/BiP and its role in cancer. *Curr Mol Med* 6: 45–54

Liu P, Kerins M, Tian W, Neupane D, Zhang D, Ooi A (2019) Differential and overlapping targets of the transcriptional regulators NRF1, NRF2, and NRF3 in human cells. *J Biol Chem* 294: 18131–18149

Liu Q, Luo Q, Feng J, Zhao Y, Ma B, Cheng H, Zhao T, Lei H, Mu C, Chen L *et al* (2022) Hypoxia-induced proteasomal degradation of DBC1 by SIAH2 in breast cancer progression. *Elife* 11: e81247

Love M, Huber W, Anders S (2014) Moderated estimation of fold change and dispersion for RNA-seq data with DESeq2. *Genome Biol* 15: 550

Madden E, Logue S, Healy S, Manie S, Samali A (2019) The role of the unfolded protein response in cancer progression: from oncogenesis to chemoresistance. *Biol Cell* 111: 1–17

Nouhi Z, Chevillard G, Derjuga A, Blank V (2007) Endoplasmic reticulum association and N-linked glycosylation of the human Nrf3 transcription factor. *FEBS Lett* 581: 5401–5406

Oshimori N, Oristian D, Fuchs E (2015) TGF-β promotes heterogeneity and drug resistance in squamous cell carcinoma. *Cell* 160: 963–976

Pepe A, Xiao Q, Zampetaki A, Zhang Z, Kobayashi A, Hu Y, Xu Q (2010) Crucial role of Nrf3 in smooth muscle cell differentiation from stem cells. *Circ Res* 106: 870–879

Perez-Riverol Y, Bai J, Bandla C, García-Seisdedos D, Hewapathirana S, Kamatchinathan S, Kundu DJ, Prakash A, Frericks-Zipper A, Eisenacher M *et al* (2022) The PRIDE database resources in 2022: a hub for mass spectrometry-based proteomics evidences. *Nucleic Acids Res* 50: D543–D552

Rangel D, Dubeau L, Park R, Chan P, Ha D, Pulido M, Mullen D, Vorobyova I, Zhou B, Borok Z *et al* (2021) Endoplasmic reticulum chaperone GRP78/BiP is critical for mutant Kras-driven lung tumorigenesis. *Oncogene* 40: 3624–3632

Rheinwald J, Beckett M (1981) Tumorigenic keratinocyte lines requiring anchorage and fibroblast support cultured from human squamous cell carcinomas. *Cancer Res* 41: 1657–1663

Rojo de la Vega M, Chapman E, Zhang D (2018) NRF2 and the hallmarks of cancer. *Cancer Cell* 34: 21–43

Rundhaug J, Fischer S (2010) Molecular mechanisms of mouse skin tumor promotion. *Cancer* 2: 436–482

Ryle C, Breitkreutz D, Stark H, Leigh I, Steinert P, Roop D, Fusenig N (1989) Density- dependent modulation of synthesis of keratins 1 and 10 in the human keratinocyte line HACAT and in ras-transfected tumorigenic clones. *Differentiation* 40: 42–54

Saliba J, Coutaud B, Makhani K, Epstein Roth N, Jackson J, Park J, Gagnon N, Costa P, Jeyakumar T, Bury M *et al* (2022) Loss of NFE2L3 protects against inflammation- induced colorectal cancer through modulation of the tumor microenvironment. *Oncogene* 41: 1563–1575

Samanta S, Yang S, Debnath B, Xue D, Kuang Y, Ramkumar K, Lee A, Ljungman M, Neamati N (2021) The hydroxyquinoline analogue YUM70 inhibits GRP78 to induce ER stress-mediated apoptosis in pancreatic cancer. *Cancer Res* 81: 1883–1895

© 2023 The Authors

Sanjana N, Shalem O, Zhang F (2014) Improved vectors and genome-wide libraries for CRISPR screening. *Nat Methods* 11: 783 – 784

Schäfer M, Dütsch S, auf dem Keller U, Navid F, Schwarz A, Johnson D, Johnson J, Werner S (2010) Nrf2 establishes a glutathione-mediated gradient of UVB cytoprotection in the epidermis. *Genes Dev* 24: 1045 – 1058

Schmidlin C, Shakya A, Dodson M, Chapman E, Zhang D (2021) The intricacies of NRF2 regulation in cancer. *Semin Cancer Biol* 76: 110 – 119

Seo S, Kim S, Lee S (2020) ER stress induced by ER calcium depletion and UVB irradiation regulates tight junction barrier integrity in human keratinocytes. *J Dermatol Sci* 98: 41 – 49

Siegenthaler B, Defila C, Muzumdar S, Beer H, Meyer M, Tanner S, Bloch W, Blank V, Schäfer M, Werner S (2018) Nrf3 promotes UV-induced keratinocyte apoptosis through suppression of cell adhesion. *Cell Death Differ* 25: 1749 – 1765

Sporn M, Liby K (2012) NRF2 and cancer: the good, the bad and the importance of context. *Nat Rev Cancer* 12: 564 – 571

Sun J, Zheng Z, Chen Q, Pan Y, Lu H, Zhang H, Yu Y, Dai Y (2019) NRF3 suppresses breast cancer cell metastasis and cell proliferation and is a favorable predictor of survival in breast cancer. *Onco Targets Ther* 12: 3019 – 3030

Sung H, Ferlay J, Siegel R, Laversanne M, Soerjomataram I, Jemal A, Bray F (2021) Global cancer statistics 2020: GLOBOCAN estimates of incidence and mortality worldwide for 36 cancers in 185 countries. *CA Cancer J Clin* 71: 209 – 249

Urra H, Dufey E, Avril T, Chevet E, Hetz C (2016) Endoplasmic reticulum stress and the hallmarks of cancer. *Trends Cancer* 2: 252 – 262

Waku T, Katayama H, Hiraoka M, Hatanaka A, Nakamura N, Tanaka Y, Tamura N, Watanabe A, Kobayashi A (2020) NFE2L1 and NFE2L3 complementarily maintain basal proteasome activity in cancer cells through CPEB3-mediated translational repression. *Mol Cell Biol* 40: e00010 – e00020

Waku T, Hagiwara T, Tamura N, Atsumi Y, Urano Y, Suzuki M, Iwami T, Sato K, Yamamoto M, Noguchi N *et al* (2021) NRF3 upregulates gene expression in SREBP2-dependent mevalonate pathway with cholesterol uptake and lipogenesis inhibition. *iScience* 24: 103180

Wang M, Kaufman R (2014) The impact of the endoplasmic reticulum protein-folding environment on cancer development. *Nat Rev Cancer* 14: 581 – 597

Wang M, Wey S, Zhang Y, Ye R, Lee A (2009) Role of the unfolded protein response regulator GRP78/BiP in development, cancer, and neurological disorders. *Antioxid Redox Signal* 11: 2307 – 2316

Werner R, Sammain A, Erdmann R, Hartmann V, Stockfleth E, Nast A (2013) The natural history of actinic keratosis: a systematic review. *Br J Dermatol* 169: 502 – 518

Wu J, Wu Y, Lian X (2020) Targeted inhibition of GRP78 by HA15 promotes apoptosis of lung cancer cells accompanied by ER stress and autophagy. *Biol Open* 9: bio053298

Xu C, Huang M, Shen G, Yuan X, Lin W, Khor T, Conney A, Kong A (2006) Inhibition of 7,12-dimethylbenz(a)anthracene-induced skin tumorigenesis in C57BL/6 mice by sulforaphane is mediated by nuclear factor E2-related factor 2. *Cancer Res* 66: 8293 – 8296

Zambrano-Román M, Padilla-Gutiérrez J, Valle Y, Muñoz-Valle J, Valdés-Alvarado E (2022) Non-melanoma skin cancer: a genetic update and future perspectives. *Cancer* 14: 2371

License: This is an open access article under the terms of the Creative Commons Attribution License, which permits use, distribution and reproduction in any medium, provided the original work is properly cited.

Expanded View Figures

Figure EV1. Loss of Nrf3 has no significant effect on the immune cell composition of chemically induced papillomas.

A Representative immunohistochemistry stainings of DMBA/TPA-induced acanthopapillomas (AP) and SCCs for $CD3^+$ T-cells (upper panel) or $Ly6G^+$ neutrophils (middle panel) and toluidine blue staining for mast cells (lower panel). Scale bar: 500 μm.

B–D Quantification of $CD3^+$ cells (B) $Ly6G^+$ cells (C) toluidine $blue^+$ (TB) (D) cells per mm^2 tumor tissue. $N_{AP\text{-}wt} = 22$, $N_{AP\text{-}ko} = 15$, $N_{SCC\text{-}wt} = 3$, $N_{SCC\text{-}ko} = 10$ tumors.

Data information: Graphs show mean ± SD.

© 2023 The Authors

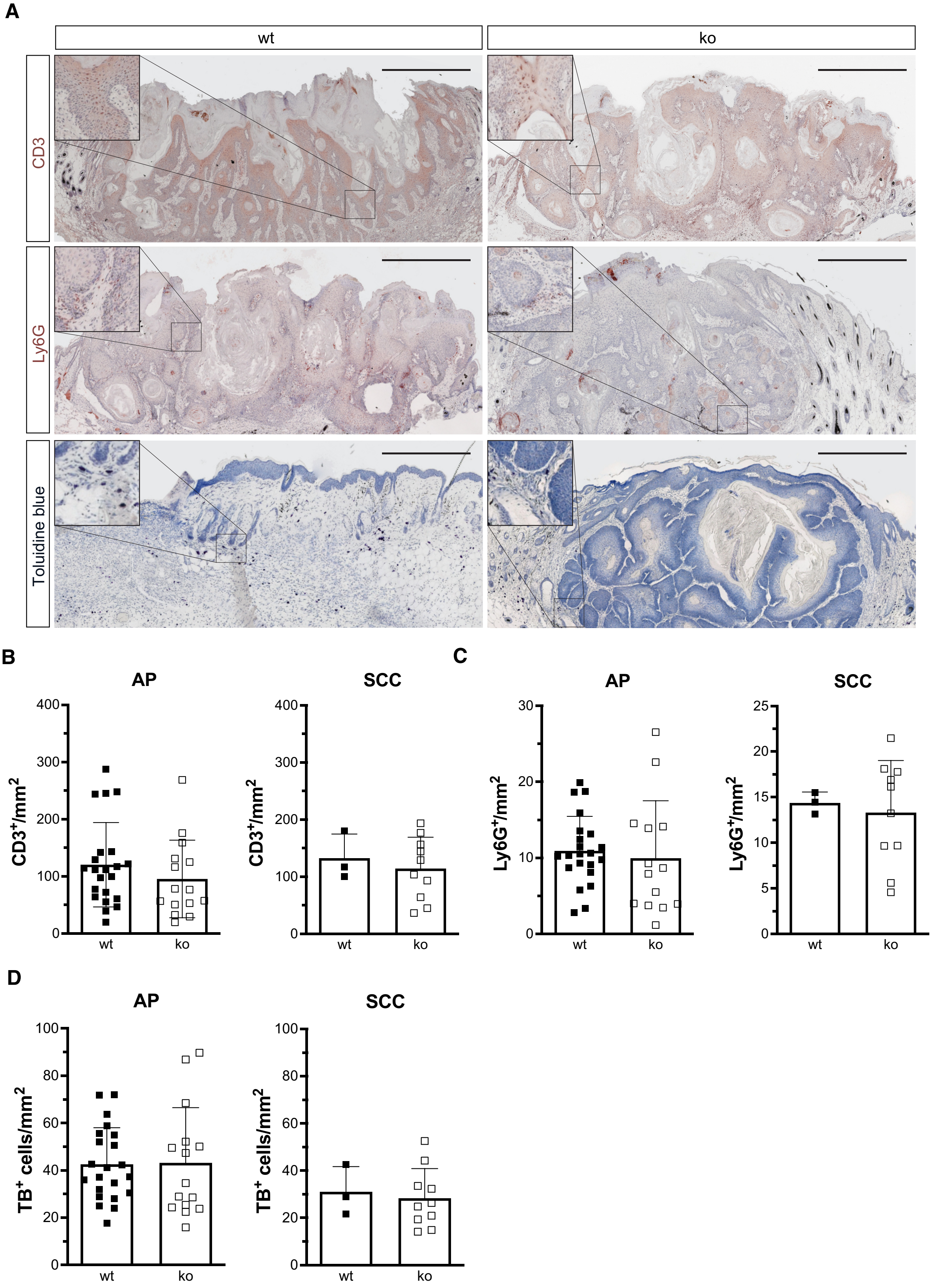

Figure EV1.

 © 2023 The Authors

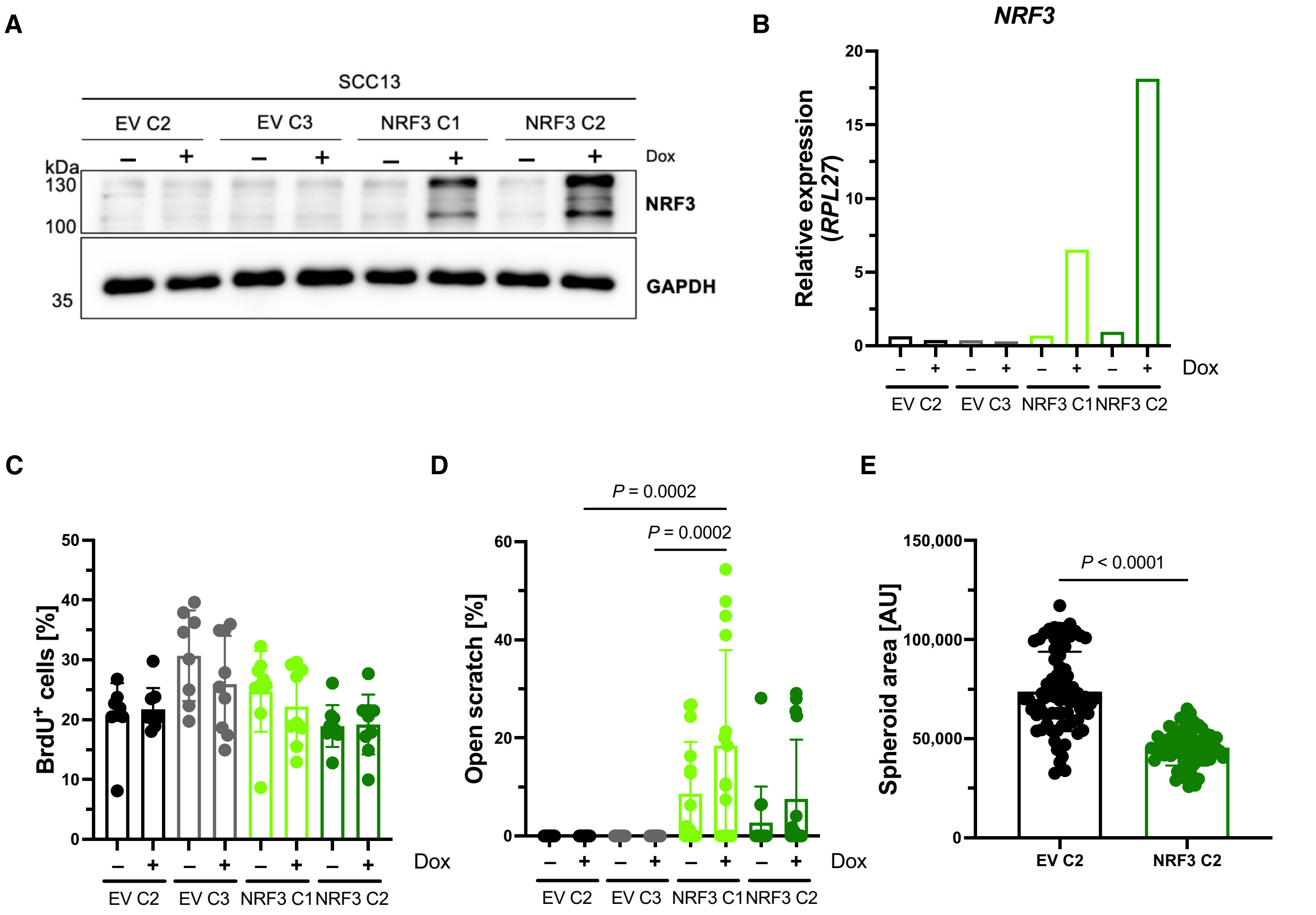

Figure EV2. Overexpression of NRF3 mildly reduces malignant features of SCC13 cells in 2D and 3D cell cultures.

A Western blot of total lysates of SCC13 cells with Dox-inducible overexpression of NRF3, treated with Dox (100 ng/ml) or vehicle, using antibodies against NRF3 and GAPDH.

B qRT-PCR for *NRF3* relative to *RPL27* using RNA from SCC13 cells with Dox-inducible overexpression of NRF3, treated with Dox (100 ng/ml) or vehicle. $N = 1$ per cell line, genotype and treatment group.

C Percentage of cells that had incorporated BrdU in cultures of SCC13 cells transduced with EV or NRF3 expression vectors and treated with Dox (100 ng/ml) or vehicle. $N = 8$–9 cultures.

D Quantification of the area of open scratch at 24 h post scratch wounding in percentage to the original scratch area in confluent SCC13 cells transduced with EV or NRF3 expression vectors and treated with Dox (100 ng/ml) or vehicle. $N = 12$.

E Area of spheroids formed by SCC13 cells transduced with EV or NRF3 expression vectors and treated with Dox (100 ng/ml) in single hanging drops. $N = 75$–80 hanging drops.

Data information: Graphs show mean ± SD. *P*-values were determined using Kruskal–Wallis test (C, D) or Mann–Whitney *U* test (E).

© 2023 The Authors

Figure EV3. Loss of NRF3 promotes malignancy of HaCaT-Ras cells in 2D and 3D cell cultures and enhances tumorigenesis and invasiveness in a xenograft mouse model.

- A Western blot of cell lysates from transduced and clonally expanded HaCaT-Ras EV and *NRF3*-KO cells, treated with MG-132 or vehicle, using antibodies against NRF3, NRF1, NRF2, and tubulin.
- B qRT-PCR using RNA from HaCaT-Ras EV and *NRF3*-KO cells for *NRF1*, *NRF2*, *GCLC*, and *NQO1* relative to *RPL27*. $N = 3$. Mean expression in EV C2 cells was set to 1.
- C Area of colonies formed by HaCaT-Ras EV and *NRF3*-KO cells relative to the whole area of the well. $N = 18–23$.
- D Percentage of HaCaT-Ras EV and *NRF*3-KO cells that had incorporated BrdU. $N = 9$.
- E Quantification of the area of open scratch at 9 h post scratch wounding in percentage of the original scratch area in confluent HaCaT-Ras EV and *NRF*3-KO cells. $N = 9$.
- F Area of spheroids formed by HaCaT-Ras EV and *NRF*3-KO cells in single hanging drops. $N = 14$ hanging drops.
- G Representative pictures of ~ 6.5-week-old tumors (indicated by arrows) formed in the ear of NOD/SCID mice upon intradermal injection of HaCaT-Ras EV and *NRF3*-KO cells and tumor volume at different time points of tumor development. $N = 6$ tumors.
- H Representative images of H&E-stained sections from tumors formed by HaCaT-Ras EV and *NRF3*-KO cells at day 45. Scale bar: 500 μm. Graph shows tumor weight at end point. $N = 6$ tumors.
- I Percentage of tumors that had invaded through the basement membrane based on H&E-stained tumor sections. $N = 6$.
- J Percentage of Ki67-positive cells among all E-cadherin-positive cells in tumors formed by HaCaT-Ras EV and *NRF3*-KO cells. $N = 3$ tumors, $n = 15$ sections.

Data information: Bar graph in (G) shows mean ± SEM, and the other graphs show mean ± SD. *P*-values were determined using Kruskal–Wallis (B–E), Mann–Whitney *U* (F, H, J), 2-way ANOVA (G), or Fisher's exact test (I).

 © 2023 The Authors

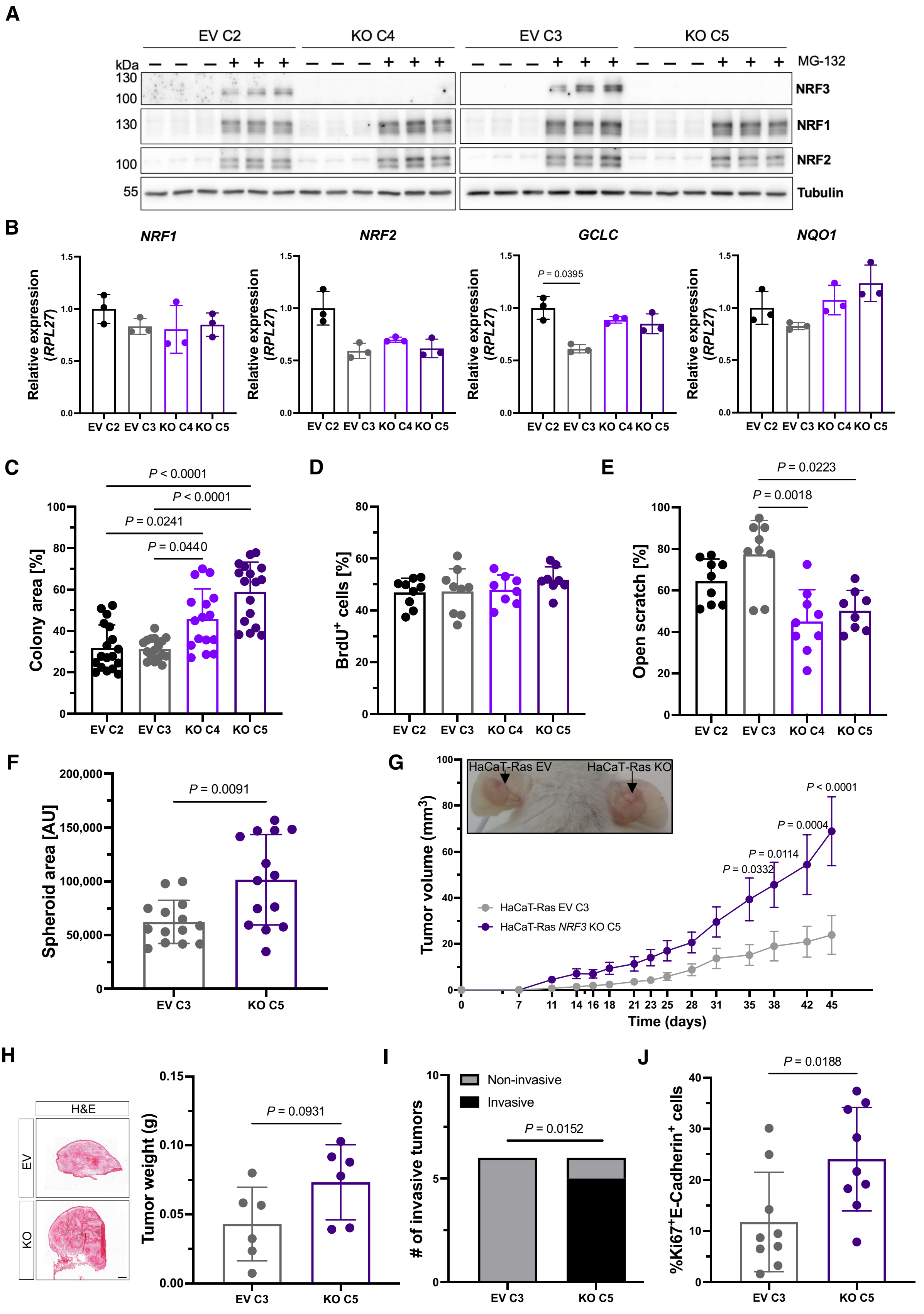
A
EV C2
KO C4
EV C3
KO C5
MG-132
kDa
NRF3
NRF1
NRF2
Tubulin
B
NRF1
NRF2
GCLC
NQO1
Relative expression (RPL27)
P = 0.0395
EV C2 EV C3 KO C4 KO C5
C
Colony area [%]
P < 0.0001
P < 0.0001
P = 0.0241
P = 0.0440
D
BrdU+ cells [%]
E
Open scratch [%]
P = 0.0223
P = 0.0018
F
Spheroid area [AU]
P = 0.0091
EV C3
KO C5
G
HaCaT-Ras EV
HaCaT-Ras KO
Tumor volume (mm3)
HaCaT-Ras EV C3
HaCaT-Ras NRF3 KO C5
P < 0.0001
P = 0.0004
P = 0.0114
P = 0.0332
Time (days)
H
H&E
EV
KO
Tumor weight (g)
P = 0.0931
I
Non-invasive
Invasive
P = 0.0152
# of invasive tumors
J
%Ki67+E-Cadherin+ cells
P = 0.0188

Figure EV3.

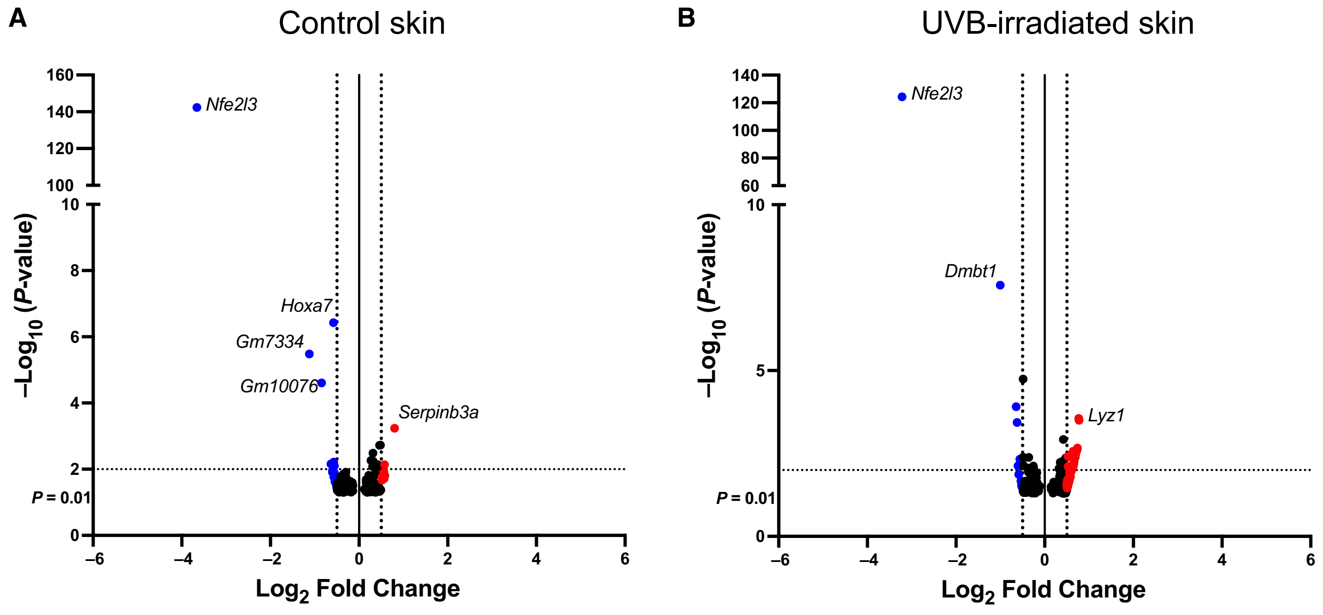

Figure EV4. Very few genes are differentially expressed in normal and UVB-irradiated epidermis of Nrf3-ko mice vs. wt littermates.

A, B Volcano plot of RNA-Seq data of isolated epidermis from untreated *Nrf3*-ko and wt mice (A) and at 24 h after irradiation with 100 mJ/cm^2 UVB (B). Significantly up- or downregulated transcripts are shown in red, respectively blue. A *P*-value < 0.05 (dotted black line at $-\log_{10}$(*P*-value) = 2) and a fold change > 0.5 (dotted black line at $\log_2$(FC) = ±0.5) were used as a cutoff.

Figure EV5. HSPA5 is important for the tumor-suppressive effect of NRF3 in HaCaT-Ras cells.

A Top: Western blot using total cell lysates of SCC13 RescueNRF3 cells treated with DMSO, 20 ng/ml Dox or 1,000 ng/ml Dox for 24 h. The membrane was probed with antibodies against NRF3, HSPA5, and tubulin. Bottom: qRT-PCR using RNA from SCC13 RescueNRF3 cells treated with DMSO, 20 ng/ml Dox or 1,000 ng/ml Dox for 10 h for *NRF3* and *HSPA5* relative to *RPL27*. Mean expression in DMSO-treated cells was set to 1. *N* = 3 cultures per treatment group.

B Top: Western blot using total cell lysates of HaCaT-Ras EV and *NRF3*-KO cells treated with 10 µM MG-132 or vehicle (DMSO) for 6 h. The membrane was probed with antibodies against NRF3 and tubulin. Bottom: Quantification of the HSPA5/tubulin ratio (left) and qRT-PCR using RNA from HaCaT-Ras EV and *NRF3*-KO cells for *HSPA5* (right). Mean expression in EV cells was set to 1. Black or gray dots indicate data points from different EV cell lines; dark or light purple dots indicate data points from different KO cell lines. *N* = 6 cultures per genotype and treatment group (3 per cell line).

C, D Area of spheroids formed by HaCaT-Ras EV and *NRF3*-KO cells in single hanging drops cultured in the presence of 25 µM HA15 (C) or 5 µM YUM70 (D) or DMSO (vehicle). *N* = 23–28 hanging drops.

E Area of spheroids formed by HaCaT-Ras EV and *NRF3*-KO cells, which had been transfected with scrambled (scr) or HSPA5 siRNAs. *N* = 24–28 hanging drops.

F Quantification of the area of open scratch at 6 h in percentage of the original scratch area in confluent HaCaT-Ras control and *NRF3*-KO cells after transfection with scrambled (scr) or HSPA5 siRNAs. *N* = 6.

G Left: Bar graph showing volume of tumors formed by HaCaT-Ras EV and *NRF3*-KO cells in NOD/SCID mice at different time points after intratumoral injection of vehicle or HA15. Right: Three-way ANOVA output table. The relevant parameter is shown in bold. *N* = 6 tumors per genotype and treatment group, pooled from two independent experiments.

Data information: Bar graphs indicate mean ± SD. *P*-values were determined using Kruskal–Wallis test (A, B), 2-way ANOVA test (C–F), or 3-way ANOVA test (G).

 © 2023 The Authors

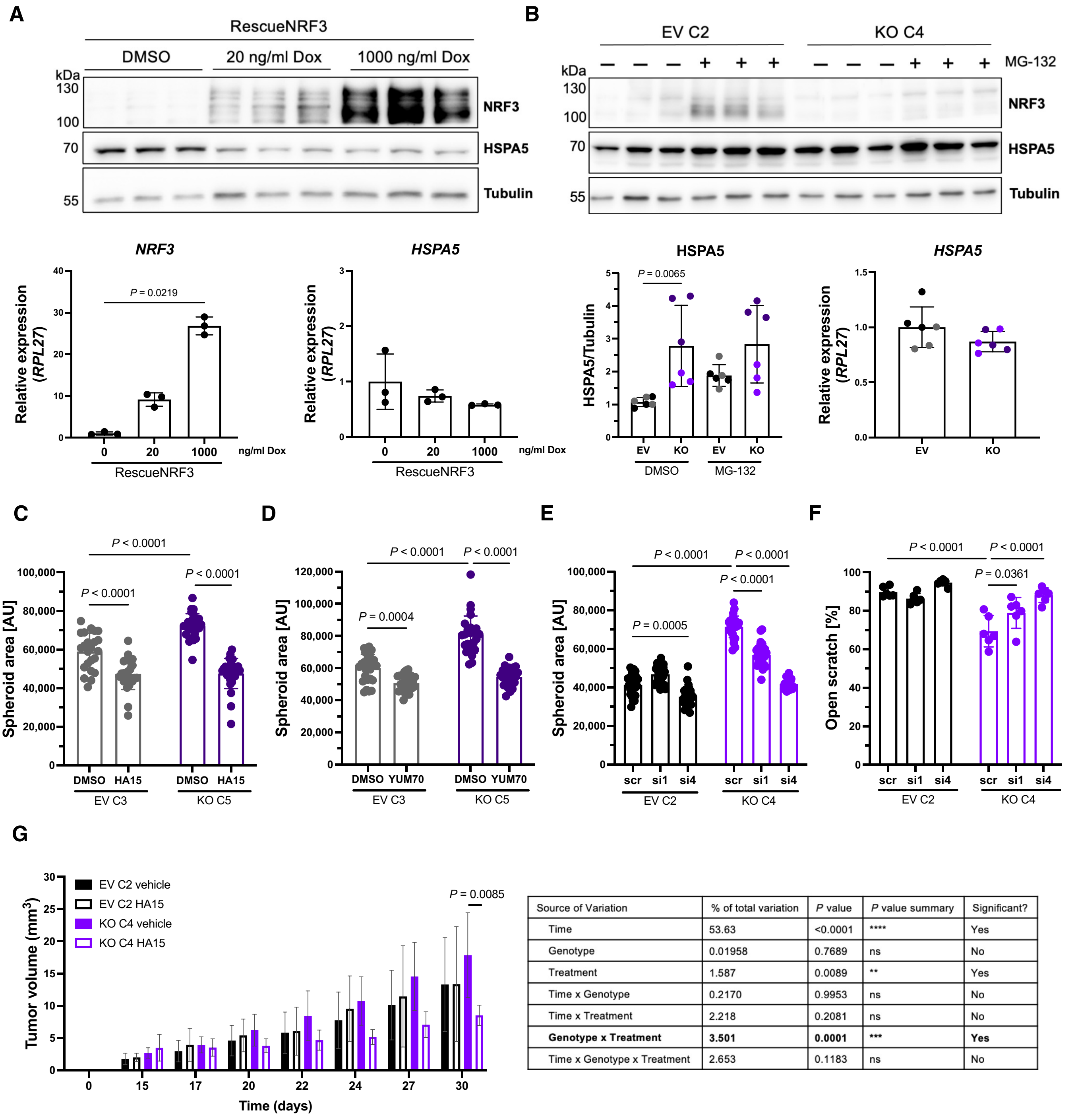

Source of Variation	% of total variation	P value	P value summary	Significant?
Time	53.63	<0.0001	****	Yes
Genotype	0.01958	0.7689	ns	No
Treatment	1.587	0.0089	**	Yes
Time x Genotype	0.2170	0.9953	ns	No
Time x Treatment	2.218	0.2081	ns	No
**Genotype x Treatment**	**3.501**	**0.0001**	***	**Yes**
Time x Genotype x Treatment	2.653	0.1183	ns	No

Figure EV5.

Table of Content

Appendix Figure S1	Page 2
Appendix Figure S2	Page 3
Appendix Figure S3	Page 4
Appendix Figure S4	Page 5
Appendix Figure S5	Page 6
Appendix Figure S6	Page 7

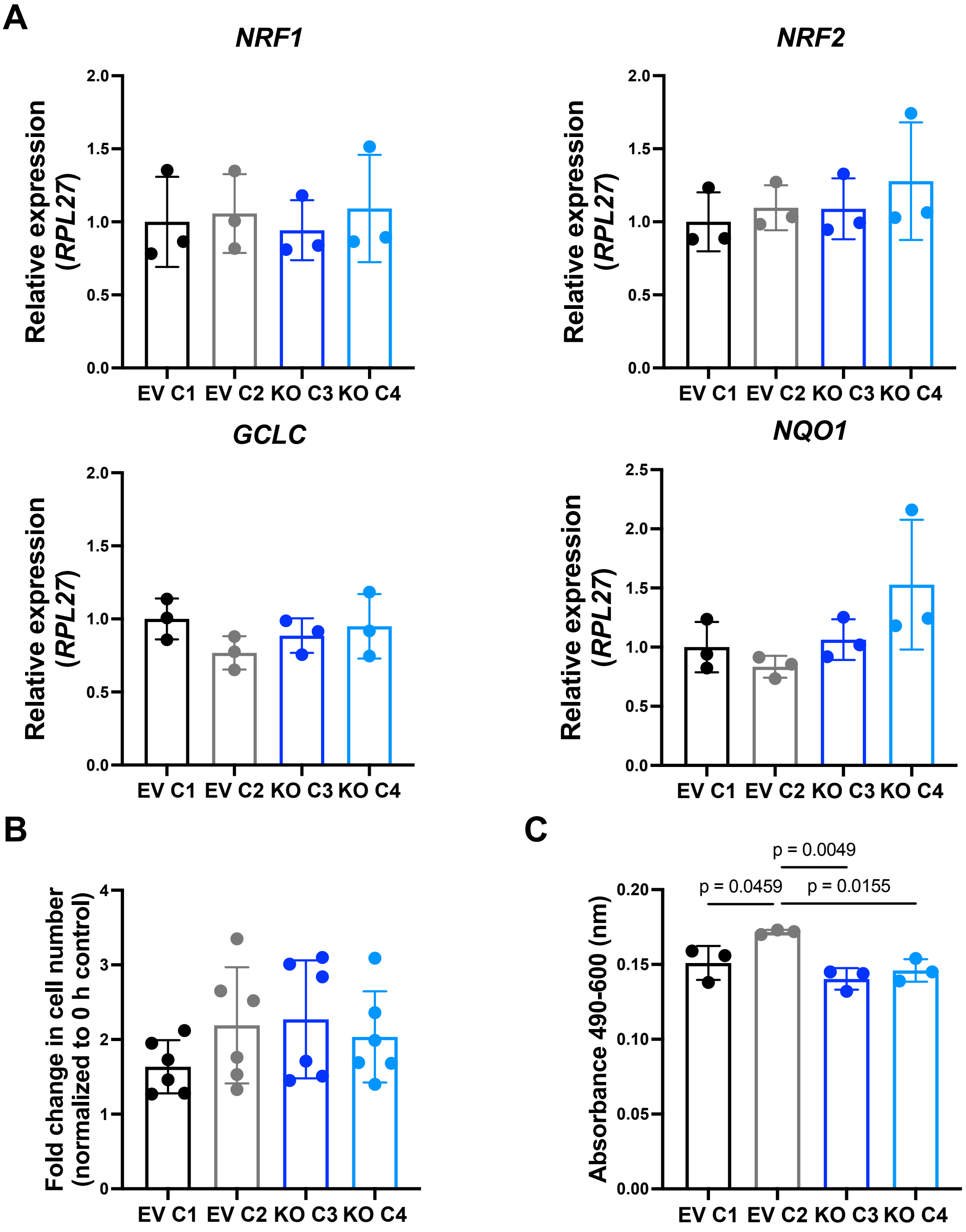

Appendix Figure S1 - Loss of NRF3 neither affects the viability of SCC13 cells nor the expression of *NRF1* and *NRF2* and of major NRF2 target genes under normal culture conditions

SCC13 EV and *NRF3*-KO cells were cultivated under normal culture conditions for 24 h.

A qRT-PCR using RNA from SCC13 EV and *NRF3*-KO cells for *NRF1, NRF2, GCLC* and *NQO1* relative to *RPL27*. N=3. Mean expression in EV C1 cells was set to 1.

B Fold change in cell number based on MTT assay (N = 6)

C Cytotoxicity based on LDH Assay (N = 3).

Data information: Bar graphs show mean +/- S.D. P-values were determined using Kruskal-Wallis test.

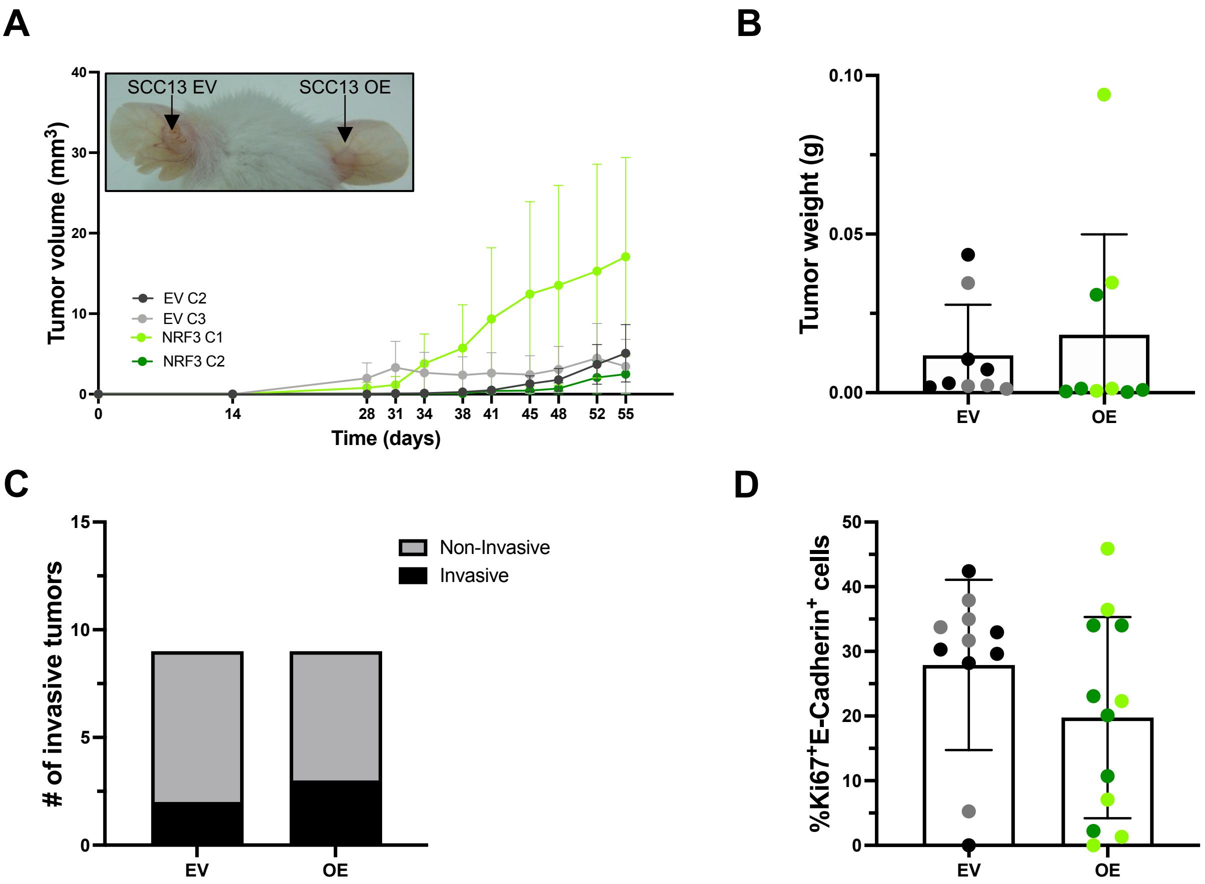

Appendix Figure S2 - Overexpression of NRF3 does not affect tumorigenesis and invasiveness of SCC13 cells in a xenograft mouse model

SCC13 cells transduced with EV or an NRF3 expression vector were intradermally injected into the ear of NOD/SCID mice to induce tumor formation.

A Representative pictures of ~8-week-old tumors (indicated by arrows) and tumor volume at different time points of tumor development. N = 4-5 tumors per cell line. Graph shows mean +/- S.E.M.

B Tumor weight at endpoint. N = 9 tumors.

C Percentage of tumors that had invaded through the basement membrane based on H&E-stained tumor sections. N = 9.

D Percentage of Ki67/E-cadherin-positive cells among all E-cadherin positive cells in the tumors. N = 4 tumors, n = 9-15 sections.

Data information: Bar graphs show absolute values (C), mean +/- S.E.M. (A) or mean +/- S.D. (B,D). P-values were determined using Mann-Whitney U test (B, D) or Fisher's exact test (C). Black or grey dots indicate data points from different EV cell lines; dark or light green dots indicate data points from different OE cell lines.

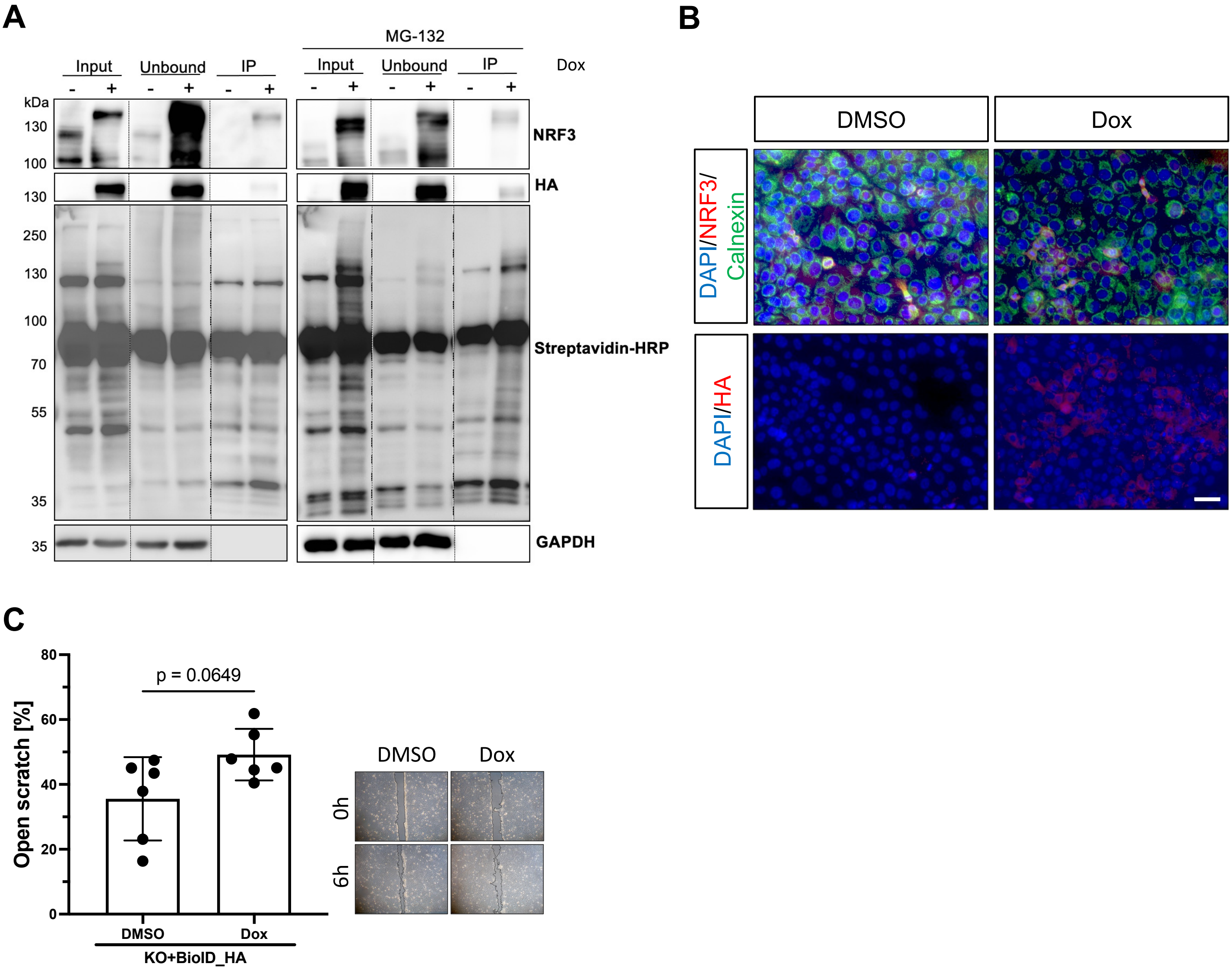

Appendix Figure S3 - Validation of NRF3-BioID fusions proteins in SCC13 cells

A Western blot for NRF3, HA fusion proteins and GAPDH and detection of biotinylated proteins using horseradish peroxidase (HRP)-coupled streptavidin in lysates from SCC13 cells with inducible expression of NRF3-BioID fusion proteins in the presence of Dox and the proteasome inhibitor MG-132 or vehicle. The dashed lines indicate where the membrane was cropped.

B Representative immunofluorescence stainings of SCC13 cells with Dox-inducible expression of HA-tagged NRF3-BioID fusion proteins cultured in the presence or absence of Dox. Cells were stained with antibodies against the ER marker calnexin (green), NRF3 (red) or the HA epitope (red) and counterstained with Hoechst (blue). Scale bar: 50 µm.

C Confluent SCC13 cells with Dox-inducible expression of HA-tagged NRF3-BioID fusion proteins were subjected to scratch wounding in the presence or absence of Dox (1 µg/ml). N = 6. The area of open scratch at 6 h is shown as percentage of the initial open scratch.

Data information: Bar graphs show mean +/- S.D.. P-values were determined using Mann-Whitney U test.

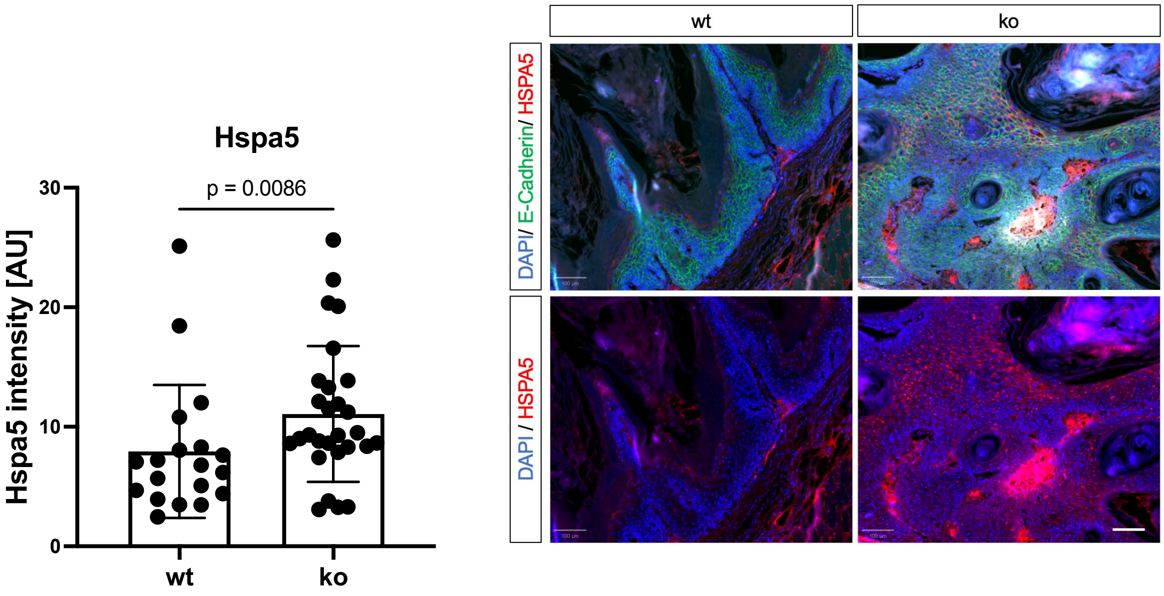

Appendix Figure S4 - HSPA5 levels are enhanced in the DMBA/TPA-induced tumors in *Nrf3*-ko mice

A Quantification of the HSPA5 staining intensity and representative images of sections from DMBA/TPA-induced tumors in wt and *Nrf3*-ko mice stained for HSPA5 (red), E-Cadherin (green) combined with Hoechst staining (blue). N = 8-9 tumors, n = 1-4 histological sections. Scale bar: 100 µm.

Data information: Graphs show mean +/- S.D.. P-value was determined using Mann-Whitney U test.

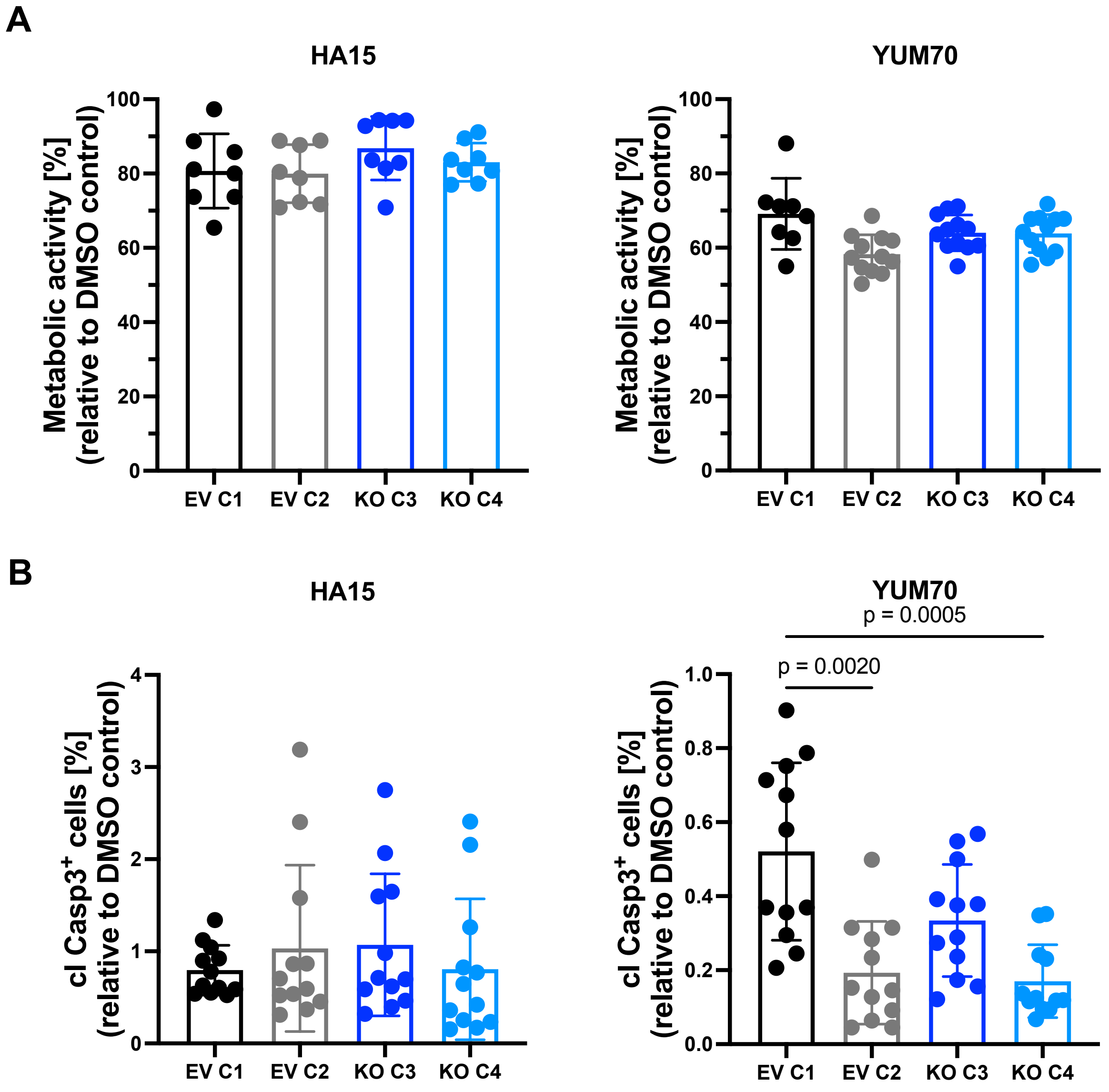

Appendix Figure S5 - HSPA5 inhibitors do not affect survival of SCC13 EV and *NRF3*-KO cells under non-challenged conditions

SCC13 EV and *NRF3*-KO cells were treated with DMSO, 25 µM HA15 (left) or 5 µM YUM70 (right) for 24 h.

A Metabolic activity of inhibitor-treated cells based on MTT assay relative to DMSO-treated control cells (N = 8).

B Percentage of cleaved caspase 3 positive inhibitor-treated cells relative to DMSO-treated control cells (N = 12).

Data information: Bar graphs show mean +/- S.D.. P-values were determined using Kruskal-Wallis test.

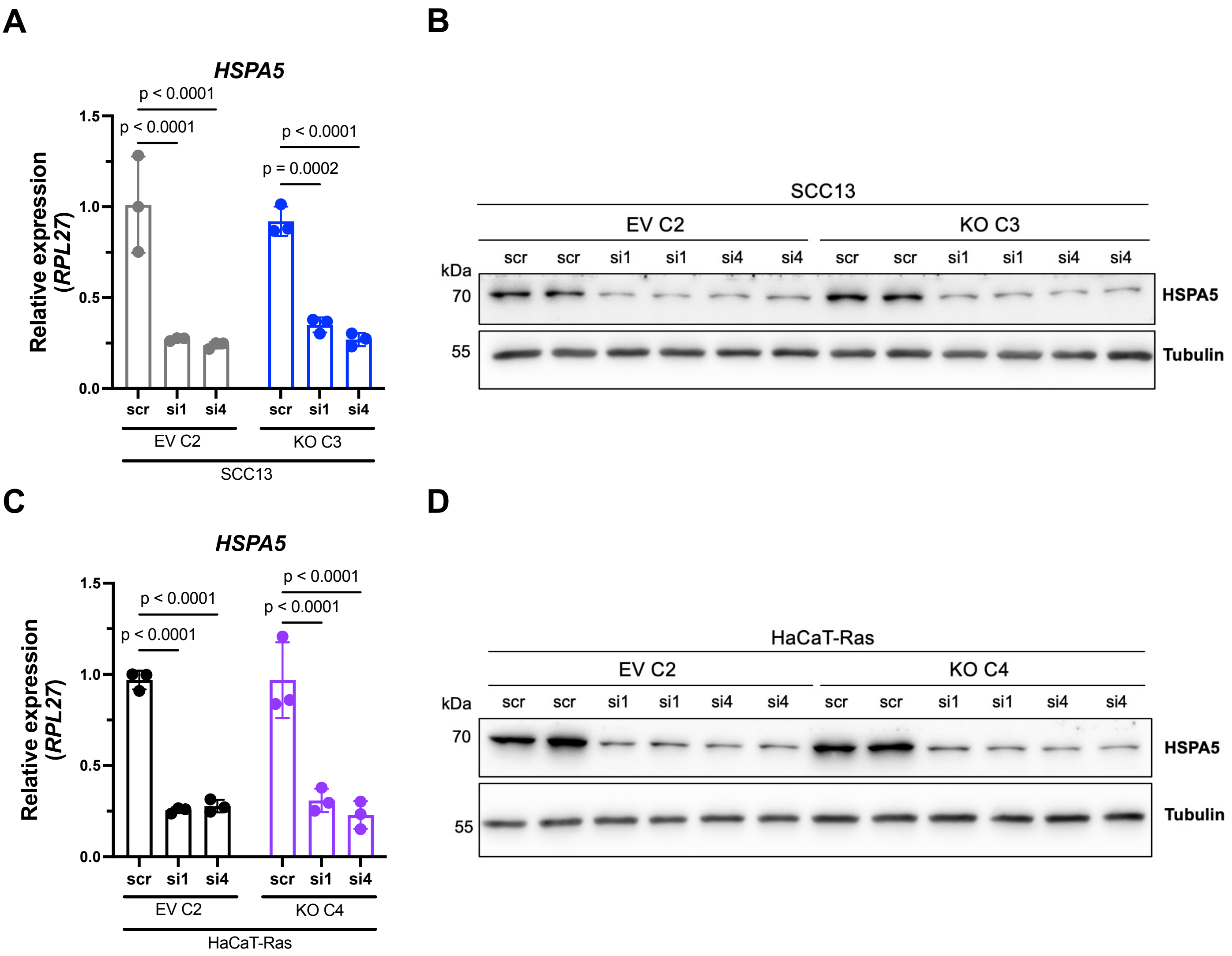

Appendix Figure S6 - Validation of siRNA-mediated HSPA5 knock-down

A-D SCC13 EV or *NRF*3-KO cells (A, B) or HaCaT-Ras EV or *NRF3*-KO cells (C, D) were transfected with scrambled (scr) or HSPA5 siRNAs and analyzed 24 h (RNA) or 48 h (protein) after transfection.

(A, C) qRT-PCR using RNA from the knock-down cells for *HSPA5* relative to *RPL27*.

(B, D) Western blot of total cell lysates of the knock-down cells using antibodies against HSPA5 or tubulin (loading control).

Data information: Bar graphs show mean +/- S.D.. P-values were determined using 2-way ANOVA.
