# Supplementary figures and images for: NRF3 suppresses squamous carcinogenesis, involving the unfolded protein response regulator HSPA5 (part 1 of 2)
Source: EMBO Mol Med. 2023 Oct 9;15(11):e17761. doi: 10.15252/emmm.202317761 (PMC10630885; doi:10.15252/emmm.202317761)

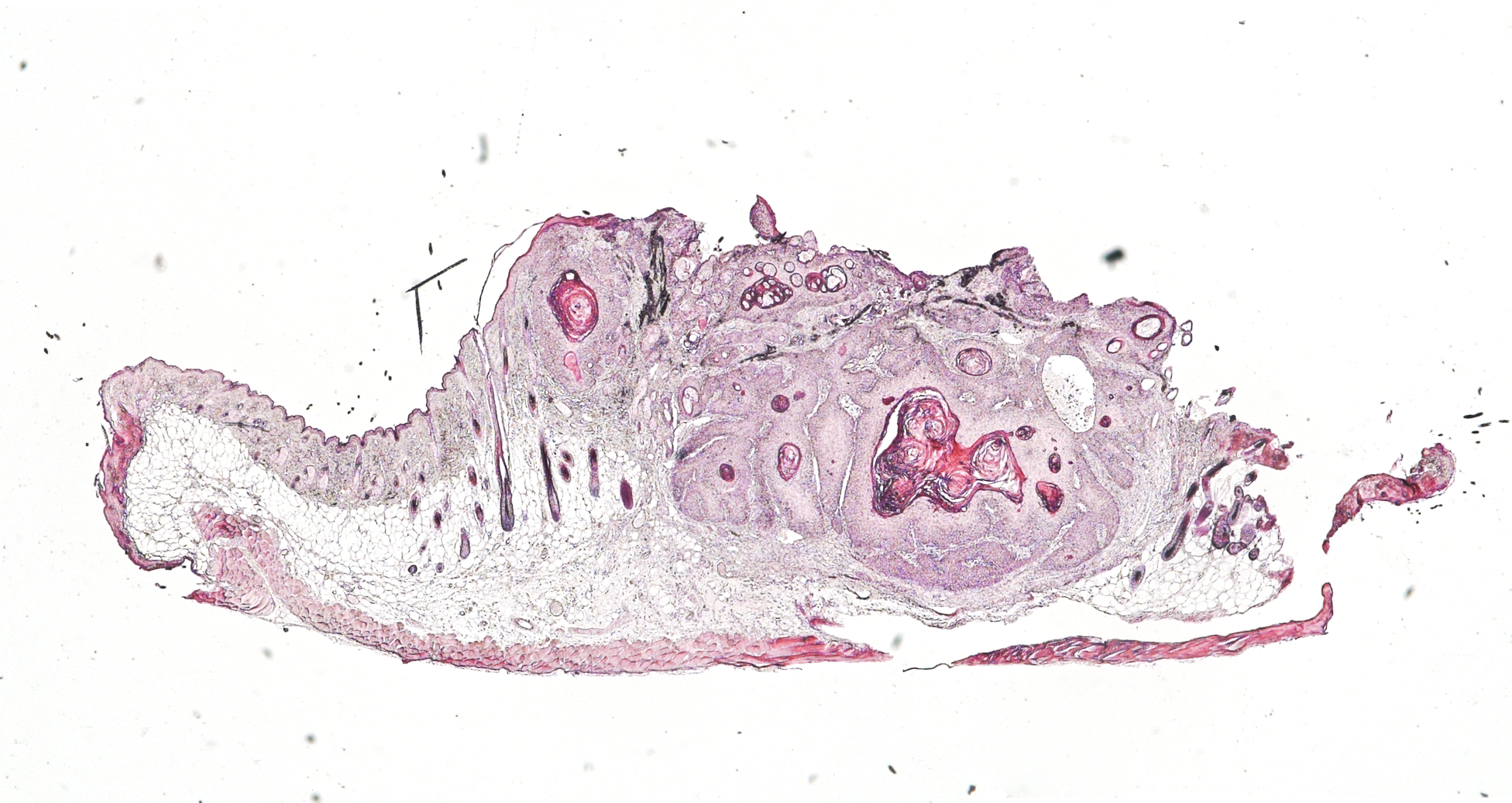

Supplement: Supplementary file 7 — Source Data for Figure 2 [file EMMM-15-e17761-s006.zip › Figure 2/2A/micr.image_SCC_ko.tif]

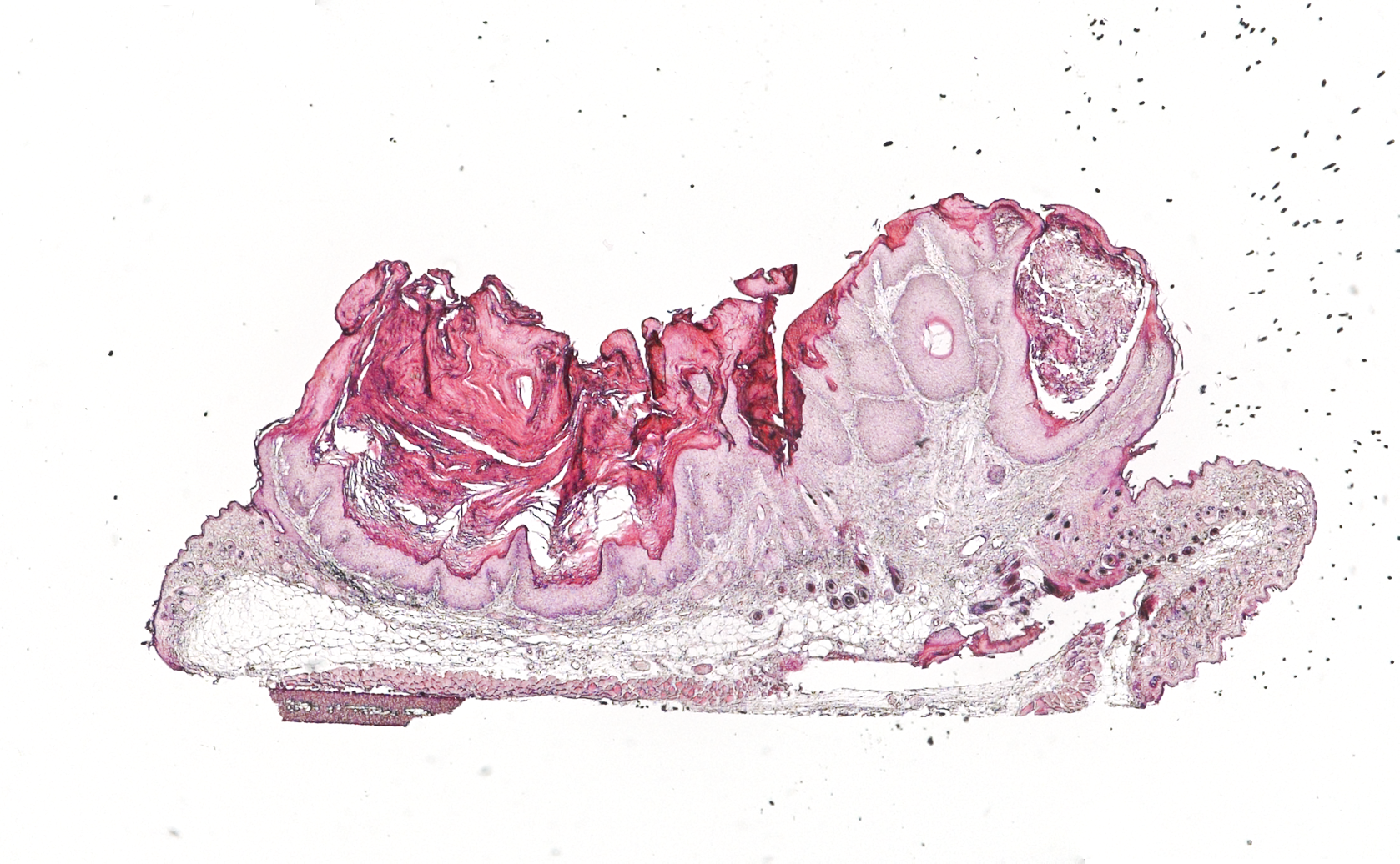

Supplement: Supplementary file 7 — Source Data for Figure 2 [file EMMM-15-e17761-s006.zip › Figure 2/2A/micr.image_AP_ko.tif]

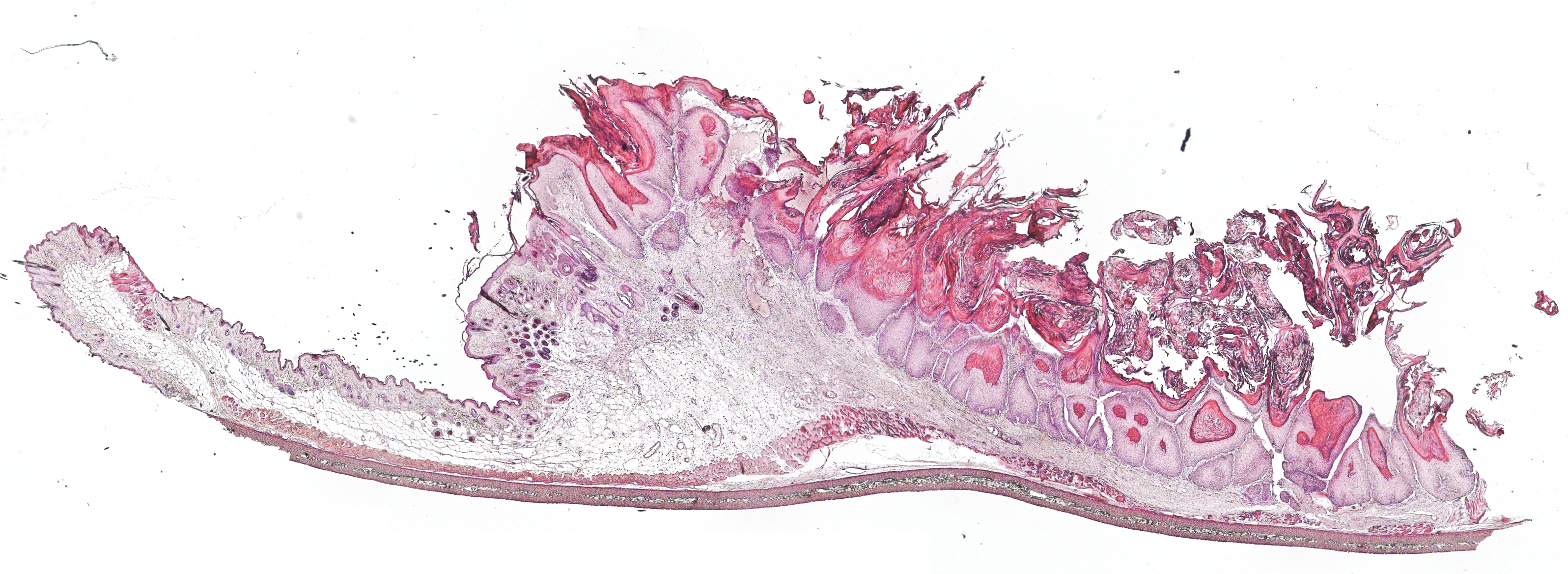

Supplement: Supplementary file 7 — Source Data for Figure 2 [file EMMM-15-e17761-s006.zip › Figure 2/2A/micr.image_SCC_wt.tif]

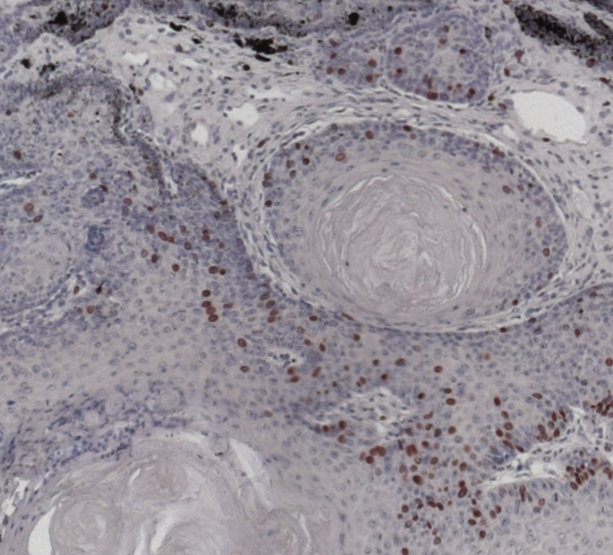

Supplement: Supplementary file 7 — Source Data for Figure 2 [file EMMM-15-e17761-s006.zip › Figure 2/2A/micr.image_BrdU_ko_higher magnification.tif]

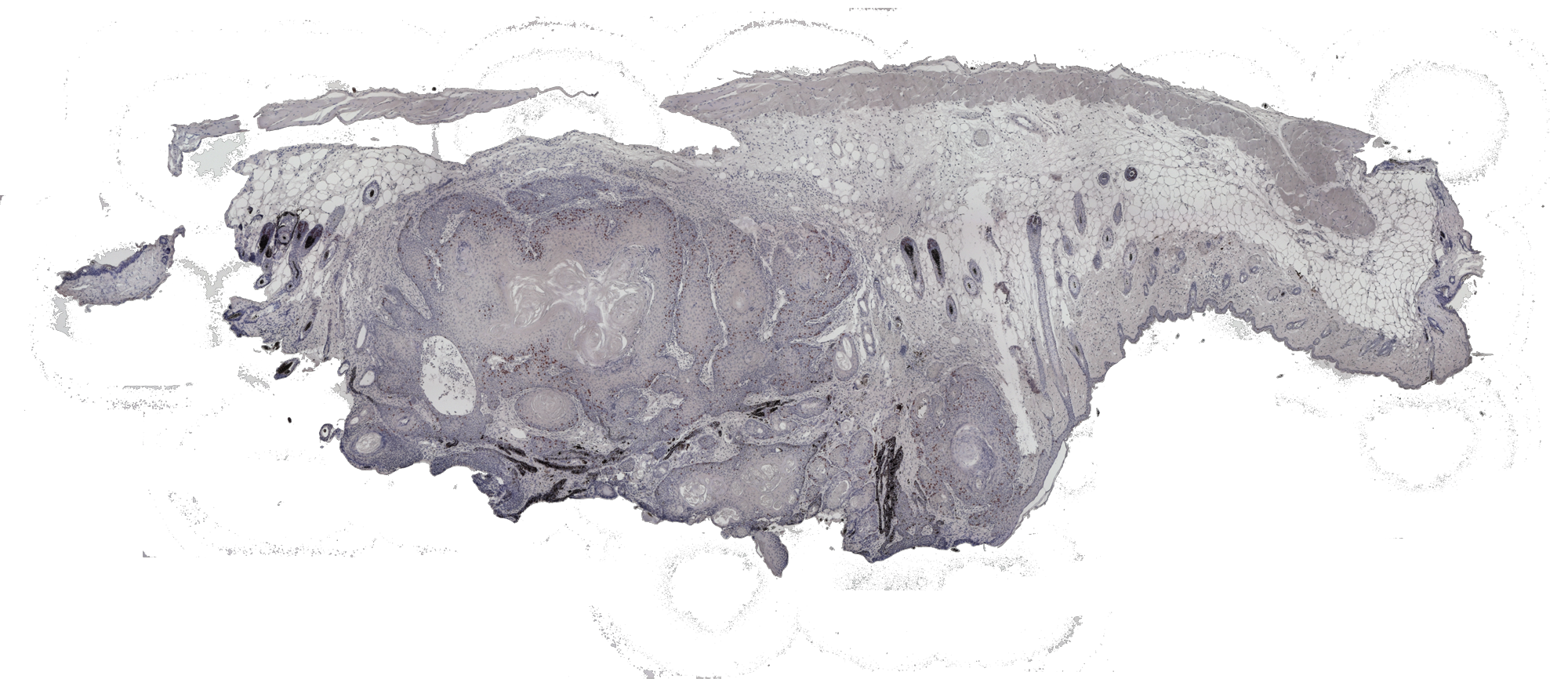

Supplement: Supplementary file 7 — Source Data for Figure 2 [file EMMM-15-e17761-s006.zip › Figure 2/2A/micr.image_BrdU_ko.tif]

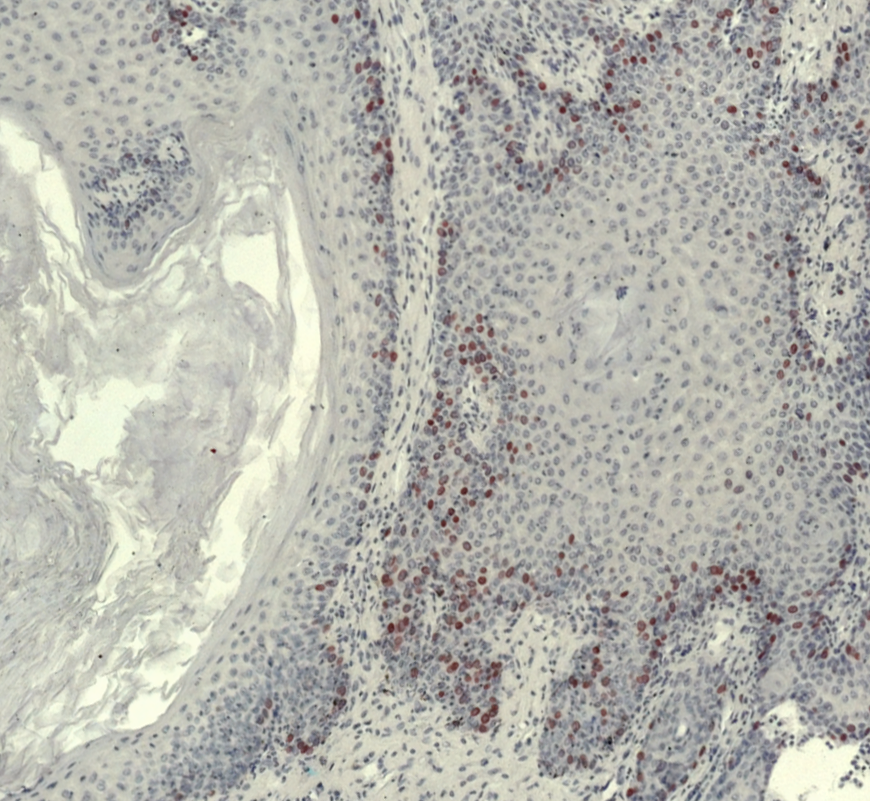

Supplement: Supplementary file 7 — Source Data for Figure 2 [file EMMM-15-e17761-s006.zip › Figure 2/2A/micr.image_BrdU_wt_higher magnification.tif]

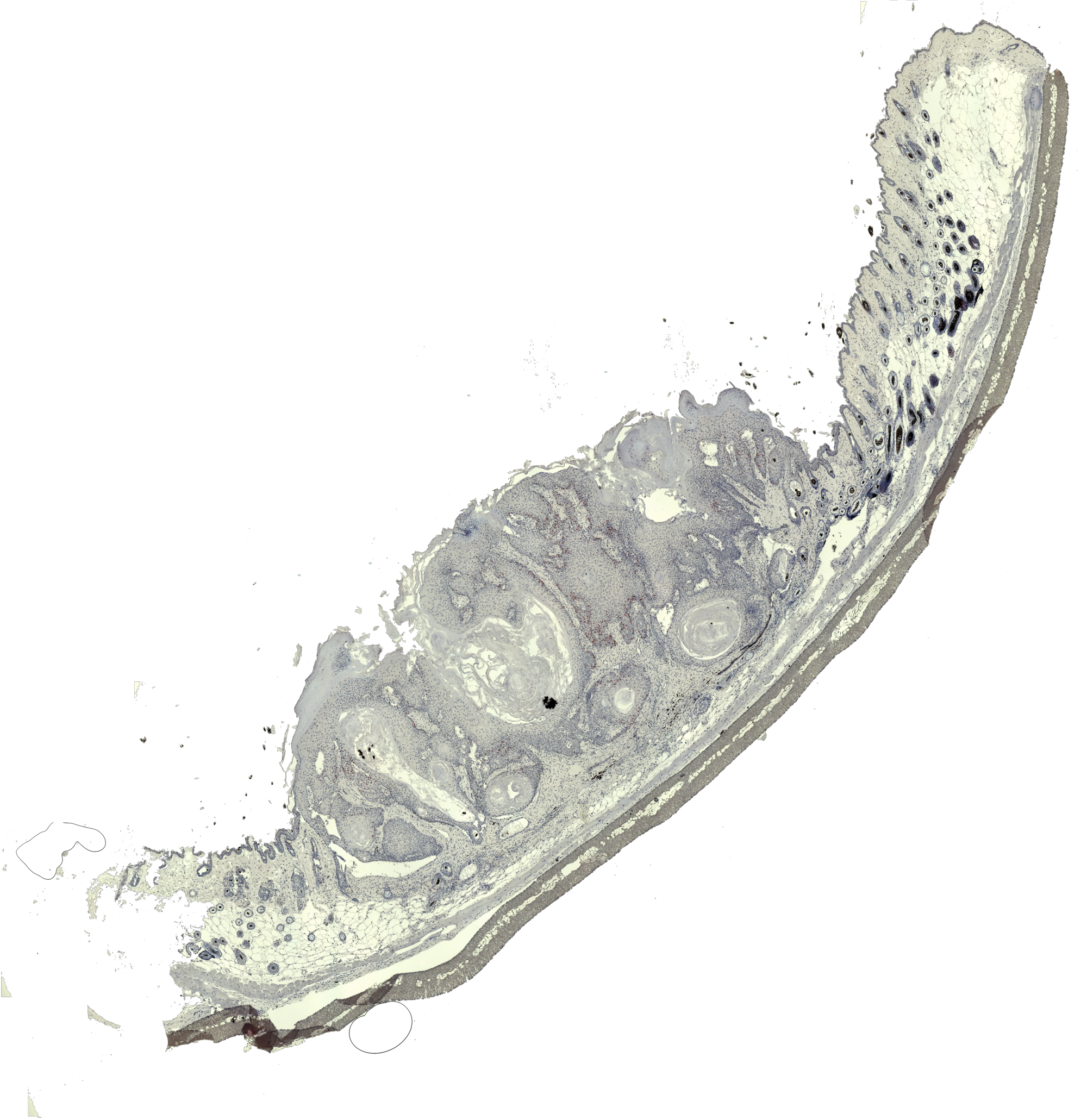

Supplement: Supplementary file 7 — Source Data for Figure 2 [file EMMM-15-e17761-s006.zip › Figure 2/2A/micr.image_BrdU_wt.tif]

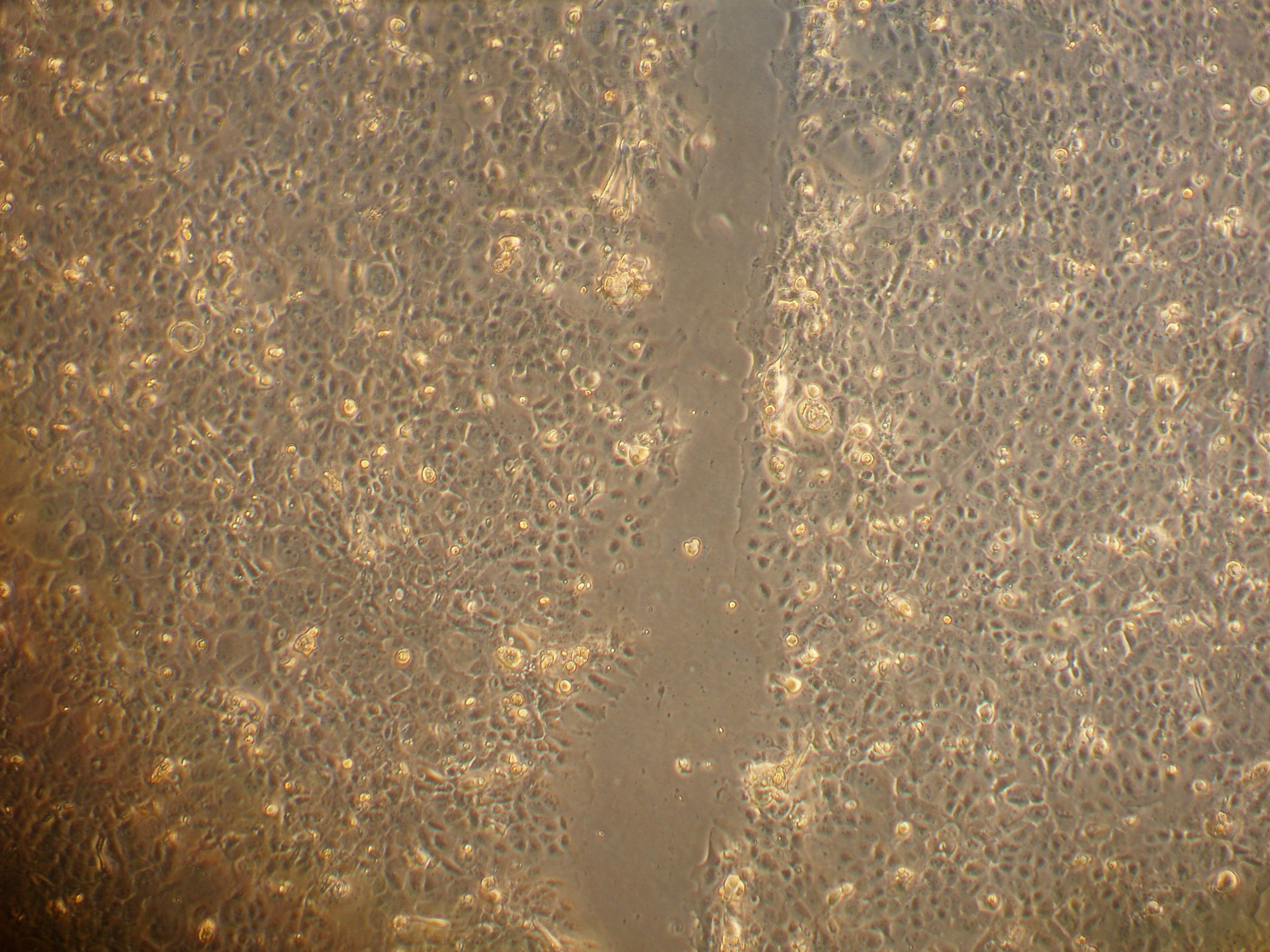

Supplement: Supplementary file 8 — Source Data for Figure 3 [file EMMM-15-e17761-s002.zip › Figure 3/3D/micr.image_E64 9h SCC13 EV C1 1.JPG]

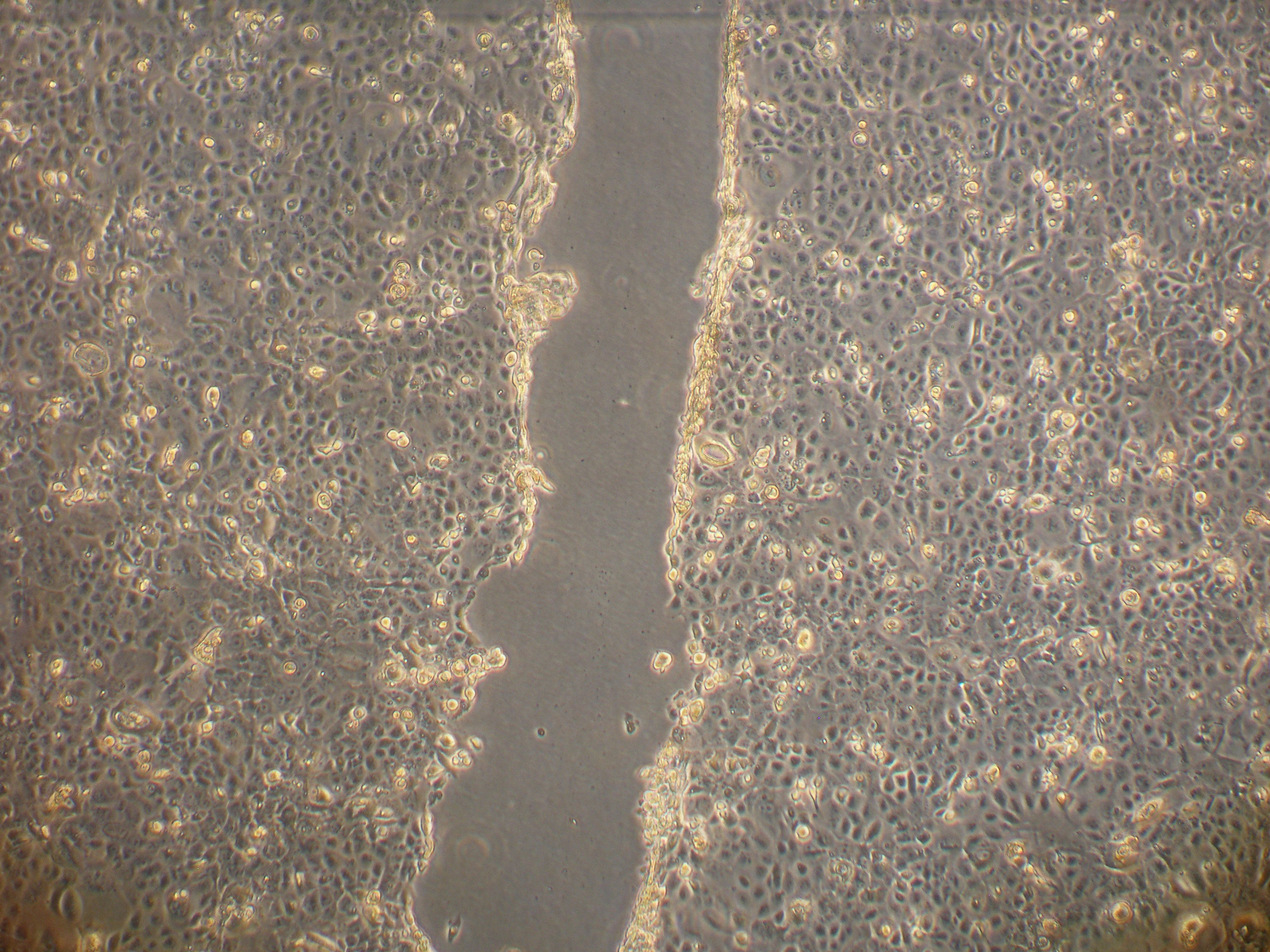

Supplement: Supplementary file 8 — Source Data for Figure 3 [file EMMM-15-e17761-s002.zip › Figure 3/3D/micr.image_E64 0h SCC13 EV C1 1.JPG]

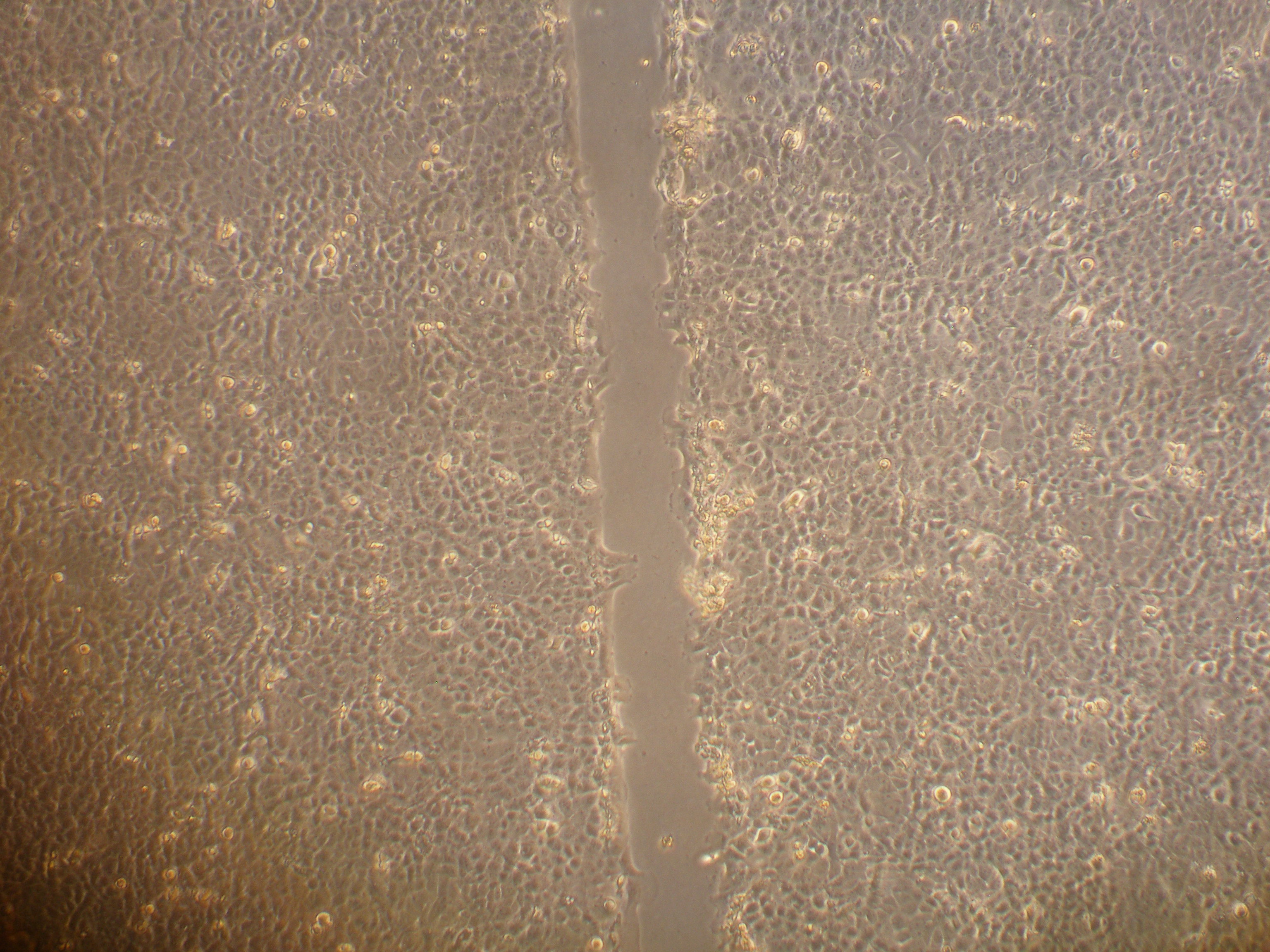

Supplement: Supplementary file 8 — Source Data for Figure 3 [file EMMM-15-e17761-s002.zip › Figure 3/3D/micr.image_E64 9h SCC13 sg1 C4 2.JPG]

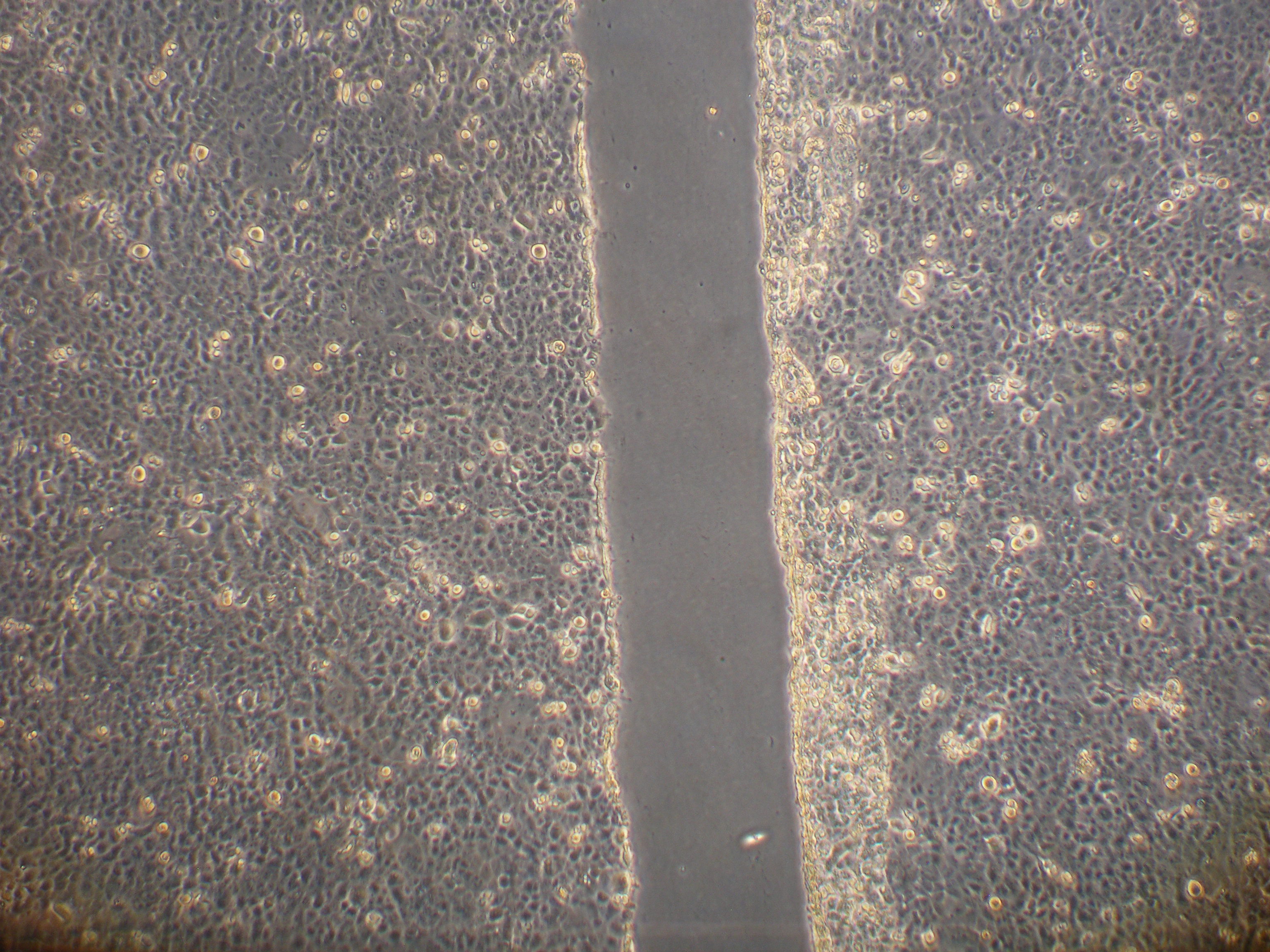

Supplement: Supplementary file 8 — Source Data for Figure 3 [file EMMM-15-e17761-s002.zip › Figure 3/3D/micr.image_E64 0h SCC13 sg1 C4 2.JPG]

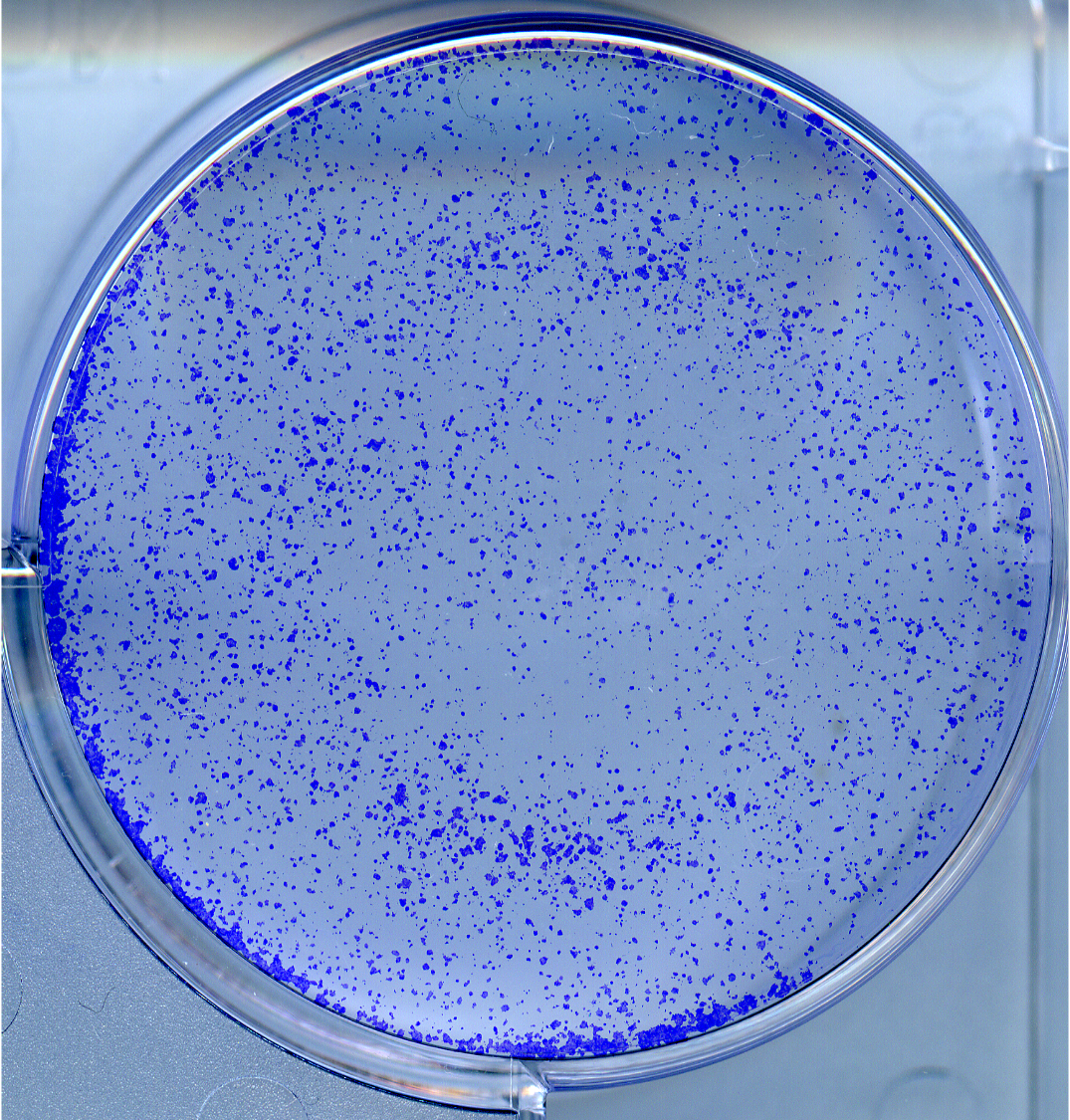

Supplement: Supplementary file 8 — Source Data for Figure 3 [file EMMM-15-e17761-s002.zip › Figure 3/3B/image_KO C4.png]

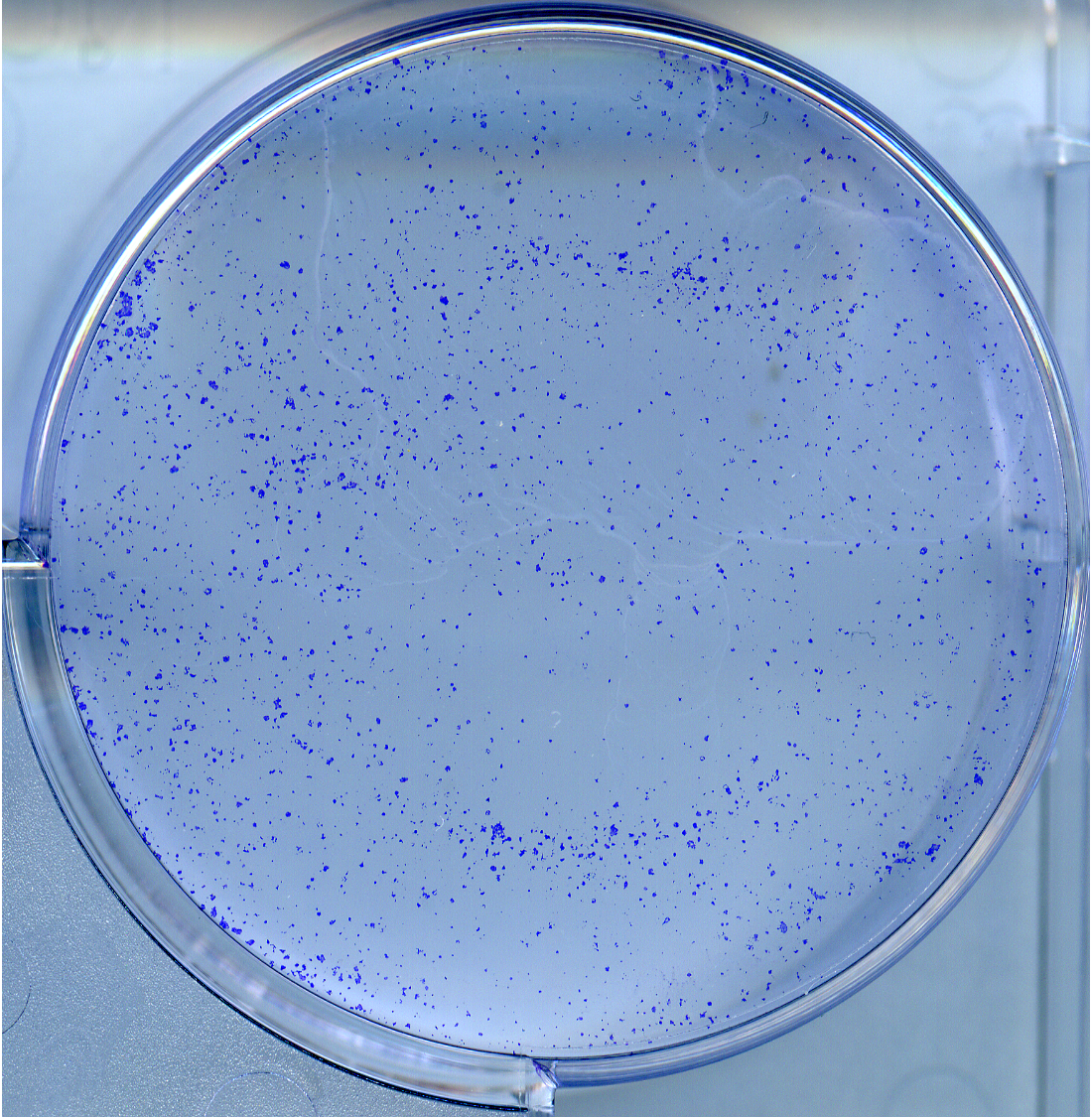

Supplement: Supplementary file 8 — Source Data for Figure 3 [file EMMM-15-e17761-s002.zip › Figure 3/3B/image_EV C1.png]

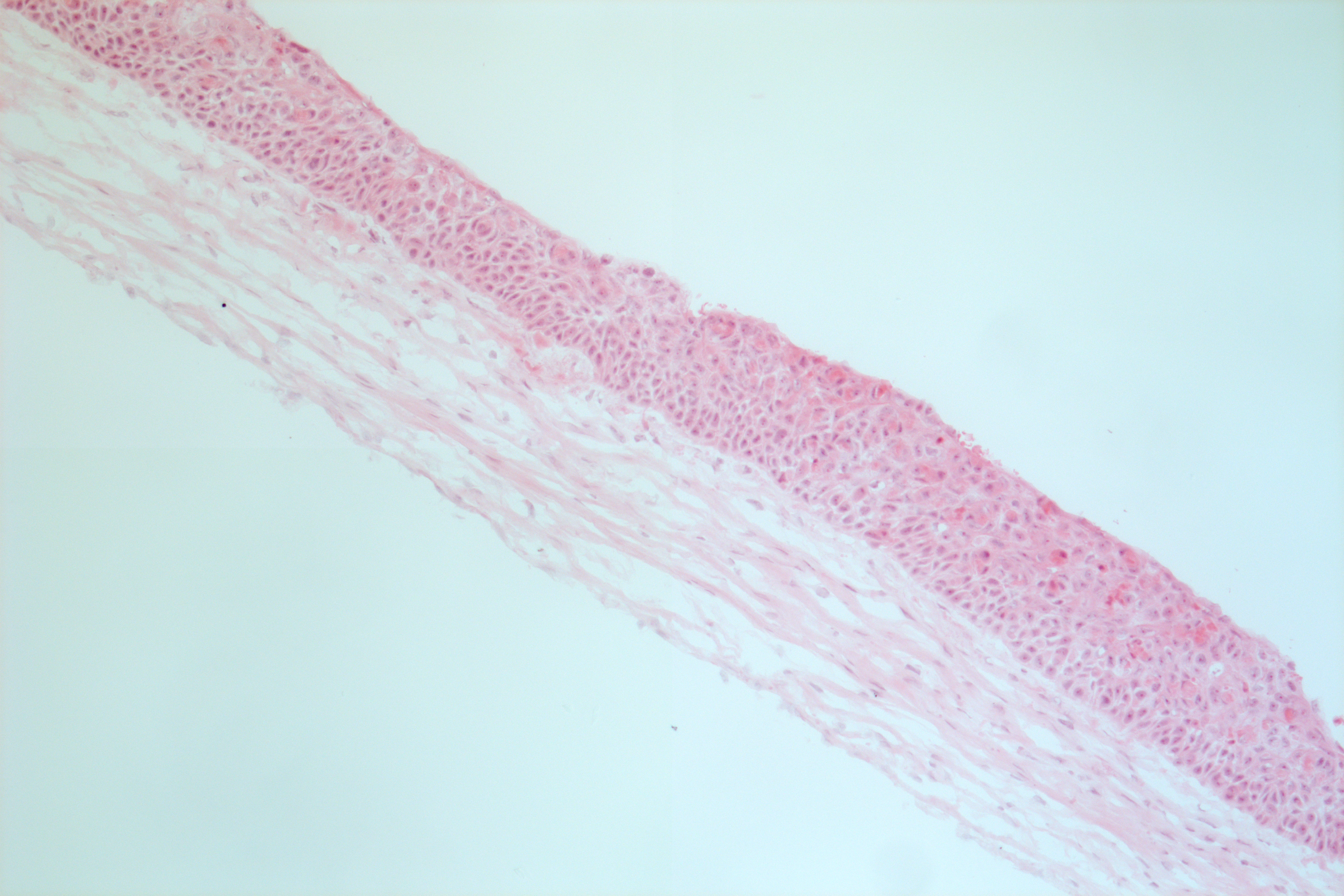

Supplement: Supplementary file 8 — Source Data for Figure 3 [file EMMM-15-e17761-s002.zip › Figure 3/3J/micr.image_EV C1.tiff]

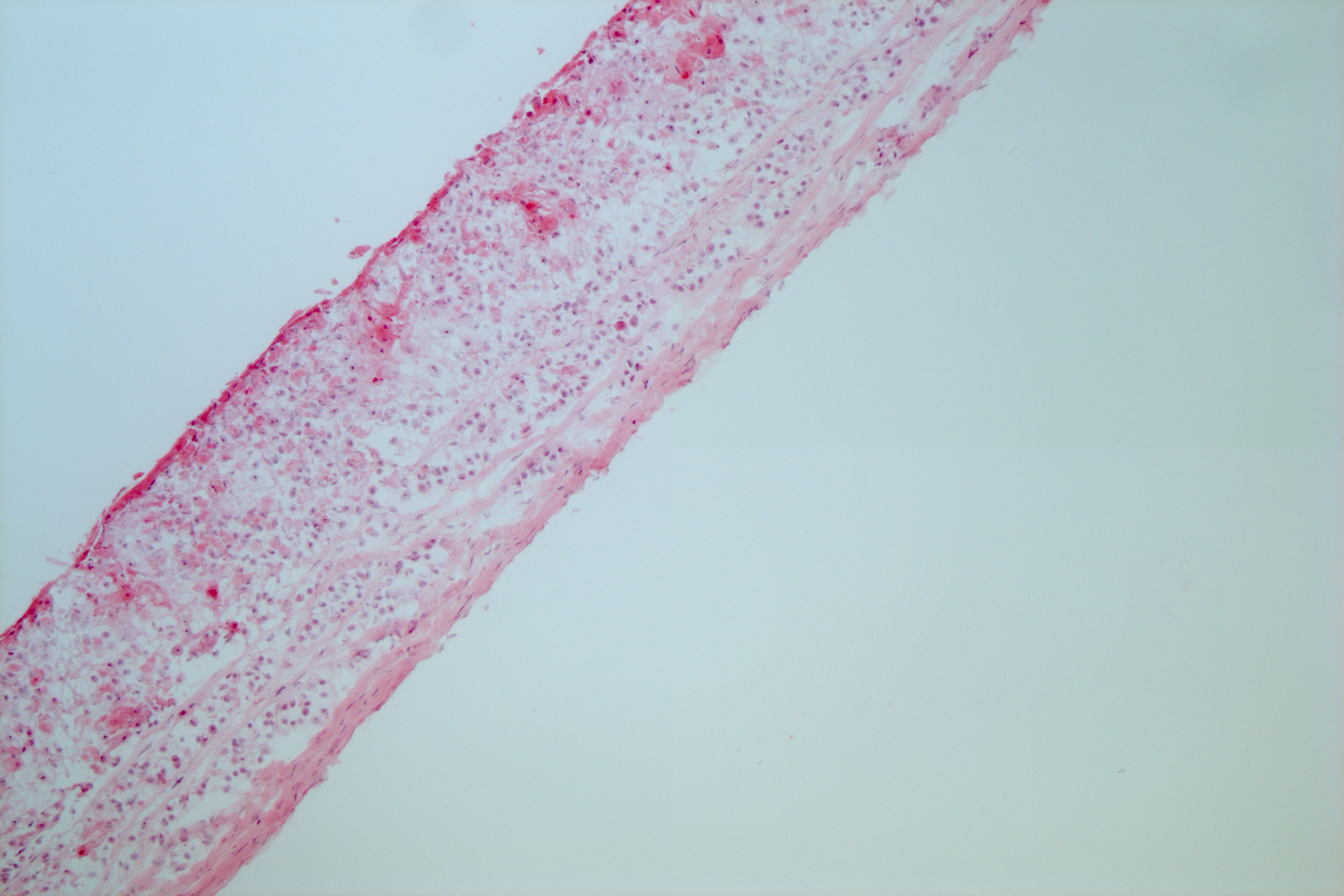

Supplement: Supplementary file 8 — Source Data for Figure 3 [file EMMM-15-e17761-s002.zip › Figure 3/3J/micr.image_KO C4.tiff]

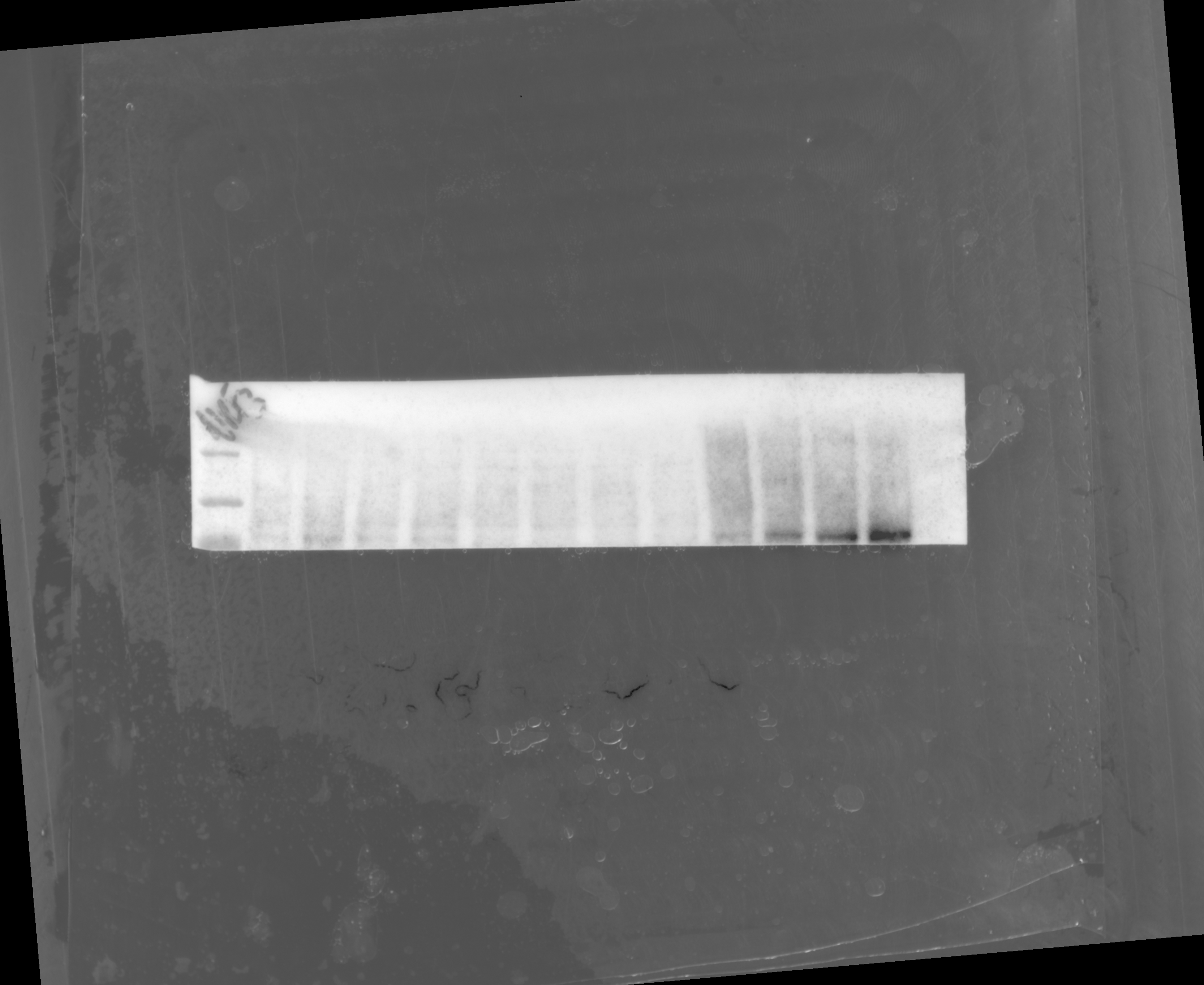

Supplement: Supplementary file 8 — Source Data for Figure 3 [file EMMM-15-e17761-s002.zip › Figure 3/3F/western NRF3 marker.Tif]

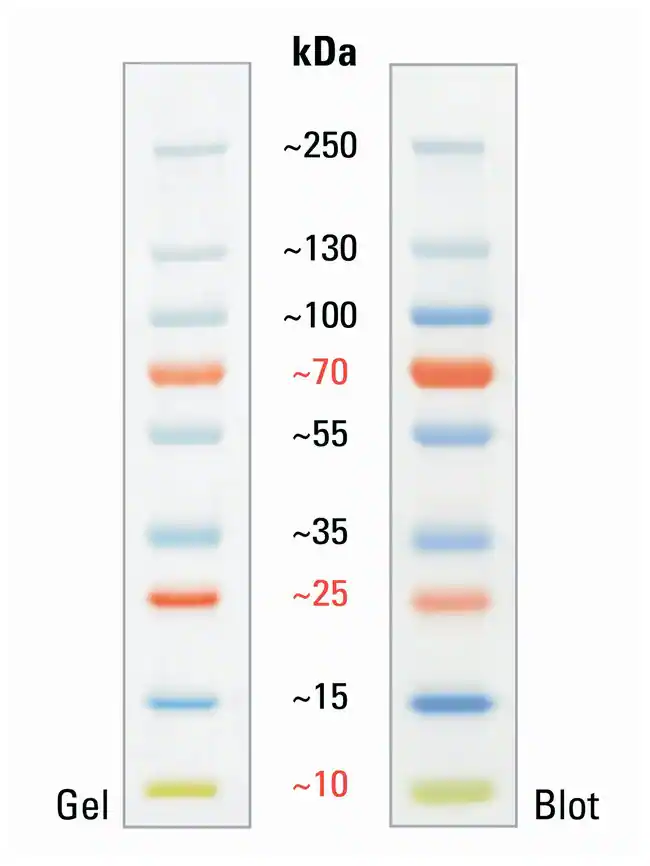

Supplement: Supplementary file 8 — Source Data for Figure 3 [file EMMM-15-e17761-s002.zip › Figure 3/3F/WB marker.webp]

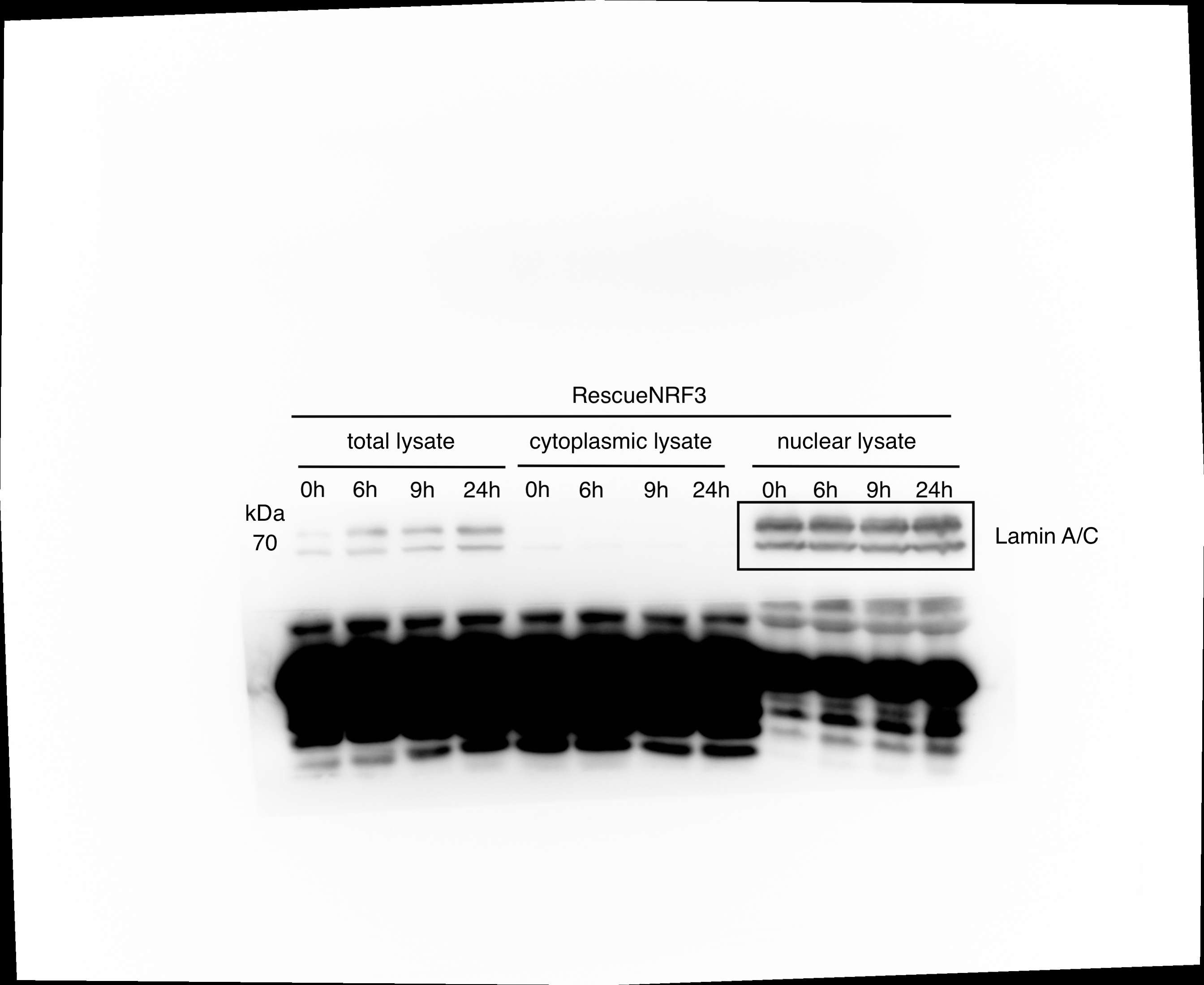

Supplement: Supplementary file 8 — Source Data for Figure 3 [file EMMM-15-e17761-s002.zip › Figure 3/3F/western lamin A:C labelled.png]

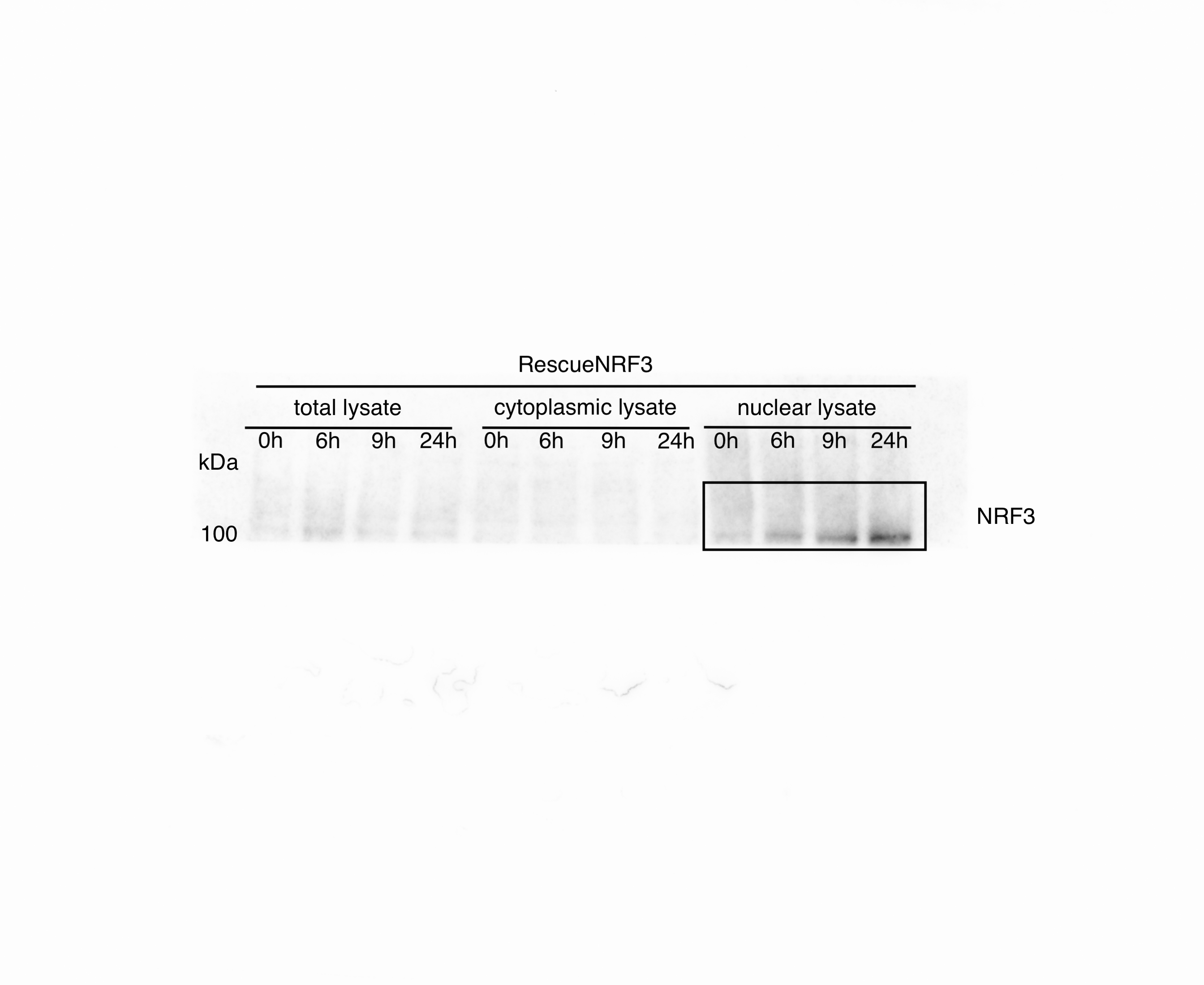

Supplement: Supplementary file 8 — Source Data for Figure 3 [file EMMM-15-e17761-s002.zip › Figure 3/3F/western NRF3 labelled.png]

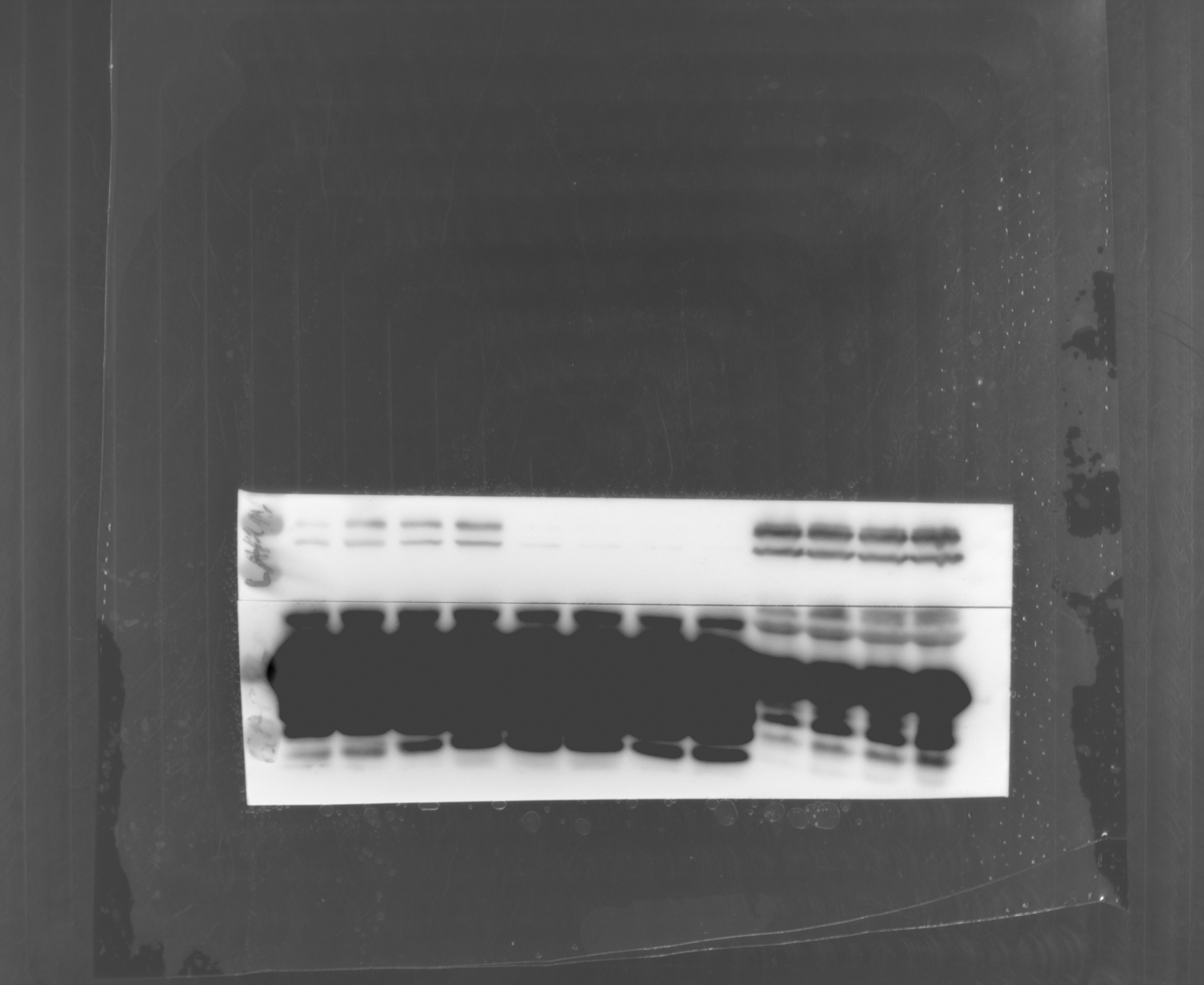

Supplement: Supplementary file 8 — Source Data for Figure 3 [file EMMM-15-e17761-s002.zip › Figure 3/3F/western lamin marker.Tif]

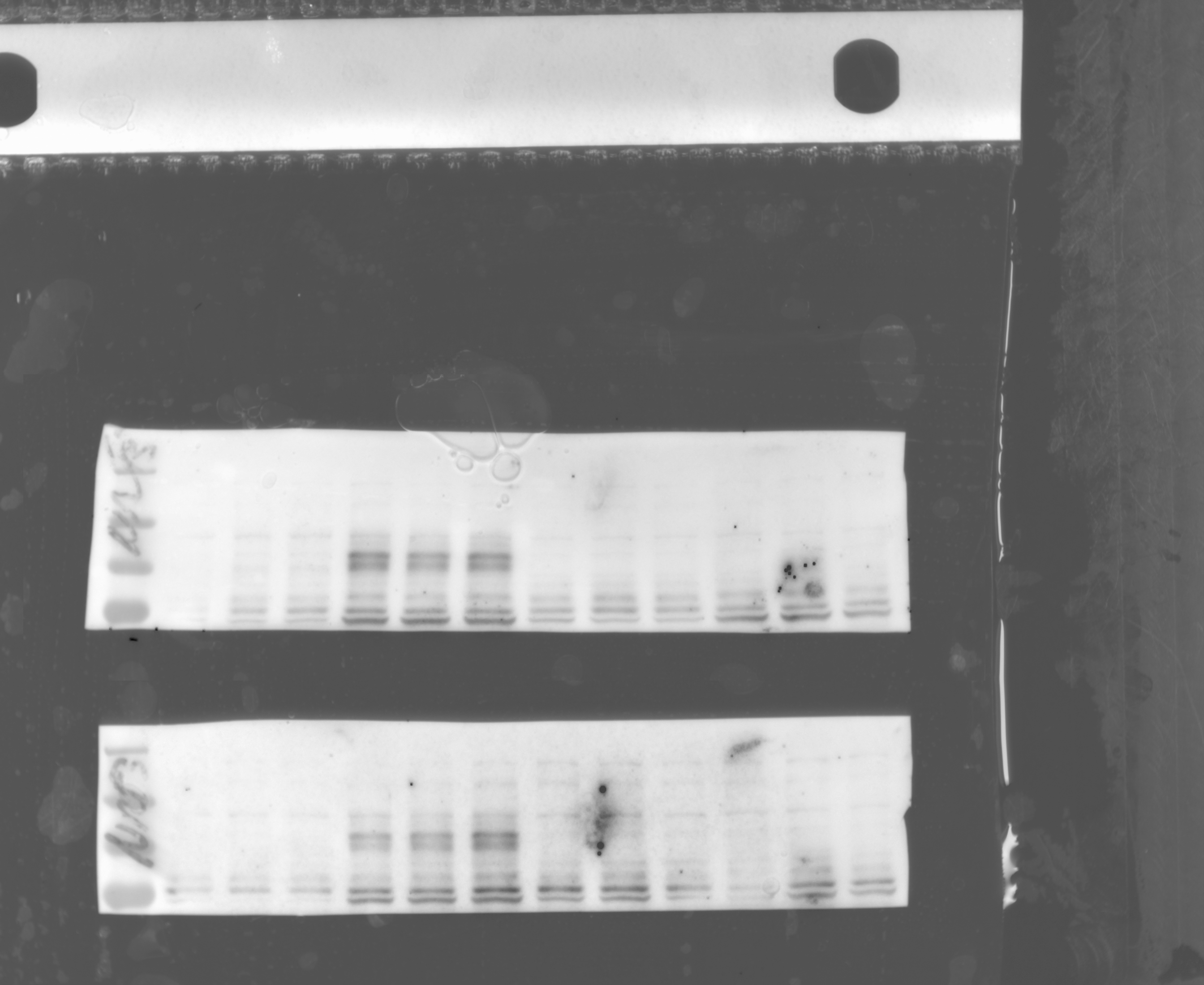

Supplement: Supplementary file 8 — Source Data for Figure 3 [file EMMM-15-e17761-s002.zip › Figure 3/3A/western NRF3 marker.Tif]

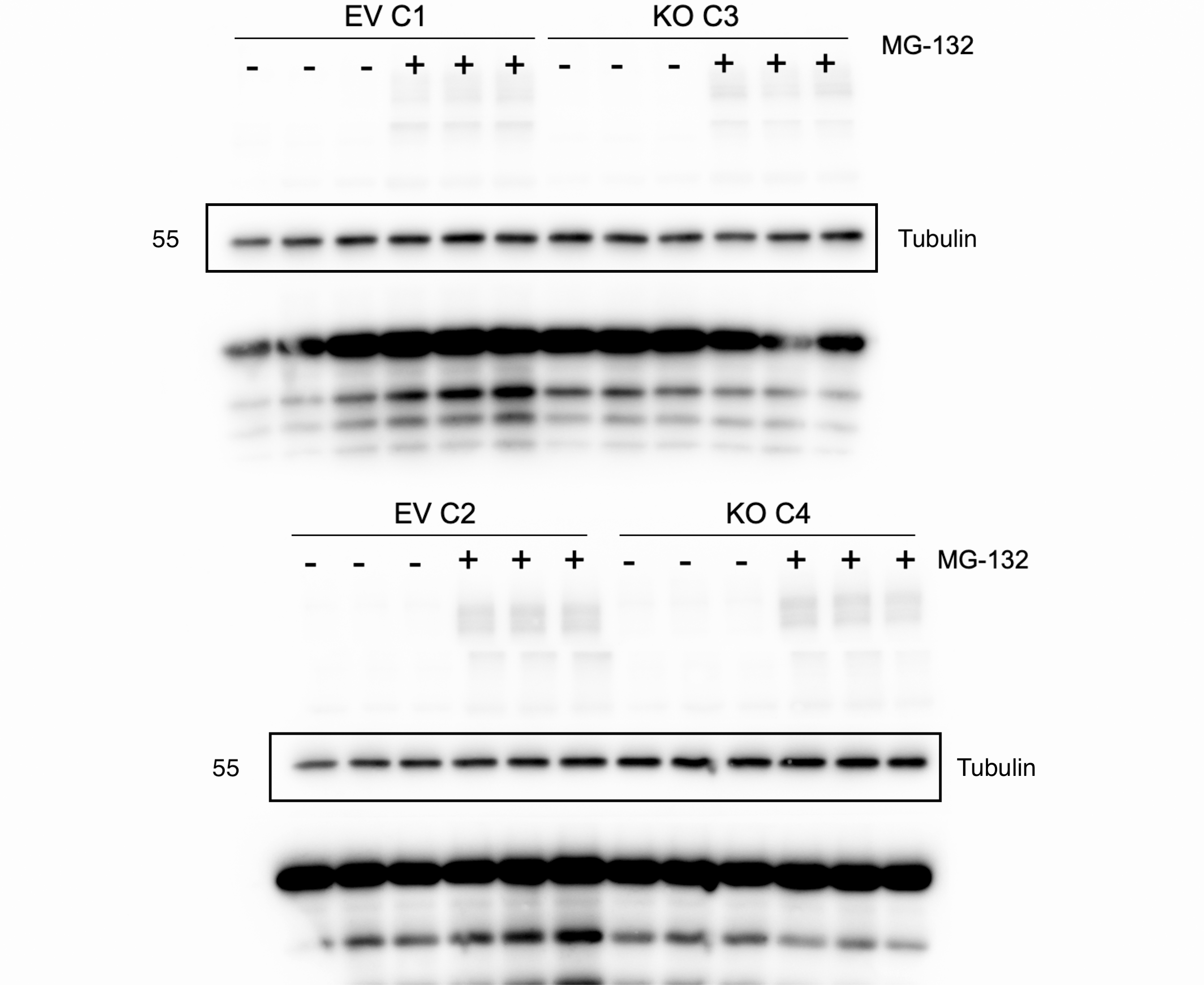

Supplement: Supplementary file 8 — Source Data for Figure 3 [file EMMM-15-e17761-s002.zip › Figure 3/3A/western tubulin labelled.png]

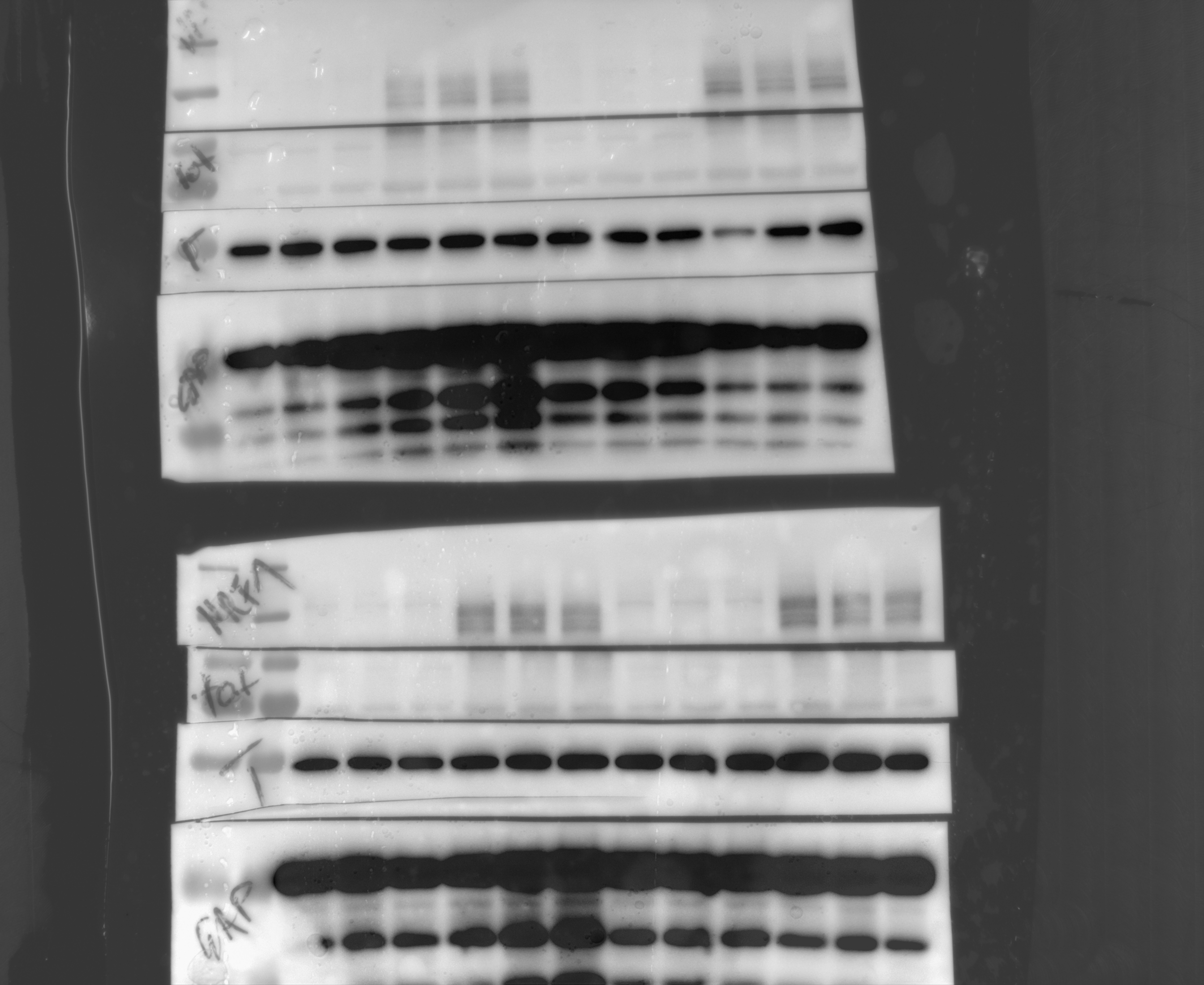

Supplement: Supplementary file 8 — Source Data for Figure 3 [file EMMM-15-e17761-s002.zip › Figure 3/3A/western NRF1 tubulin marker.Tif]

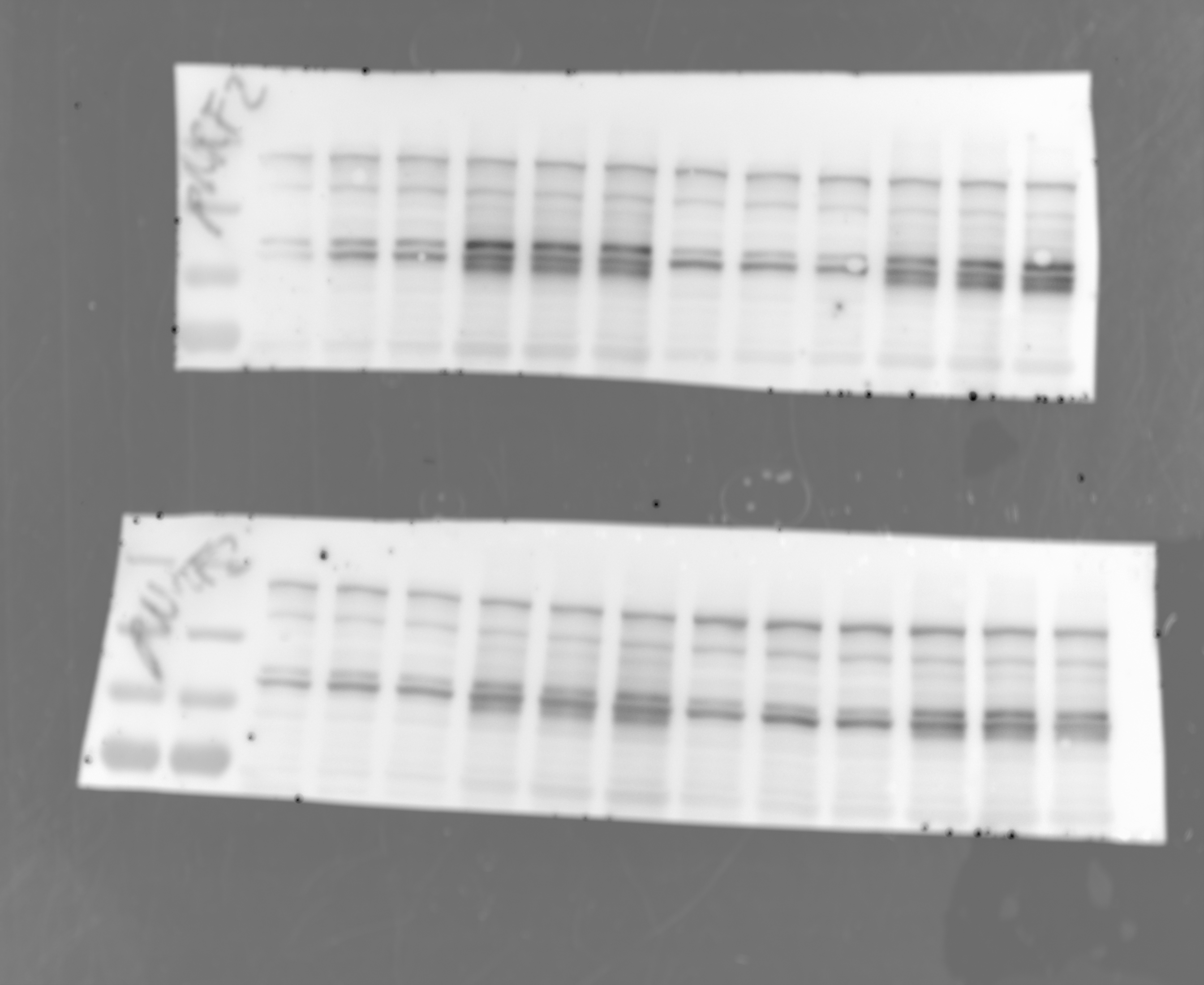

Supplement: Supplementary file 8 — Source Data for Figure 3 [file EMMM-15-e17761-s002.zip › Figure 3/3A/western NRF2 marker.Tif]

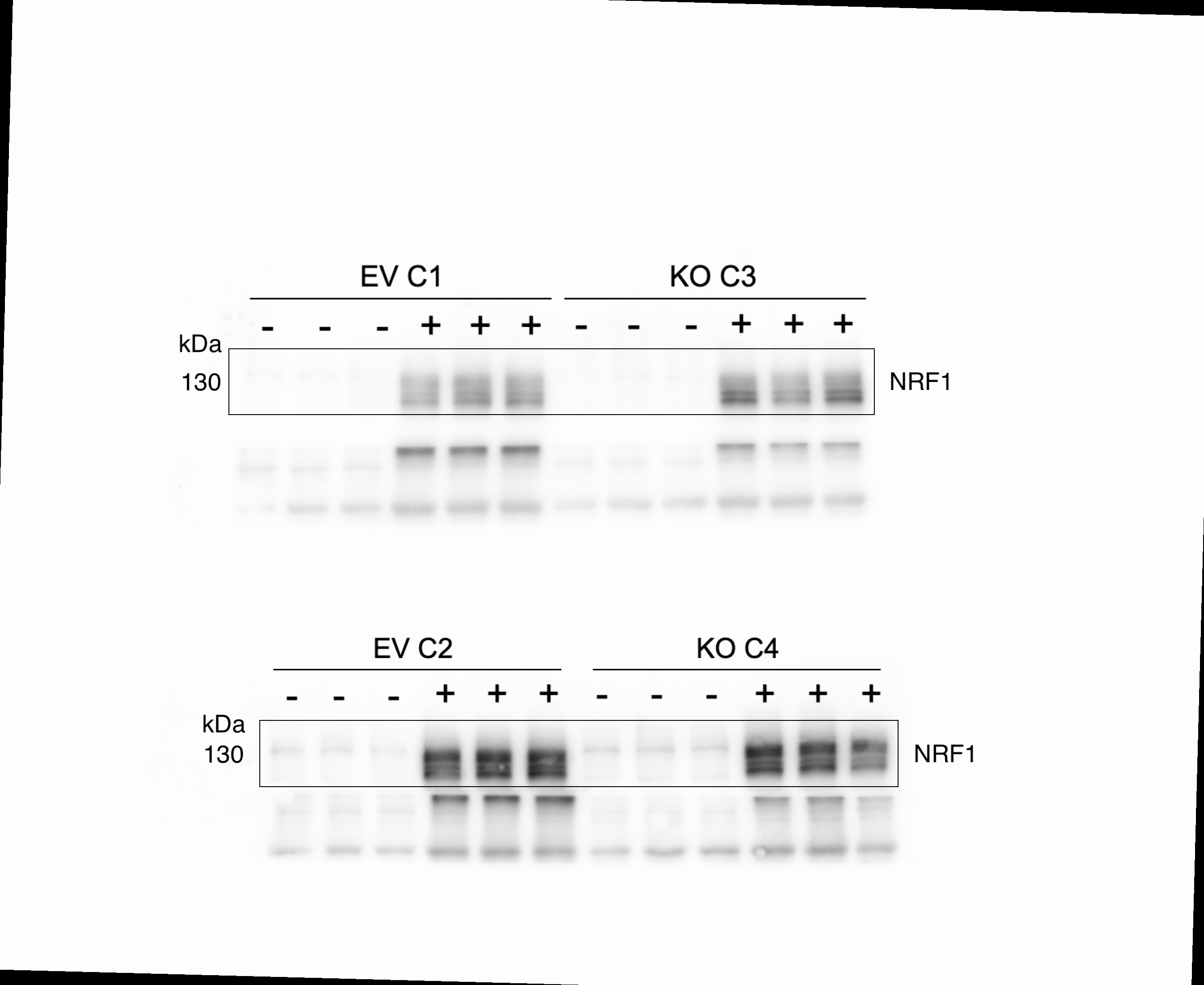

Supplement: Supplementary file 8 — Source Data for Figure 3 [file EMMM-15-e17761-s002.zip › Figure 3/3A/western NRF1 labelled.png]

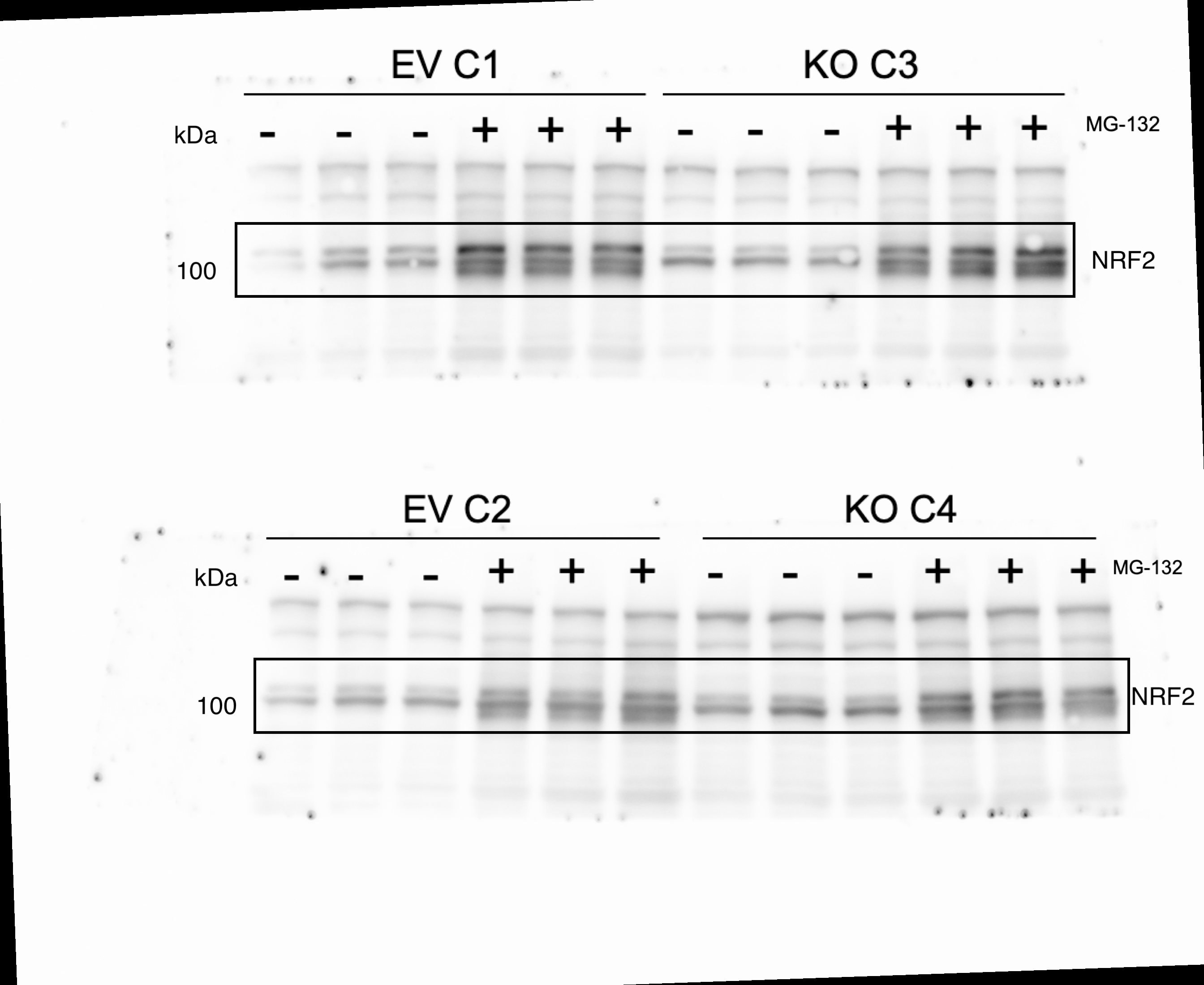

Supplement: Supplementary file 8 — Source Data for Figure 3 [file EMMM-15-e17761-s002.zip › Figure 3/3A/western NRF2 labelled.png]

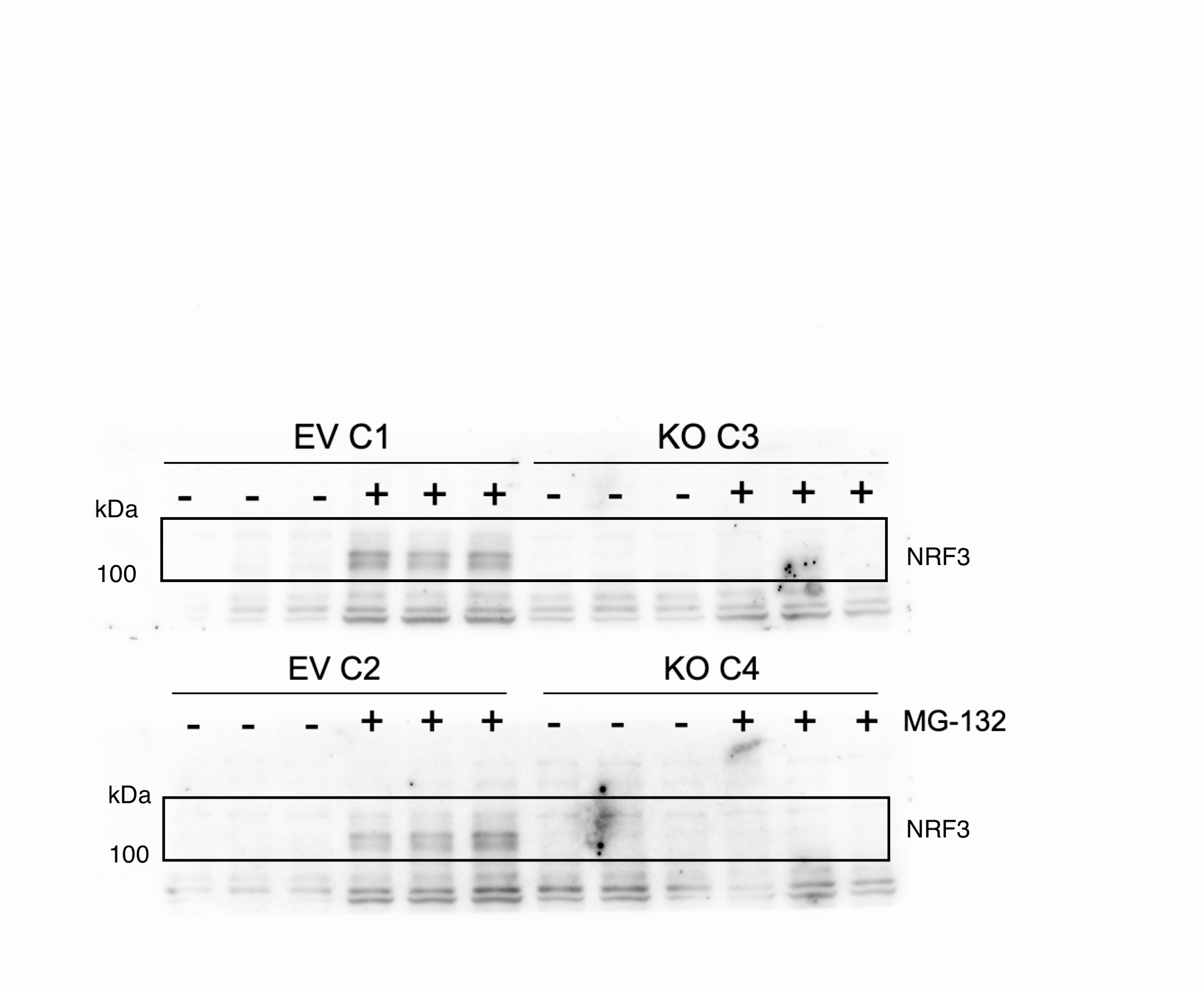

Supplement: Supplementary file 8 — Source Data for Figure 3 [file EMMM-15-e17761-s002.zip › Figure 3/3A/western NRF3 labelled.png]

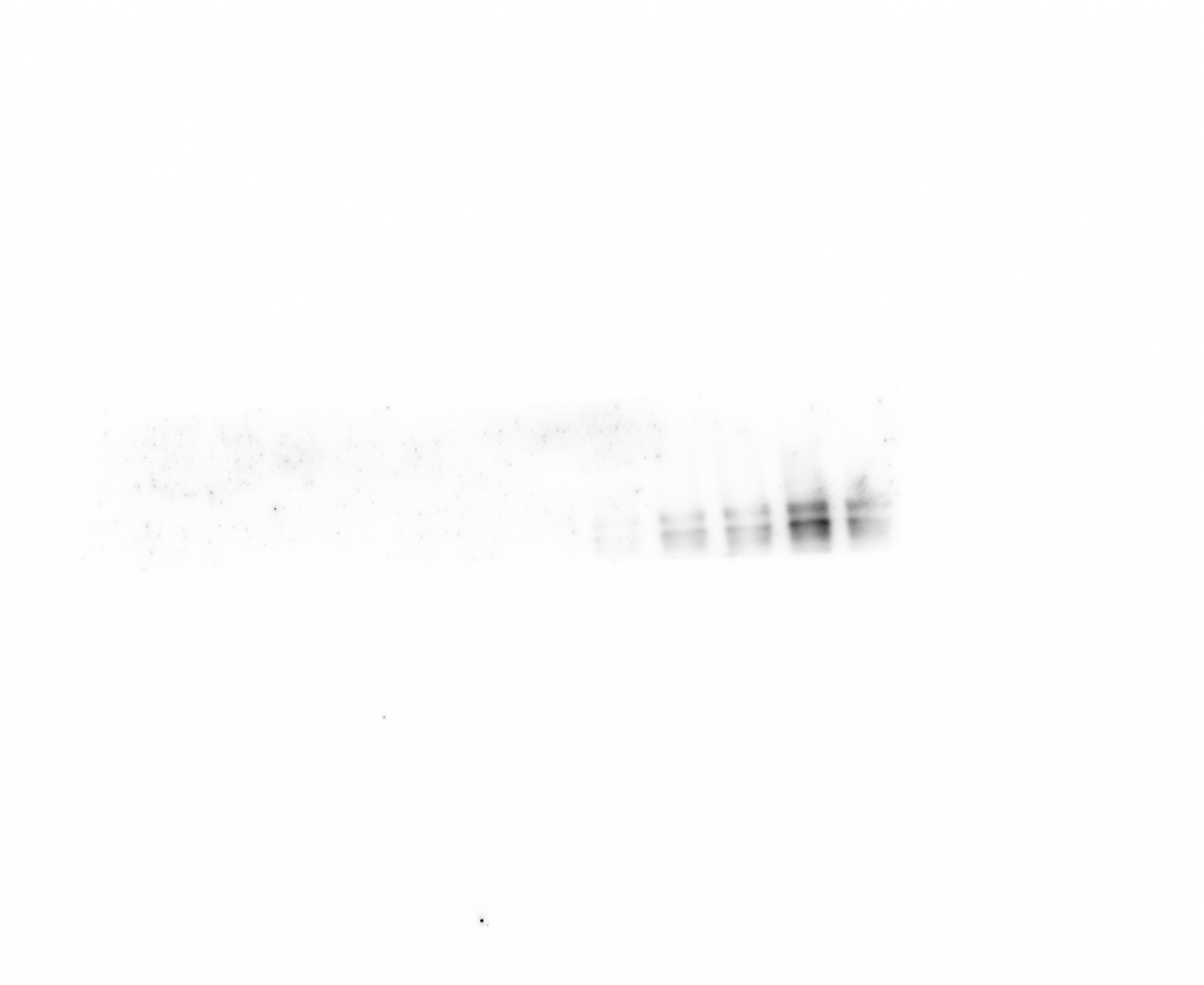

Supplement: Supplementary file 8 — Source Data for Figure 3 [file EMMM-15-e17761-s002.zip › Figure 3/3E/western NRF3.Tif]

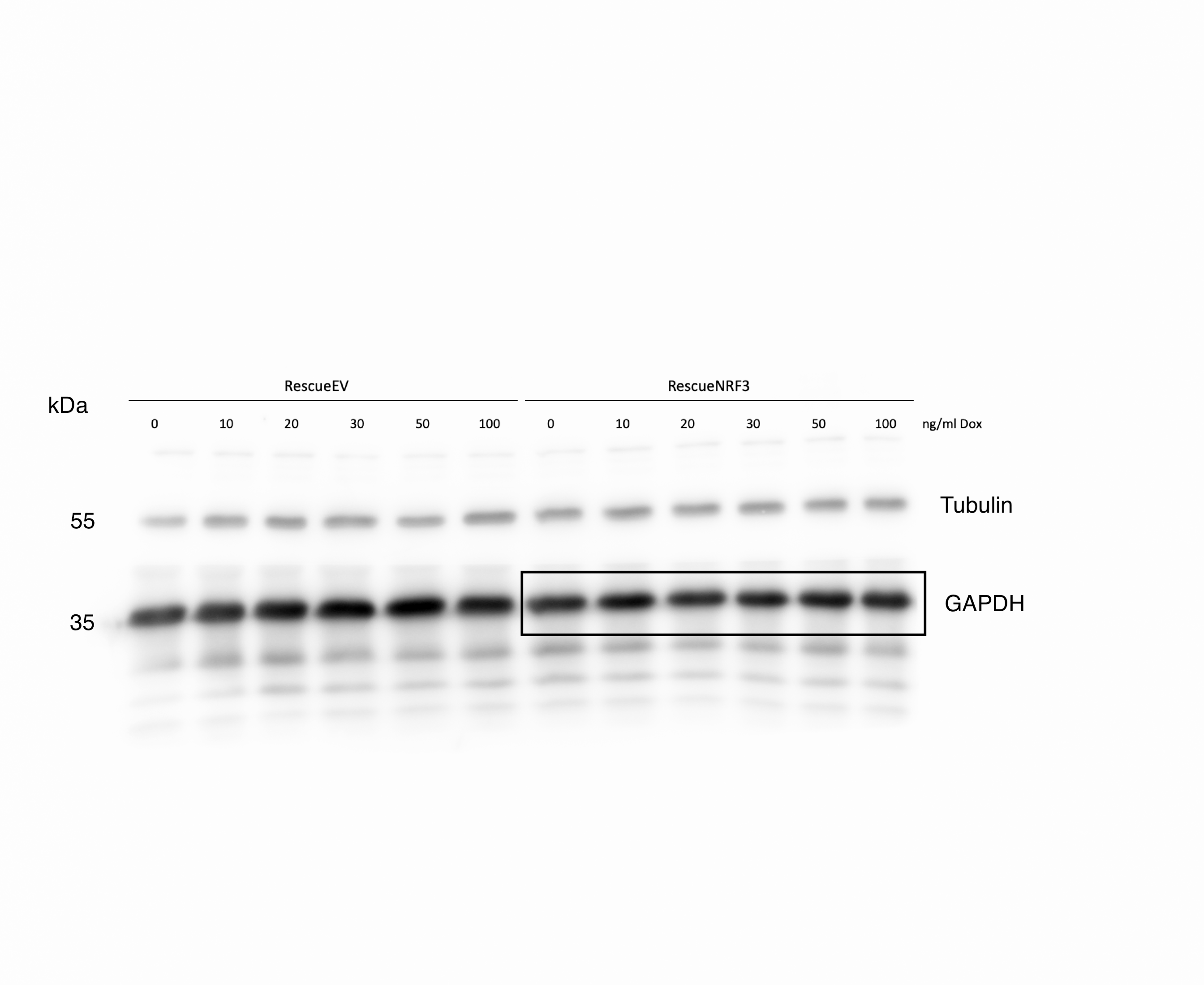

Supplement: Supplementary file 8 — Source Data for Figure 3 [file EMMM-15-e17761-s002.zip › Figure 3/3E/western GAPDH labelled.png]

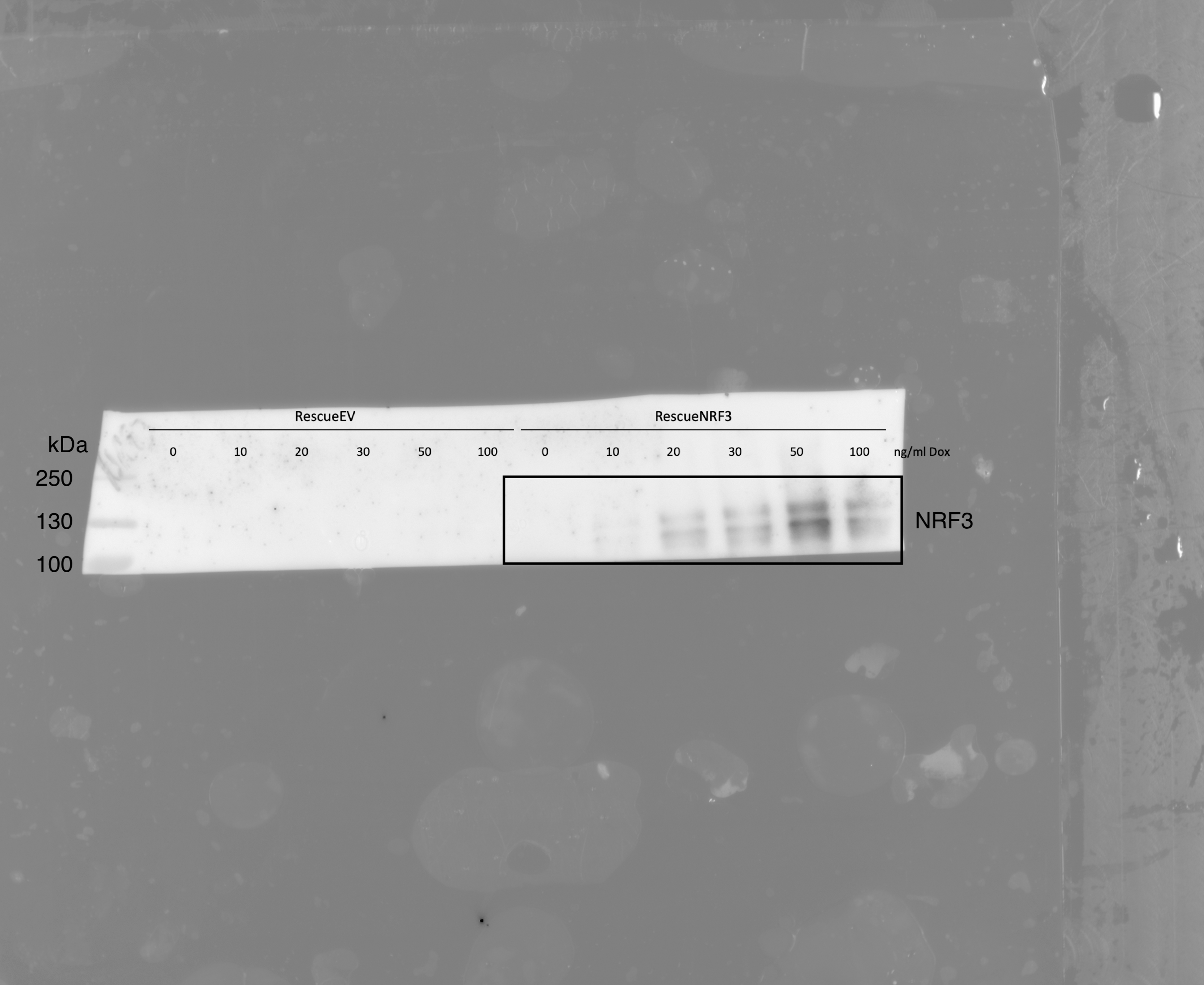

Supplement: Supplementary file 8 — Source Data for Figure 3 [file EMMM-15-e17761-s002.zip › Figure 3/3E/western NRF3 marker labelled.png]

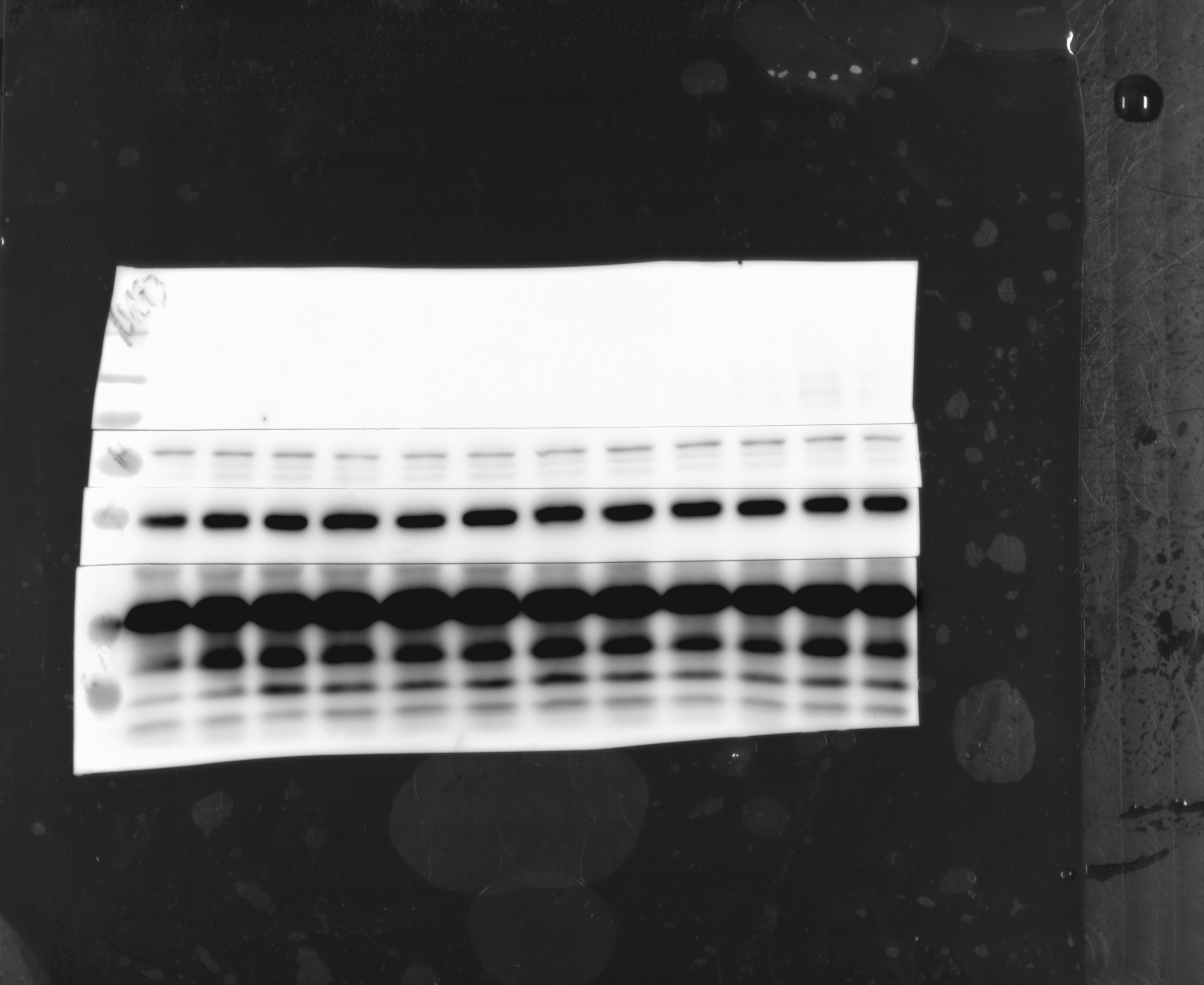

Supplement: Supplementary file 8 — Source Data for Figure 3 [file EMMM-15-e17761-s002.zip › Figure 3/3E/western GAPDH marker.Tif]

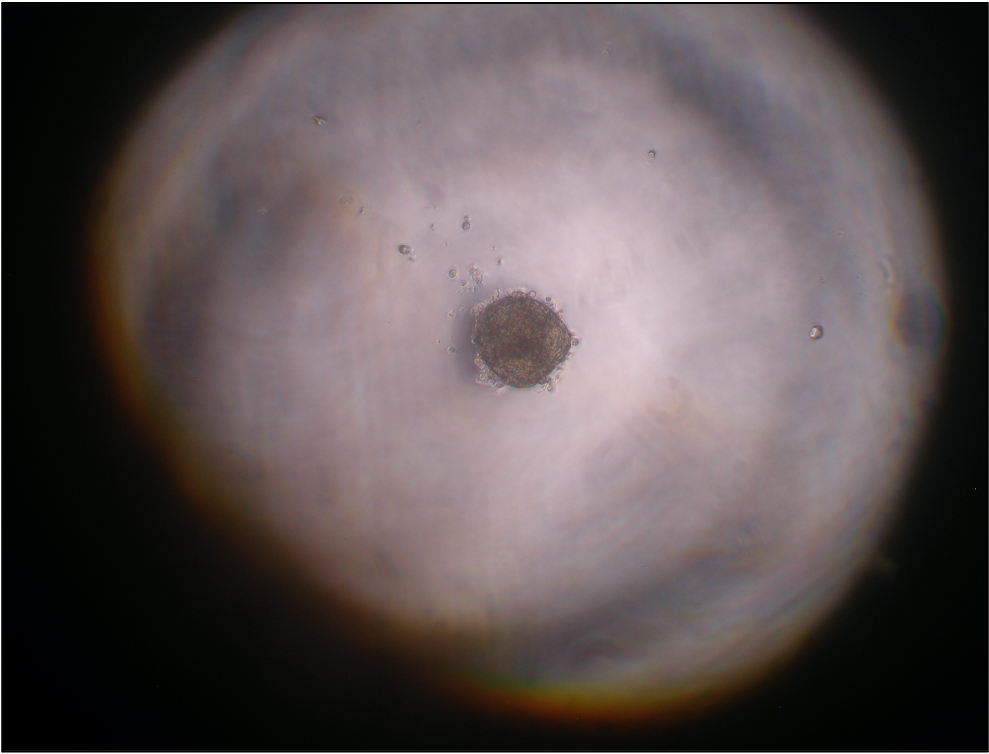

Supplement: Supplementary file 8 — Source Data for Figure 3 [file EMMM-15-e17761-s002.zip › Figure 3/3H/micr.image_EVC1.png]

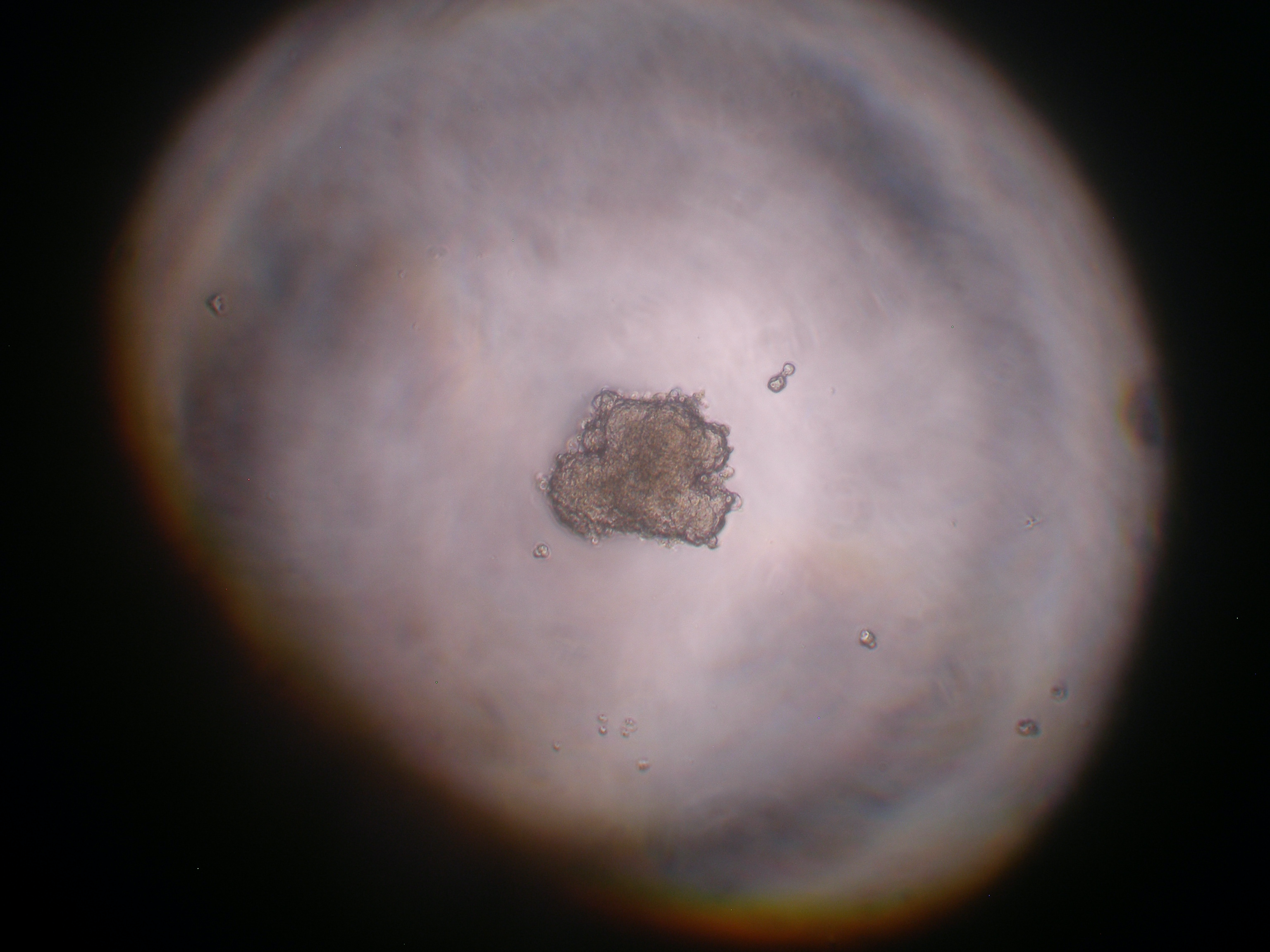

Supplement: Supplementary file 8 — Source Data for Figure 3 [file EMMM-15-e17761-s002.zip › Figure 3/3H/micr.image_KOC4.png]

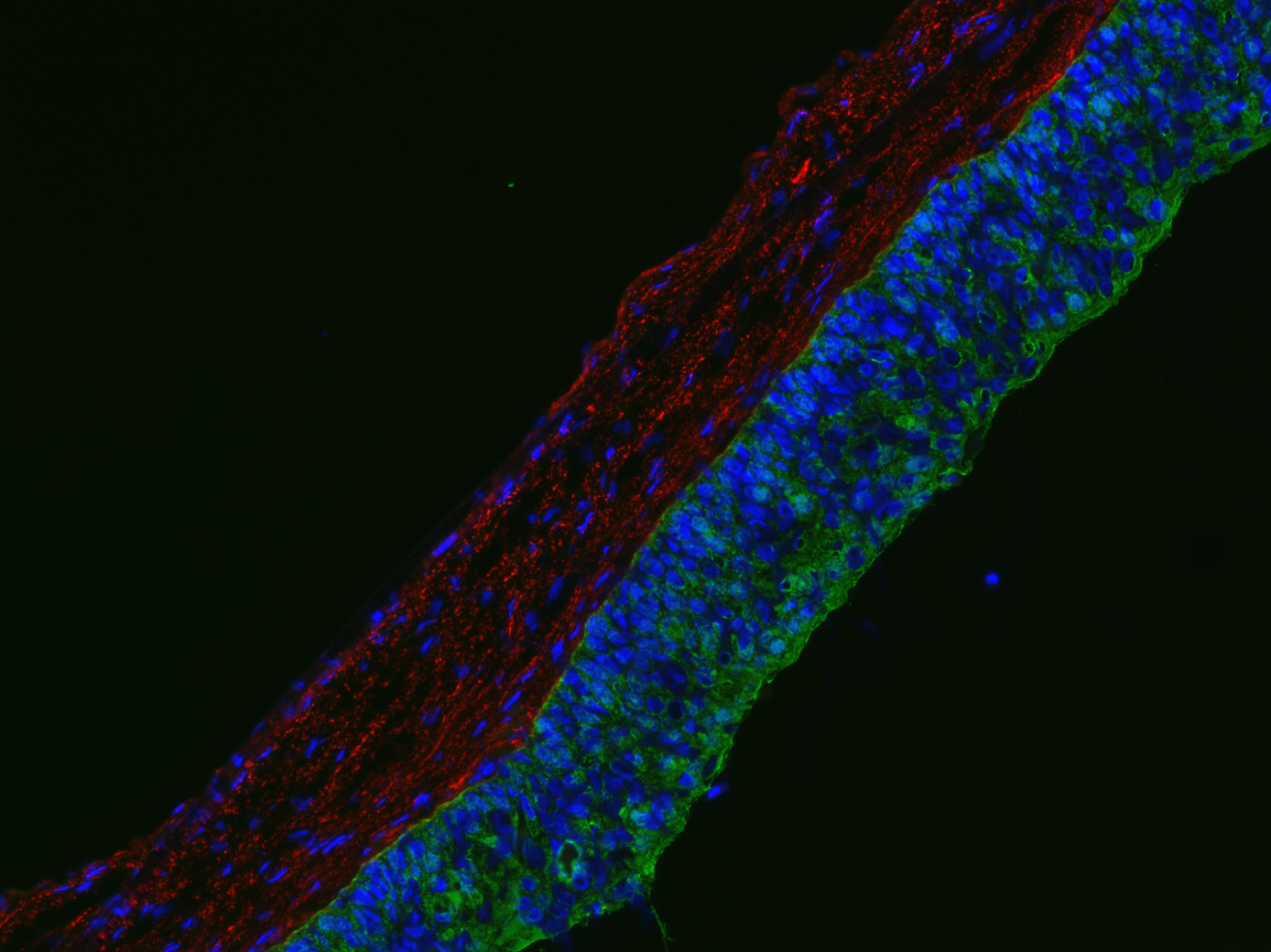

Supplement: Supplementary file 8 — Source Data for Figure 3 [file EMMM-15-e17761-s002.zip › Figure 3/3K/micr.image_EV C1.tif]

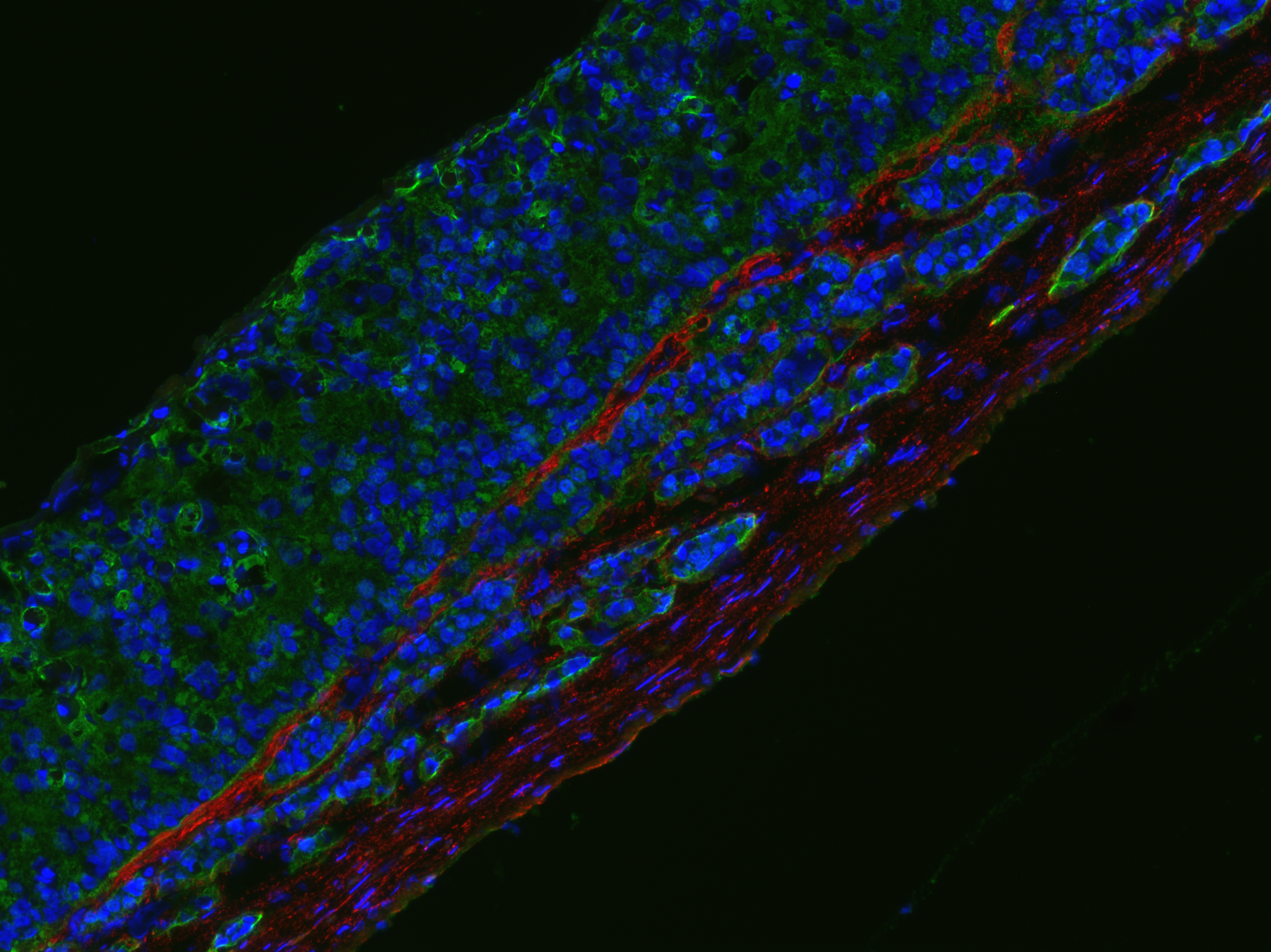

Supplement: Supplementary file 8 — Source Data for Figure 3 [file EMMM-15-e17761-s002.zip › Figure 3/3K/micr.image_KO C4.tif]

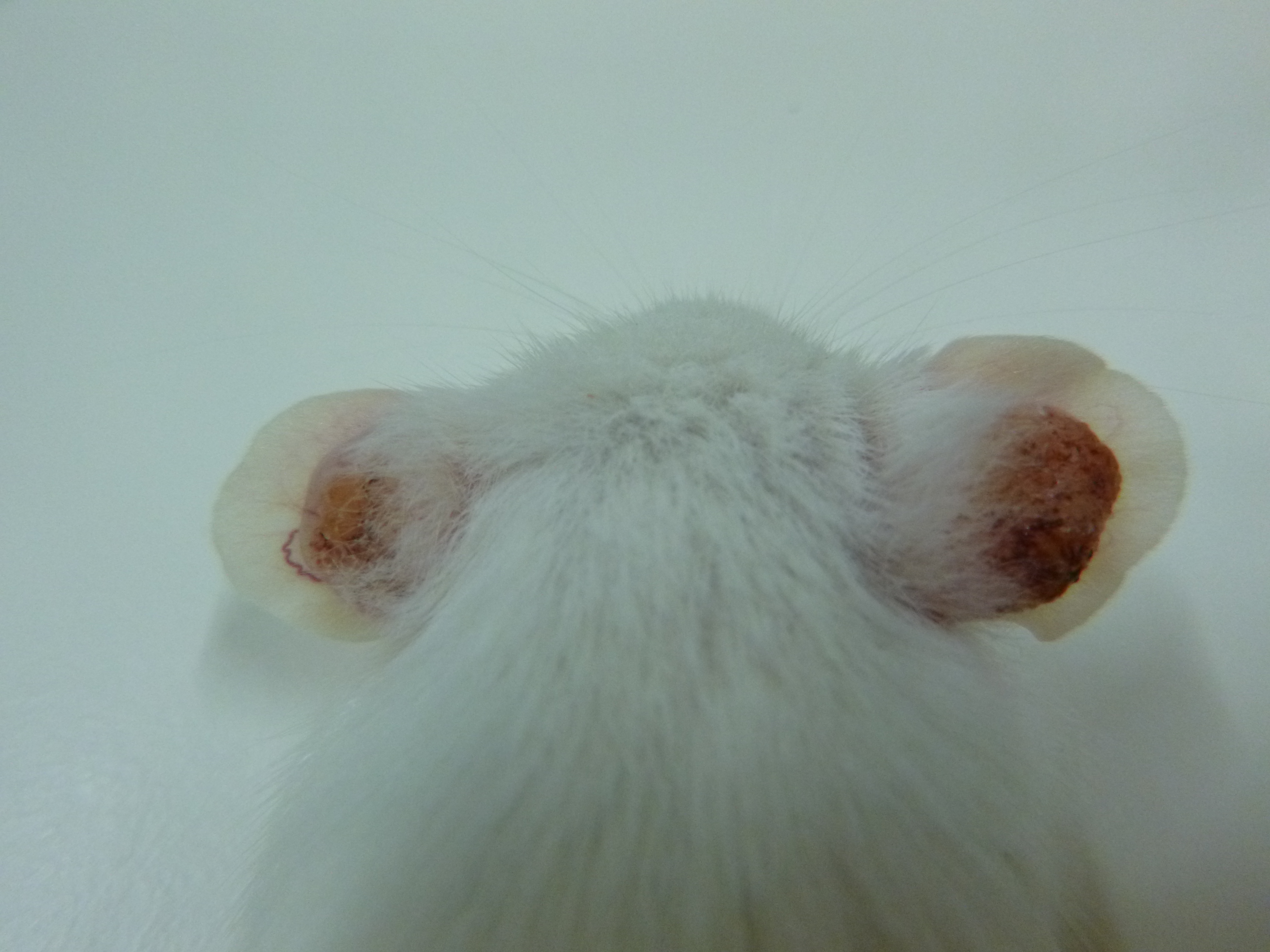

Supplement: Supplementary file 9 — Source Data for Figure 4 [file EMMM-15-e17761-s008.zip › Figure 4/4A/image_mouse ear tumors.JPG]

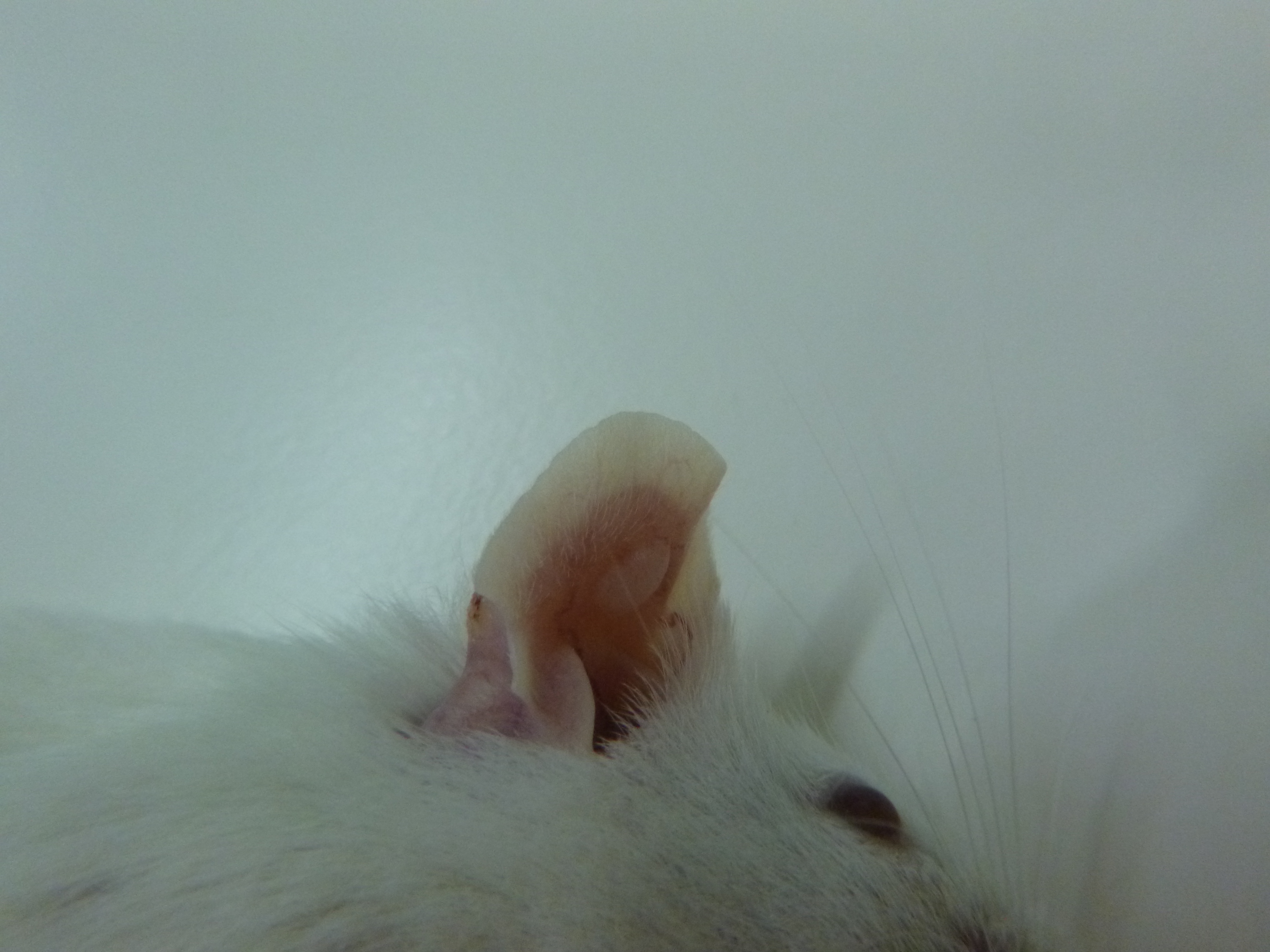

Supplement: Supplementary file 9 — Source Data for Figure 4 [file EMMM-15-e17761-s008.zip › Figure 4/4C/image_4C.JPG]

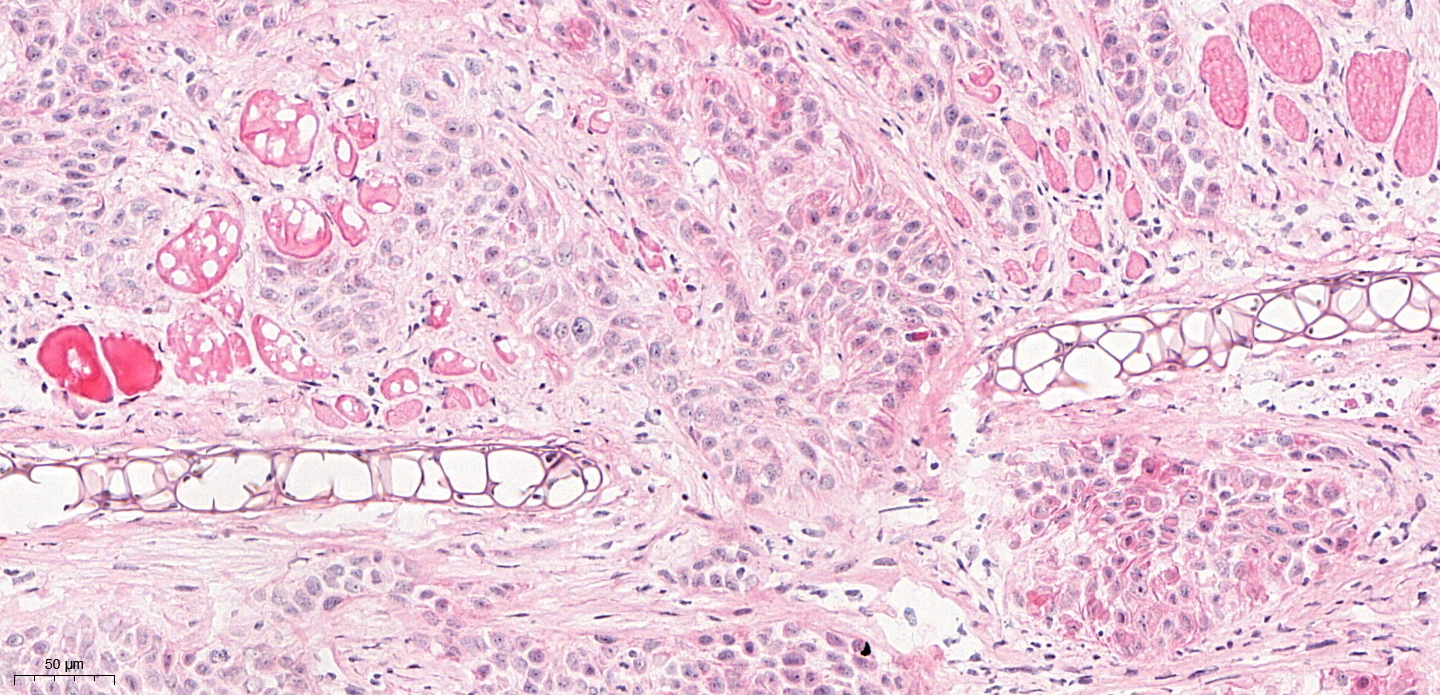

Supplement: Supplementary file 9 — Source Data for Figure 4 [file EMMM-15-e17761-s008.zip › Figure 4/4C/micro.image_magnification 4C.jpg]

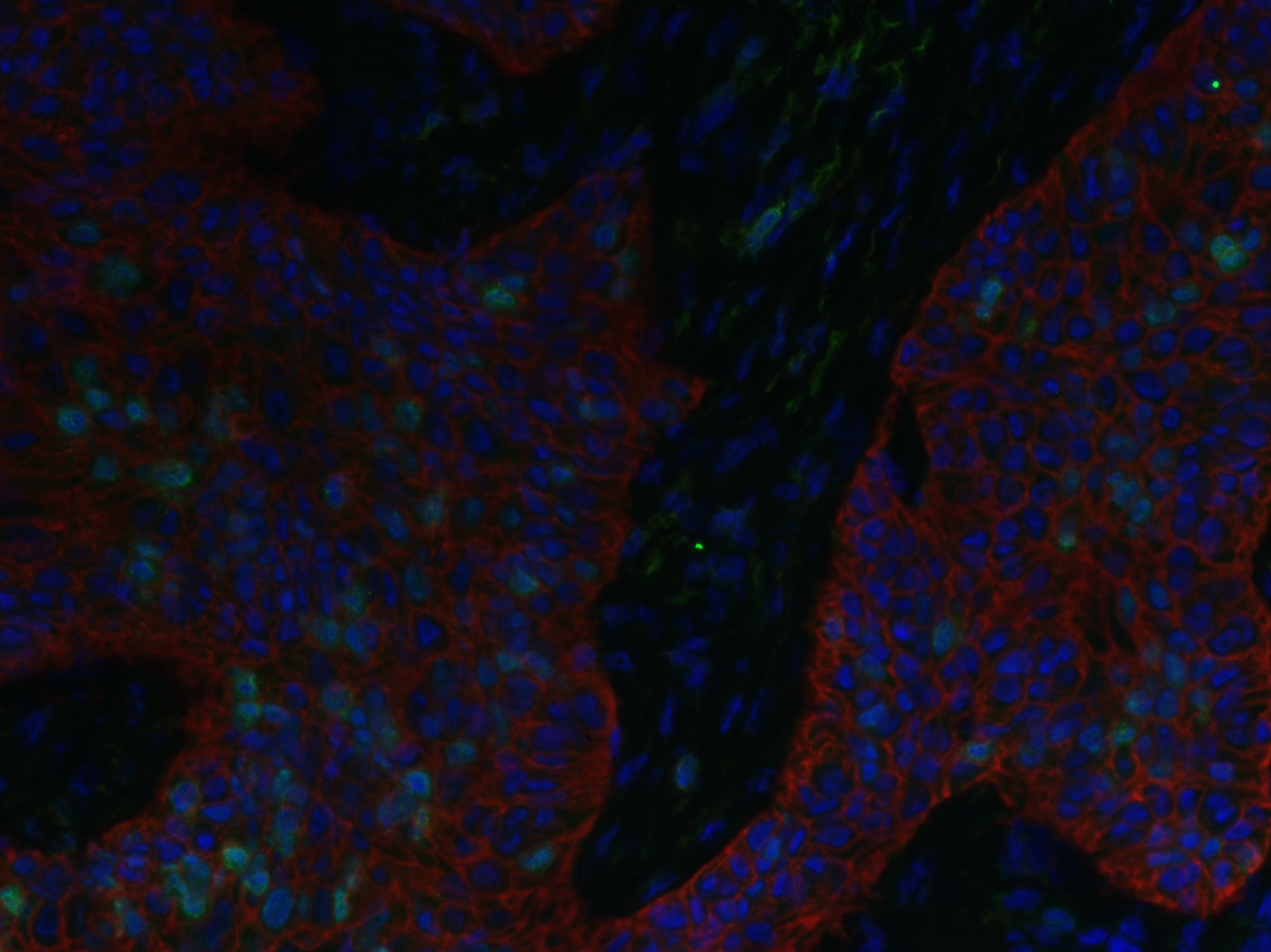

Supplement: Supplementary file 9 — Source Data for Figure 4 [file EMMM-15-e17761-s008.zip › Figure 4/4D/micro.image_EV.tiff]

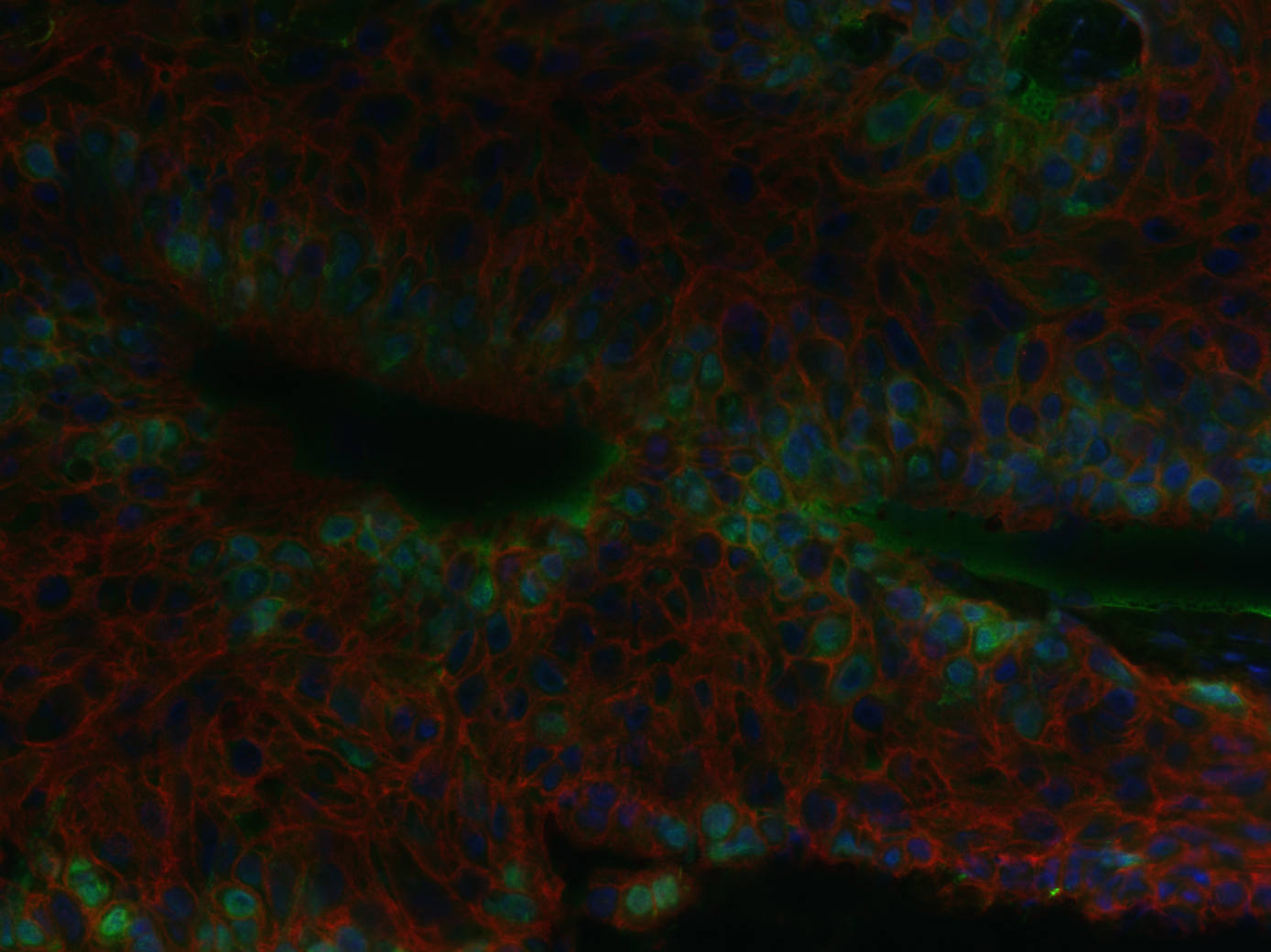

Supplement: Supplementary file 9 — Source Data for Figure 4 [file EMMM-15-e17761-s008.zip › Figure 4/4D/micro.image_KO.tiff]

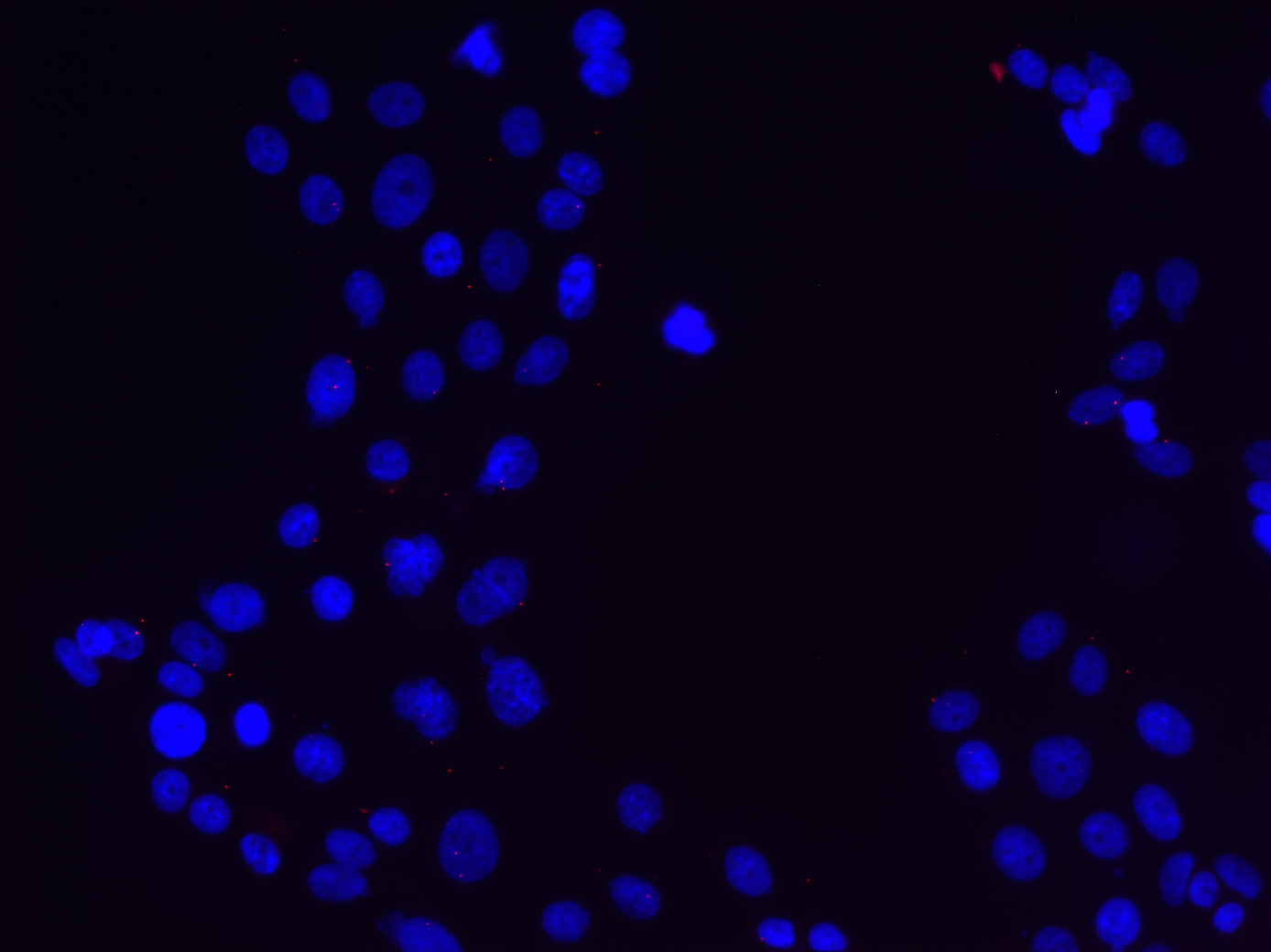

Supplement: Supplementary file 10 — Source Data for Figure 5 [file EMMM-15-e17761-s005.zip › Figure 5/5H/micro.image neg.ctrl bottom panel.tif]

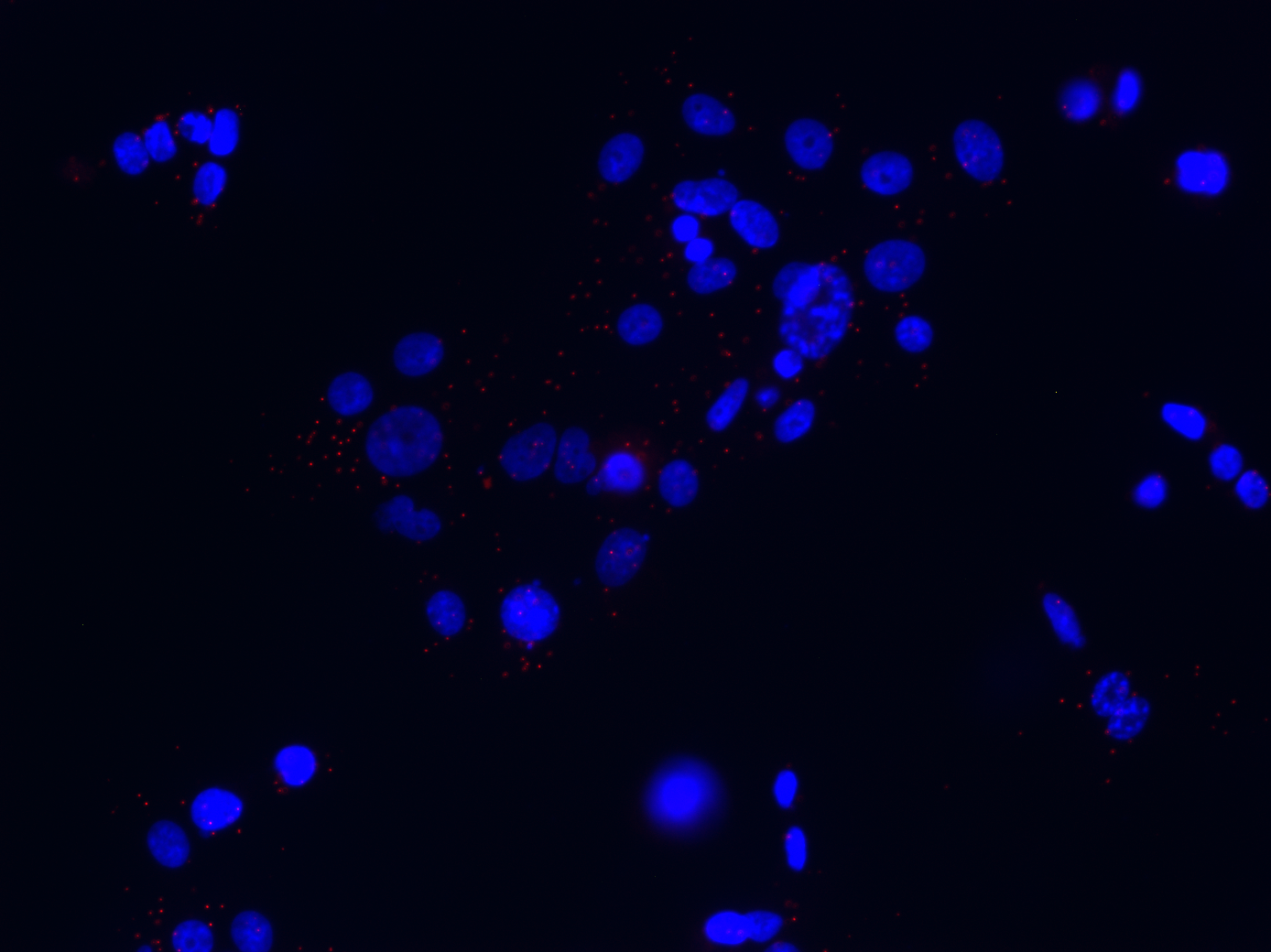

Supplement: Supplementary file 10 — Source Data for Figure 5 [file EMMM-15-e17761-s005.zip › Figure 5/5H/micro.image HSPA5 bottom panel.tif]

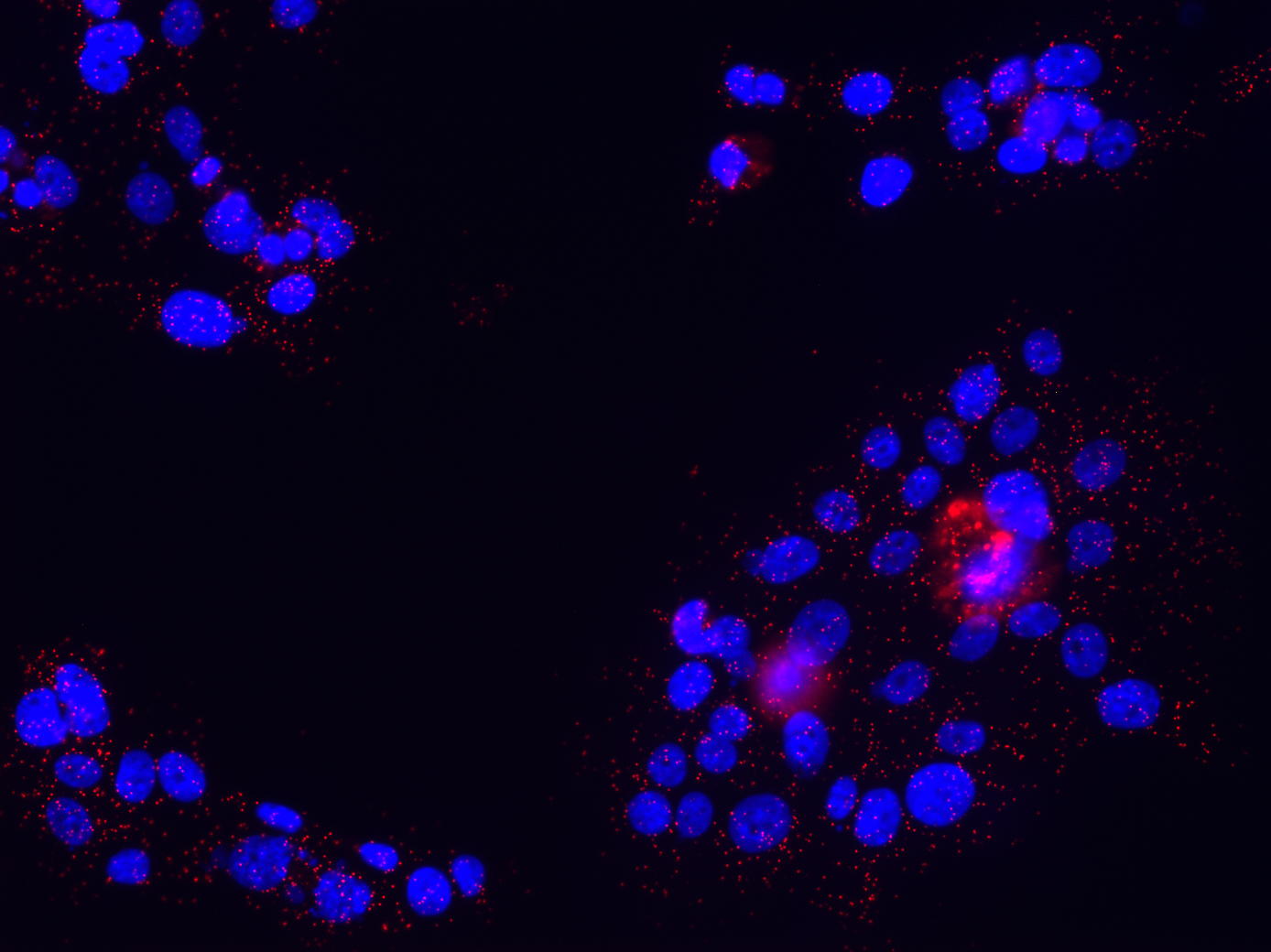

Supplement: Supplementary file 10 — Source Data for Figure 5 [file EMMM-15-e17761-s005.zip › Figure 5/5H/micro.image NRF3+HSPA5 bottom panel.tif]

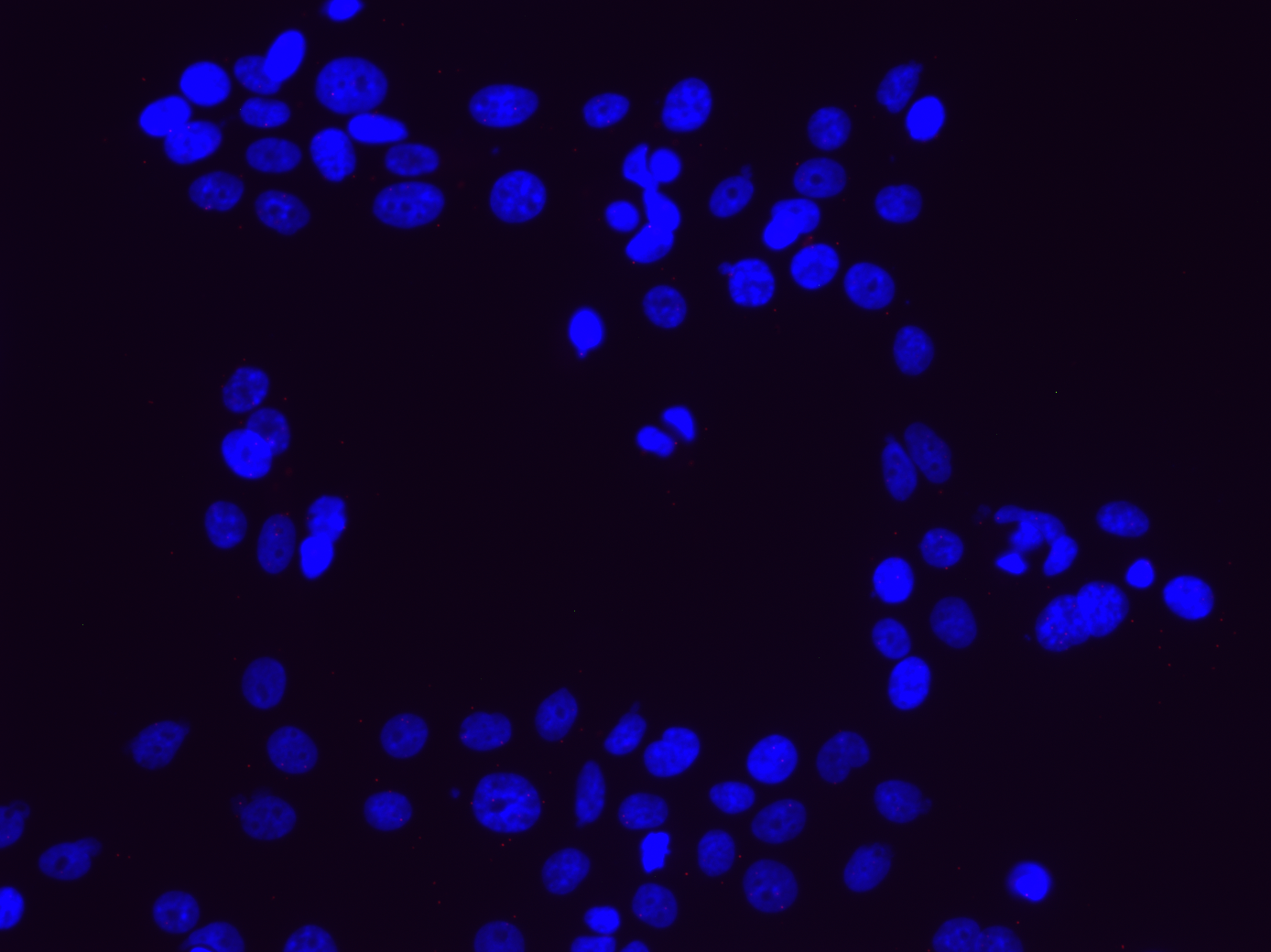

Supplement: Supplementary file 10 — Source Data for Figure 5 [file EMMM-15-e17761-s005.zip › Figure 5/5H/micro.image NRF3 bottom panel.tif]

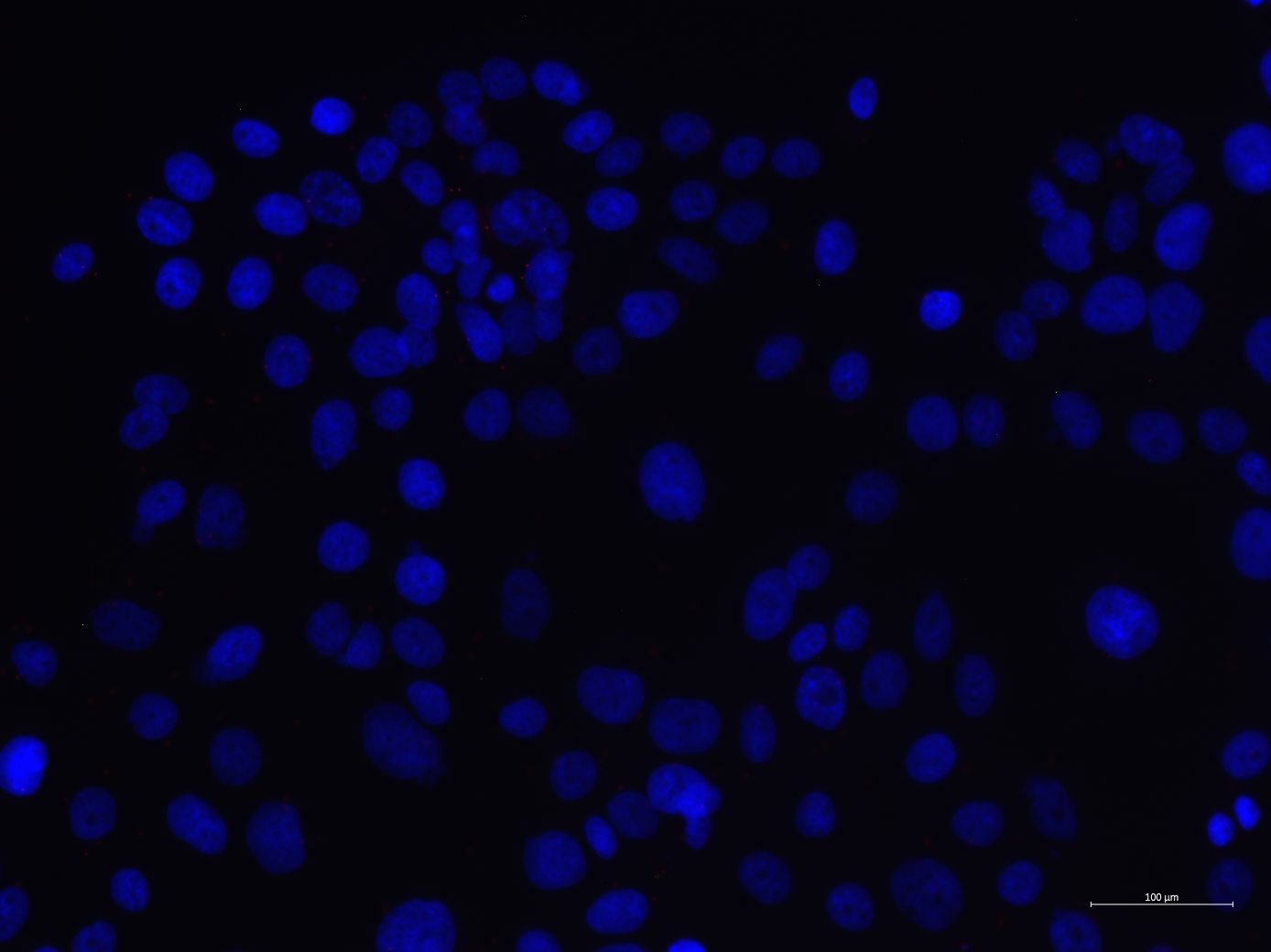

Supplement: Supplementary file 10 — Source Data for Figure 5 [file EMMM-15-e17761-s005.zip › Figure 5/5H/micro.image HA top panel.tif]

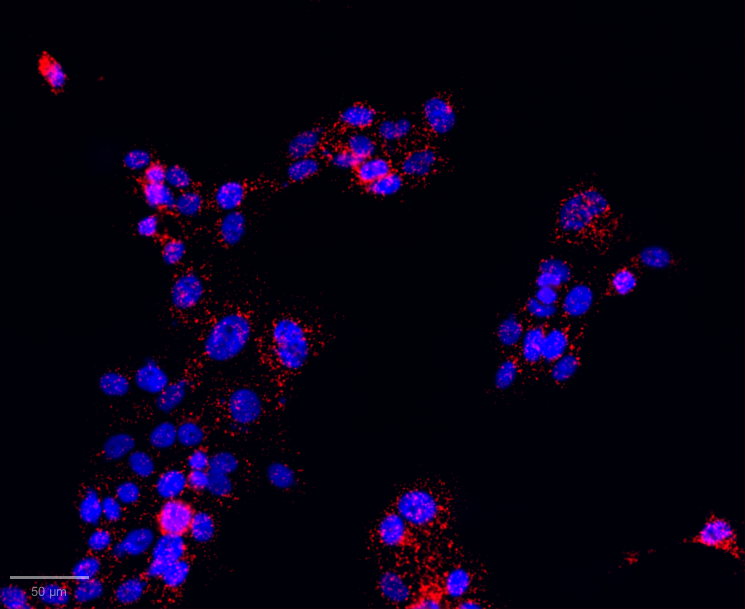

Supplement: Supplementary file 10 — Source Data for Figure 5 [file EMMM-15-e17761-s005.zip › Figure 5/5H/micro.image NRF3+PDIA3 middle panel.tif]

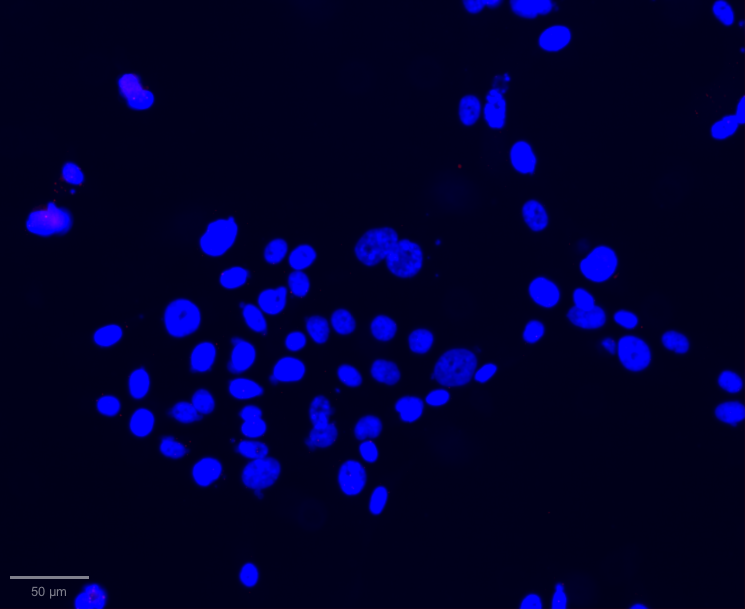

Supplement: Supplementary file 10 — Source Data for Figure 5 [file EMMM-15-e17761-s005.zip › Figure 5/5H/micro.image PDIA3 middle panel.tiff]

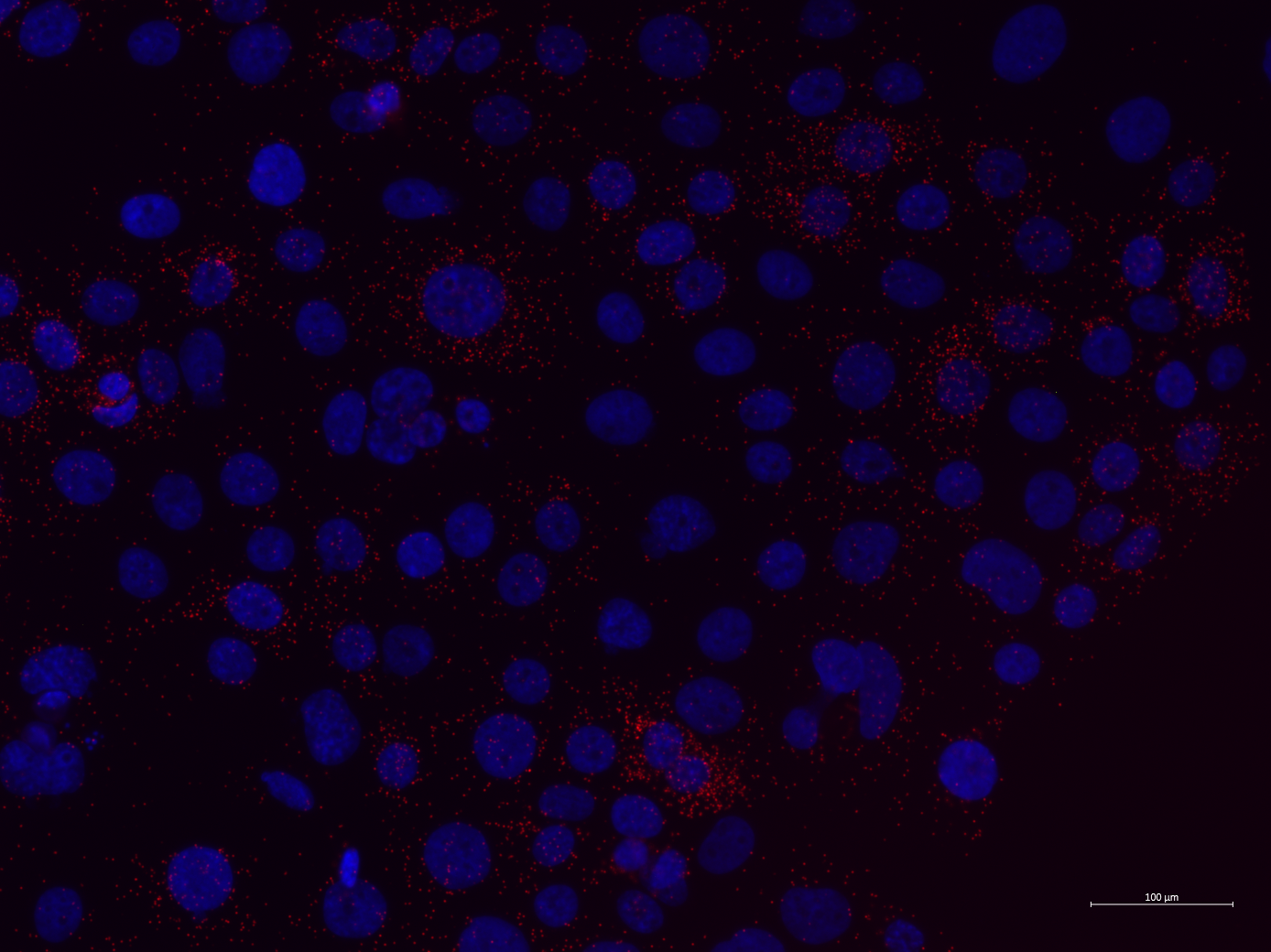

Supplement: Supplementary file 10 — Source Data for Figure 5 [file EMMM-15-e17761-s005.zip › Figure 5/5H/micro.image HA+PDIA3 top panel.tif]

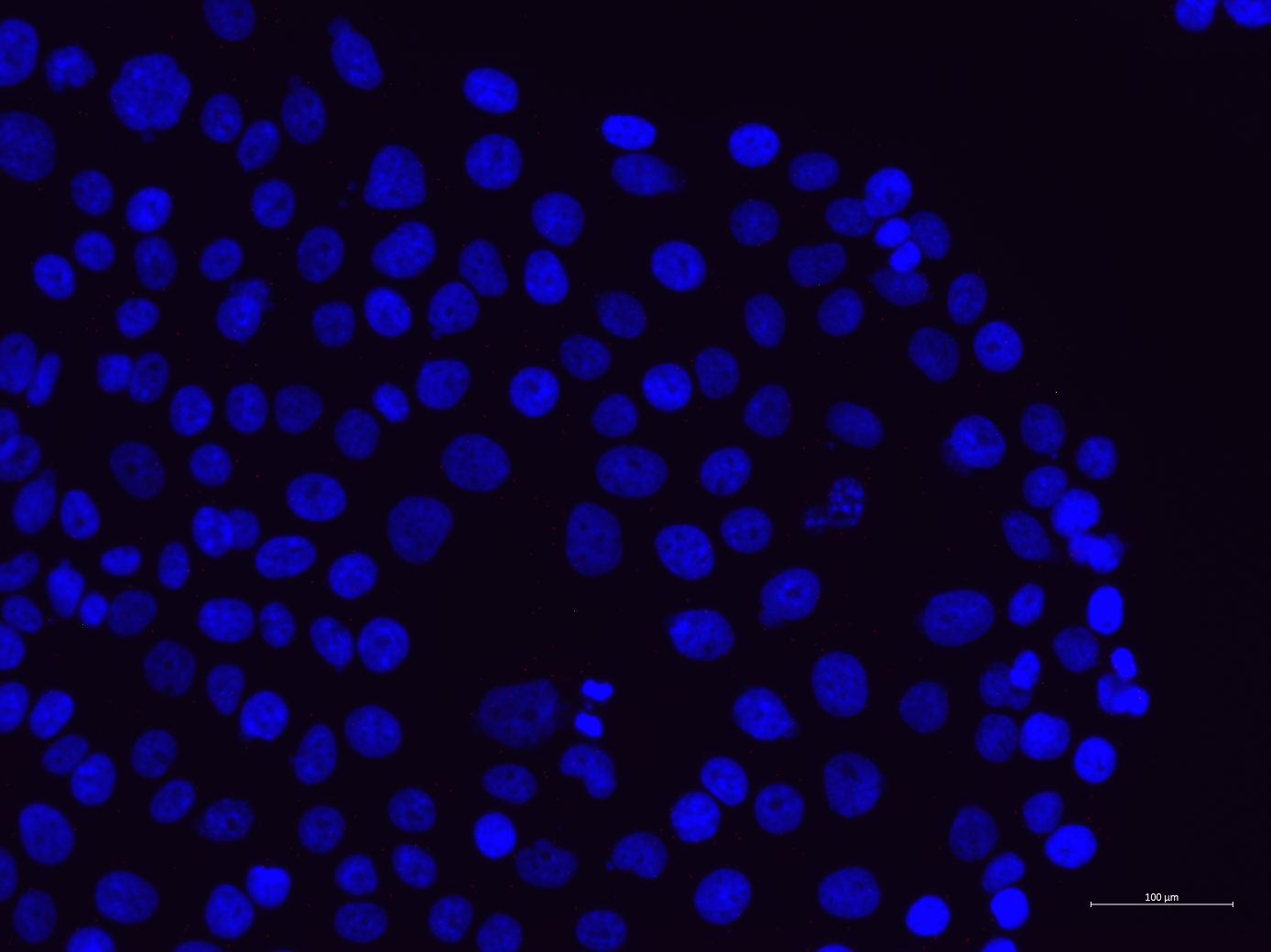

Supplement: Supplementary file 10 — Source Data for Figure 5 [file EMMM-15-e17761-s005.zip › Figure 5/5H/micro.image PDIA3 top panel.tif]

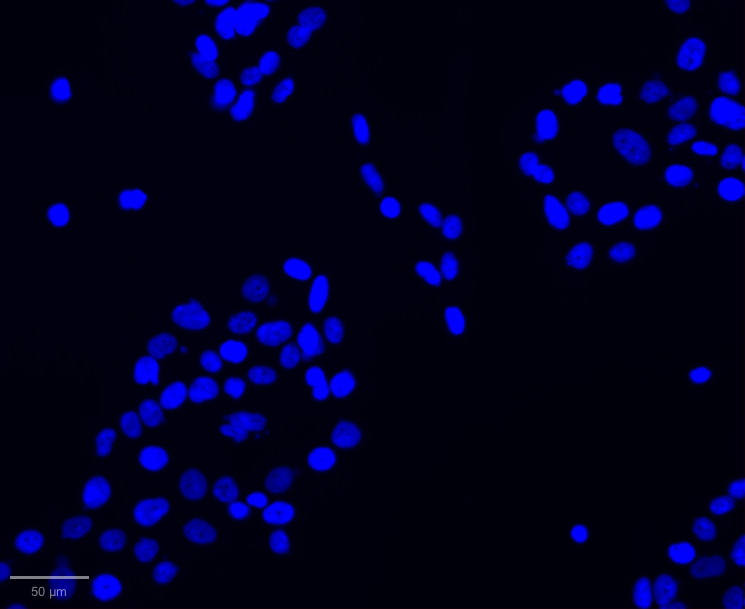

Supplement: Supplementary file 10 — Source Data for Figure 5 [file EMMM-15-e17761-s005.zip › Figure 5/5H/micro.image neg. ctrl middle panel.tif]

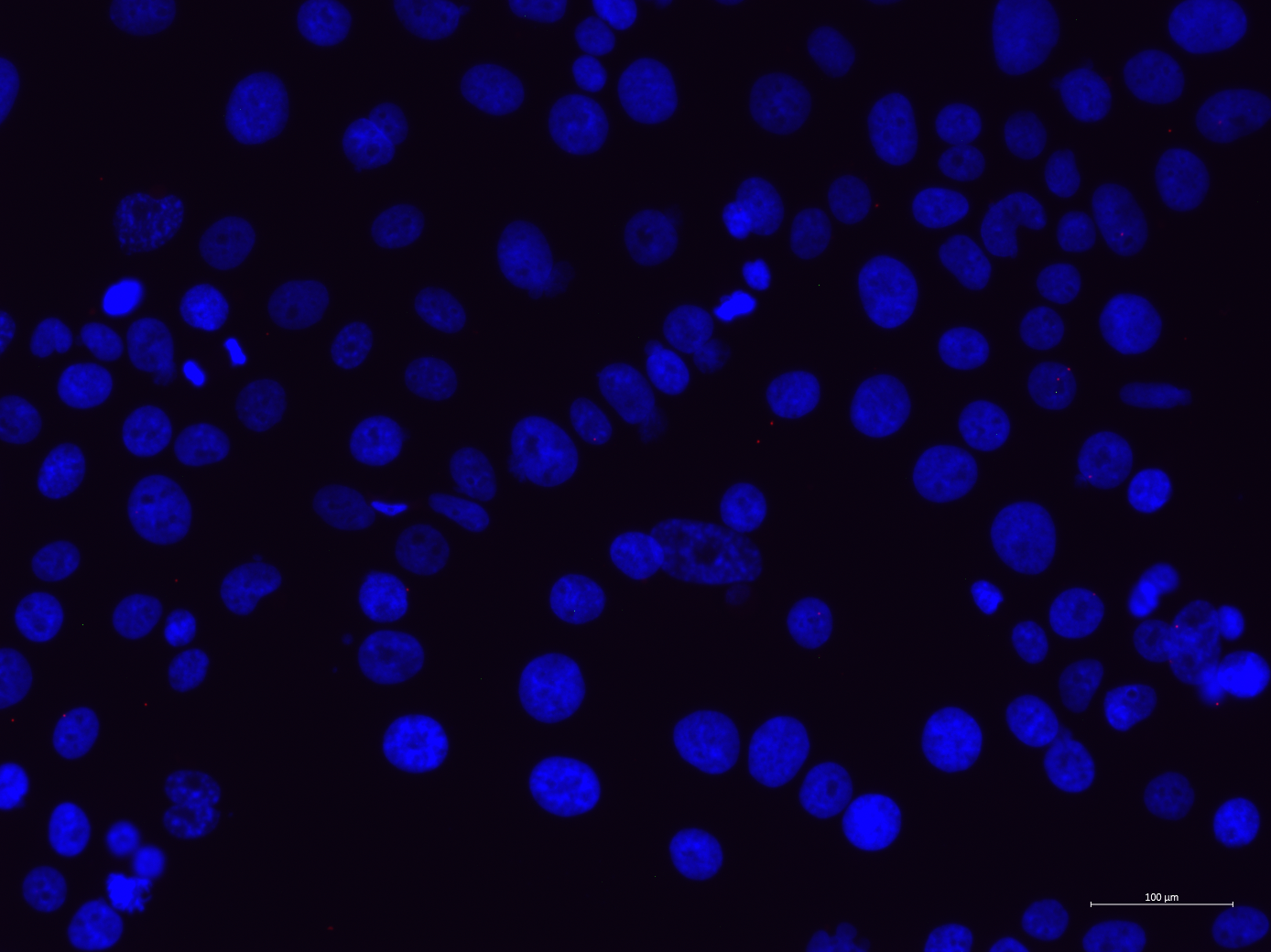

Supplement: Supplementary file 10 — Source Data for Figure 5 [file EMMM-15-e17761-s005.zip › Figure 5/5H/micro.image neg. ctrl top panel.tif]

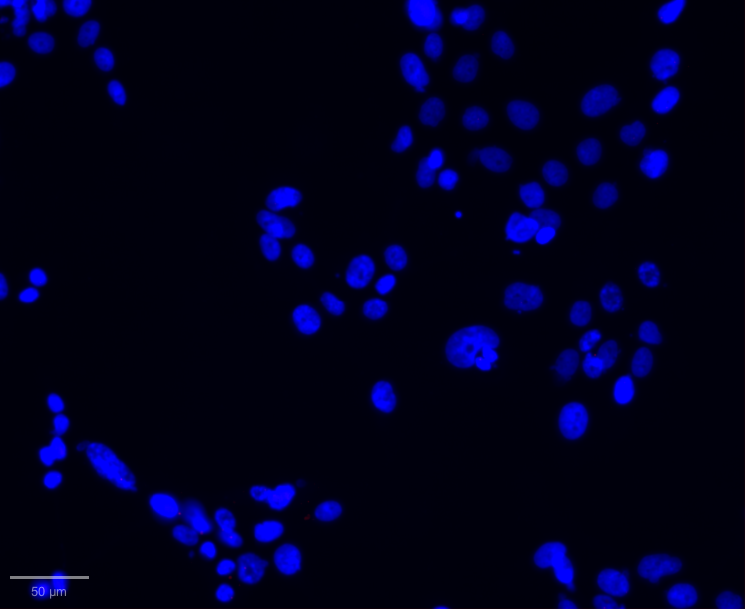

Supplement: Supplementary file 10 — Source Data for Figure 5 [file EMMM-15-e17761-s005.zip › Figure 5/5H/micro.image NRF3 middle panel.tif]

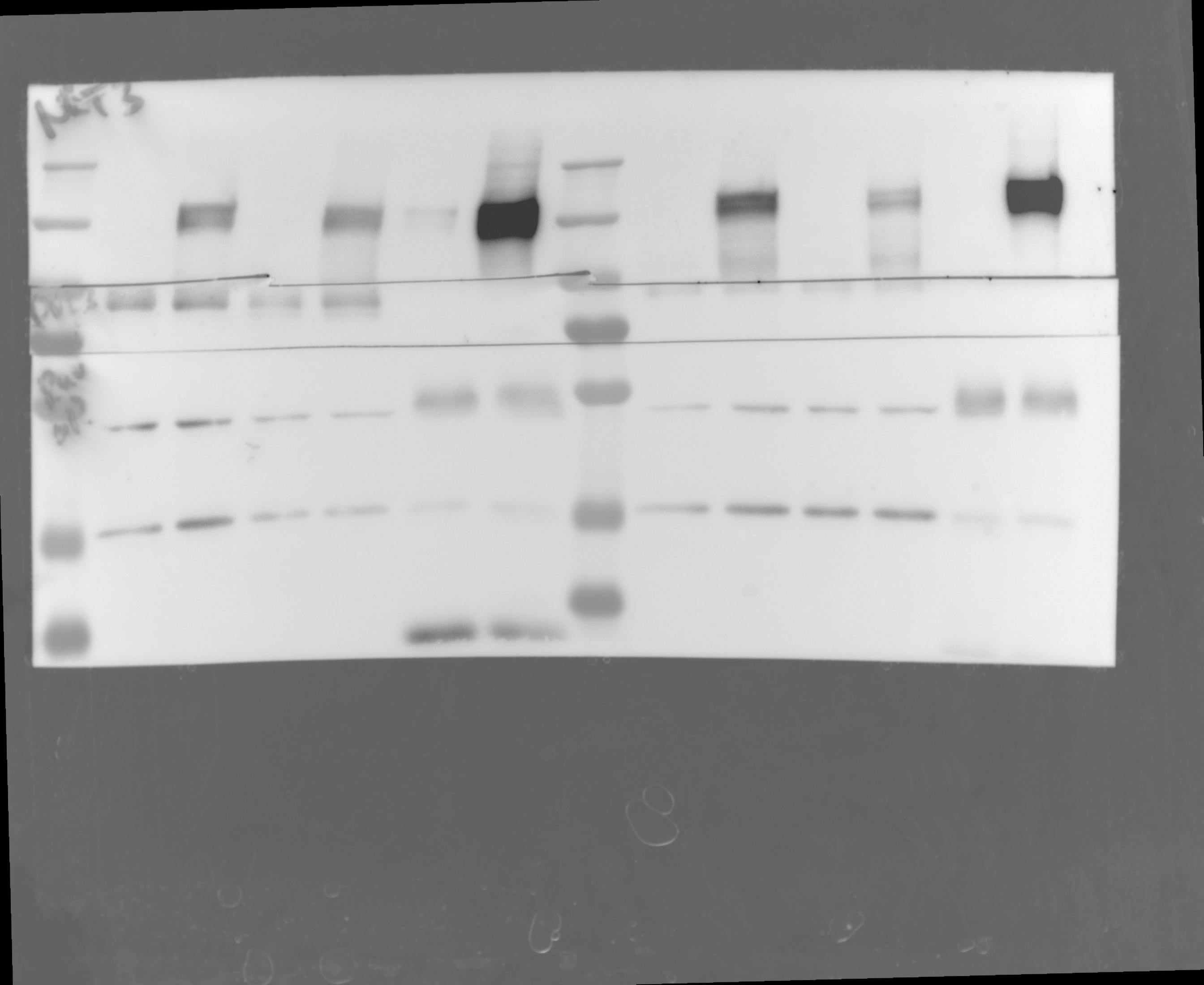

Supplement: Supplementary file 10 — Source Data for Figure 5 [file EMMM-15-e17761-s005.zip › Figure 5/5G/western NRF3 marker.Tif]

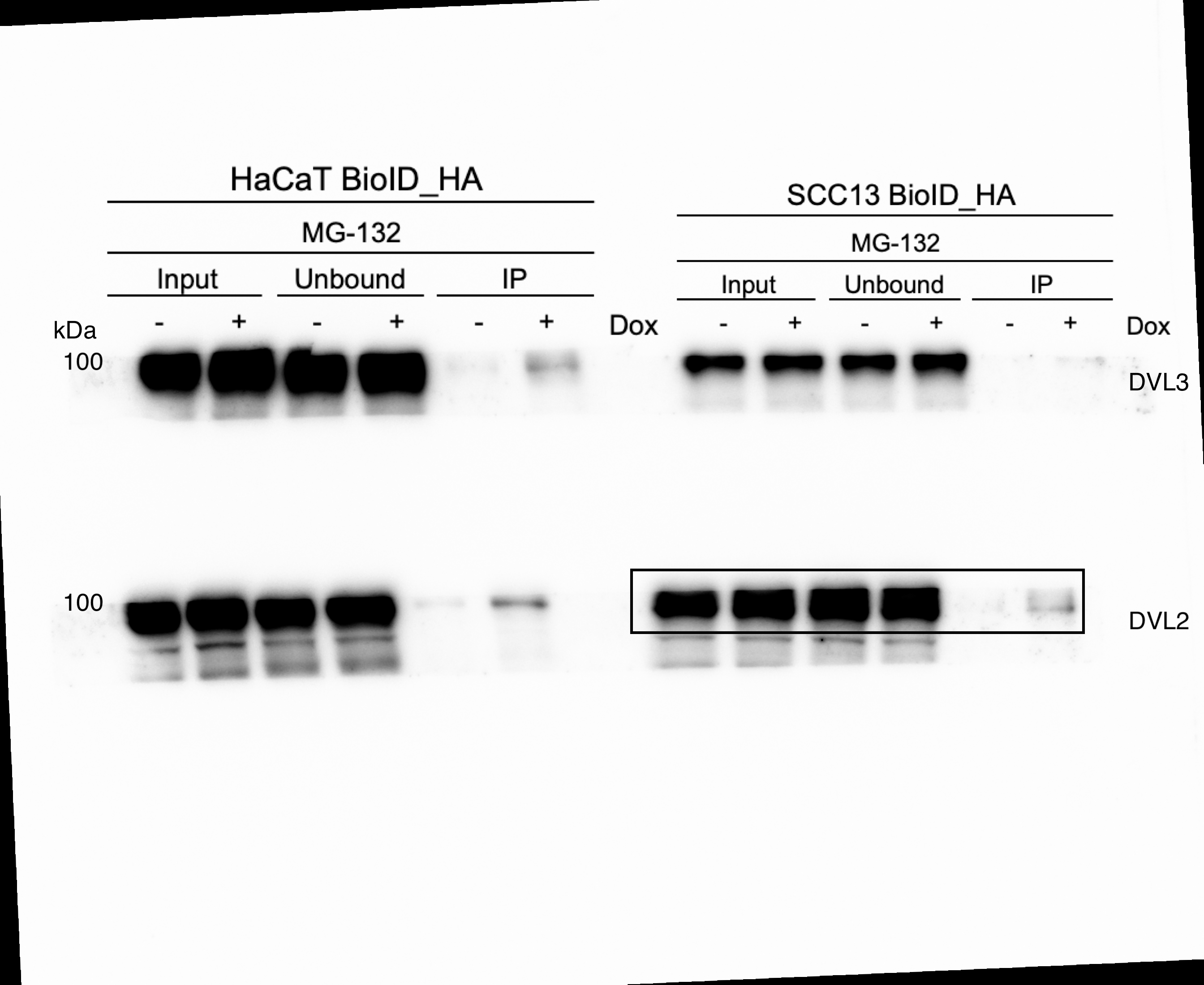

Supplement: Supplementary file 10 — Source Data for Figure 5 [file EMMM-15-e17761-s005.zip › Figure 5/5G/western DVL2 labelled.png]

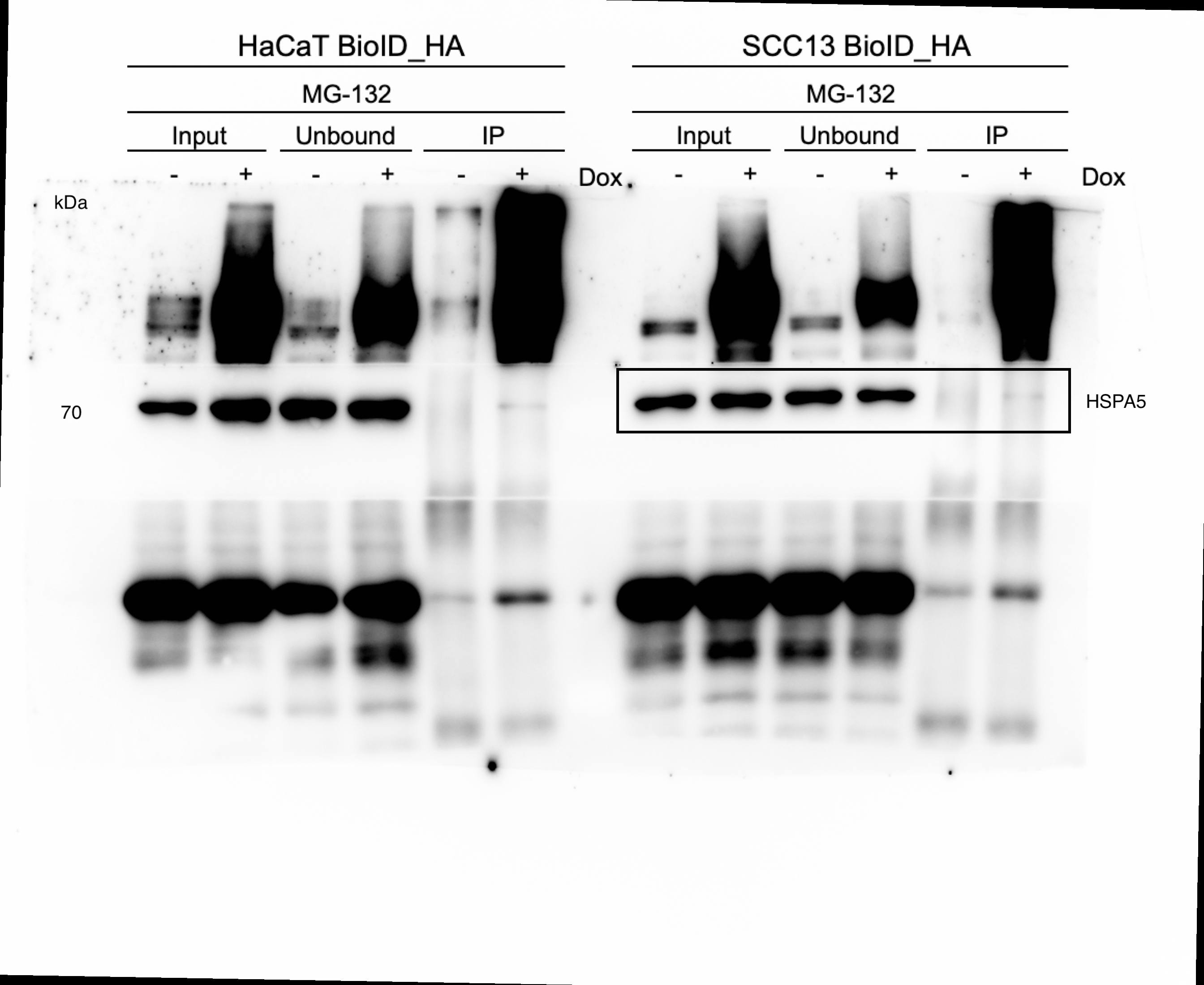

Supplement: Supplementary file 10 — Source Data for Figure 5 [file EMMM-15-e17761-s005.zip › Figure 5/5G/western HSPA5 labelled.png]

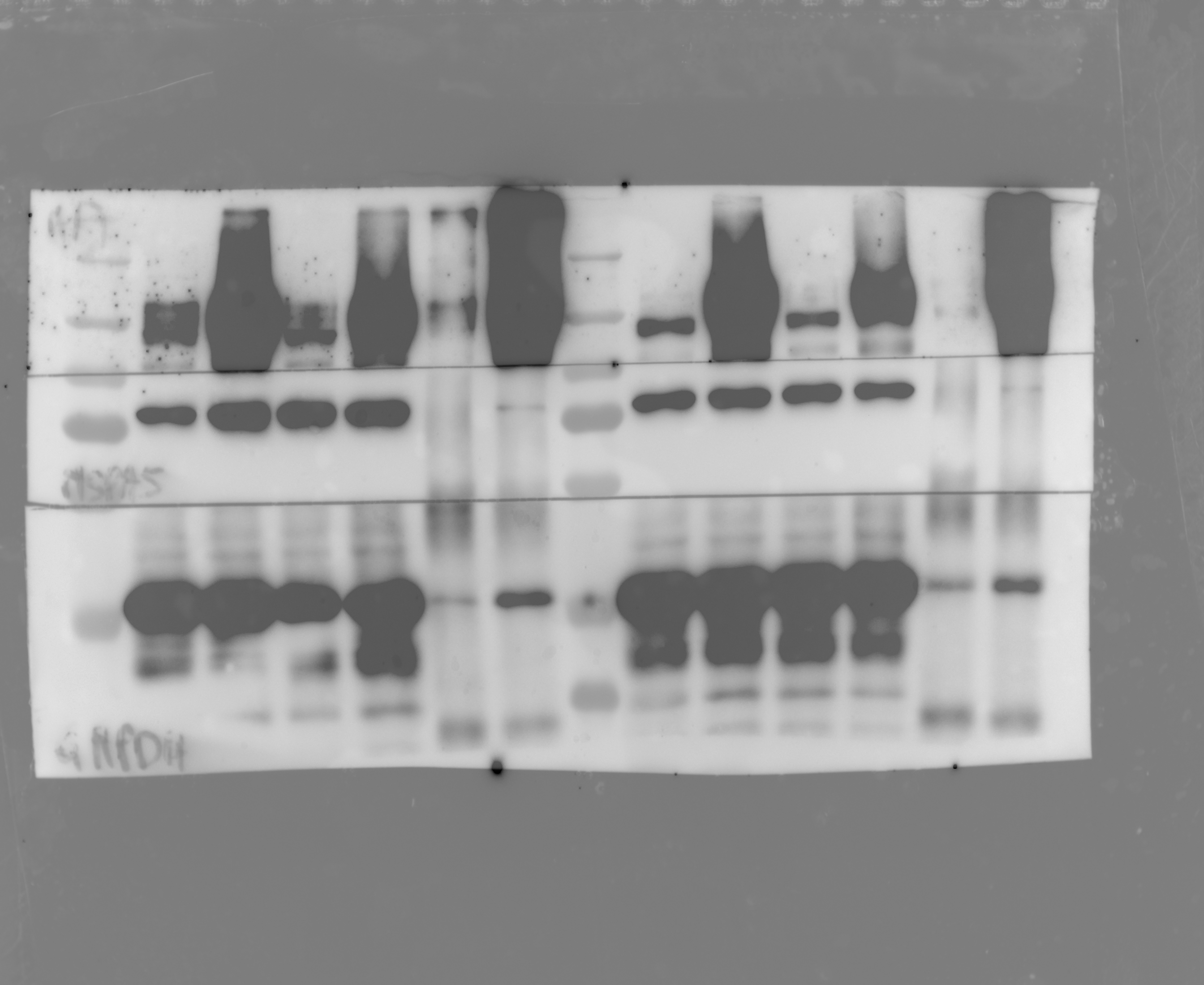

Supplement: Supplementary file 10 — Source Data for Figure 5 [file EMMM-15-e17761-s005.zip › Figure 5/5G/western HSPA5 marker.Tif]

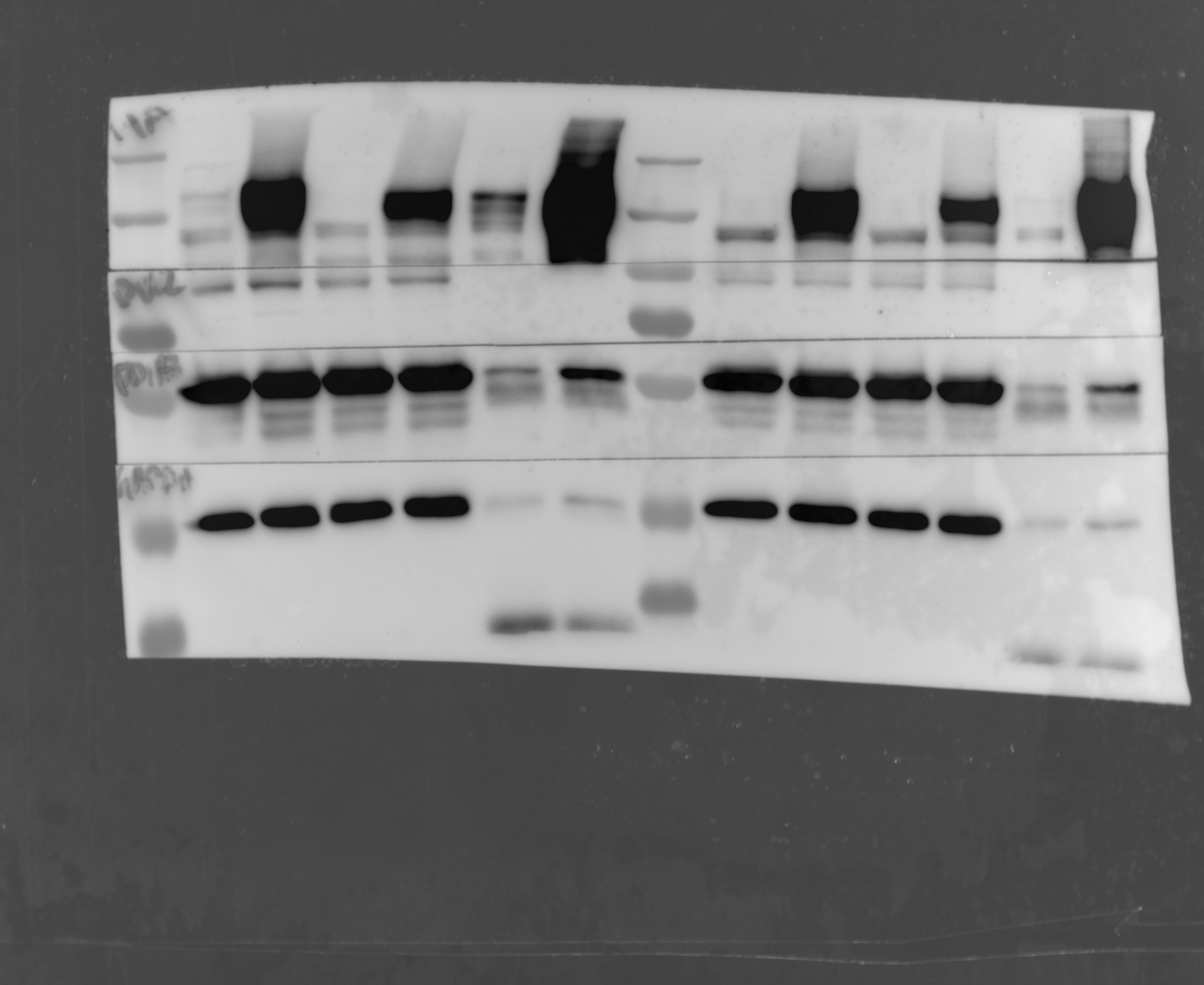

Supplement: Supplementary file 10 — Source Data for Figure 5 [file EMMM-15-e17761-s005.zip › Figure 5/5G/western HA DVL2 PDIA3 GAPDH marker.Tif]

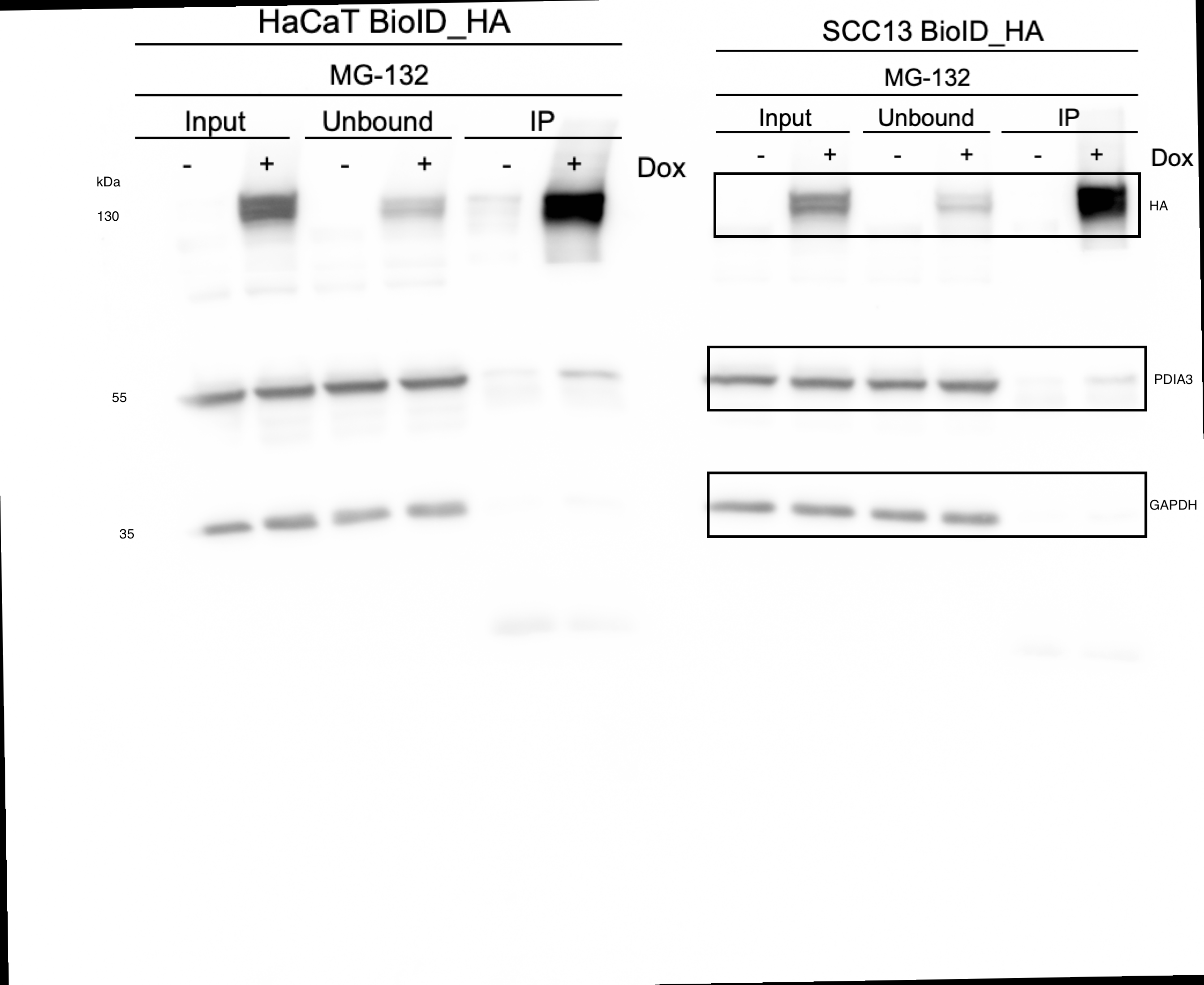

Supplement: Supplementary file 10 — Source Data for Figure 5 [file EMMM-15-e17761-s005.zip › Figure 5/5G/western HA PDIA3 GAPDH labelled.png]

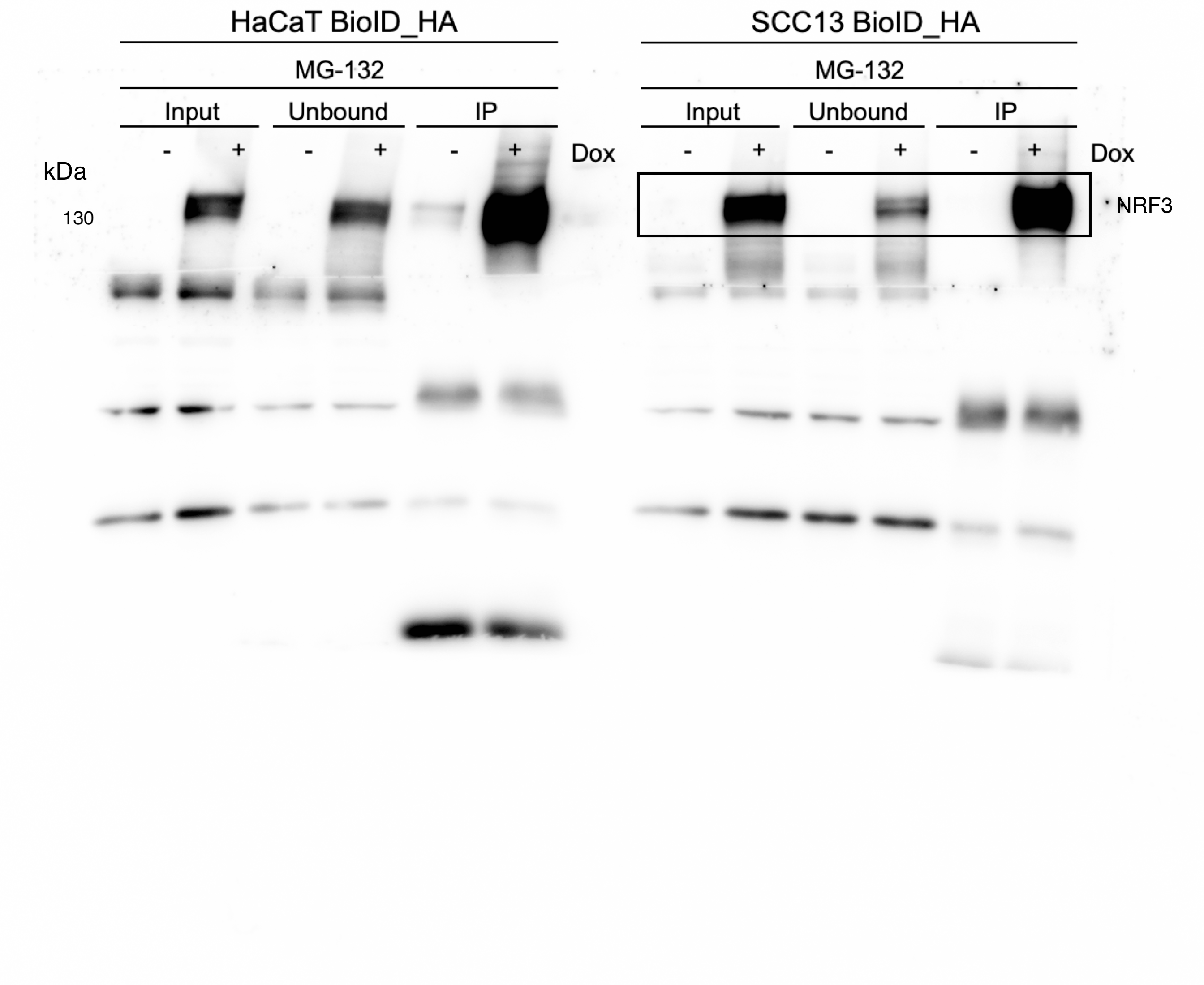

Supplement: Supplementary file 10 — Source Data for Figure 5 [file EMMM-15-e17761-s005.zip › Figure 5/5G/western NRF3 labelled.png]

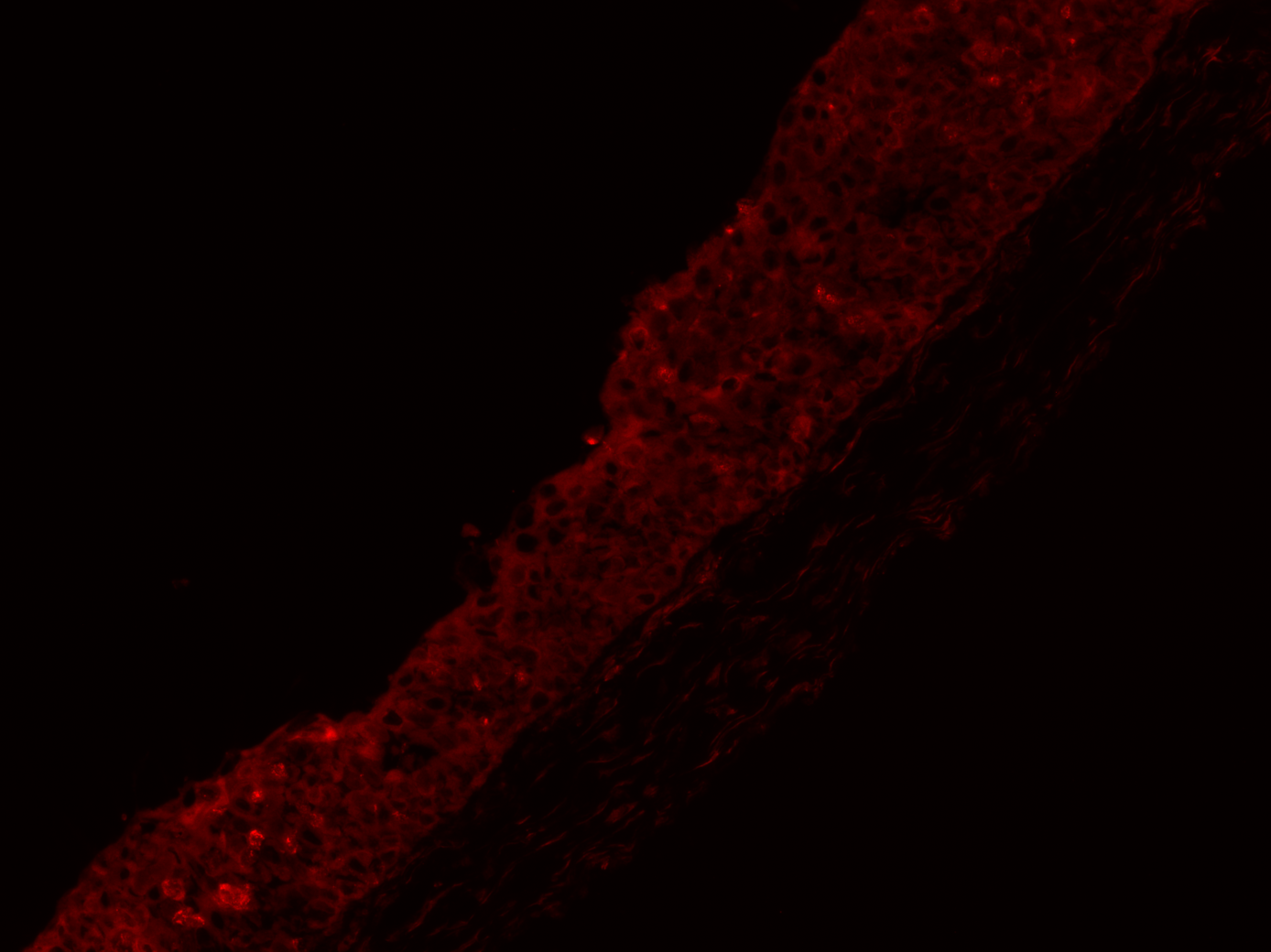

Supplement: Supplementary file 11 — Source Data for Figure 6 [file EMMM-15-e17761-s007.zip › Figure 6/6D/micro.image_EV C1 HSPA5.tif]

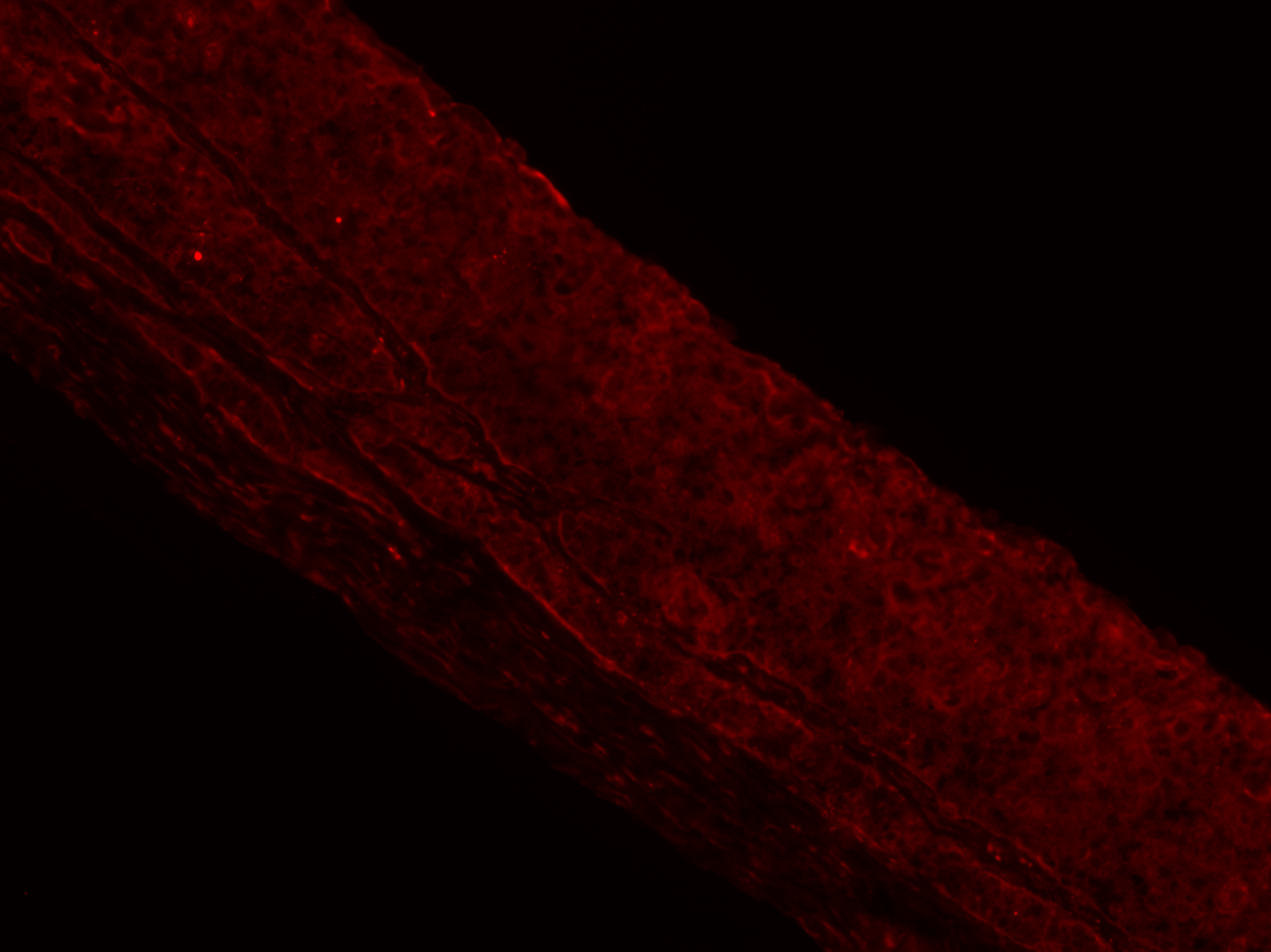

Supplement: Supplementary file 11 — Source Data for Figure 6 [file EMMM-15-e17761-s007.zip › Figure 6/6D/micro.image_KO C4 HSPA5.tif]

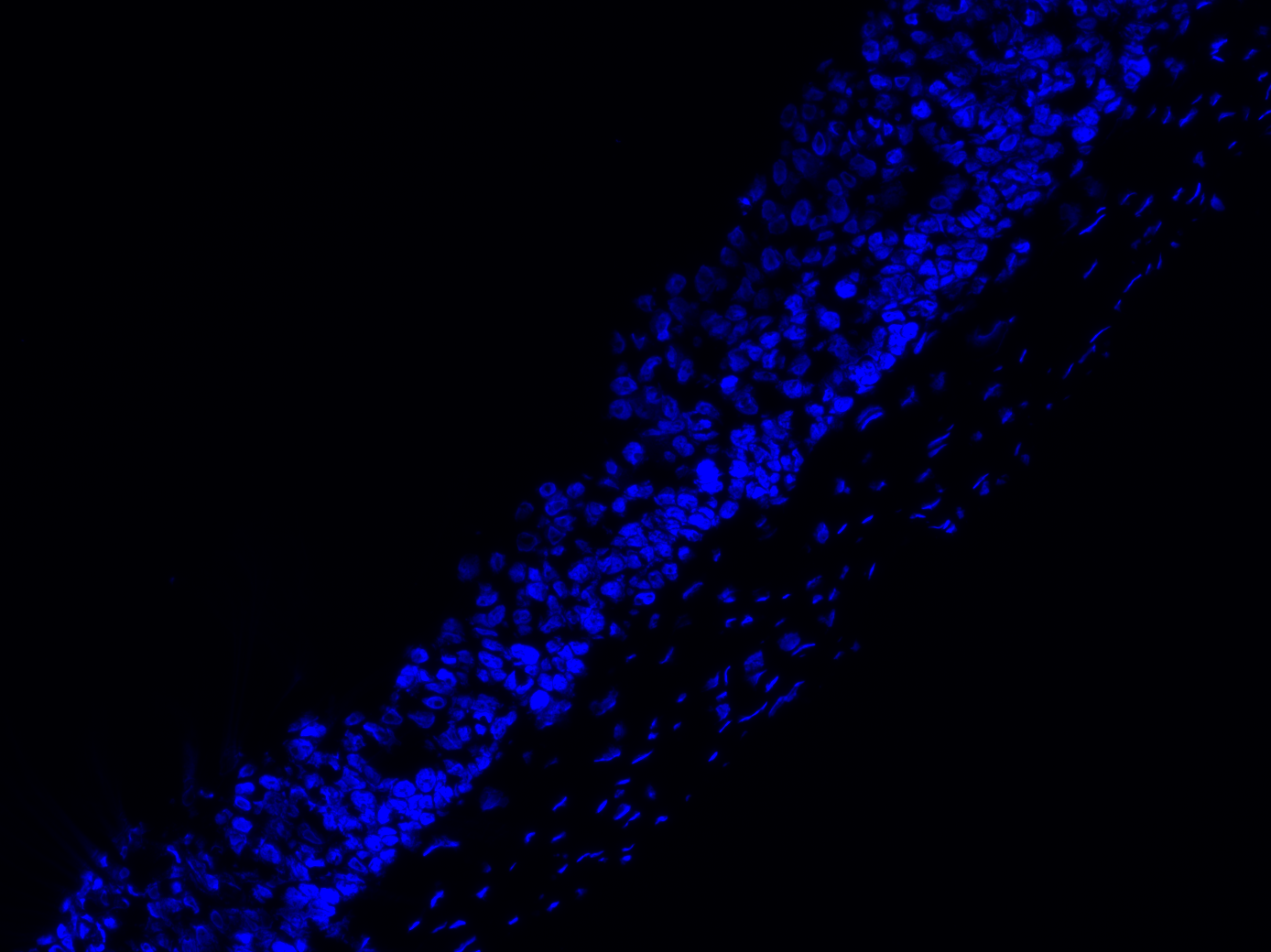

Supplement: Supplementary file 11 — Source Data for Figure 6 [file EMMM-15-e17761-s007.zip › Figure 6/6D/micro.image_EV C1 DAPI.tif]

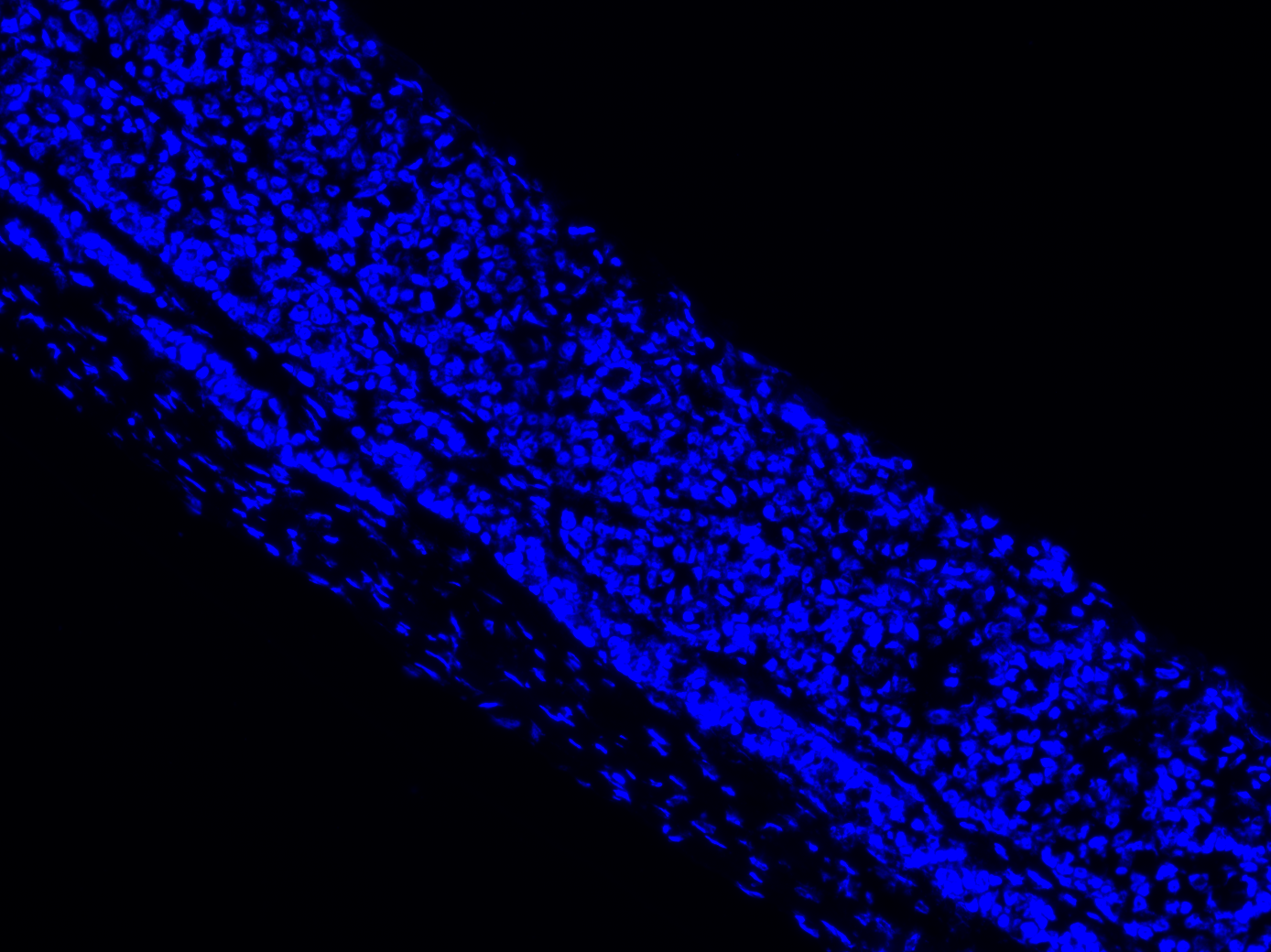

Supplement: Supplementary file 11 — Source Data for Figure 6 [file EMMM-15-e17761-s007.zip › Figure 6/6D/micro.image_KO C4 DAPI.tif]

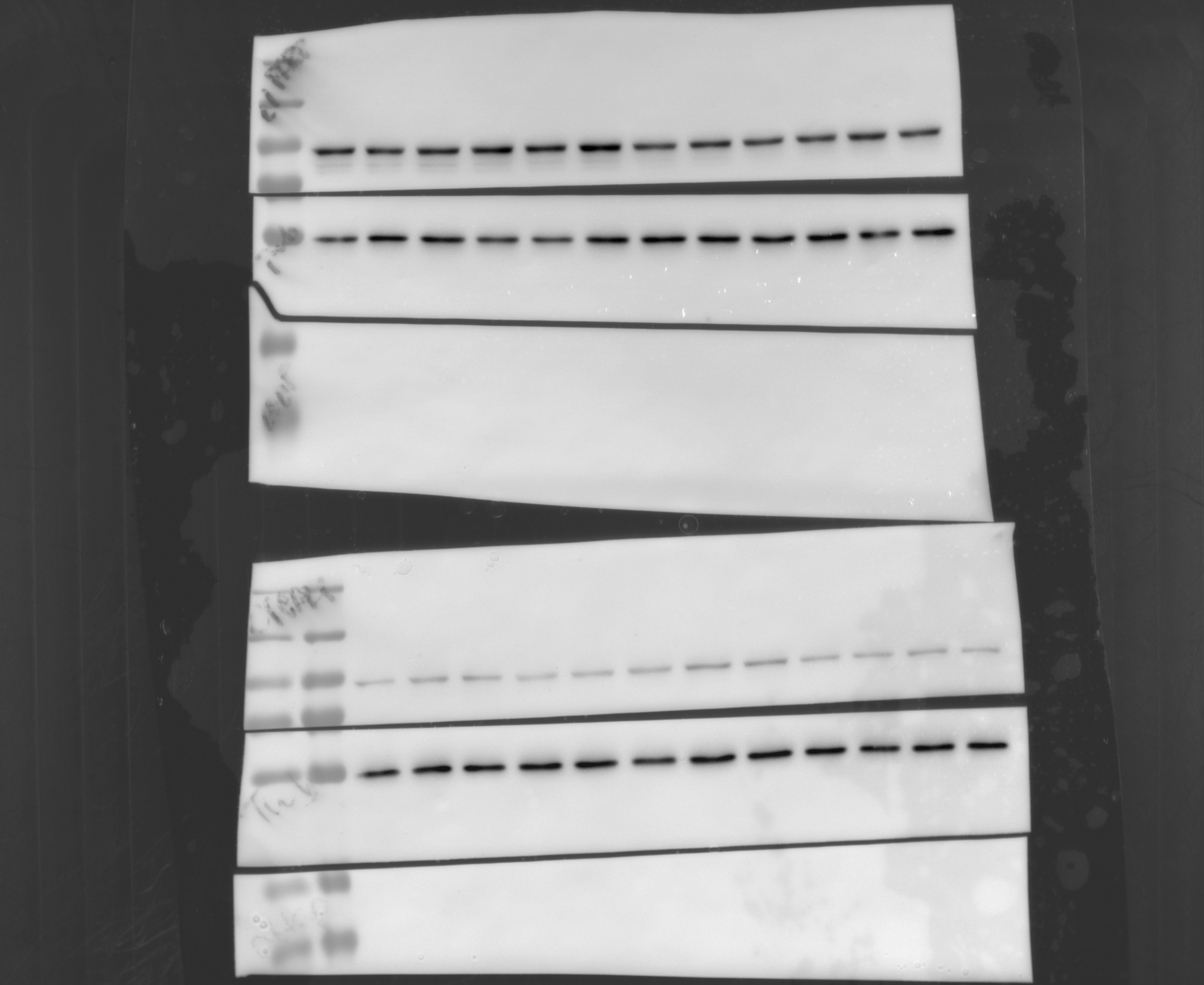

Supplement: Supplementary file 11 — Source Data for Figure 6 [file EMMM-15-e17761-s007.zip › Figure 6/6C/western tubulin marker.Tif]

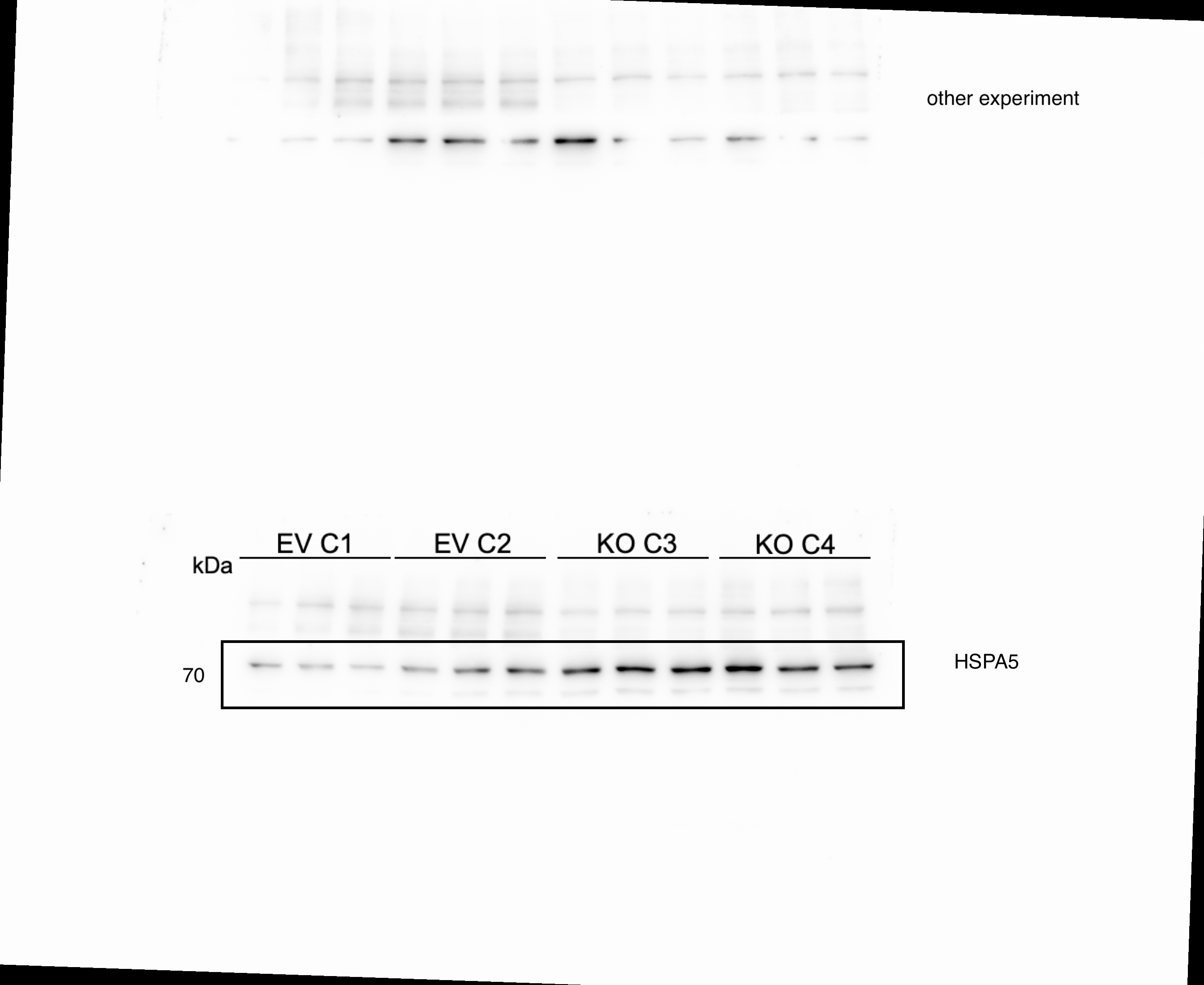

Supplement: Supplementary file 11 — Source Data for Figure 6 [file EMMM-15-e17761-s007.zip › Figure 6/6C/western HSPA5 labelled.tiff]

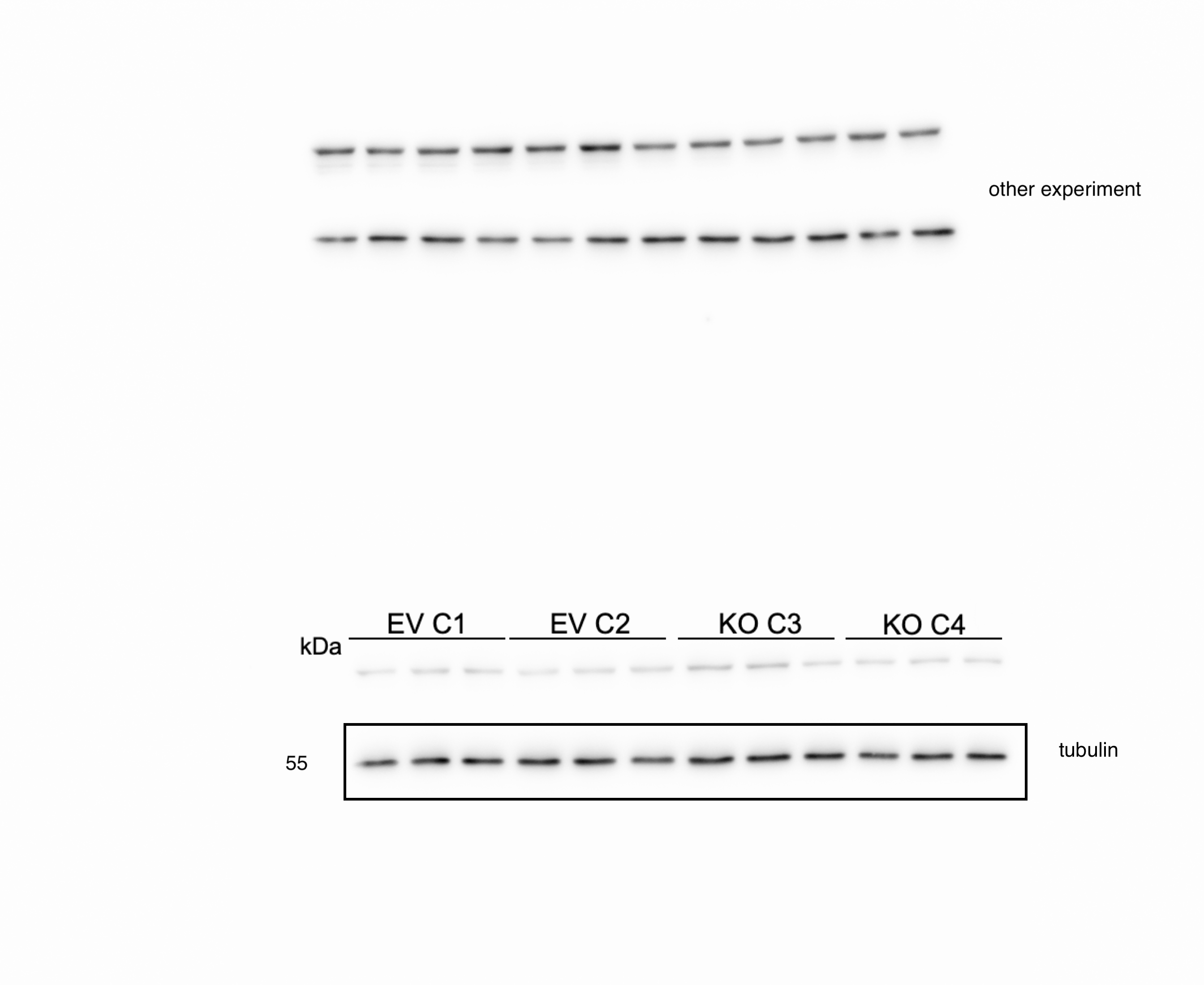

Supplement: Supplementary file 11 — Source Data for Figure 6 [file EMMM-15-e17761-s007.zip › Figure 6/6C/western tubulin labelled.tiff]

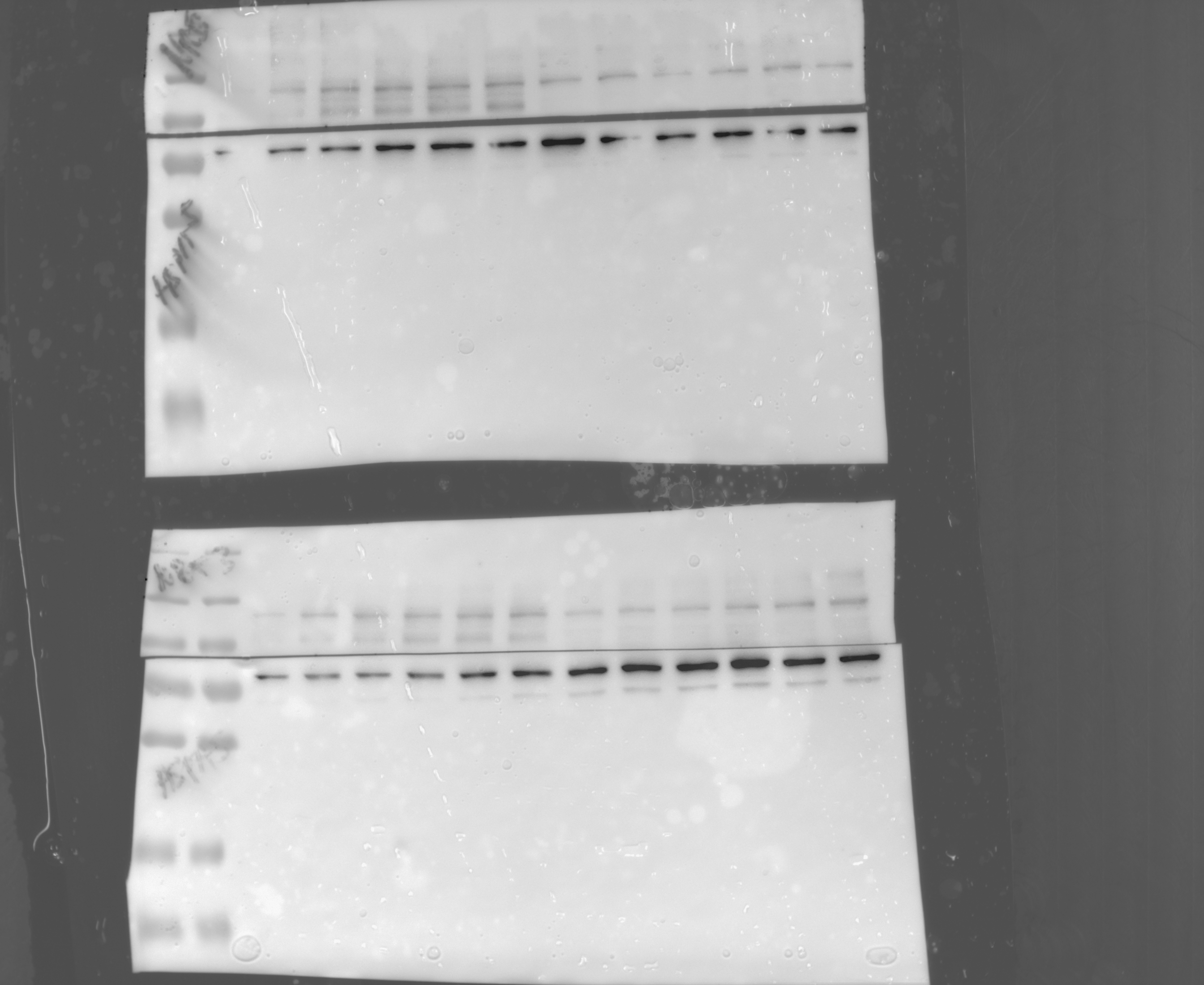

Supplement: Supplementary file 11 — Source Data for Figure 6 [file EMMM-15-e17761-s007.zip › Figure 6/6C/western HSPA5 marker.Tif]

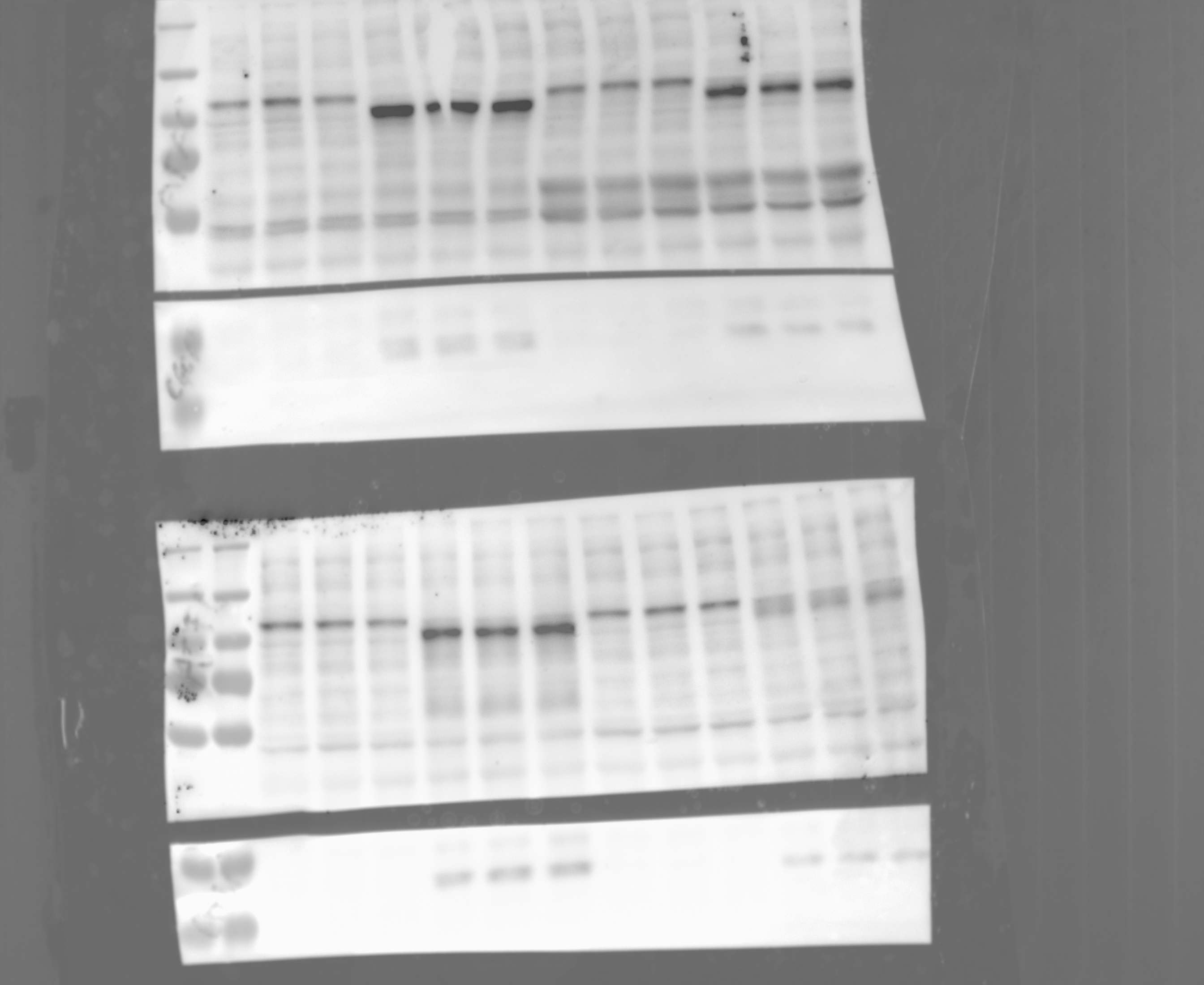

Supplement: Supplementary file 11 — Source Data for Figure 6 [file EMMM-15-e17761-s007.zip › Figure 6/6B/western CHOP EV C1 KO C3 marker.Tif]

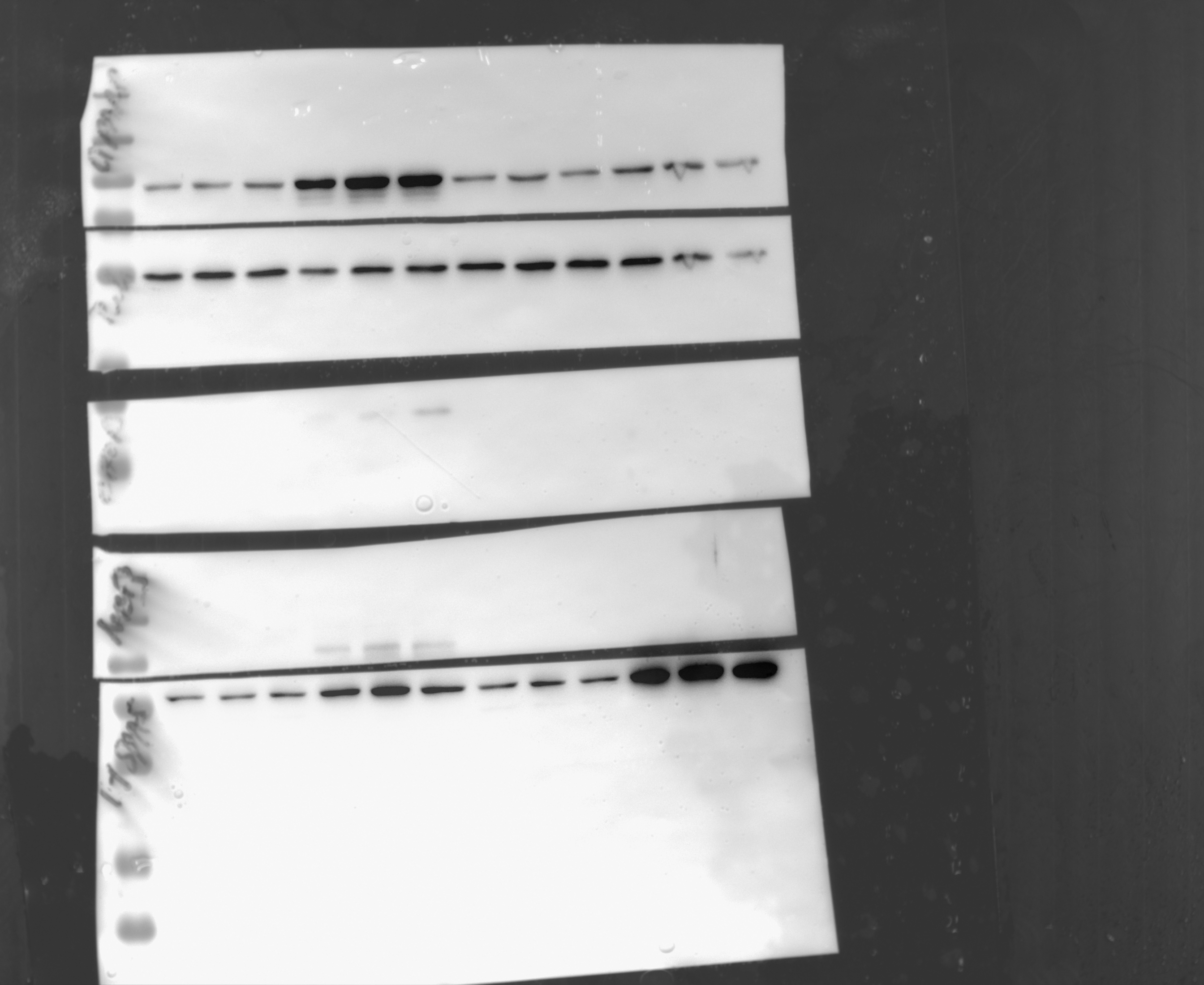

Supplement: Supplementary file 11 — Source Data for Figure 6 [file EMMM-15-e17761-s007.zip › Figure 6/6B/western clPARP Tubulin HSPA5 EV C2 KO C4 marker.Tif]

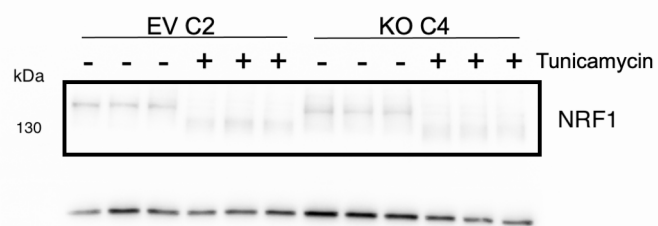

Supplement: Supplementary file 11 — Source Data for Figure 6 [file EMMM-15-e17761-s007.zip › Figure 6/6B/western NRF1 EV C2 KO C4 labelled.pdf]

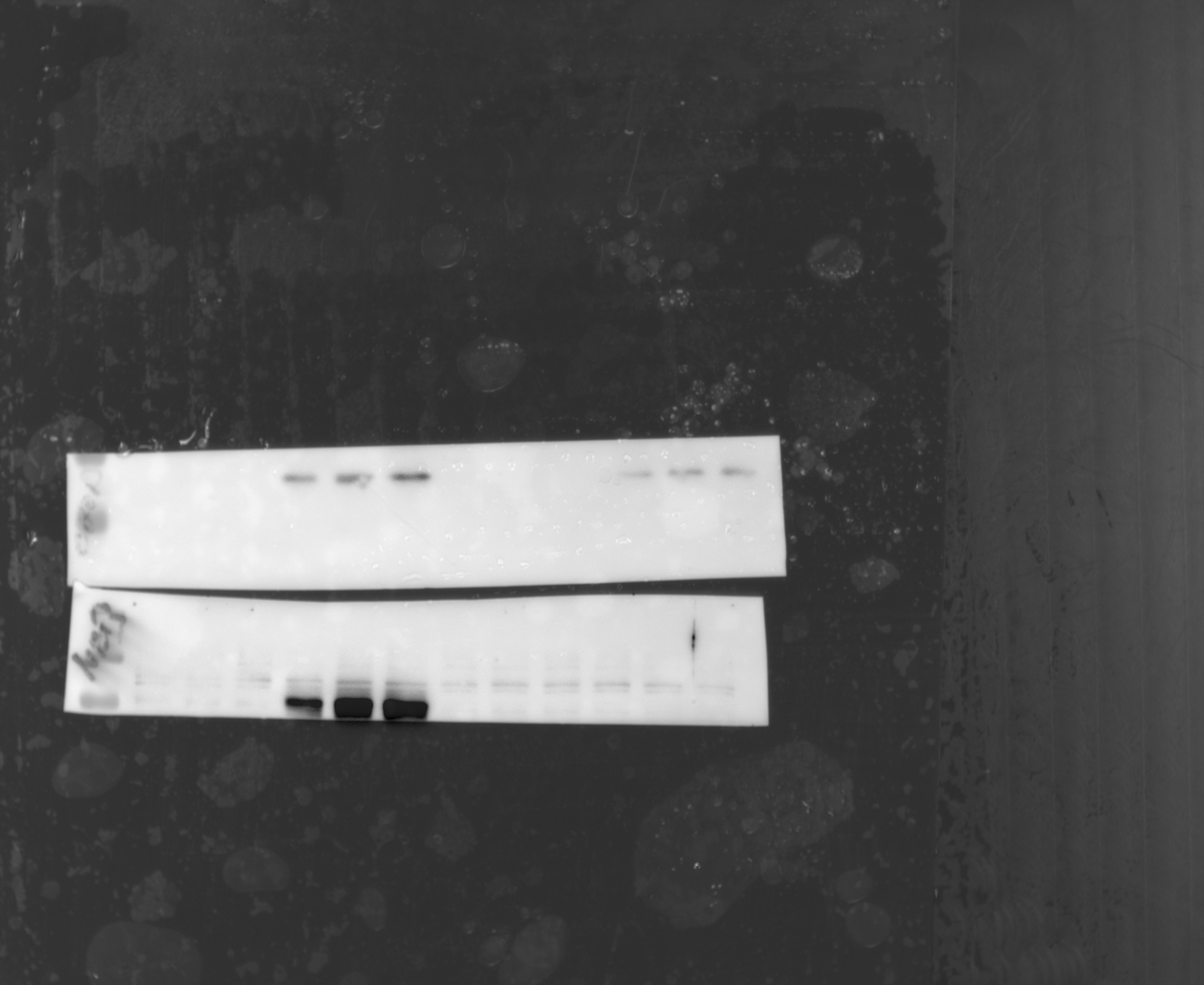

Supplement: Supplementary file 11 — Source Data for Figure 6 [file EMMM-15-e17761-s007.zip › Figure 6/6B/western CHOP NRF3 EV C2 KO C4 marker.Tif]

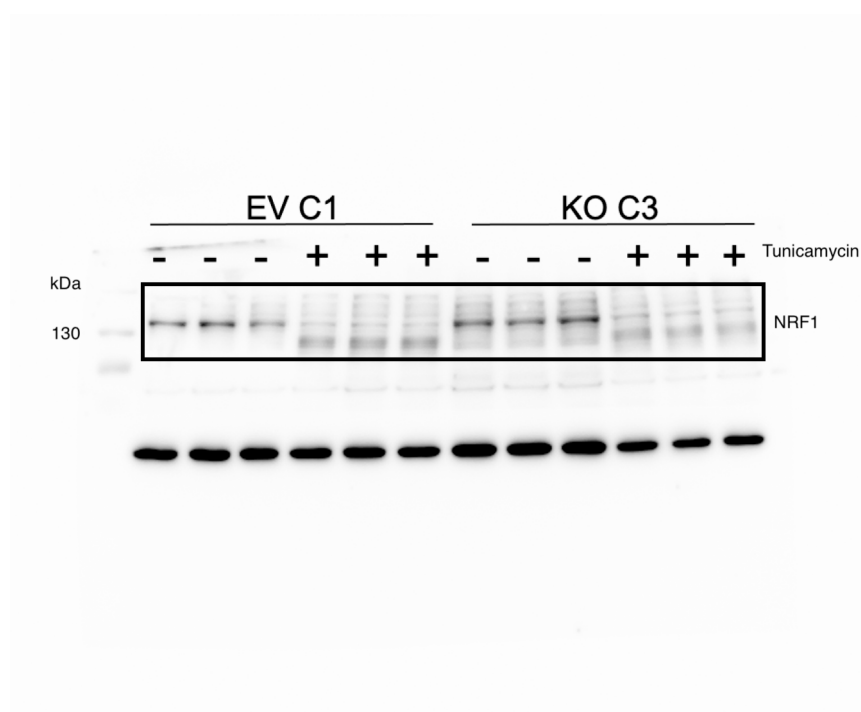

Supplement: Supplementary file 11 — Source Data for Figure 6 [file EMMM-15-e17761-s007.zip › Figure 6/6B/western NRF1 EV C1 KO C3 labelled.pdf]

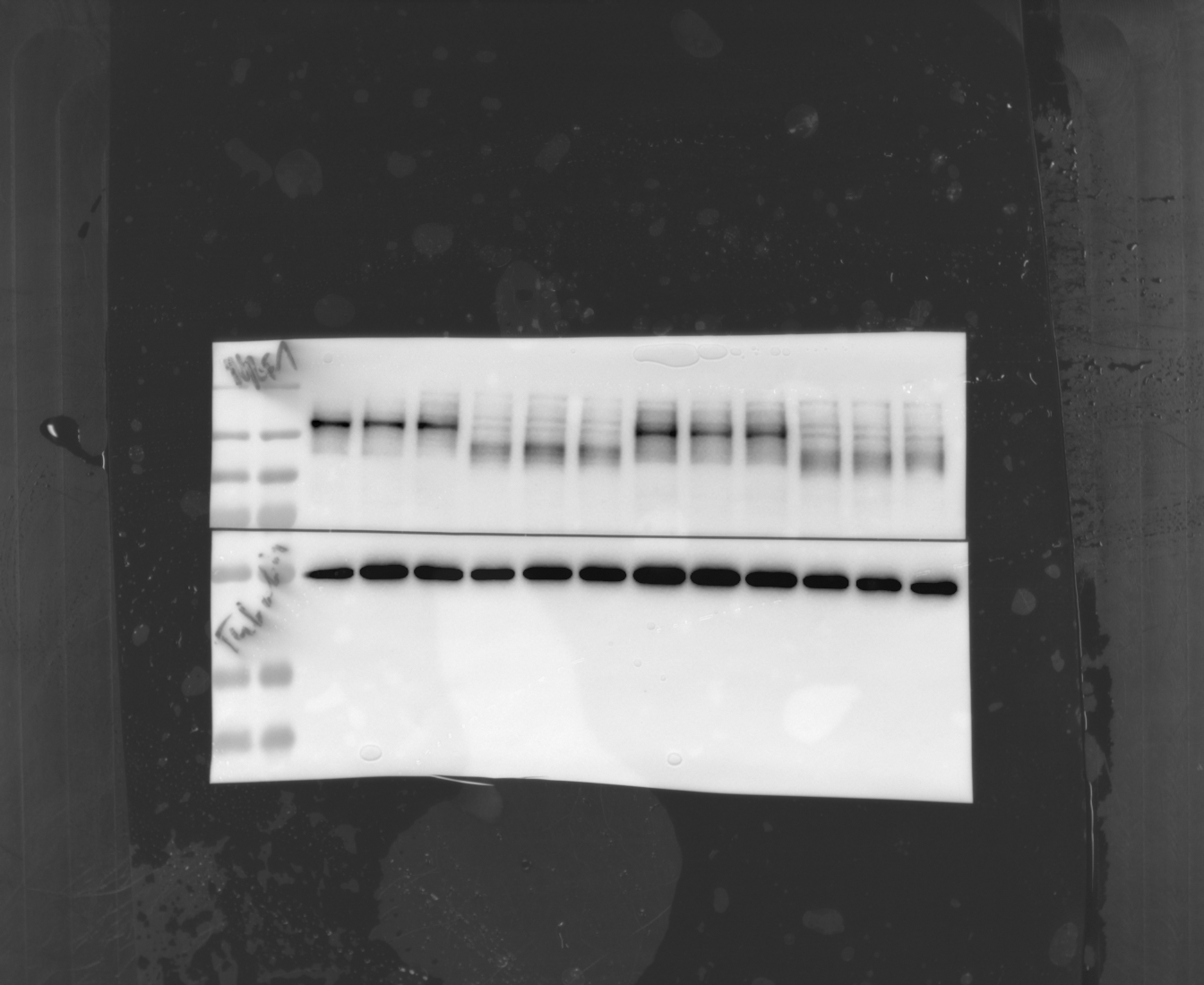

Supplement: Supplementary file 11 — Source Data for Figure 6 [file EMMM-15-e17761-s007.zip › Figure 6/6B/western NRF1 EV C2 KO C4 marker.Tif]

1  
2  
3  
4  
5  
6  
7  
8  
9  
10  
11  
12

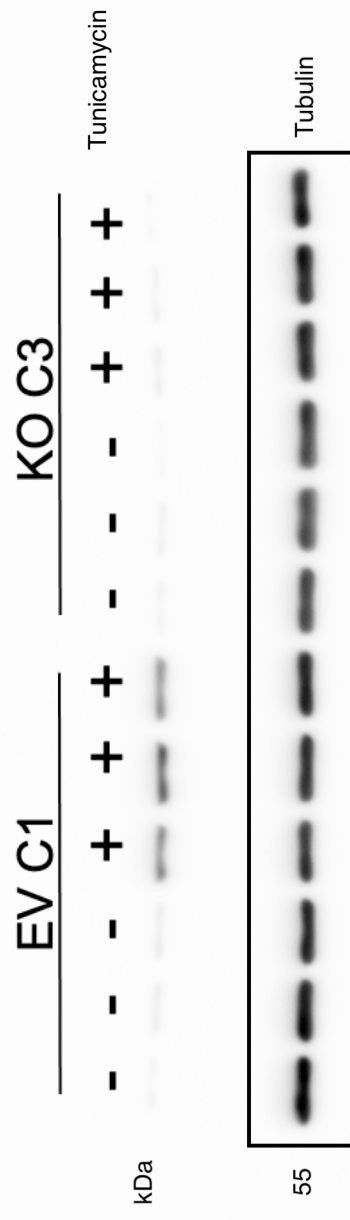

Supplement: Supplementary file 11 — Source Data for Figure 6 [file EMMM-15-e17761-s007.zip › Figure 6/6B/western Tubulin EV C1 KO C3 labelled .pdf]

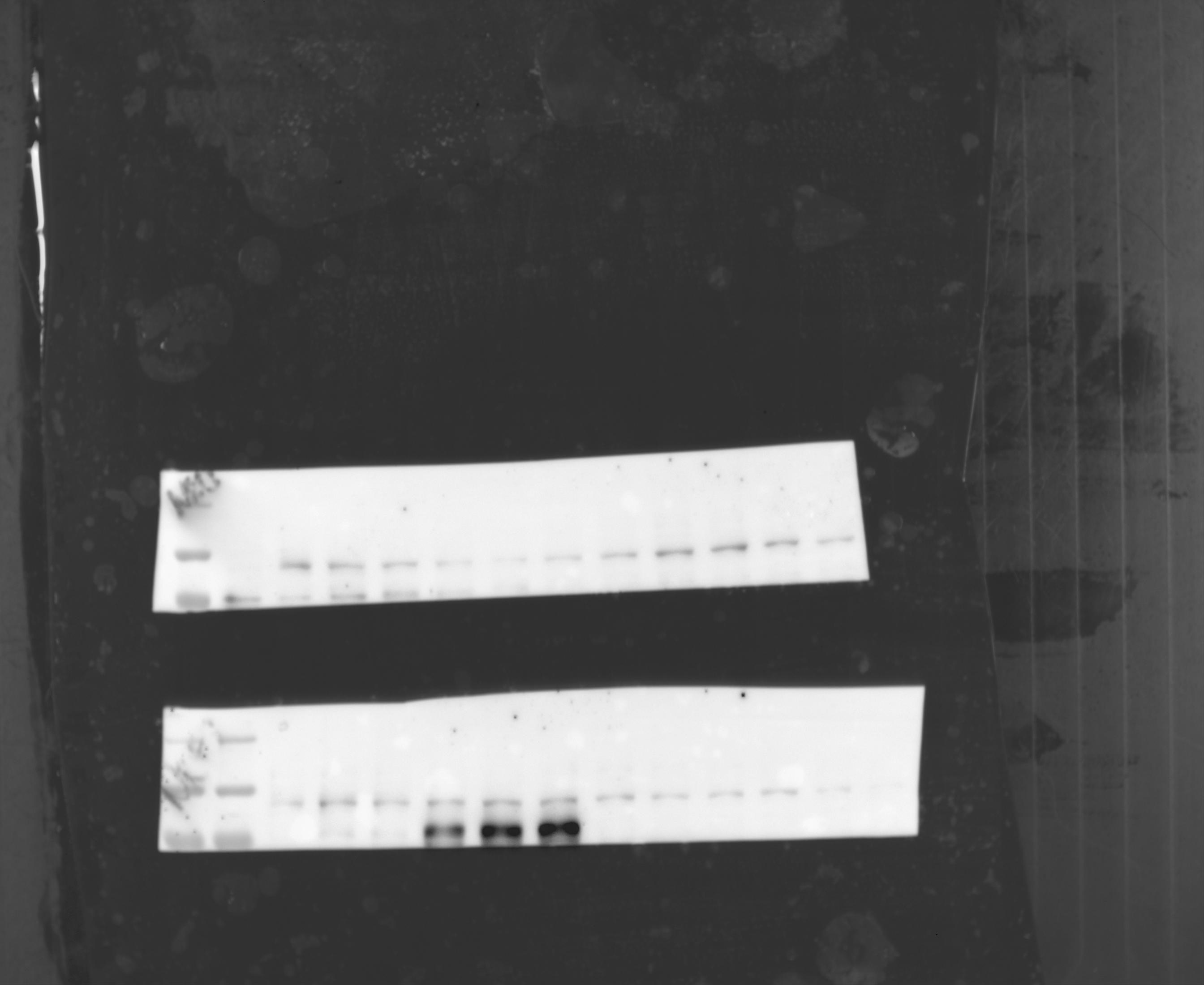

Supplement: Supplementary file 11 — Source Data for Figure 6 [file EMMM-15-e17761-s007.zip › Figure 6/6B/western NRF3 EV C1 KO C3 marker.Tif]

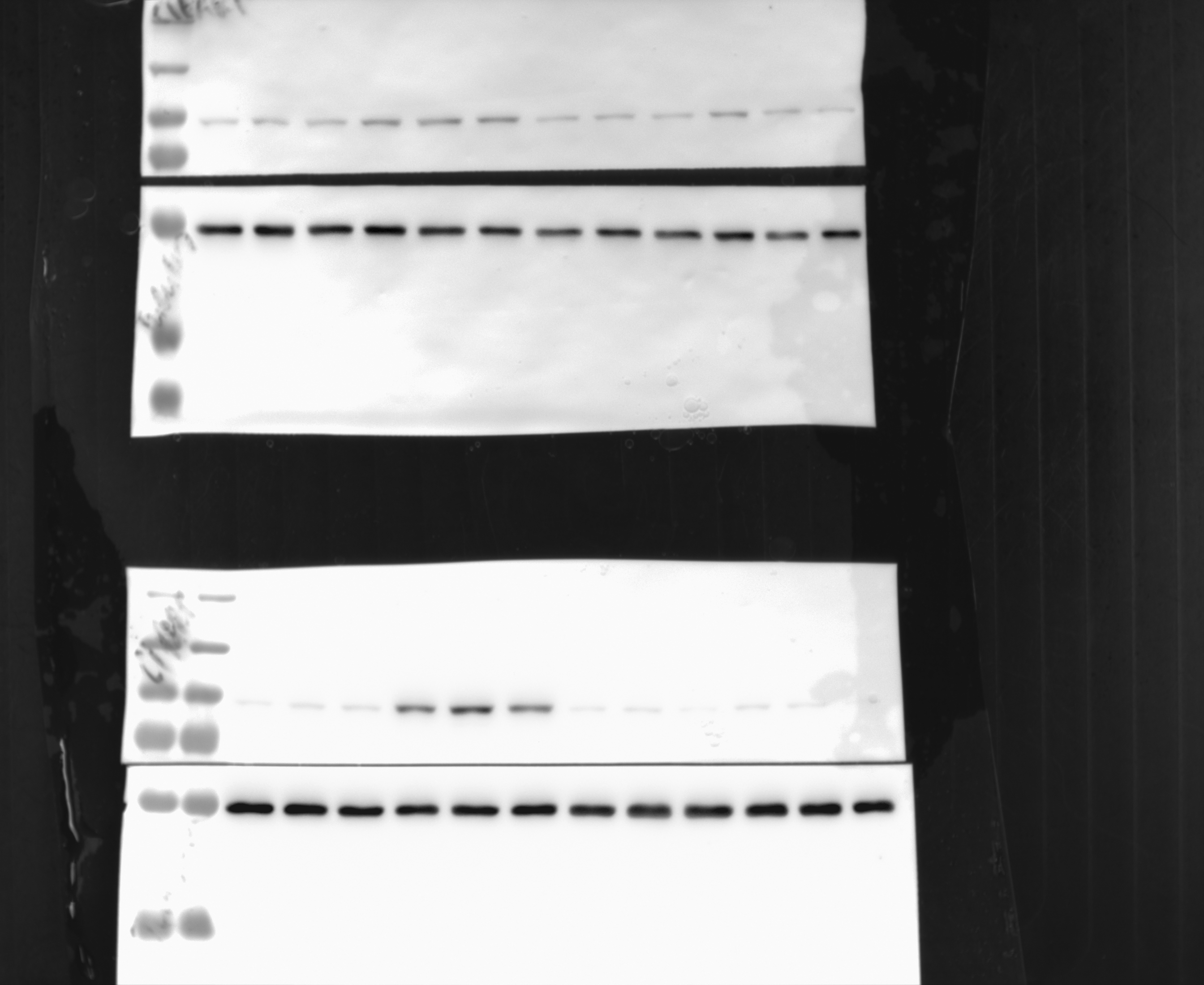

Supplement: Supplementary file 11 — Source Data for Figure 6 [file EMMM-15-e17761-s007.zip › Figure 6/6B/western clPARP Tubulin EV C1 KO C3 marker.Tif]

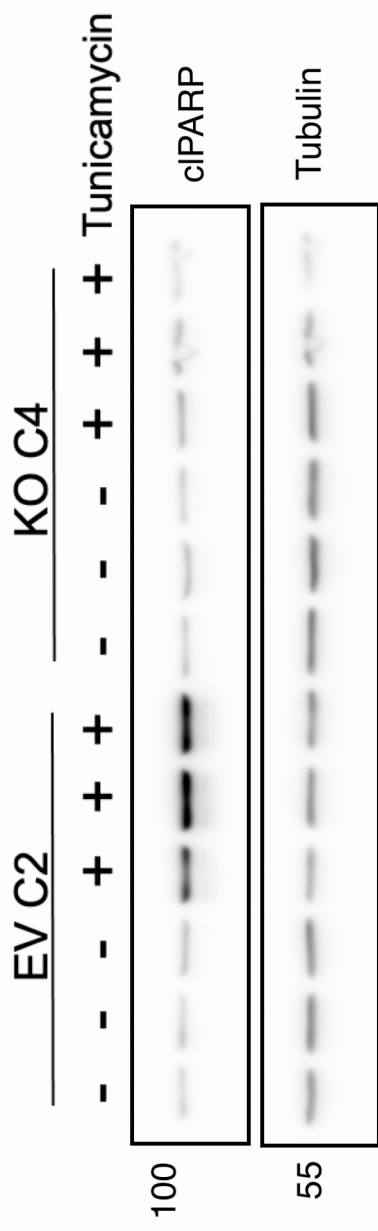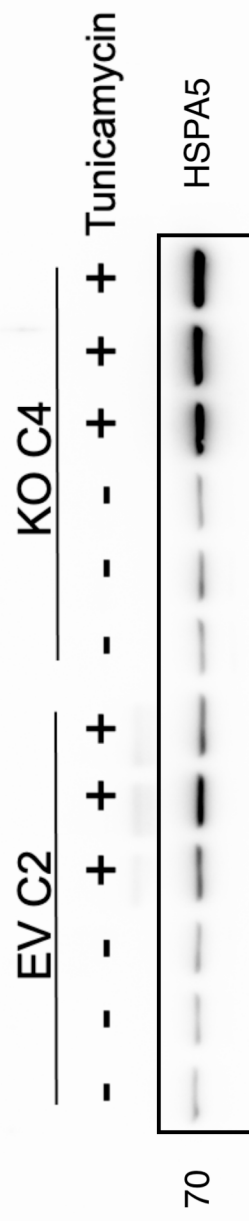

Supplement: Supplementary file 11 — Source Data for Figure 6 [file EMMM-15-e17761-s007.zip › Figure 6/6B/western clPARP Tubulin HSPA5 EV C2 KO C4 labelled.pdf]

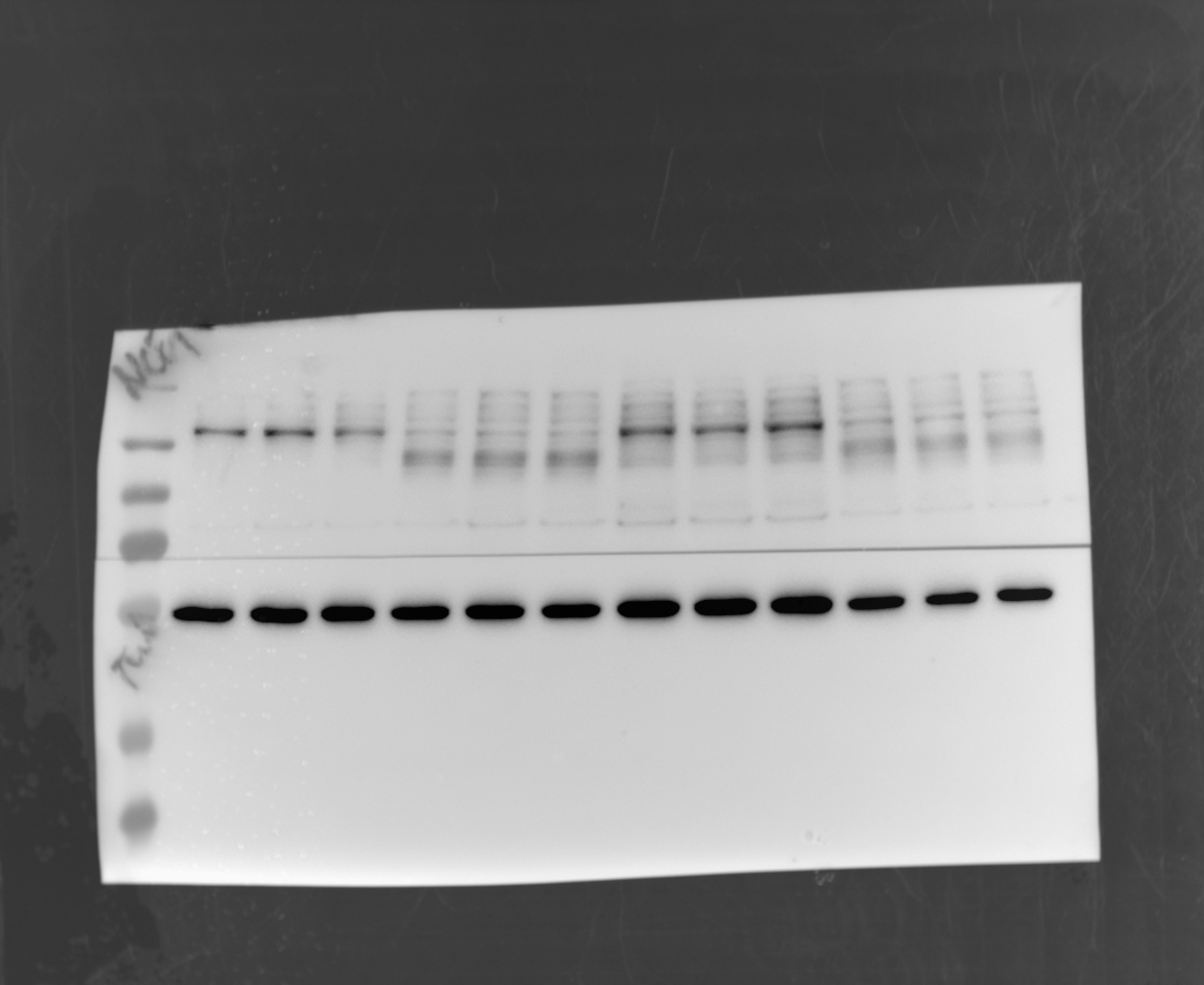

Supplement: Supplementary file 11 — Source Data for Figure 6 [file EMMM-15-e17761-s007.zip › Figure 6/6B/western NRF1 EV C1 KO C3 marker.Tif]

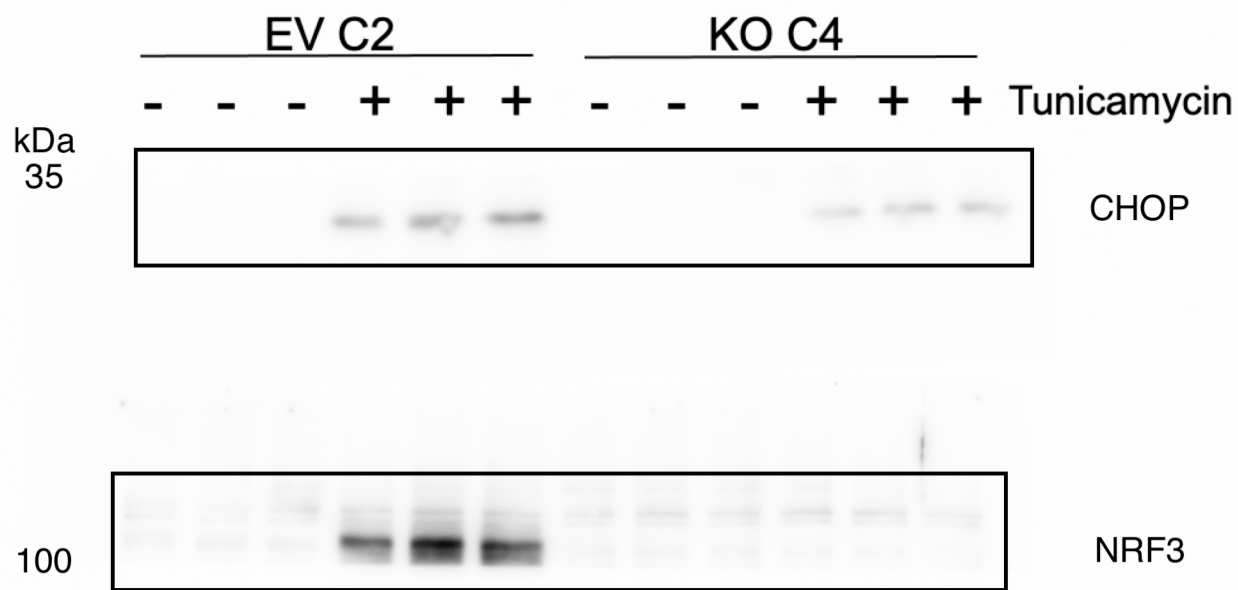

Supplement: Supplementary file 11 — Source Data for Figure 6 [file EMMM-15-e17761-s007.zip › Figure 6/6B/western CHOP NRF3 EV C2 KO C4 labelled.pdf]

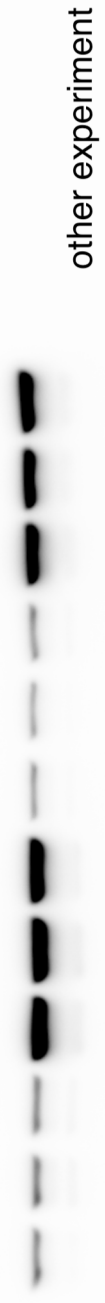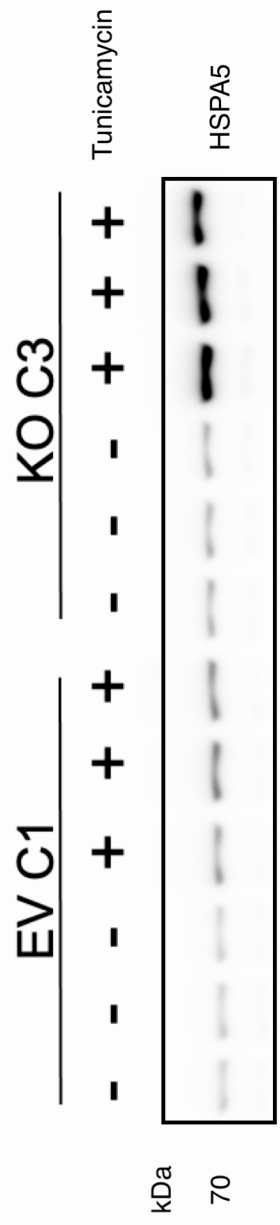

Supplement: Supplementary file 11 — Source Data for Figure 6 [file EMMM-15-e17761-s007.zip › Figure 6/6B/western HSPA5 EV C1 KO C3 labelled .pdf]

other experiment

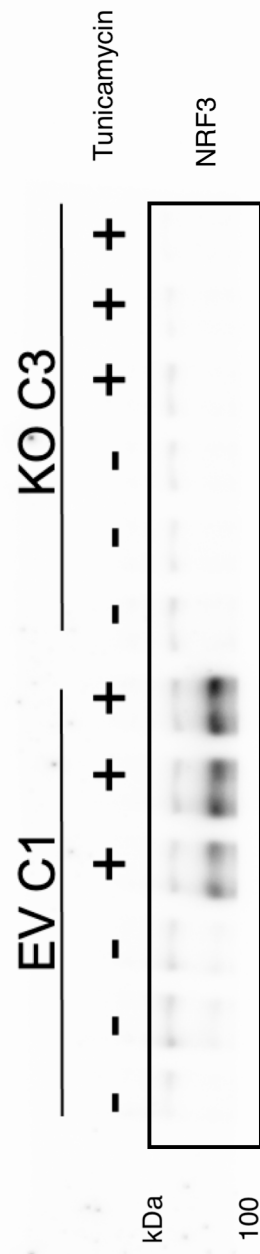

Supplement: Supplementary file 11 — Source Data for Figure 6 [file EMMM-15-e17761-s007.zip › Figure 6/6B/western NRF3 EV C1 KO C3 labelled .pdf]

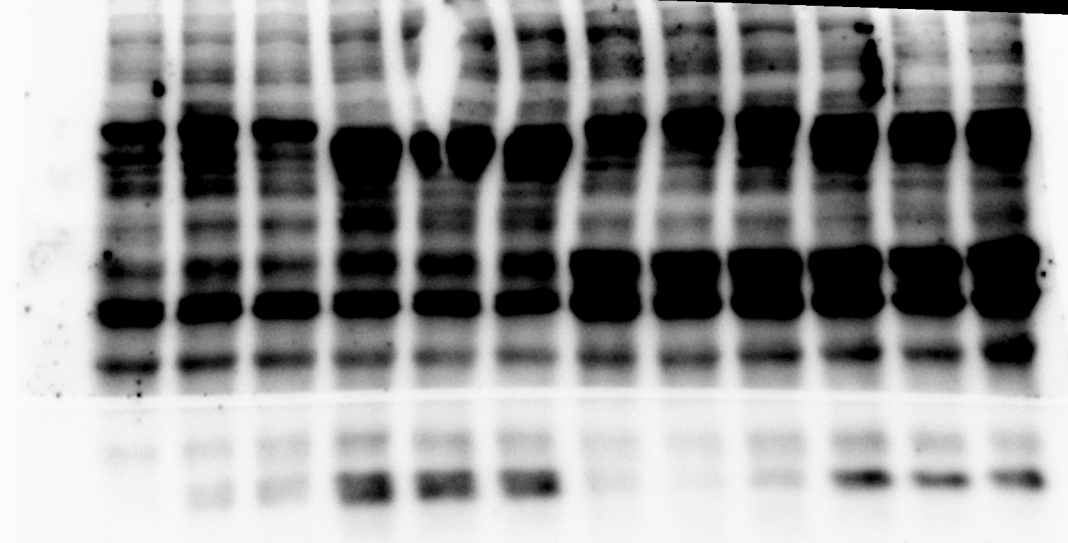

other experiment

EV C1

KO C3

- - - + + + - - - + + +

Tunicamycin

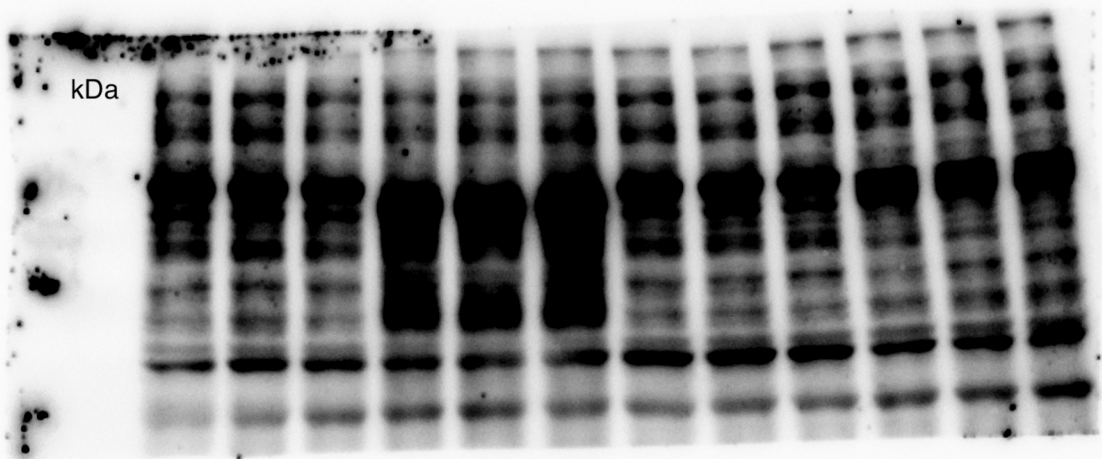

kDa

35

CHOP

Supplement: Supplementary file 11 — Source Data for Figure 6 [file EMMM-15-e17761-s007.zip › Figure 6/6B/western CHOP EV C1 KO C3 labelled.pdf]

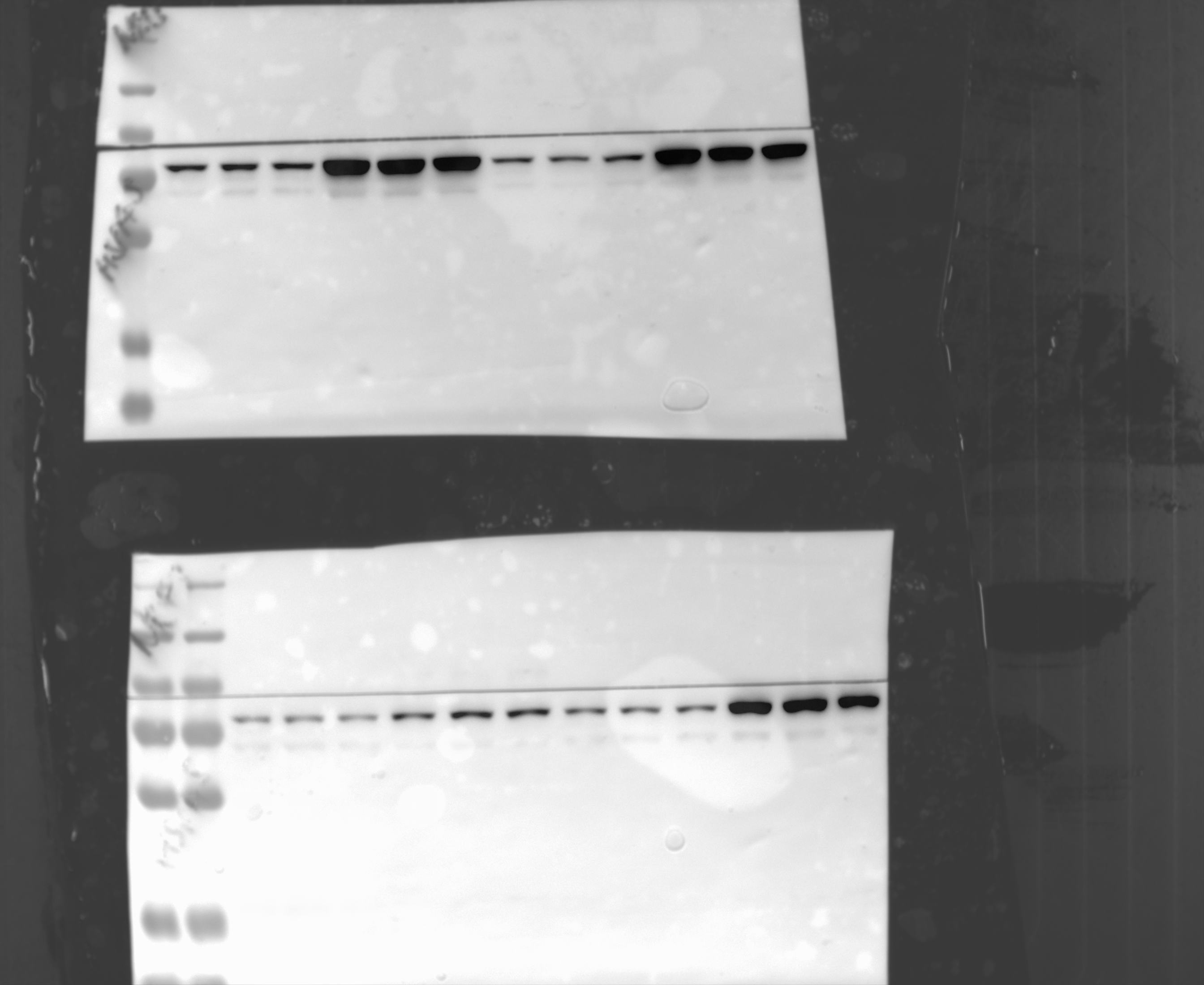

Supplement: Supplementary file 11 — Source Data for Figure 6 [file EMMM-15-e17761-s007.zip › Figure 6/6B/western HSPA5 EV C1 KO C3 marker.Tif]

other experiment

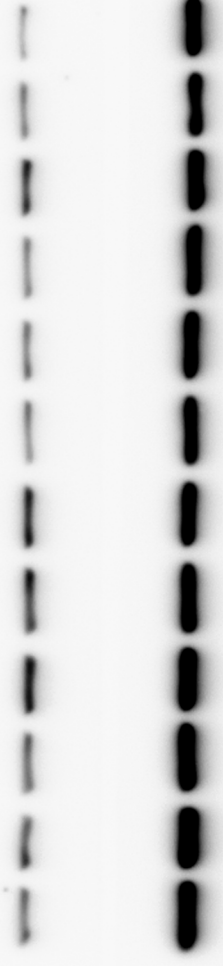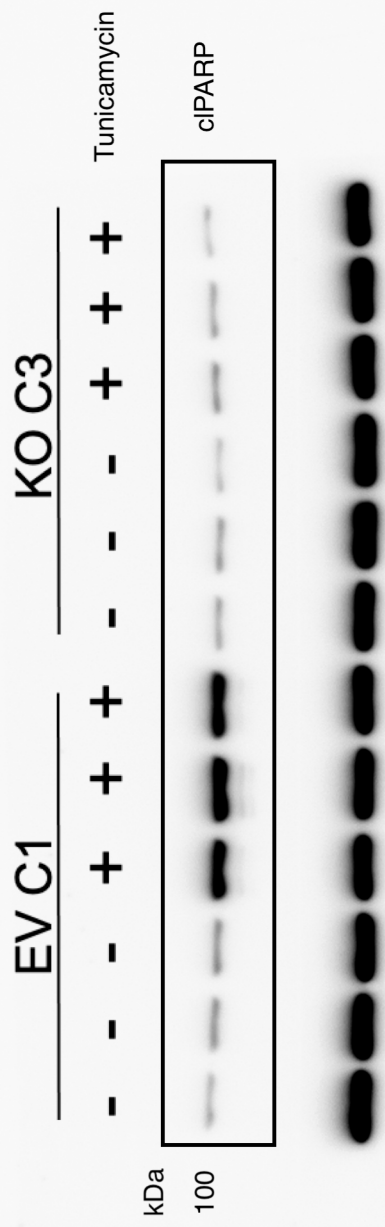

Supplement: Supplementary file 11 — Source Data for Figure 6 [file EMMM-15-e17761-s007.zip › Figure 6/6B/western clPARP EV C1 KO C3 labelled .pdf]

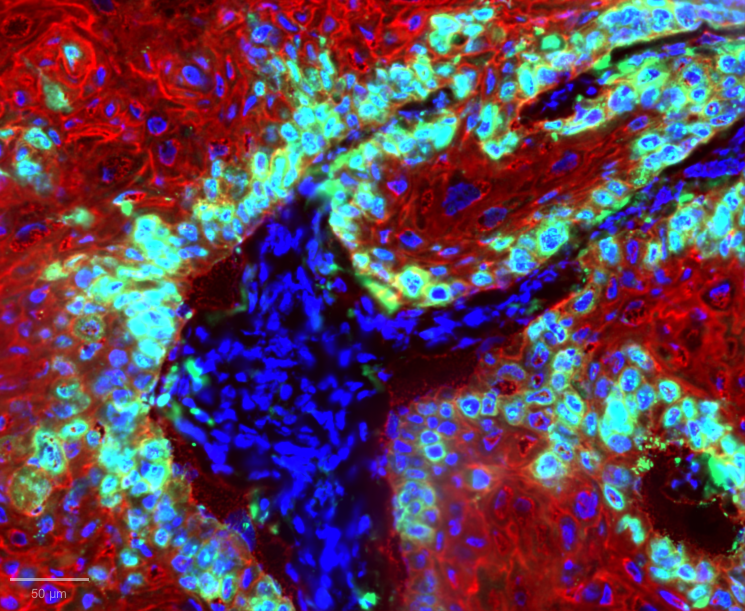

Supplement: Supplementary file 12 — Source Data for Figure 7 [file EMMM-15-e17761-s003.zip › Figure 7/7H/micro.image_KO vehicle higher magnification.png]

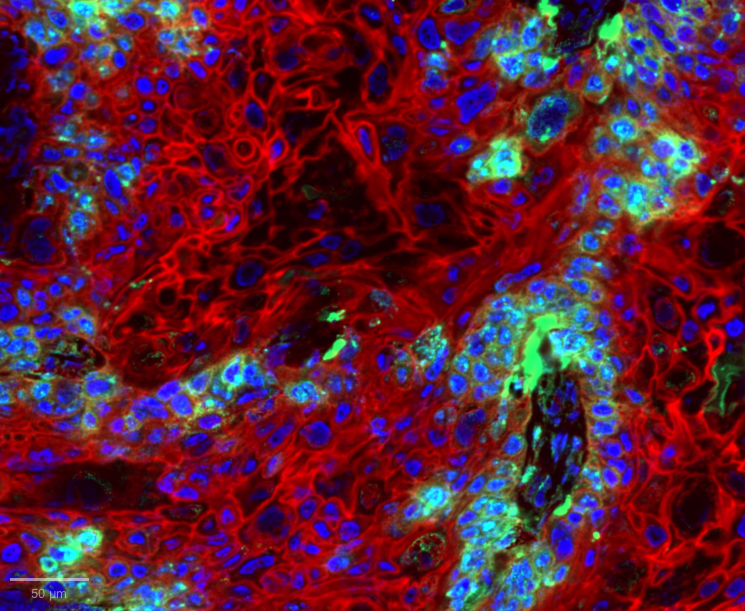

Supplement: Supplementary file 12 — Source Data for Figure 7 [file EMMM-15-e17761-s003.zip › Figure 7/7H/micro.image_EV vehicle higher magnification.png]

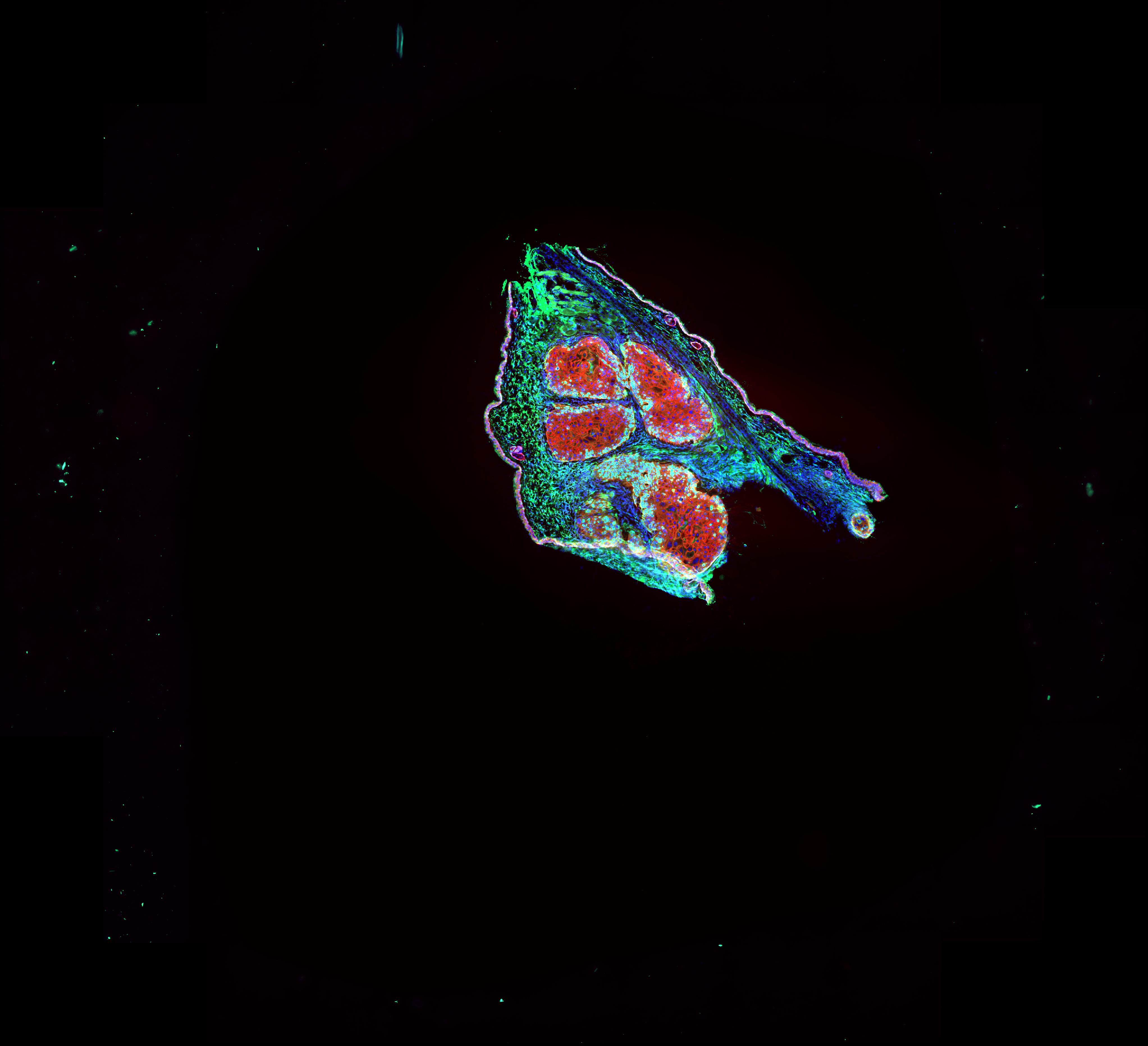

Supplement: Supplementary file 12 — Source Data for Figure 7 [file EMMM-15-e17761-s003.zip › Figure 7/7H/micro.image_EV HA15.jpg]

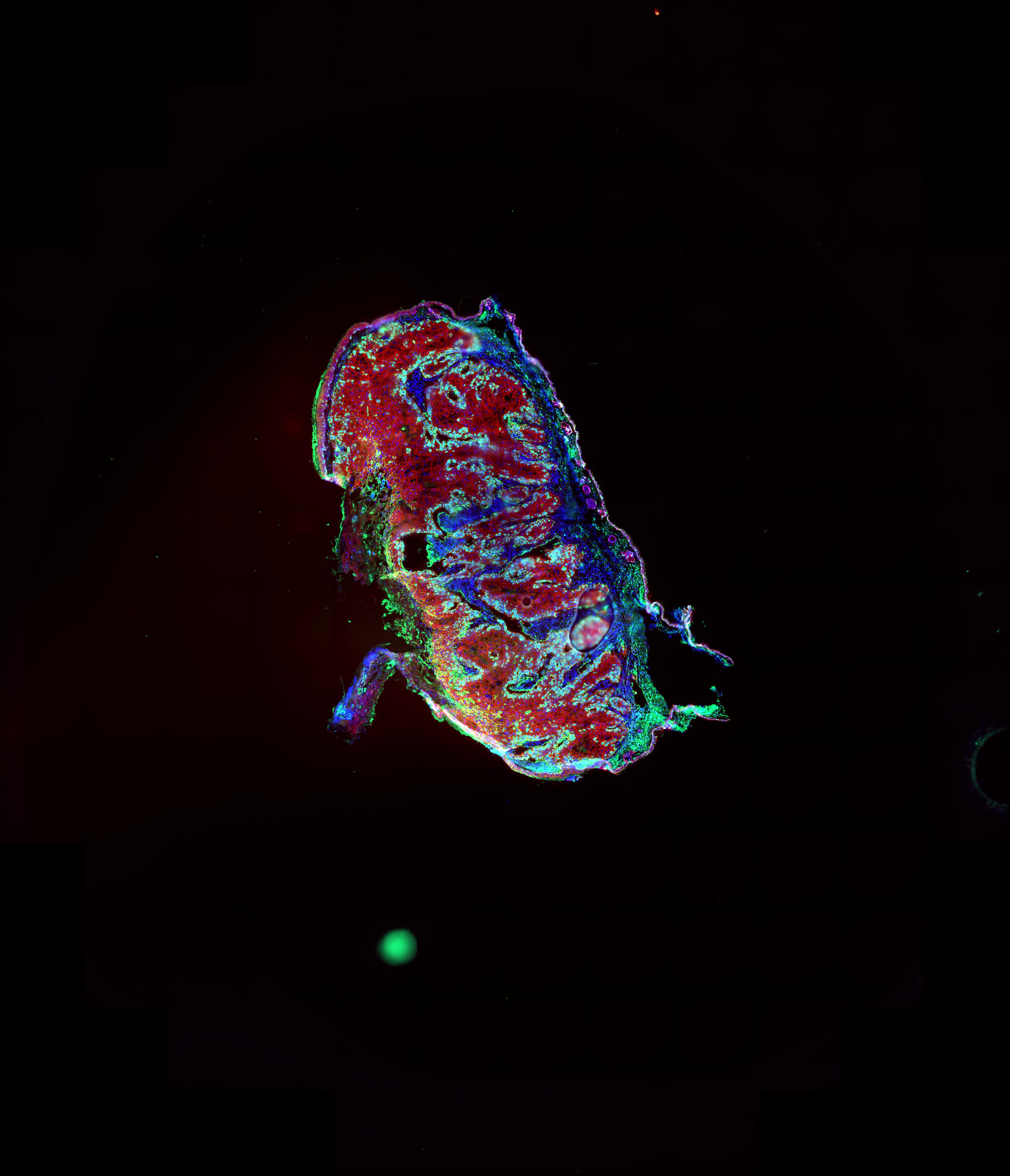

Supplement: Supplementary file 12 — Source Data for Figure 7 [file EMMM-15-e17761-s003.zip › Figure 7/7H/micro.image_KO vehicle.jpg]

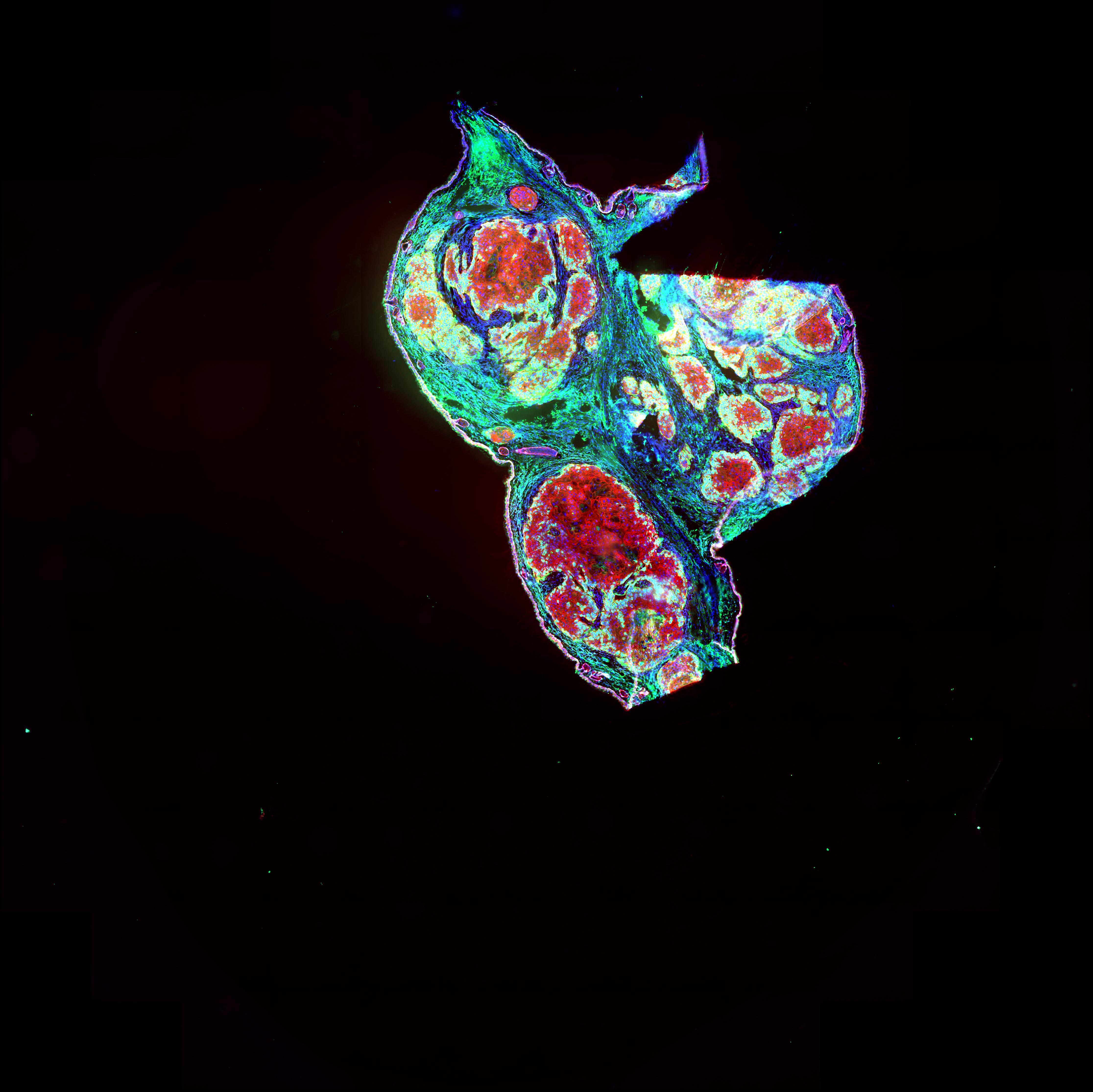

Supplement: Supplementary file 12 — Source Data for Figure 7 [file EMMM-15-e17761-s003.zip › Figure 7/7H/micro.image_KO HA15.jpg]

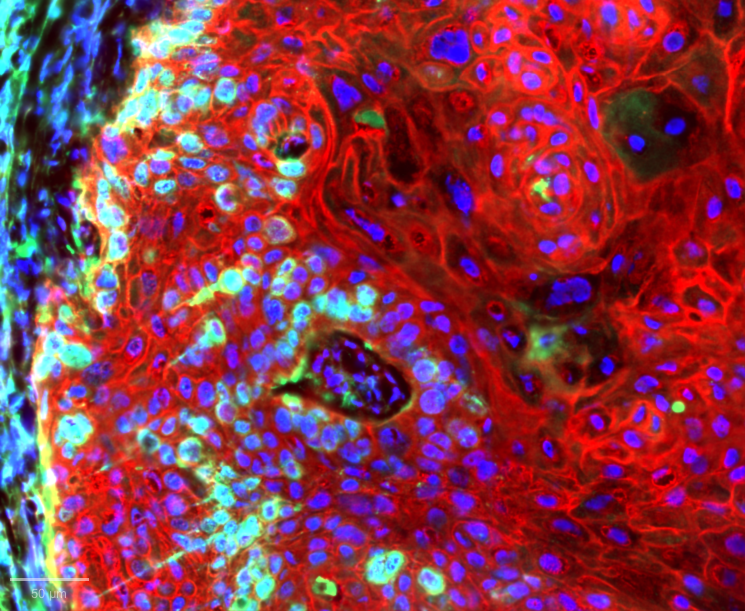

Supplement: Supplementary file 12 — Source Data for Figure 7 [file EMMM-15-e17761-s003.zip › Figure 7/7H/micro.image_KO HA15 higher magnification.png]

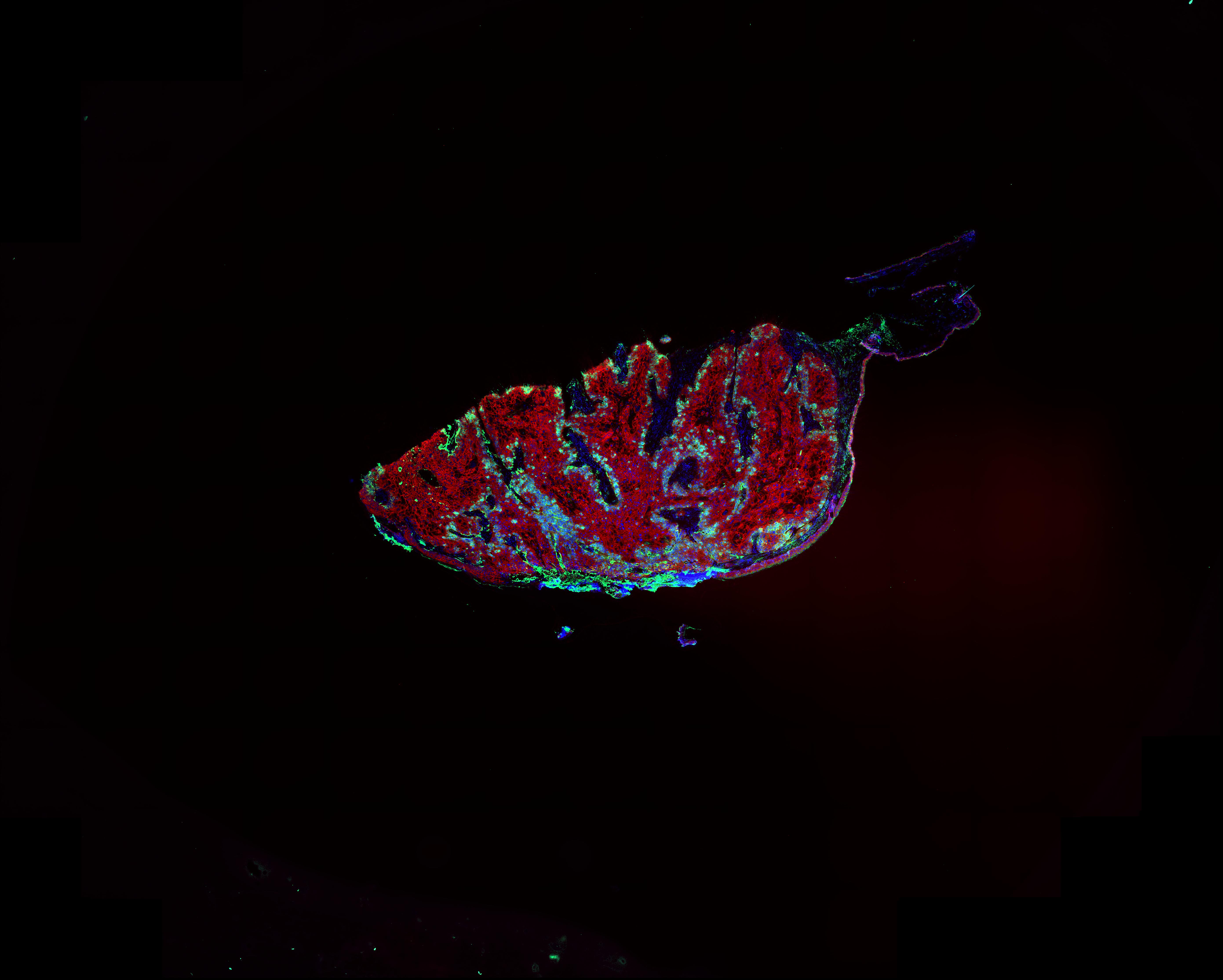

Supplement: Supplementary file 12 — Source Data for Figure 7 [file EMMM-15-e17761-s003.zip › Figure 7/7H/micro.image_EV vehicle.jpg]

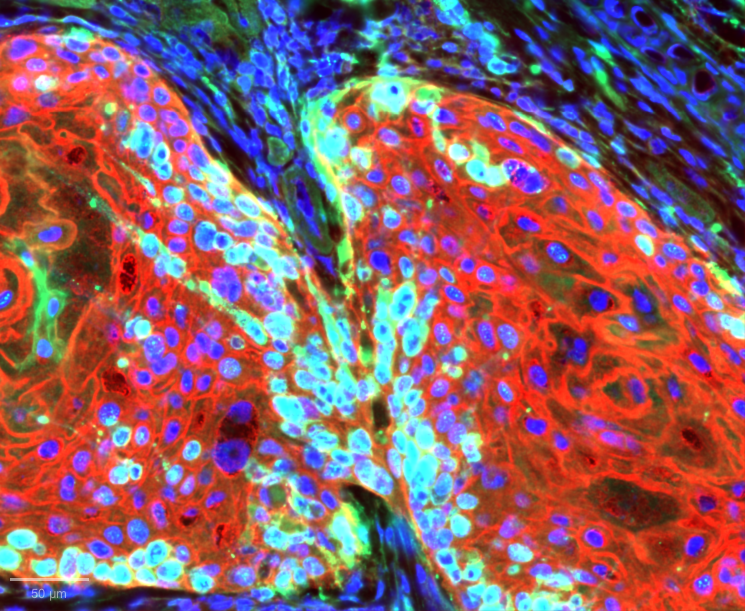

Supplement: Supplementary file 12 — Source Data for Figure 7 [file EMMM-15-e17761-s003.zip › Figure 7/7H/micro.image_EV HA15 higher magnification.png]

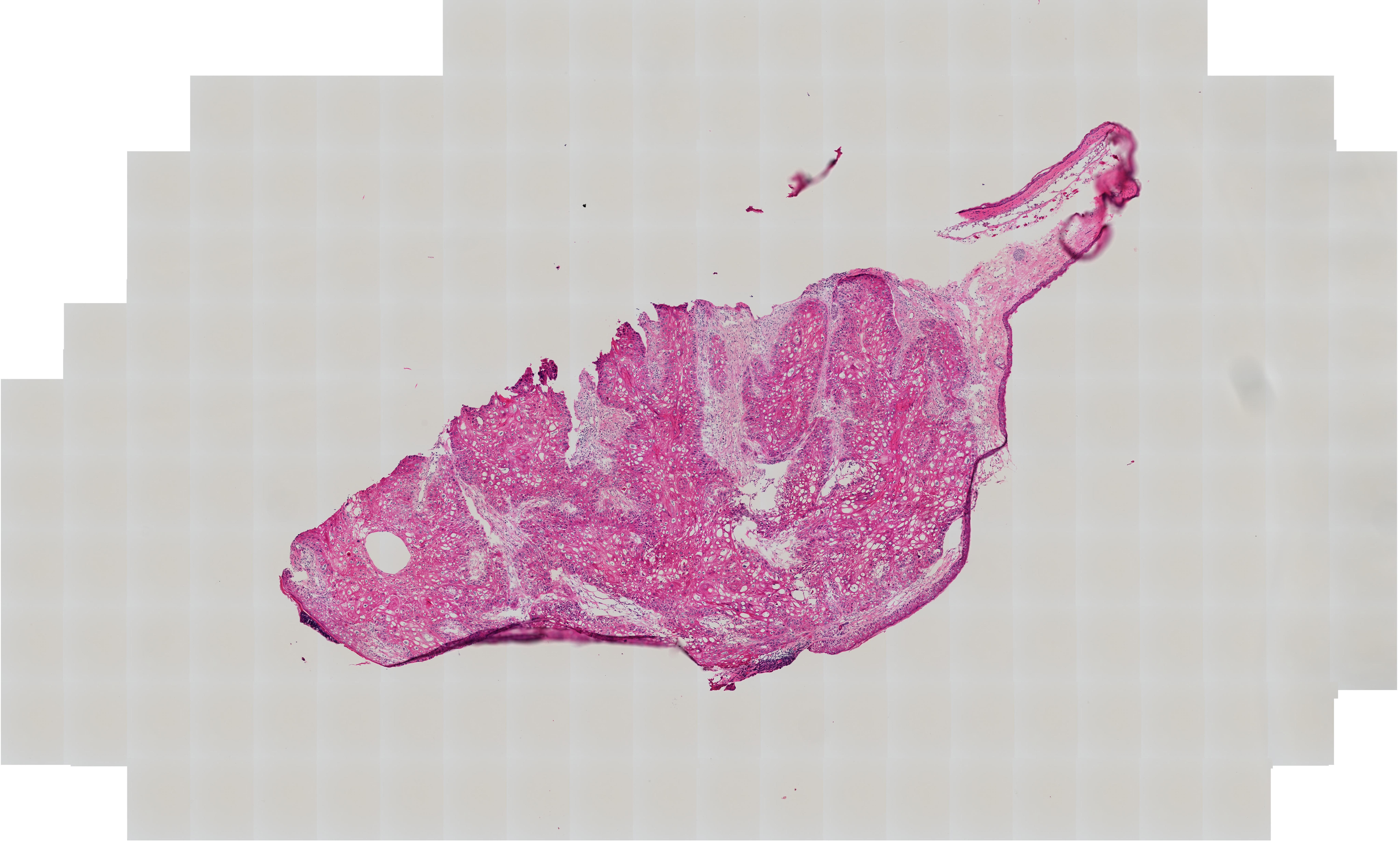

Supplement: Supplementary file 12 — Source Data for Figure 7 [file EMMM-15-e17761-s003.zip › Figure 7/7F-G/micro.image_EV vehicle.jpg]

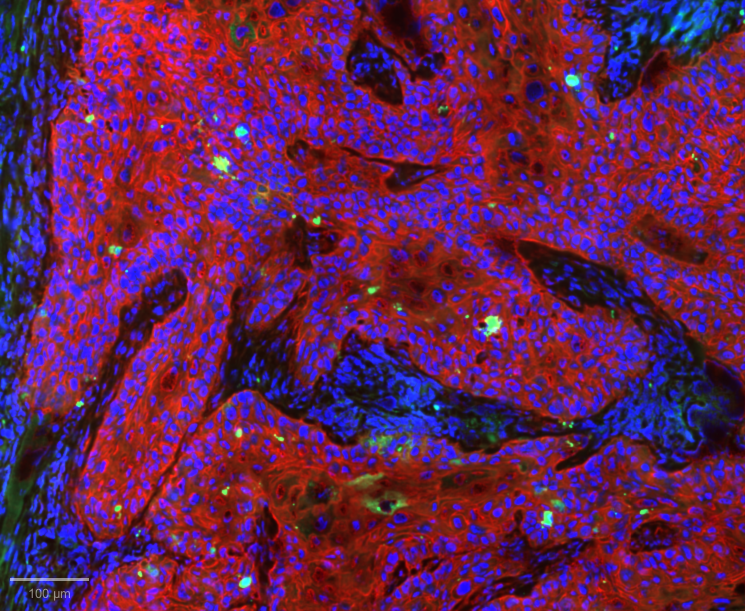

Supplement: Supplementary file 12 — Source Data for Figure 7 [file EMMM-15-e17761-s003.zip › Figure 7/7I/micro.image_KO vehicle higher magnification.png]

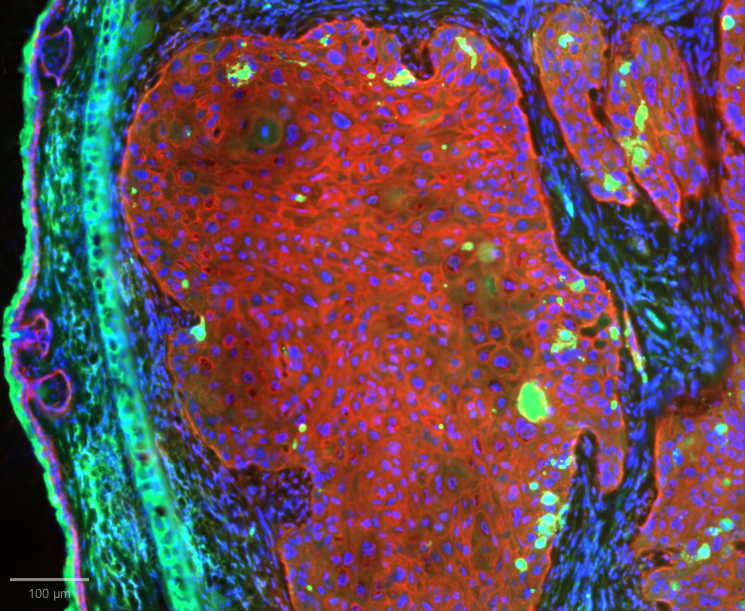

Supplement: Supplementary file 12 — Source Data for Figure 7 [file EMMM-15-e17761-s003.zip › Figure 7/7I/micro.image_EV vehicle higher magnification.png]

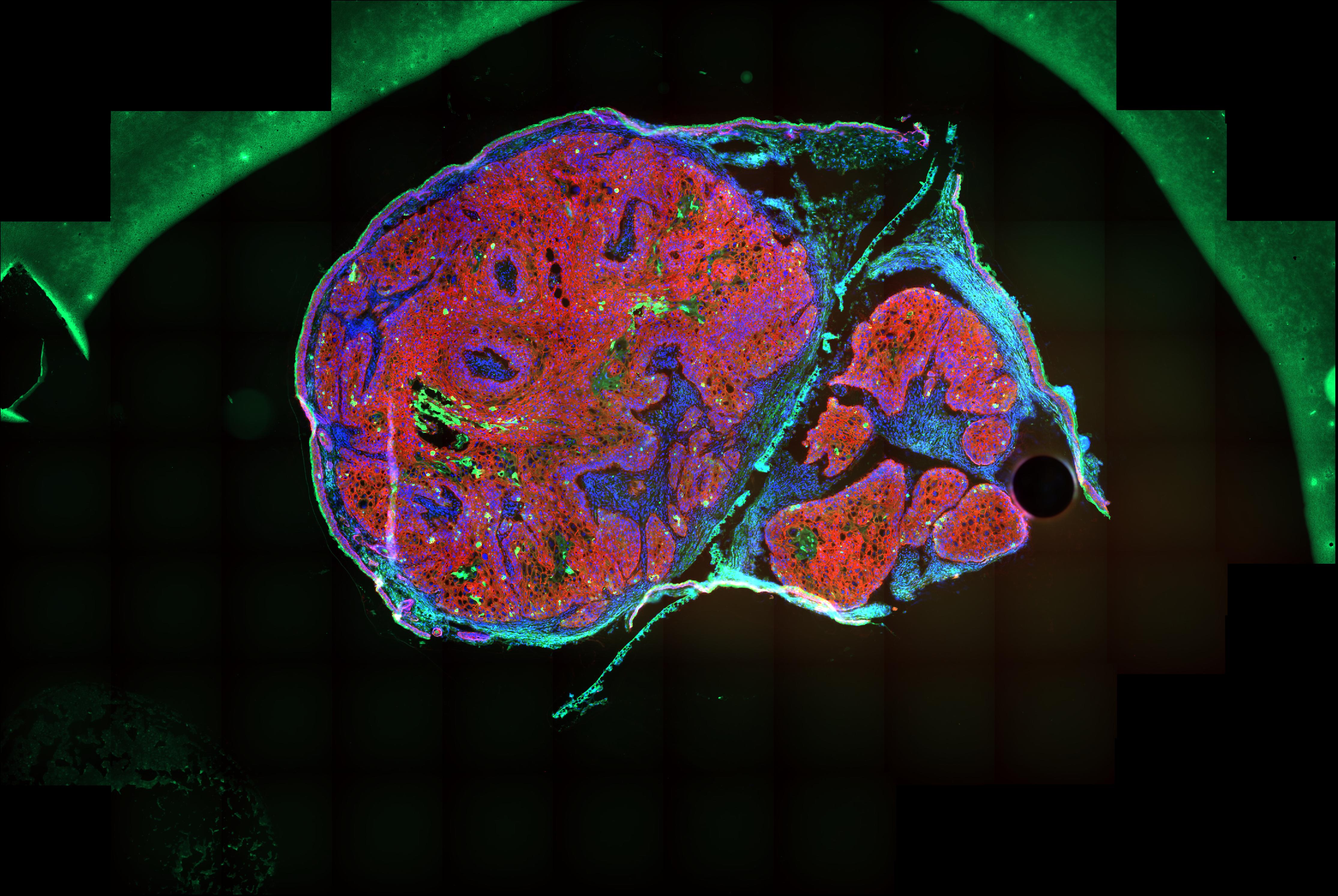

Supplement: Supplementary file 12 — Source Data for Figure 7 [file EMMM-15-e17761-s003.zip › Figure 7/7I/micro.image_EV HA15.jpg]

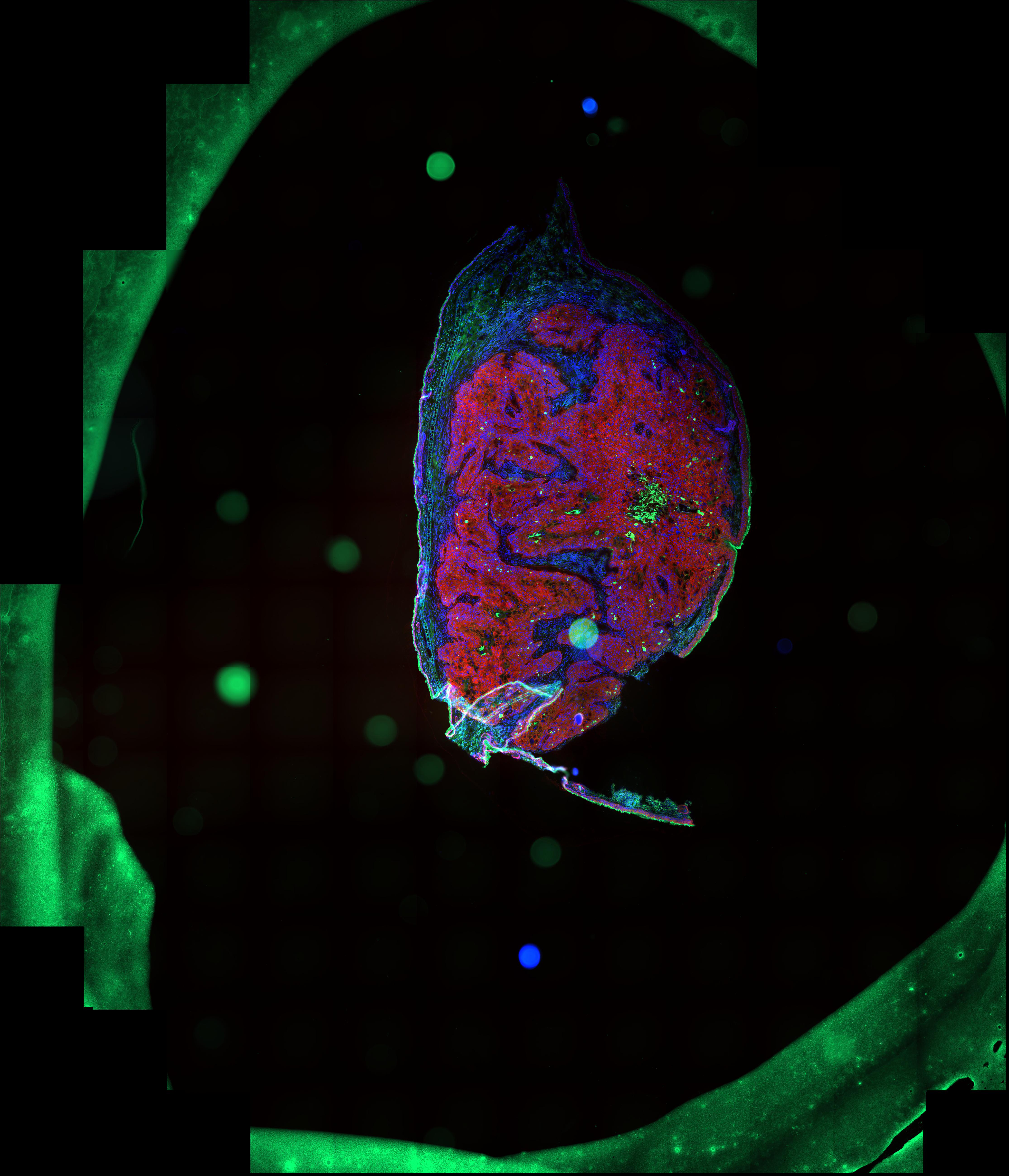

Supplement: Supplementary file 12 — Source Data for Figure 7 [file EMMM-15-e17761-s003.zip › Figure 7/7I/micro.image_KO vehicle.jpg]

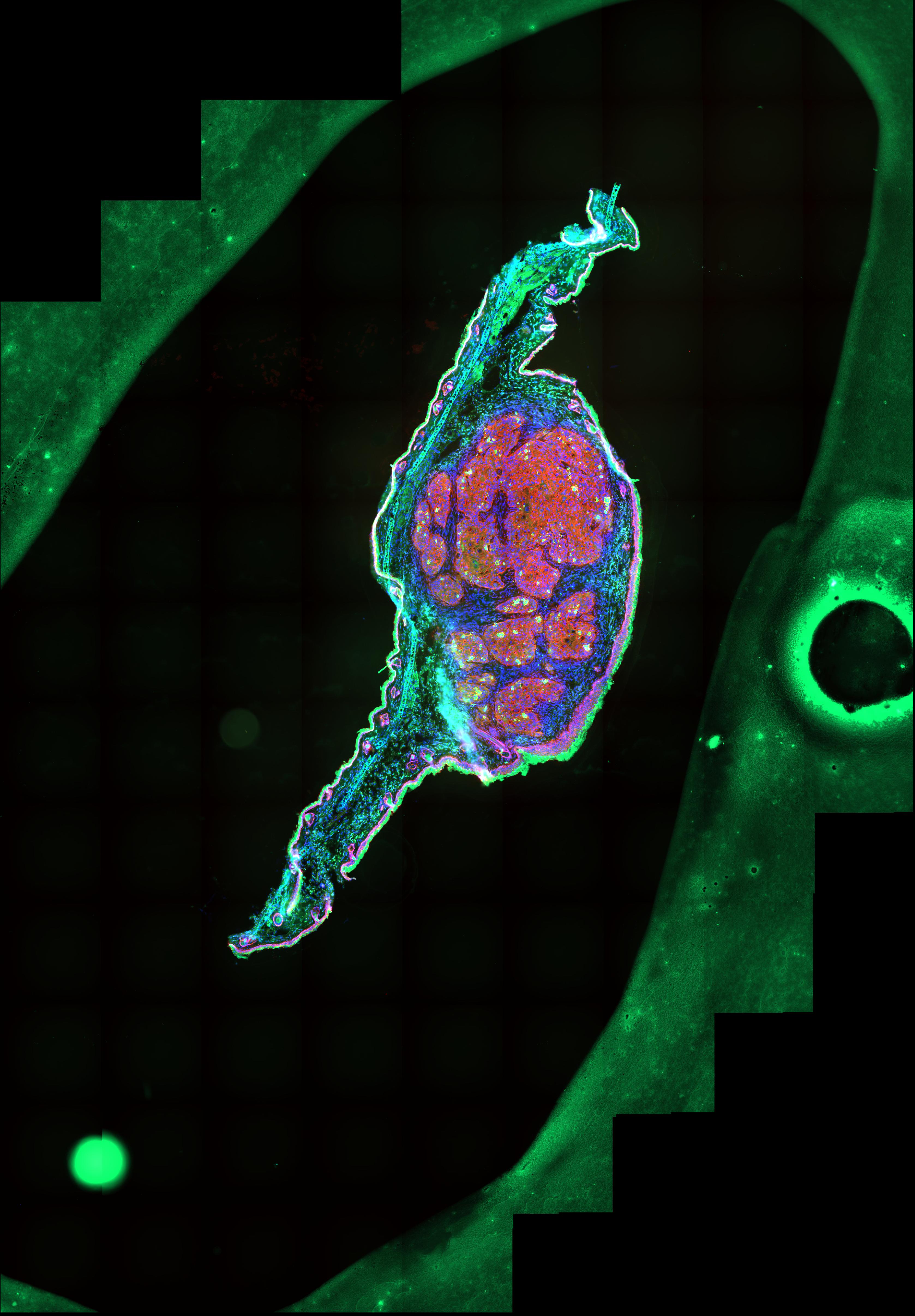

Supplement: Supplementary file 12 — Source Data for Figure 7 [file EMMM-15-e17761-s003.zip › Figure 7/7I/micro.image_KO HA15.jpg]

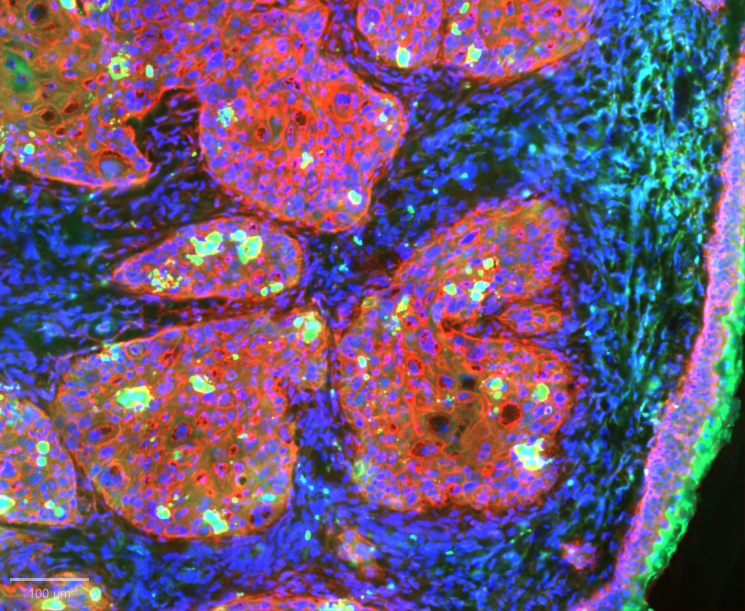

Supplement: Supplementary file 12 — Source Data for Figure 7 [file EMMM-15-e17761-s003.zip › Figure 7/7I/micro.image_KO HA15 higher magnification.png]

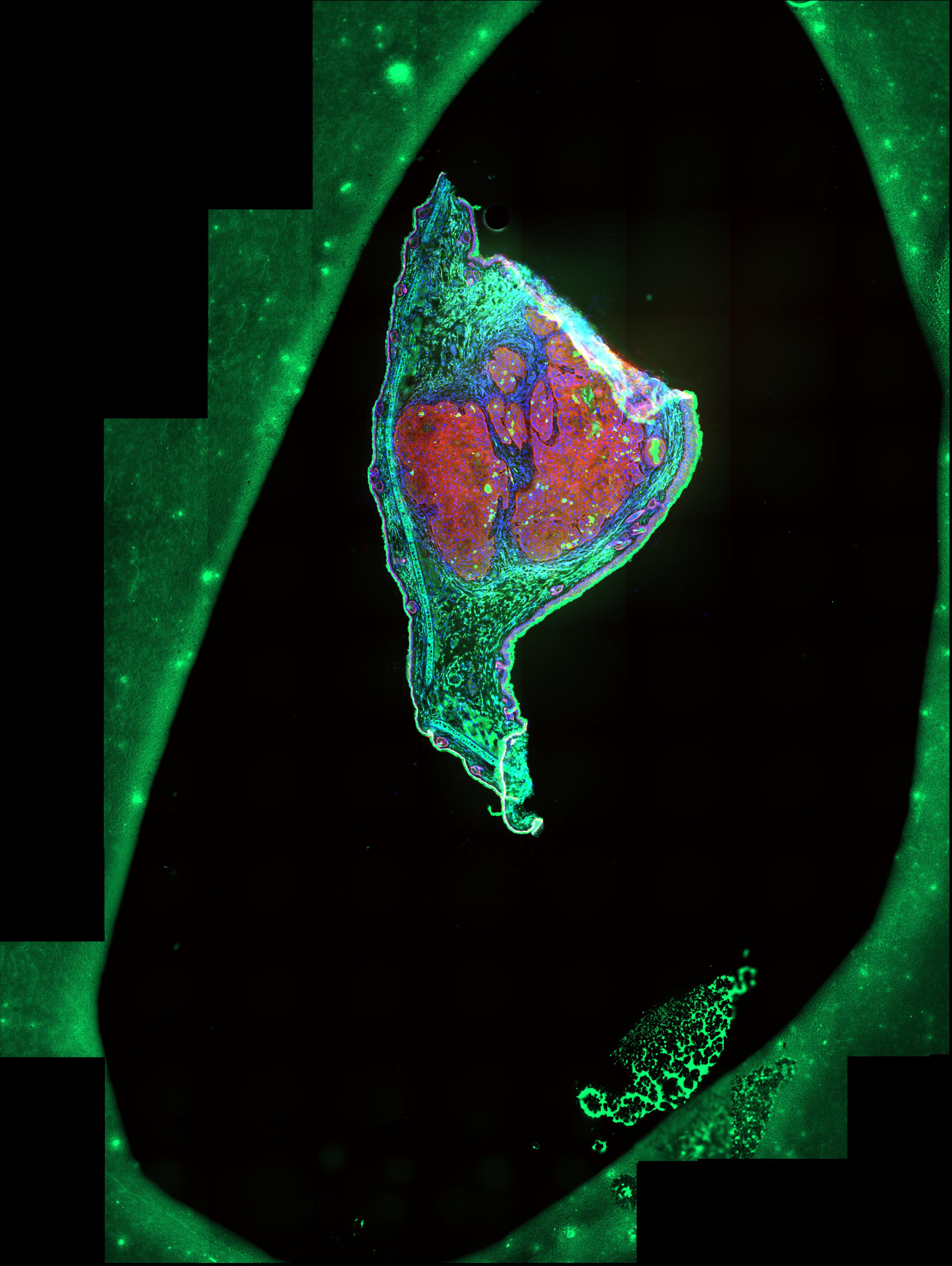

Supplement: Supplementary file 12 — Source Data for Figure 7 [file EMMM-15-e17761-s003.zip › Figure 7/7I/micro.image_EV vehicle.jpg]
