# Supplementary figures and images for: NRF3 suppresses squamous carcinogenesis, involving the unfolded protein response regulator HSPA5 (part 2 of 2)
Source: EMBO Mol Med. 2023 Oct 9;15(11):e17761. doi: 10.15252/emmm.202317761 (PMC10630885; doi:10.15252/emmm.202317761)

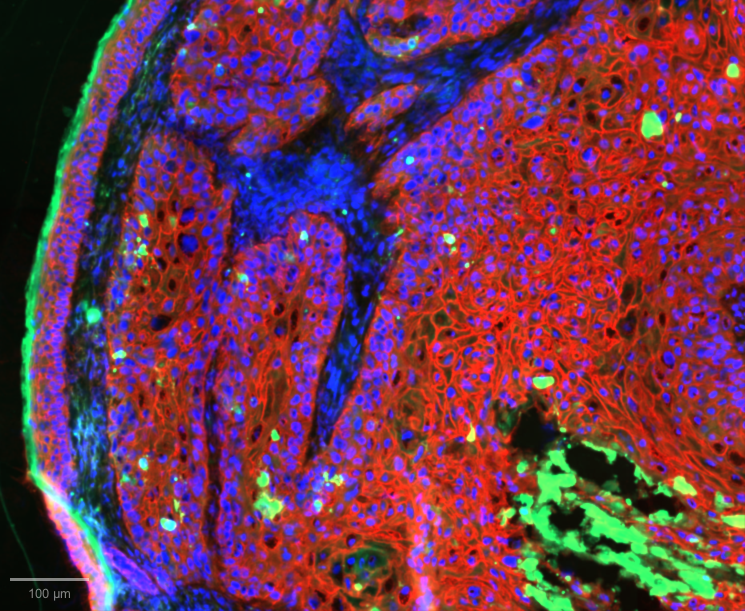

Supplement: Supplementary file 12 — Source Data for Figure 7 [file EMMM-15-e17761-s003.zip › Figure 7/7I/micro.image_EV HA15 higher magnification.png]
