# Supplementary material for: NRF3 suppresses squamous carcinogenesis, involving the unfolded protein response regulator HSPA5
Source: EMBO Mol Med. 2023 Oct 9;15(11):e17761. doi: 10.15252/emmm.202317761 (PMC10630885; doi:10.15252/emmm.202317761)
Supplement: Supplementary file 13 [file EMMM-15-e17761-s012.pdf]

EMBO Molecular Medicine

NRF3 suppresses squamous carcinogenesis, involving the unfolded protein response regulator HSPA5

Selina Gurri, Beat Siegenthaler, Michael Cangkrama, Gaetana Restivo, Marcel Huber, James Saliba, Reinhard Dummer, Volker Blank, Daniel Hohl, and Sabine Werner
DOI: 10.15252/emmm.202317761

Corresponding authors: Sabine Werner (sabine.werner@biol.ethz.ch)

Review Timeline:

Submission Date:	28th Mar 23
Editorial Decision:	25th Apr 23
Revision Received:	12th Jul 23
Editorial Decision:	14th Aug 23
Revision Received:	5th Sep 23
Accepted:	15th Sep 23

Editor: Lise Roth

Transaction Report:

(Note: With the exception of the correction of typographical or spelling errors that could be a source of ambiguity, letters and reports are not edited. Depending on transfer agreements, referee reports obtained elsewhere may or may not be included in this compilation. Referee reports are anonymous unless the Referee chooses to sign their reports.)

25th Apr 2023

Dear Sabine,

Thank you for the submission of your manuscript to EMBO Molecular Medicine. We have now received feedback from the three reviewers who agreed to evaluate your manuscript. As you will see from the reports below, the referees acknowledge the interest of the study and are overall supporting publication of your work pending appropriate revisions.

After cross-commenting with the referees, referee #3 agreed that bone marrow chimeras would be nice to have but not mandatory (point #1 from referee #3), but NRF3 expression in immune cells should be shown. Similarly, we agreed that it will not be required to repeat all experiments in NRF2;NRF3 DKO cells (point #1 from referee #2), but this point should be adequately discussed.

Addressing the other reviewers' concerns in full will be necessary for further considering the manuscript in our journal, and acceptance of the manuscript will entail a second round of review. EMBO Molecular Medicine encourages a single round of revision only and therefore, acceptance or rejection of the manuscript will depend on the completeness of your responses included in the next, final version of the manuscript. For this reason, and to save you from any frustrations in the end, I would strongly advise against returning an incomplete revision.

If you would like to discuss further the points raised by the referees, I am available to do so via email or video. Let me know if you are interested in this option.

Revised manuscripts should be submitted within three months of a request for revision; they will otherwise be treated as new submissions, except under exceptional circumstances in which a short extension is obtained from the editor.

When submitting your revised manuscript, please carefully review the instructions that follow below. We perform an initial quality control of all revised manuscripts before re-review; failure to include requested items will delay the evaluation of your revision.

We require:

1) A .docx formatted version of the manuscript text (including legends for main figures, EV figures and tables). Please make sure that the changes are highlighted to be clearly visible.

2) Individual production quality figure files as .eps, .tif, .jpg (one file per figure). For guidance, download the 'Figure Guide PDF' (https://www.embopress.org/page/journal/17574684/authorguide#figureformat).

3) At EMBO Press we ask authors to provide source data for the main figures. Our source data coordinator will contact you to discuss which figure panels we would need source data for and will also provide you with helpful tips on how to upload and organize the files.

4) A .docx formatted letter INCLUDING the reviewers' reports and your detailed point-by-point responses to their comments. As part of the EMBO Press transparent editorial process, the point-by-point response is part of the Review Process File (RPF), which will be published alongside your paper.

5) A complete author checklist, which you can download from our author guidelines (https://www.embopress.org/page/journal/17574684/authorguide#submissionofrevisions). Please insert information in the checklist that is also reflected in the manuscript. The completed author checklist will also be part of the RPF.

6) It is mandatory to include a 'Data Availability' section after the Materials and Methods. Before submitting your revision, primary datasets produced in this study need to be deposited in an appropriate public database, and the accession numbers and database listed under 'Data Availability'. Please remember to provide a reviewer password if the datasets are not yet public (see https://www.embopress.org/page/journal/17574684/authorguide#dataavailability).
Note that the Data Availability Section is restricted to new primary data that are part of this study.

7) For data quantification: please specify the name of the statistical test used to generate error bars and P values, the number (n) of independent experiments (specify technical or biological replicates) underlying each data point and the test used to calculate p-values in each figure legend. The figure legends should contain a basic description of n, P and the test applied. Graphs must include a description of the bars and the error bars (s.d., s.e.m.). Please provide exact p values.

8) Our journal encourages inclusion of *data citations in the reference list* to directly cite datasets that were re-used and

obtained from public databases. Data citations in the article text are distinct from normal bibliographical citations and should directly link to the database records from which the data can be accessed. In the main text, data citations are formatted as follows: "Data ref: Smith et al, 2001" or "Data ref: NCBI Sequence Read Archive PRJNA342805, 2017". In the Reference list, data citations must be labeled with "[DATASET]". A data reference must provide the database name, accession number/identifiers and a resolvable link to the landing page from which the data can be accessed at the end of the reference. Further instructions are available at .

9) We replaced Supplementary Information with Expanded View (EV) Figures and Tables that are collapsible/expandable online. A maximum of 5 EV Figures can be typeset. EV Figures should be cited as 'Figure EV1, Figure EV2" etc... in the text and their respective legends should be included in the main text after the legends of regular figures.
- For the figures that you do NOT wish to display as Expanded View figures, they should be bundled together with their legends in a single PDF file called *Appendix*, which should start with a short Table of Content. Appendix figures should be referred to in the main text as: "Appendix Figure S1, Appendix Figure S2" etc.
- Additional Tables/Datasets should be labeled and referred to as Table EV1, Dataset EV1, etc. Legends have to be provided in a separate tab in case of .xls files. Alternatively, the legend can be supplied as a separate text file (README) and zipped together with the Table/Dataset file.
See detailed instructions here:

10) For more information: There is space at the end of each article to list relevant web links for further consultation by our readers. Could you identify some relevant ones and provide such information as well? Some examples are patient associations, relevant databases, OMIM/proteins/genes links, author's websites, etc...

11) Author contributions: CRediT has replaced the traditional author contributions section because it offers a systematic machine readable author contributions format that allows for more effective research assessment. Please remove the Authors Contributions from the manuscript and use the free text boxes beneath each contributing author's name in our system to add specific details on the author's contribution. More information is available in our guide to authors.

12) Disclosure statement and competing interests: We updated our journal's competing interests policy in January 2022 and request authors to consider both actual and perceived competing interests. Please review the policy https://www.embopress.org/competing-interests and update your competing interests if necessary.

13) Every published paper now includes a 'Synopsis' to further enhance discoverability. Synopses are displayed on the journal webpage and are freely accessible to all readers. They include a short stand first (maximum of 300 characters, including space) as well as 2-5 one-sentences bullet points that summarizes the paper. Please write the bullet points to summarize the key NEW findings. They should be designed to be complementary to the abstract - i.e. not repeat the same text. We encourage inclusion of key acronyms and quantitative information (maximum of 30 words / bullet point). Please use the passive voice. Please attach these in a separate file or send them by email, we will incorporate them accordingly.

Please also suggest a striking image or visual abstract to illustrate your article as a PNG file 550 px wide x 300-600 px high.

14) As part of the EMBO Publications transparent editorial process initiative (see our Editorial at http://embomolmed.embopress.org/content/2/9/329), EMBO Molecular Medicine will publish online a Review Process File (RPF) to accompany accepted manuscripts.
In the event of acceptance, this file will be published in conjunction with your paper and will include the anonymous referee reports, your point-by-point response and all pertinent correspondence relating to the manuscript. Let us know whether you agree with the publication of the RPF and as here, if you want to remove or not any figures from it prior to publication.
Please note that the Authors checklist will be published at the end of the RPF.

EMBO Molecular Medicine has a "scooping protection" policy, whereby similar findings that are published by others during review or revision are not a criterion for rejection. Should you decide to submit a revised version, I do ask that you get in touch after three months if you have not completed it, to update us on the status.

I look forward to receiving your revised manuscript.

With kind regards,

Lise

Lise Roth, PhD
Senior Editor
EMBO Molecular Medicine

***** Reviewer's comments *****

Referee #1 (Remarks for Author):

This manuscript by Gurri and colleagues reveals a tumor suppressive role for Nrf3 in skin cancers. They further identified Nrf3 binding partners, including HSPA5, a master regulator of the UPR. Loss of Nrf3 was associated with increased HSPA5, and importantly, suppression of HSPA5 pharmacologically or by RNAi reduced tumor formation of Nrf3 KO SCC cells. Overall, this is a very comprehensive study with high clinical relevance. The experiments are solid, well done and clearly presented, and the study uncovers an interesting Nrf3-UPR interaction that is novel and will be of great interest to many in the field. My comments are all minor:

Does doxy-induced overexpression of Nrf3 cause downregulation of HSPA5? Either positive or negative result would be useful to know.

Fig. 1B. Is Nrf3 protein detected in hair follicles from normal skin? If not, this may suggest that reduced Nrf3 in tumors may be due to the cellular origin of these cancers, rather than protein downregulation.

Fig. 6E. The area identified in BCC2 has scant nuclei and is likely not a tumor region.

Fig. 7B. For clarity, it is suggested that the authors avoid excessive statistical comparisons that are not crucial for conveying the main message, both in this figure and elsewhere.

Please list the 34 putative Nrf3 interacting proteins in the main or supplemental figures.

Sunny Wong

Referee #2 (Comments on Novelty/Model System for Author):

Additional experiments are suggested in the comments to the authors

Referee #2 (Remarks for Author):

In this manuscript, the authors first demonstrate a down-regulation of NRF3 in basal and squamous cell carcinomas using human biopsies. They then proceed to make NRF3 KO in SCC13 and HaCat cells, as well as rescue SCC13 cells by epitopically reintroducing NRF3 into NRF3 KO cells. Utilizing these cell lines and NRF3 KO mice, the authors demonstrate a negative correlation between NRF3 protein level and malignancy phenotypes, including cell transformation induced by chemical carcinogens, anchorage-independent growth, cancer cell migration and invasion, and xenograft tumor formation. In the second part, the authors identify the mechanism underlying the role of NRF3 in malignant transformation. They show that NRF3 interacts with HSPA5, destabilizing HSPA5 proteins, and these results are consistent with the observation that SCC patients with low NRF3 expression have a high level of HSPA5. The authors then demonstrate that pharmacological or genetic inhibition of HSPA5 reduces the malignancy of NRF3-KO SCC cells in vitro and in vivo.

Overall, this study presents an important work dissecting the function of NRF3 in suppressing tumorigenicity of skin cancer. Intriguingly, the authors found that NRF3-mediated tumor suppression is independent of its function as a transcription factor in skin cells. While the study has the potential to be novel and impactful, the data presented and conclusions reached at this point are premature, and a major revision is suggested.

Two major issues:
(1) While this study presents an exciting function of NRF3, it raises an important question: are the observed effects indirectly modulated by the compensatory increase of NRF2 in NRF3-deficient cells? Enhanced NRF2 expression can promote malignant phenotypes, such as enhanced proliferation, invasion, and migration of cancer cells, larger organoids, and increased tumor incidence and multiplicity in xenograft models. All of these effects were observed in NRF3-KO cells or mice. Therefore, the authors should measure if NRF2 protein levels negatively correlate with NRF3 in these cells and determine if NRF3 regulates NRF2 levels. If this is the case, then all experiments should be repeated using NRF2; NRF3 DKO cells and Dox-inducible NRF3-

rescued cells in DKO cells. Additionally, the authors should pay attention to NRF1. For example, in tunicamycin-treated cells, NRF1 should be measured to see if it is induced.

(2) The tumor-suppressing effects of NRF2 through the NRF3-HSPA5 interaction presented in Figure 7 were not very convincing. It is known that HSPA5 is abundantly expressed in cells and forms oligomers itself or binds to many proteins, such as IRE1, ATF6, and PERK, which facilitates their activation and function later in response to ER stress. Identifying NRF3 as an interacting protein of HSPA5 is exciting, but many questions remain unanswered. For example, how does NRF3 negatively regulate the protein level of HSPA5, and is there a direct interaction between NRF3 and HSPA5? The fact that HSPA5 inhibition or knock-down reverts the high malignancy of NRF3-deficient cells is not sufficient evidence to support the argument that the tumor-suppressing effects of NRF3 occur through the NRF3-HSPA5 interaction. Acturally, this is expected based on several preclinical studies that have demonstrated the efficacy of HSPA5 inhibitors in inducing cell death in various cancer cell lines and inhibiting tumor growth in animal models. These studies suggest that targeting HSPA5 could be a promising therapeutic approach for cancer treatment, regardless of the status of NRF3.
Minor points:
Figure 3E: why is there a Dox dose-dependent increase in the GAPDH protein level?
Figure 3F&G were not discussed in the same paragraph following figure 3E. Figure 3F was discussed in the paragraph that described figure 5, but Figre 3G was never even mentioned in the text.
Figure 5A: In the figure legend, please add information of what the RNA-seq data was comparing, SCC13 NRF3-KO cells vs. "RescueNRF3" cells"?
In Figure 5: Why were cells treated with MG132 during proximity-dependent biotin identification? Even though 34 potential NRF3 interactors were chosen, which were present in both the MG-132 treated and non-treated groups, using MG132 may only increase the interaction of proteasomal proteins with NRF3, leading to increased background noise.
Figure 5G: Why was the fusion NRF3 protein used to detect PDIA3 whereas endogenous NRF3 was used to detect HSPA5 in the proximity ligation assays? Why did the authors not use the fusion NRF3 to detect both PDIA3 and HSPA5 or the endogenous NRF3 to detect both PDIA3 and HSPA5.
Figure 6C: it is better to label the Y axis in the lower right panel as "relative mRNA expression"
Figure 6E: except for SCC2, HSPA5 expression is higher inside the white dotted line than outside the white dotted line, the other three panels for HSPA5 expression are the same.
Figure 6C: Figure 6C demonstrates that "Loss of NRF3 seems to stabilize HSPA5, because HSPA5 protein levels were also higher in NRF3- KO cells under homeostatic conditions, while HSPA5 mRNA levels were not significantly affected (Fig 6C)". However, in figure 6B, the basal levels of HSPA5 in non-tunicamycin treated cells are the same. The authors need to explain this discrepancy between figure 6B and 6C.
Figure 6C: why are the error bars of the data from qRT-PCR for HSPA5 mRNA expression unusually large? Is each dot representive each experiments or difference cell lines? With such large errors, the conclusion that NRF2 negatively regulates the protein level, not the mRNA level, of HSPA5 is not solid.
Figure 6E: Are these tissues from human patients or mouse models?
Figure 7A-B: The authors stated in their conclusion that "Both inhibitors significantly reduced the spheroid size of NRF3-KO cells, while they had no or only a minimal effect on control cells (Fig 7A and B). None of the inhibitors caused significant cell death at the concentration used for the spheroid assay, and there were also no or only slight differences in survival between inhibitor-treated control and NRF3-KO cells (Appendix Fig S4A and B)." However, upon closer inspection, it was found that there were indeed statistically significant differences between the inhibitor-treated control and NRF3-KO cells in Figure S4B. This should be clarified.
Figure 7D: There is a discrepancy between Figure 7D and Figure 3D: In Figure 3D, the open scratch% for EV C2 is 80% and KO C3 is 30%, however the open scratch% for EV C2 is 80% and KO C3 is 60% in Figure 7D.

Referee #3 (Remarks for Author):

This in an interesting manuscript describing the mechanism by which NRF3 suppresses malignant progression of epithelial skin cancers. The authors demonstrate that NRF3 is downregulated in human SCC and BCC. By employing different in vivo and in vitro model systems, they show that NRF3 deficiency leads to malignant conversion of skin tumors by a mechanism involving the interaction with HSPA5, an important regulator of the unfolded protein response which the authors identified as an interaction partner of NRF3 and which is increased in the absence of NRF3. Moreover, HSPA5 seems to be upregulated also in a subset of NRF3 deficient SCC patients thus highlighting the translational potential of these findings. Inhibition or knock-down of HSPA5 can revert malignant progression in the absence of NRF3 in a preclinical setting highlighting the importance of pharmacological inhibition of HSPA5 as a future treatment option for patients.
Overall, this is a nice and important study providing convincing results that support the author's claims. Below are some suggestions that would further strengthen some of the conclusions.

1) Although the result point to a cell-autonomous function of NRF3 in epidermal cells and the number of immune cells infiltrating the tumors is similar in wt and ko mice, immune contribution can still not be formally excluded as the authors employ a complete KO model. The function of some immune cells could still be affected in the absence of NRF3 despite that their numbers are not affected. Bone marrow (BM) chimeras (wt BM in KO mice) would be a way how to demonstrate this. The authors should include

data showing lack/presence of NRF3 expression in the various infiltrating immune cells.

2) Fig 4C is small and it is not possible to see the penetration of cancer cells through the cartilage. A better or bigger picture should be shown.

3) Would it be possible to inject the "RescueNRF3" in the Xenograft model and treat with Dox to show that re-expression of NRF3 in KO cells would also revert tumor growth and malignant progression in vivo?

4) The authors could investigate if HSPA5 is also high in NRF3 KO tumors generated by DMBA/TPA. Treating these mice with the pharmacological HSPA5 inhibitor and showing that malignant progression is prevented/reverted would strengthen the translational potential of the findings. Furthermore, it would take possible effects of HSPA5 inhibitors on immune cells into consideration and the possible resulting therapeutic interferences.

5) Minor: In the graphical abstract: "Control skin cancer cells" is misleading, maybe it would be better to call it "benign skin cancer cells" or just "skin cancer cells".

Replies to reviewer comments:

Referee #1 (Remarks for Author):

This manuscript by Gurri and colleagues reveals a tumor suppressive role for Nrf3 in skin cancers. They further identified Nrf3 binding partners, including HSPA5, a master regulator of the UPR. Loss of Nrf3 was associated with increased HSPA5, and importantly, suppression of HSPA5 pharmacologically or by RNAi reduced tumor formation of Nrf3 KO SCC cells. Overall, this is a very comprehensive study with high clinical relevance. The experiments are solid, well done and clearly presented, and the study uncovers an interesting Nrf3-UPR interaction that is novel and will be of great interest to many in the field. My comments are all minor:

Does doxy-induced overexpression of Nrf3 cause downregulation of HSPA5? Either positive or negative result would be useful to know.

Our reply: We addressed this question using Dox-inducible expression of recombinant NRF3 in RescueNRF3 cells. While expression of moderate levels of NRF3 (similar to the levels seen in wild-type cells) suppressed the levels of endogenous HSPA5, a strong overexpression of NRF3 did not further reduce the levels of HSPA5. Neither low nor high expression of NRF3 affected the HSPA5 mRNA levels. These new data are shown in Fig. EV5A.

Fig. 1B. Is Nrf3 protein detected in hair follicles from normal skin? If not, this may suggest that reduced Nrf3 in tumors may be due to the cellular origin of these cancers, rather than protein downregulation.

Our reply: We have replaced the immunostained section of healthy skin (Fig. 1B) by a section from skin with multiple hair follicles. The new figure shows that NRF3 is also strongly expressed in the outer root sheath of hair follicles.

Fig. 6E. The area identified in BCC2 has scant nuclei and is likely not a tumor region.

Our reply: We have replaced the picture by another one, which clearly shows the tumor tissue.

Fig. 7B. For clarity, it is suggested that the authors avoid excessive statistical comparisons that are not crucial for conveying the main message, both in this figure and elsewhere.

Our reply: We reduced the statistical comparisons to those that are crucial for conveying the message.

Please list the 34 putative Nrf3 interacting proteins in the main or supplemental figures.

Our reply: We now show these putative interactors in the main figure (Fig. 5E).

Referee #2 (Remarks for Author):

In this manuscript, the authors first demonstrate a down-regulation of NRF3 in basal and squamous cell carcinomas using human biopsies. They then proceed to make NRF3 KO in SCC13 and HaCat cells, as well as rescue SCC13 cells by epitopically reintroducing NRF3 into NRF3 KO cells. Utilizing these cell lines and NRF3 KO mice, the authors demonstrate a negative correlation between NRF3 protein level and malignancy phenotypes, including cell transformation induced by chemical carcinogens, anchorage-independent growth, cancer cell migration and invasion, and xenograft tumor formation. In the second part, the authors identify the mechanism underlying the role of NRF3 in malignant transformation. They show that NRF3 interacts with HSPA5, destabilizing HSPA5 proteins, and these

results are consistent with the observation that SCC patients with low NRF3 expression have a high level of HSPA5. The authors then demonstrate that pharmacological or genetic inhibition of HSPA5 reduces the malignancy of NRF3-KO SCC cells in vitro and in vivo.

Overall, this study presents an important work dissecting the function of NRF3 in suppressing tumorigenicity of skin cancer. Intriguingly, the authors found that NRF3-mediated tumor suppression is independent of its function as a transcription factor in skin cells. While the study has the potential to be novel and impactful, the data presented and conclusions reached at this point are premature, and a major revision is suggested.

Two major issues:
(1) While this study presents an exciting function of NRF3, it raises an important question: are the observed effects indirectly modulated by the compensatory increase of NRF2 in NRF3-deficient cells? Enhanced NRF2 expression can promote malignant phenotypes, such as enhanced proliferation, invasion, and migration of cancer cells, larger organoids, and increased tumor incidence and multiplicity in xenograft models. All of these effects were observed in NRF3-KO cells or mice. Therefore, the authors should measure if NRF2 protein levels negatively correlate with NRF3 in these cells and determine if NRF3 regulates NRF2 levels. If this is the case, then all experiments should be repeated using NRF2; NRF3 DKO cells and Dox-inducible NRF3-rescued cells in DKO cells. Additionally, the authors should pay attention to NRF1. For example, in tunicamycin-treated cells, NRF1 should be measured to see if it is induced.

<u>Our reply:</u> In response to the reviewer comment, we analyzed the expression of NRF1, NRF2 and of major NRF2 target genes in SCC13 and HaCaT-Ras *NRF3*-KO and EV control cells, but we did not find an effect of the *NRF3* knockout on the expression or activity of the other NRF transcription factors (Revised manuscript Fig. 3A and Appendix S1 for SCC13 cells and Fig EV3A and B for HaCaT-Ras cells). We also checked for NRF1 expression in the tunicamycin-treated samples and did not find a difference between EV control cells and KO cells (shown now in Figure 6B). This finding strongly suggests that the effect of the *NRF3* knockout is independent of NRF1 and NRF2. This is consistent with our previous findings, which showed that the effect of the *Nrf3* knockout on the UVB response of keratinocytes is independent of Nrf2 (Siegenthaler et al., 2018)

(2) The tumor-suppressing effects of NRF2 through the NRF3-HSPA5 interaction presented in Figure 7 were not very convincing. It is known that HSPA5 is abundantly expressed in cells and forms oligomers itself or binds to many proteins, such as IRE1, ATF6, and PERK, which facilitates their activation and function later in response to ER stress. Identifying NRF3 as an interacting protein of HSPA5 is exciting, but many questions remain unanswered. For example, how does NRF3 negatively regulate the protein level of HSPA5, and is there a direct interaction between NRF3 and HSPA5? The fact that HSPA5 inhibition or knock-down reverts the high malignancy of NRF3-deficient cells is not sufficient evidence to support the argument that the tumor-suppressing effects of NRF3 occur through the NRF3-HSPA5 interaction. Acturally, this is expected based on several preclinical studies that have demonstrated the efficacy of HSPA5 inhibitors in inducing cell death in various cancer cell lines and inhibiting tumor growth in animal models. These studies suggest that targeting HSPA5 could be a promising therapeutic approach for cancer treatment, regardless of the status of NRF3.

<u>Our reply:</u> We indeed show interaction between HSPA5 and NRF3 using co-immunoprecipitation and close proximity by BioID and PLA. These findings strongly suggest that these proteins directly interact. This interaction seems to reduce the stability of HSPA5.

We agree with the reviewer that inhibition of HSPA5 could be beneficial for NMSC treatment, regardless of the status of NRF3. However, our spheroid assay data and the *in vivo* xenograft data show that pharmacological inhibition or knock-down of HSPA5 only have a strong and significant effect on cells/tumors that lack NRF3 (see Fig. 7). Therefore, the effect of the inhibitors is dependent on the NRF3 status. We have now further clarified this important point in the text and have repeated

the xenograft assay with the HA15 inhibitor, which again showed that the beneficial effect of the inhibitor is only seen in the *NRF3*-KO cells. The combined data of the experiments are now shown in Figure 7F and G. Below we show the graphs of the repetition experiment alone as additional information for the reviewer.

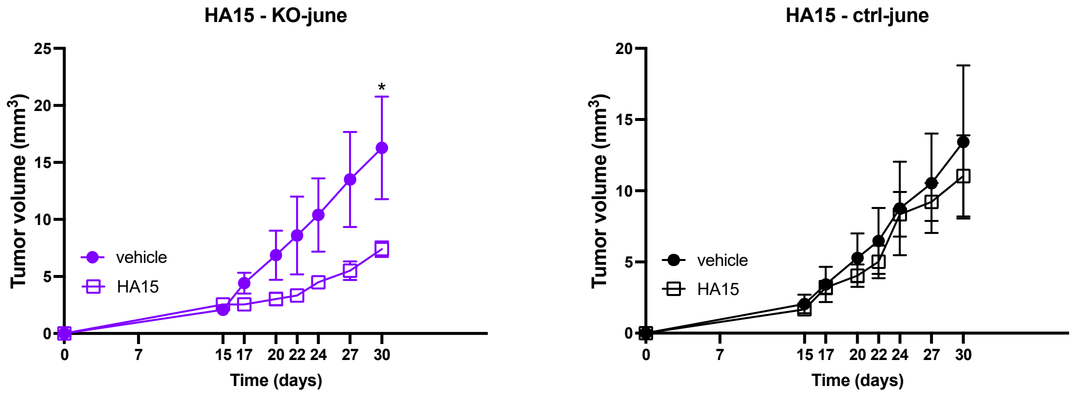

Minor points:
Figure 3E: why is there a Dox dose-dependent increase in the GAPDH protein level?

Our reply: We repeated this experiment, and we did not see an increase in GAPDH in this experiment (and also in other experiments with Dox-treated cells). The figure has been replaced.

Figure 3F&G were not discussed in the same paragraph following figure 3E. Figure 3F was discussed in the paragraph that described figure 5, but Figre 3G was never even mentioned in the text.

Our reply: Fig. 3F and G were cited together with Fig. 3E in the original version of the manuscript. We have now extended this sentence to refer specifically to each panel.

Figure 5A: In the figure legend, please add information of what the RNA-seq data was comparing, SCC13 NRF3-KO cells vs. "RescueNRF3" cells"?

Our reply: We apologize for not having mentioned this in the legend - this has now been done.

In Figure 5: Why were cells treated with MG132 during proximity-dependent biotin identification? Even though 34 potential NRF3 interactors were chosen, which were present in both the MG-132 treated and non-treated groups, using MG132 may only increase the interaction of proteasomal proteins with NRF3, leading to increased background noise.

Our reply: NRF3 has a very short half-life, and we expected to detect only very few interactors. Therefore, the experiment was also done in the presence of MG-132 to increase the chance of success. We now clarify this further in the text (page 10, lines 12-15). The fact that we identified the same interactors with or without MG-132 (and even more interactors with MG-132) is a useful validation of our results.

Figure 5G: Why was the fusion NRF3 protein used to detect PDIA3 whereas endogenous NRF3 was used to detect HSPA5 in the proximity ligation assays? Why did the authors not use the fusion NRF3 to detect both PDIA3 and HSPA5 or the endogenous NRF3 to detect both PDIA3 and HSPA5.

Our reply: The most important experiment is the PLA with the endogenous proteins, and this was done for NRF3 and HSPA5. In response to the reviewer comment, we also performed the PLA for endogenous NRF3 and PDIA3, and the new data are shown in Fig. 5H.

Figure 6C: it is better to label the Y axis in the lower right panel as "relative mRNA expression"

Our reply: We changed the Y axis label to "relative expression". We did not include the word mRNA, because it is in fact gene expression (at the RNA level)

Figure 6E: except for SCC2, HSPA5 expression is higher inside the white dotted line than outside the white dotted line, the other three panels for HSPA5 expression are the same.

Our reply: We carefully re-analyzed all photomicrographs, and exchanged some pictures by more representative ones. We also improved the labeling by white dotted lines to highlight that HSPA5 expression is overall high, but especially in NRF3-deficient areas, while the expression is lower in areas with more NRF3 expression.

Figure 6C: Figure 6C demonstrates that "Loss of NRF3 seems to stabilize HSPA5, because HSPA5 protein levels were also higher in NRF3- KO cells under homeostatic conditions, while HSPA5 mRNA levels were not significantly affected (Fig 6C)". However, in figure 6B, the basal levels of HSPA5 in non-tunicamycin treated cells are the same. The authors need to explain this discrepancy between figure 6B and 6C.

Our reply: When comparing only the basal levels of HSPA5 in the KO cells to EV cells in Fig. 6B using Mann-Whitney U test, the levels in the KO cells are also significantly higher (see graph below for comparison of basal levels). In Fig. 6B we compare four groups and therefore use a Kruskal-Wallis test, with which the difference between basal HSPA5 levels in KO and EV cells is no longer significant because of the strong increase in HSPA5 in the tunicamycin-treated cells.

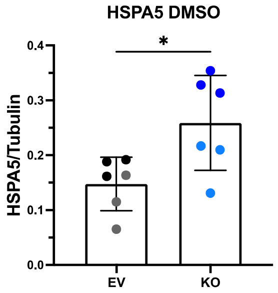

Figure 6C: why are the error bars of the data from qRT-PCR for HSPA5 mRNA expression unusually large? Is each dot representive each experiments or difference cell lines? With such large errors, the conclusion that NRF2 negatively regulates the protein level, not the mRNA level, of HSPA5 is not solid.

Our reply: We agree with the reviewer, and we therefore repeated the experiment. The result of the initial experiment was reproduced in this new experiment, but the new experiment has much smaller error bars. We now show the result of this new experiment in the manuscript (Fig. 6C). Additionally, we determined the HSPA5 RNA levels in EV and *NRF3*-KO HaCaT-Ras cells and did also not find a significant difference (shown in Fig. EV5B, right graph).

Figure 6E: Are these tissues from human patients or mouse models?

Our reply: We now clarify in the Results, in the Figure and in the Legend that these tissues are from human patients.

Figure 7A-B: The authors stated in their conclusion that "Both inhibitors significantly reduced the spheroid size of NRF3-KO cells, while they had no or only a minimal effect on control cells (Fig 7A and B). None of the inhibitors caused significant cell death at the concentration used for the spheroid assay, and there were also no or only slight differences in survival between inhibitor-treated control and NRF3-KO cells (Appendix Fig S4A and B)." However, upon closer inspection, it was found that there were indeed statistically significant differences between the inhibitor-treated control and NRF3-KO cells in Figure S4B. This should be clarified.

Our reply: We agree that there is a statistically significant (although mild) difference for one cell line, but this was not the case for the second cell line. We repeated the experiment and now show the

data of both experiments relative to the DMSO control in Appendix Fig S5A and B. When analyzing cleaved caspase 3 levels, we still found significant differences between cell line EV C1 *vs.* EVC2 and between cell line EV1 *vs.* KO C4 with the YUM70 inhibitor (but not with the HA15 inhibitor). This results from the slightly higher number of apoptotic cells in the EVC1 cell line compared to the other cell lines. Since the difference is also significant between the two control clones, this cannot be attributed to the *NRF3* knockout, but to some variability between control clones.

Figure 7D: There is a discrepancy between Figure 7D and Figure 3D: In Figure 3D, the open scratch% for EV C2 is 80% and KO C3 is 30%, however the open scratch% for EV C2 is 80% and KO C3 is 60% in Figure 7D.

Our reply: We reproducibly found a significant increase in migration in the *NRF3*-KO cells. However, there was some variability between different experiments, because they were done at different time points and with cells at another passage. Furthermore, the cells used for the experiment shown in Fig. 7D had undergone siRNA transfection, while this was not the case for the cells used for the experiment in Fig. 3D. This may well affect the extent of migration. We now clarify in the text that Fig. 7D shows migration of siRNA-transfected cells.

Referee #3 (Remarks for Author):

This in an interesting manuscript describing the mechanism by which NRF3 suppresses malignant progression of epithelial skin cancers. The authors demonstrate that NRF3 is downregulated in human SCC and BCC. By employing different in vivo and in vitro model systems, they show that NRF3 deficiency leads to malignant conversion of skin tumors by a mechanism involving the interaction with HSPA5, an important regulator of the unfolded protein response which the authors identified as an interaction partner of NRF3 and which is increased in the absence of NRF3. Moreover, HSPA5 seems to be upregulated also in a subset of NRF3 deficient SCC patients thus highlighting the translational potential of these findings. Inhibition or knock-down of HSPA5 can revert malignant progression in the absence of NRF3 in a preclinical setting highlighting the importance of pharmacological inhibition of HSPA5 as a future treatment option for patients.
Overall, this is a nice and important study providing convincing results that support the author's claims. Below are some suggestions that would further strengthen some of the conclusions.

1) Although the result point to a cell-autonomous function of NRF3 in epidermal cells and the number of immune cells infiltrating the tumors is similar in wt and ko mice, immune contribution can still not be formally excluded as the authors employ a complete KO model. The function of some immune cells could still be affected in the absence of NRF3 despite that their numbers are not affected. Bone marrow (BM) chimeras (wt BM in KO mice) would be a way how to demonstrate this. The authors should include data showing lack/presence of NRF3 expression in the various infiltrating immune cells.

Our reply: To address this comment, we analyzed the Immunological Genome (Immgen) database and found that immune cells generally express no or very low levels of *NRF3* (http://rstats.immgen.org/Skyline/skyline.html). The only immune cells that show detectable *Nrf3* expression are dendritic cells, and this was also seen by analysis of single cell RNA sequencing data of 10 skin SCCs (Ji et al., 2020, Cell). By contrast, analysis of RNA-sequencing data of macrophages from pre-tumorigenic mouse skin (Antsiferova et al, EMBO Molecular Medicine 2017) did not show detectable levels of Nrf3 mRNA. Finally, *Nrf3* expression was not detectable by qRT-PCR using RNA extracted from isolated bone marrow-derived macrophages (BMDM) both before and after polarization, while expression of *Nrf3* was clearly detectable in cultured primary mouse keratinocytes (pmKC; see additional information for the reviewer shown below).

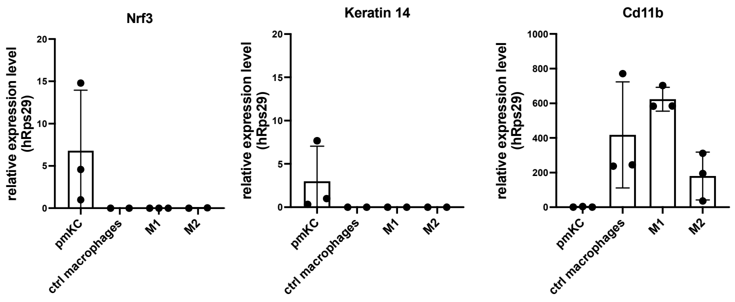

Together, these data further suggest that the loss of Nrf3 in keratinocytes is mainly responsible for the enhanced malignant conversion that we observed in the DMBA/TPA carcinogenesis study. Nevertheless, we cannot fully rule out a contribution of immune cells, and we therefore mention this specifically in the manuscript (page6/7, lines 25/1-7).

2) Fig 4C is small and it is not possible to see the penetration of cancer cells through the cartilage. A better or bigger picture should be shown.

Our reply: We now show a better picture with a higher magnification in Fig. 4C.

3) Would it be possible to inject the "RescueNRF3" in the Xenograft model and treat with Dox to show that re-expression of NRF3 in KO cells would also revert tumor growth and malignant progression in vivo?

Our reply: This is generally possible, but it is not fully clear if sufficient amounts of Dox reach the ear to induce robust expression of the transgene. This is also an issue in the experiment shown in Appendix Fig. S2 (Dox-inducible expression of NRF3 in wild-type cells), and we now mention this in the discussion (page 16, lines 2-7). Therefore, we used the approach suggested by the reviewer in a spheroid assay with RescueNRF3 cells. Indeed, spheroid size was also reduced by Dox-inducible re-expression of recombinant NRF3 in the *NRF3*-KO cells (Fig 3I, revised version).

4) The authors could investigate if HSPA5 is also high in NRF3 KO tumors generated by DMBA/TPA. Treating these mice with the pharmacological HSPA5 inhibitor and showing that malignant progression is prevented/reverted would strengthen the translational potential of the findings. Furthermore, it would take possible effects of HSPA5 inhibitors on immune cells into consideration and the possible resulting therapeutic interferences.

Our reply: As suggested by the reviewer, we stained sections from the DMBA/TPA-induced tumors for HSPA5. However, because to the strong hyperkeratosis in these tumors, there was a lot of background staining. Therefore, we quantified the HSPA5 intensity in the non-hyperkeratotic epithelial (E-Cadherin positive) area and found that the tumors formed in *Nrf3*-ko mice indeed show increased HSPA5 staining intensity. These data are now shown in Appendix Fig. S4.

We agree that treatment of mice subjected to DMBA/TPA-induced carcinogenesis with the HSPA5 inhibitor would be an interesting experiment. However, this was not possible within the 3-months period that was allowed for the revision, because the tumorigenesis study takes at least 20 weeks (not including the time for the breeding of a large number of mice). In addition, we would have to inject the inhibitor for many weeks, and we currently do not have a licence to perform such long-term treatment experiments.

5) Minor: In the graphical abstract: "Control skin cancer cells" is misleading, maybe it would be better to call it "benign skin cancer cells" or just "skin cancer cells".

Our reply: We have replaced "Control skin cancer cells" by "NRF3-positive skin cancer cells".

14th Aug 2023

Dear Sabine,

Thank you for the submission of your revised manuscript to EMBO Molecular Medicine, and please accept my apologies for the delay in getting back to you as one referee needed more time to complete his/her review. We have now received the enclosed reports from the two referees who were invited to evaluate your revised manuscript.
As you will see below, while they appreciate the work that was done to address their initial comments, they still have remaining concerns:

- Referee #2 is not convinced of the tumor-suppressing effects of NRF3 through HSPA5, in particular in light of the figure 3 presented in the rebuttal letter.
- Referee #3 regrets that NRF3 rescue experiment was not performed in vivo.

Referee #3 agreed with referee #2's comment, and further suggested to show the 4 tumor growth curves all in one graph (Fig. 7F, G) to allow statistical comparison and better visualization of the NRF3-KO spheroid growth.
Referee #3 further commented that while HA-15 inhibitor clearly inhibits tumor growth only in cells with NRF-3 KO, the interdependence between NRF-3 and HSPA5 remains unclear. This referee mentioned that performing the HSPA5 inhibitor experiment in another system as suggested in their report (point 4) would have helped clarifying this issue. Performing in vivo tumor study / inhibitor treatment with re-expressing NRF-3 cells (rescue NRF3) as suggested in point 3 could also contribute to elucidate this issue.

We agree with the referees that the interdependence between NRF3 and HSPA5 is an important point to clarify and would therefore like to invite you to revise the manuscript further to address the referees' remaining concerns.

If you would like to discuss the issues raised by the referees, I am available to do so via email or video. Let me know if you are interested in this option.

Moreover, please address the following editorial requests:
- Please address the queries from our data editors in the related "Data edited MS file". Please remove the red text, and only keep in track changes mode any new modification.
- Please replace the "Conflict of interest" by "Disclosure Statement and Competing Interests". We updated our journal's competing interests policy in January 2022 and request authors to consider both actual and perceived competing interests. Accordingly, please include the following sentence: "Sabine Werner is a member of the Advisory Editorial Board of EMBO Molecular Medicine. This has no bearing on the editorial consideration of this article for publication."
- Acknowledgements and funding: please double-check whether the following also needs to be entered in the submission system: Life Science Zurich Graduate School and SKINTEGRITY.CH collaborative research program
- Thank you for providing a synopsis image. Please resize it to 550 pixels wide x 200-400 pixels high and make sure the text remains legible.
- Please use Arabic numbers instead of Roman in your manuscript tables. These tables should be placed between the main and EV figure legends
- Appendix Table SI and SII: please use Arabic numbers instead of Roman. These tables should be uploaded as Datasets and their reference in the text should be adjusted accordingly.

As part of the EMBO Publications transparent editorial process initiative (see our Editorial at http://embomolmed.embopress.org/content/2/9/329), EMBO Molecular Medicine will publish online a Review Process File (RPF) to accompany accepted manuscripts.
This file will be published in conjunction with your paper and will include the anonymous referee reports, your point-by-point response and all pertinent correspondence relating to the manuscript. Let us know whether you agree with the publication of the RPF and as here, if you want to remove or not any figures from it prior to publication.
Please note that the Authors checklist will be published at the end of the RPF.

I look forward to receiving your revised manuscript.

With kind regards,

Lise

Lise Roth, PhD
Senior Editor
EMBO Molecular Medicine

***** Reviewer's comments *****

Referee #2 (Remarks for Author):

The author has responded well to the major issue 1, but I still have some concerns regarding major issue 2. In their reply, they state that the tumor-suppressing effects of NRF3 through the NRF3-HSPA5 interaction presented in Figure 7 were not very convincing. They argue that their spheroid assay data and the in vivo xenograft data demonstrate that pharmacological inhibition or knock-down of HSPA5 only has a strong and significant effect on cells/tumors that lack NRF3 (see Fig. 7). They suggest that the effect of the inhibitors is dependent on the NRF3 status.

However, I find this explanation somewhat confusing. According to their model, the HA15-KO (NRF3 KO) cell line should have a higher expression of HAPA5 (the target protein), and therefore, it should be more resistant to the HSPA5 inhibitor. However, the presented results contradict this expectation. Furthermore, the comparison between the untreated vehicle control groups of HA15-KO and HA15-ctrl in the figure on page 3 of the point-by-point response shows no significant difference. These observations raise doubts regarding the tumor-suppressing effects of NRF3 through HSPA5.

Another factor worth considering is the role of HSP60 proteins in assisting protein-folding, while NRF3 is an intrinsically disordered protein. Therefore, it is not unexpected to find the binding of NRF3 with HSPA5.

Overall, my major concern regarding the tumor-suppressing effects of NRF3 through HSPA5 remains.

Referee #3 (Remarks for Author):

The authors have made a great effort to improve their manuscript. Most of the concerns raised by this reviewer were addressed. However, the dependence on NRF-3 status for response to HSPA5 inhibitors still remain unclear.

ad point 3) I appreciate that NRF-3 was re-expressed in NRF-3 KO cells and new data by using an in vitro spheroid assay are shown in Fig 3I. However, the authors should have performed this experiment also in vivo, to strengthen the interdependence of NRF-3 and HSPA5.Dox might not penetrate well into the tumors, but maybe Dox could be injected/applied directly to the ear, similarly to experiments with the inhibitor treatments. This experiment could be combined with HSPA5 inhibitor treatment which would also address some of the issues raised by reviewer 2.
This point should be clarified before publication.

ad point 4) Increased levels of HSPA5 are shown in DMBA/TPA tumors which supports their hypothesis. For reasons given before, it would have been important to see HSPA5 inhibitor treatment in an independent model (DMBA/TPA) but the experiment would indeed take too long.

Reply to Reviewer Comments

Referee #2 (Remarks for Author):

The author has responded well to the major issue 1, but I still have some concerns regarding major issue 2. In their reply, they state that the tumor-suppressing effects of NRF3 through the NRF3-HSPA5 interaction presented in Figure 7 were not very convincing.

Our reply: We did not make this statement. This was the statement of reviewer 2 in the initial report, which we included in italics to facilitate the review.

They argue that their spheroid assay data and the in vivo xenograft data demonstrate that pharmacological inhibition or knock-down of HSPA5 only has a strong and significant effect on cells/tumors that lack NRF3 (see Fig. 7). They suggest that the effect of the inhibitors is dependent on the NRF3 status.

However, I find this explanation somewhat confusing. According to their model, the HA15-KO (NRF3 KO) cell line should have a higher expression of HAPA5 (the target protein), and therefore, it should be more resistant to the HSPA5 inhibitor. However, the presented results contradict this expectation.

Our reply: *NRF3*-KO cells indeed have higher levels of HSPA5. This finding encouraged us to focus on this protein, because high levels of HSPA5 had previously been associated with high malignancy in several other tumors (see Discussion). Therefore, we hypothesized that the enhanced levels of HSPA5 contribute to the higher malignancy of *NRF3*-KO SCC cells. We tested this hypothesis by knock-down and pharmacological inhibition of HSPA5, and both treatments indeed reverted the higher malignancy of *NRF3*-KO cells, while they had no effect on wild-type cells.

In the inhibitor experiments, we added an excess of the inhibitor and therefore, all HSPA5 should be efficiently inhibited. Of note, HSPA5 is not mutated, but only overexpressed in the *NRF3*-KO cells. Therefore, the inhibitors are effective. Importantly, it has even been shown for melanoma cells that the inhibitor is more effective when the concentrations of HSPA5 are high, and melanoma cells expressing high levels of HSPA5 responded to HA15 better than those expressing a low level of HSPA5 (Cerezo et al., *Cancer Cell* 2016). We have clarified this important point in the text (page 13, lines 5-6 and page 18, lines 7-8).

The fact that HSPA5 inhibition (using two different inhibitors!) as well as HSPA5 knock-down reverts the higher malignancy of *NRF3*-KO SCC cells in different assays clearly supports our conclusion that HSPA5 is involved in the tumor-suppressive effect of NRF3.

Furthermore, the comparison between the untreated vehicle control groups of HA15-KO and HA15-ctrl in the figure on page 3 of the point-by-point response shows no significant difference. These observations raise doubts regarding the tumor-suppressing effects of NRF3 through HSPA5.

Our reply: This comparison was not made in this figure, but this has now been done (see new Fig. EV5G). We still prefer to show the data in two separate panels in the main figure (Fig. 7F and G), because there are too many data points in one figure when we combine it, which is confusing. However, we now show the combined data as an additional bar graph in Fig. EV5G. We used 3-way ANOVA to determine the influence of the genotype, the treatment regimen and the time, and confirmed that HSPA5 only has a significant inhibitory

effect in the tumors formed by the *NRF3*-KO cells (see table in Fig. EV5G). We would also like to point out that the comparison of untreated HaCaT-ras WT and HaCaT *NRF3*-KO cells in the xenograft assay is shown in Fig. EV3G, even with more mice and for a longer time period. The difference was clear at all time points, but it was most pronounced (and significant) after day 30. In the experiment shown in Fig. 7 we still observed this difference, but the experiment was stopped at day 30 (when the difference was not yet significant - see bar graph in new Fig. EV5G), because the difference between HSPA5-treated and vehicle-treated tumors was already obvious and because we are not allowed to inject for longer time periods according to animal welfare regulation. This has now been clarified in the text (page 13, last paragraph and page 14, first paragraph).

Another factor worth considering is the role of HSP60 proteins in assisting protein-folding, while NRF3 is an intrinsically disordered protein. Therefore, it is not unexpected to find the binding of NRF3 with HSPA5.

<u>Our reply:</u> We agree with the reviewer that the identification of HSPA5 in our BioID screen was not very surprising. We also did not confirm a direct interaction between both proteins, and we therefore expressed this more carefully in the revised manuscript (page 12, first paragraph). The <u>relevant</u> finding was the strong increase in HSPA5 levels upon *NRF3* knockout and the rescue of the higher malignancy of *NRF3*-KO cells by HSPA5 inhibition or knock-down. We further clarified this issue in the text (page 14, first paragraph).

Overall, my major concern regarding the tumor-suppressing effects of NRF3 through HSPA5 remains.

<u>Our reply:</u> We tested the effect of HSPA5 inhibition in 2D and 3D *in vitro* and in a xenograft experiment *in vivo*. The results were consistent in all these experiments - HSPA5 inhibition or knock-down rescued the malignant features of *NRF3*-KO cells. Therefore, we believe that our conclusion regarding the important role of HSPA5 in the tumor-suppressive effect of NRF3 is justified. However, it is of course possible that additional mechanisms contribute to the tumor-suppressive effect of NRF3 in keratinocytes, and we now mention this possibility in the Discussion (page 18, line 3). We also made this clear by changing the title, and we replaced "through HSPA5" by "involving" HSPA5.

Referee #3 (Remarks for Author):

The authors have made a great effort to improve their manuscript. Most of the concerns raised by this reviewer were addressed. However, the dependence on NRF-3 status for response to HSPA5 inhibitors still remain unclear.

ad point 3) I appreciate that NRF-3 was re-expressed in NRF-3 KO cells and new data by using an in vitro spheroid assay are shown in Fig 3I. However, the authors should have performed this experiment also in vivo, to strengthen the interdependence of NRF-3 and HSPA5.Dox might not penetrate well into the tumors, but maybe Dox could be injected/applied directly to the ear, similarly to experiments with the inhibitor treatments. This experiment could be combined with HSPA5 inhibitor treatment which would also address some of the issues raised by reviewer 2. This point should be clarified before publication.

<u>Our reply:</u> In his/her initial review the reviewer addressed this point as a question ("would it be possible to inject the "RescueNRF3" in the xenograft model and treat with Dox to show that re-expression of NRF3 in KO cells would also revert tumor growth and malignant progression in vivo?". Therefore, we did not understand this as a request to do this experiment. Nevertheless, we this comment is important and therefore, we performed the spheroid experiment, which gave the expected results (re-expression of NRF3 reverted the tumorigenic features of the *NRF3*-KO cells). It was not requested to combine such an experiment with an HSPA5 inhibitor.

In our opinion, the comparison of *NRF3*-KO cells with wild-type cells is the best comparison, because expression of the endogenous protein may be differently regulated compared to the expression of the recombinant protein. This is relevant, because NRF3 is obviously regulated at the posttranscriptional level during tumorigenesis. Furthermore, it is not fully clear if the overexpressed protein is present at similar levels in the different cellular compartments as the wild-type protein in control cells. Even if this were the case, we believe that repetition of the inhibitor experiments with the NRF3-Rescue cells *in vivo* would not provide additional mechanistic insight, in particular because the 3D spheroid assay gave the expected result.

ad point 4) Increased levels of HSPA5 are shown in DMBA/TPA tumors which supports their hypothesis. For reasons given before, it would have been important to see HSPA5 inhibitor treatment in an independent model (DMBA/TPA) but the experiment would indeed take too long.

<u>Our reply:</u> The reviewer agrees that this experiment is too long - it would be problematic for animal welfare reasons, and we would not get permission to inject mice for such a long time. In addition, the inhibitor would have to be used systemically. This would require a thorough optimization of the dosage, and there may be toxicity upon long-term systemic treatment.

We tested the effect of HSPA5 inhibition in 2D and 3D *in vitro* and in a xenograft experiment *in vivo*. The results were consistent in all these experiments - HSPA5 inhibition or knock-down rescued the malignant features of *NRF3*-KO cells. Therefore, we believe that our conclusion regarding the important role of HSPA5 in the tumor-suppressive effect of NRF3 is justified. However, it is of course possible that additional mechanisms contribute to the tumor-suppressive effect of NRF3 in keratinocytes, and we now mention this possibility in the Discussion (page 18, line 3). We also made this clear by changing the title, and we replaced "through HSPA5" by "involving" HSPA5.

Editorial requests:

- Please address the queries from our data editors in the related "Data edited MS file". Please remove the red text, and only keep in track changes mode any new modification.

<u>Our reply:</u> This has been done.

- Please replace the "Conflict of interest" by "Disclosure Statement and Competing Interests". We updated our journal's competing interests policy in January 2022 and request authors to consider both actual and perceived competing interests. Accordingly, please include the following sentence: "Sabine Werner is a member of the Advisory Editorial Board of EMBO Molecular Medicine. This has no bearing on the editorial consideration of this article for publication."

<u>Our reply:</u> This has been done.

- Acknowledgements and funding: please double-check whether the following also needs to be entered in the submission system: Life Science Zurich Graduate School and SKINTEGRITY.CH collaborative research program

Our reply: This does not have to be entered in the submission system, because we did not receive funding from the Life Science Zurich Graduate School or from SKINTEGRITY.CH for this project.

- Thank you for providing a synopsis image. Please resize it to 550 pixels wide x 200-400 pixels high and make sure the text remains legible.

Our reply: This has been done.

- Please use Arabic numbers instead of Roman in your manuscript tables. These tables should be placed between the main and EV figure legends

Our reply: This has been done.

- Appendix Table SI and SII: please use Arabic numbers instead of Roman. These tables should be uploaded as Datasets and their reference in the text should be adjusted accordingly.

Our reply: This has been done.

15th Sep 2023

Dear Sabine,

Please find enclosed the final reports on your manuscript. I am pleased to inform you that your manuscript is accepted for publication and is now being sent to our publisher to be included in the next available issue of EMBO Molecular Medicine!

I noted that on p.29 you wrote: "The following primary antibodies were used:" but no antibody is listed, please double-check.

We also kindly remind you that datasets must be available to the public before publication of your manuscript.

Congratulations on your interesting work!

With kind regards,

Lise

Lise Roth, Ph.D
Senior Editor
EMBO Molecular Medicine

Follow us on Twitter @EmboMolMed
Sign up for eTOCs at embopress.org/alertsfeeds

***** Reviewer's comments *****

Referee #2 (Remarks for Author):

Is suitable for publication

Referee #3 (Comments on Novelty/Model System for Author):

everything fine

Referee #3 (Remarks for Author):

The authors have addressed the concerns, and it is agreed that some of the requested in vivo experiments cannot be done easily. Nevertheless, the data are strong and support the conclusion.

*** *** *** IMPORTANT INFORMATION *** *** ***

SPEED OF PUBLICATION
The journal aims for rapid publication of papers, using using the advance online publication "Early View" to expedite the process: A properly copy-edited and formatted version will be published as "Early View" after the proofs have been corrected. Please help the Editors and publisher avoid delays by providing e-mail address(es), telephone and fax numbers at which author(s) can be contacted.

Should you be planning a Press Release on your article, please get in contact with embomolmed@wiley.com as early as possible, in order to coordinate publication and release dates.

LICENSE AND PAYMENT:

All articles published in EMBO Molecular Medicine are fully open access: immediately and freely available to read, download

and share.

EMBO Molecular Medicine charges an article processing charge (APC) to cover the publication costs. You, as the corresponding author for this manuscript, should have already received a quote with the article processing fee separately. Please let us know in case this quote has not been received.

Once your article is at Wiley for editorial production you will receive an email from Wiley's Author Services system, which will ask you to log in and will present you with the publication license form for completion. Within the same system the publication fee can be paid by credit card, an invoice, pro forma invoice or purchase order can be requested.

Payment of the publication charge and the signed Open Access Agreement form must be received before the article can be published online.

PROOFS

You will receive the proofs by e-mail approximately 2 weeks after all relevant files have been sent o our Production Office. Please return them within 48 hours and if there should be any problems, please contact the production office at embopressproduction@wiley.com.

Please inform us if there is likely to be any difficulty in reaching you at the above address at that time. Failure to meet our deadlines may result in a delay of publication.

All further communications concerning your paper proofs should quote reference number EMM-2023-17761-V3 and be directed to the production office at embopressproduction@wiley.com.

EMBO Press Author Checklist

Corresponding Author Name: Sabine Werner
Journal Submitted to: EMBO Molecular Medicine
Manuscript Number: EMM-2023-17761

USEFUL LINKS FOR COMPLETING THIS FORM
The EMBO Journal - Author Guidelines
EMBO Reports - Author Guidelines
Molecular Systems Biology - Author Guidelines
EMBO Molecular Medicine - Author Guidelines

Reporting Checklist for Life Science Articles (updated January
This checklist is adapted from Materials Design Analysis Reporting (MDAR) Checklist for Authors. MDAR establishes a minimum set of requirements in transparent reporting in the life sciences (see Statement of Task: 10.31222/osf.io/9sm4x). Please follow the journal's guidelines in preparing your
Please note that a copy of this checklist will be published alongside your article.

Abridged guidelines for figures
1. Data
The data shown in figures should satisfy the following conditions:
- the data were obtained and processed according to the field's best practice and are presented to reflect the results of the experiments in an accurate and unbiased manner.
- ideally, figure panels should include only measurements that are directly comparable to each other and obtained with the same assay.
- plots include clearly labeled error bars for independent experiments and sample sizes. Unless justified, error bars should not be shown for technical
- if n<5, the individual data points from each experiment should be plotted. Any statistical test employed should be justified.
- Source Data should be included to report the data underlying figures according to the guidelines set out in the authorship guidelines on Data

2. Captions
Each figure caption should contain the following information, for each panel where they are relevant:
- a specification of the experimental system investigated (eg cell line, species name).
- the assay(s) and method(s) used to carry out the reported observations and measurements.
- an explicit mention of the biological and chemical entity(ies) that are being measured.
- an explicit mention of the biological and chemical entity(ies) that are altered/varied/perturbed in a controlled manner.
- the exact sample size (n) for each experimental group/condition, given as a number, not a range;
- a description of the sample collection allowing the reader to understand whether the samples represent technical or biological replicates (including how many animals, litters, cultures, etc.).
- a statement of how many times the experiment shown was independently replicated in the laboratory.
- definitions of statistical methods and measures:
  - common tests, such as t-test (please specify whether paired vs. unpaired), simple χ2 tests, Wilcoxon and Mann-Whitney tests, can be unambiguously identified by name only, but more complex techniques should be described in the methods section;
  - are tests one-sided or two-sided?
  - are there adjustments for multiple comparisons?
  - exact statistical test results, e.g., P values = x but not P values < x;
  - definition of 'center values' as median or average;
  - definition of error bars as s.d. or s.e.m.

Please complete ALL of the questions below.
Select "Not Applicable" only when the requested information is not relevant for your study.

Materials

Newly Created Materials	Information included in the manuscript?	In which section is the information available? (Reagents and Tools Table, Materials and Methods, Figures, Data Availability Section)
New materials and reagents need to be available; do any restrictions apply?	Yes	Materials and Methods

Antibodies	Information included in the manuscript?	In which section is the information available? (Reagents and Tools Table, Materials and Methods, Figures, Data Availability Section)
For **antibodies** provide the following information:<br>- Commercial antibodies: RRID (if possible) or supplier name, catalogue number and or/clone number<br>- Non-commercial: RRID or citation	Yes	Tables IV, V, VII and VIII

DNA and RNA sequences	Information included in the manuscript?	In which section is the information available? (Reagents and Tools Table, Materials and Methods, Figures, Data Availability Section)
**Short novel DNA or RNA including primers, probes**: provide the sequences.	Yes	Tables I, II, III and VI

Cell materials	Information included in the manuscript?	In which section is the information available? (Reagents and Tools Table, Materials and Methods, Figures, Data Availability Section)
**Cell lines:** Provide species information, strain. Provide accession number in repository **OR** supplier name, catalog number, clone number, and/**OR** RRID.	Yes	Materials and Methods
**Primary cultures:** Provide species, strain, sex of origin, genetic modification status.	Yes	Materials and Methods, Results
Report if the cell lines were recently **authenticated** (e.g., by STR profiling) and tested for mycoplasma contamination.	Yes	Materials an Methods

Experimental animals	Information included in the manuscript?	In which section is the information available? (Reagents and Tools Table, Materials and Methods, Figures, Data Availability Section)
**Laboratory animals or Model organisms:** Provide species, strain, sex, age, genetic modification status. Provide accession number in repository **OR** supplier name, catalog number, clone number, **OR** RRID.	Yes	Materials and Methods
**Animal observed in or captured from the field:** Provide species, sex, and age where possible.	Not Applicable	
Please detail **housing and husbandry conditions**.	Yes	Materials and Methods

Plants and microbes	Information included in the manuscript?	In which section is the information available? (Reagents and Tools Table, Materials and Methods, Figures, Data Availability Section)
**Plants:** provide species and strain, ecotype and cultivar where relevant, unique accession number if available, and source (including location for collected wild specimens).	Not Applicable	
**Microbes:** provide species and strain, unique accession number if available, and source.	Not Applicable	

Human research participants	Information included in the manuscript?	In which section is the information available? (Reagents and Tools Table, Materials and Methods, Figures, Data Availability Section)
If collected and within the bounds of privacy constraints report on age, sex and gender or ethnicity for all study participants.	Not Applicable	

Core facilities	Information included in the manuscript?	In which section is the information available? (Reagents and Tools Table, Materials and Methods, Figures, Data Availability Section)
If your work benefited from core facilities, was their service mentioned in the acknowledgments section?	Yes	Acknowledgement

Design

Study protocol	Information included in the manuscript?	In which section is the information available? (Reagents and Tools Table, Materials and Methods, Figures, Data Availability Section)
If study protocol has been **pre-registered, provide DOI in the manuscript**. For clinical trials, provide the trial registration number **OR** cite DOI.	Not Applicable	
Report the **clinical trial registration number** (at ClinicalTrials.gov or equivalent), where applicable.	Not Applicable	

Laboratory protocol	Information included in the manuscript?	In which section is the information available? (Reagents and Tools Table, Materials and Methods, Figures, Data Availability Section)
Provide DOI OR other citation details if **external detailed step-by-step protocols** are available.	Not Applicable	

Experimental study design and statistics	Information included in the manuscript?	In which section is the information available? (Reagents and Tools Table, Materials and Methods, Figures, Data Availability Section)
Include a statement about **sample size** estimate even if no statistical methods were used.	Yes	Figure Legends
Were any steps taken to minimize the effects of subjective bias when allocating animals/samples to treatment (e.g. **randomization procedure**)? If yes, have they been described?	Yes	Materials and Methods
Include a statement about **blinding** even if no blinding was done.	Yes	Materials and Methods
Describe **inclusion/exclusion criteria** if samples or animals were excluded from the analysis. Were the criteria pre-established?<br><br>If sample or data points were omitted from analysis, report if this was due to attrition or intentional exclusion and provide justification.	Yes	Materials and Methods, no data points were excluded
For every figure, are **statistical tests** justified as appropriate? Do the data meet the assumptions of the tests (e.g., normal distribution)? Describe any methods used to assess it. Is there an estimate of variation within each group of data? Is the variance similar between the groups that are being statistically compared?	Yes	Materials and Methods, Figure Legends

Sample definition and in-laboratory replication	Information included in the manuscript?	In which section is the information available? (Reagents and Tools Table, Materials and Methods, Figures, Data Availability Section)
In the figure legends: state number of times the experiment was **replicated** in laboratory.	Yes	Figure Legends
In the figure legends: define whether data describe **technical or biological replicates**.	Yes	Figure Legends

Ethics

Ethics	Information included in the manuscript?	In which section is the information available? (Reagents and Tools Table, Materials and Methods, Figures, Data Availability Section)
Studies involving **human participants**: State details of **authority granting ethics approval** (IRB or equivalent committee(s), provide reference number for approval.	Yes	Materials and Methos
Studies involving **human participants**: Include a statement confirming that **informed consent** was obtained from all subjects and that the experiments conformed to the principles set out in the WMA Declaration of Helsinki and the Department of Health and Human Services Belmont Report.	Yes	Materials and Methos
Studies involving **human participants:** For publication of **patient photos**, include a statement confirming that consent to publish was obtained.	Not Applicable	
Studies involving experimental **animals**: State details of **authority granting ethics approval** (IRB or equivalent committee(s), provide reference number for approval. Include a statement of compliance with ethical regulations.	Yes	Materials and Methos
Studies involving **specimen and field samples:** State if relevant **permits** obtained, provide details of authority approving study; if none were required, explain why.	Not Applicable	

Dual Use Research of Concern (DURC)	Information included in the manuscript?	In which section is the information available? (Reagents and Tools Table, Materials and Methods, Figures, Data Availability Section)
Could your study fall under dual use research restrictions? Please check biosecurity documents and list of **select agents and toxins** (CDC): https://www.selectagents.gov/sat/list.htm	Not Applicable	
If you used a select agent, is the security level of the lab appropriate and reported in the manuscript?	Not Applicable	
If a study is subject to dual use research of concern regulations, is the name of the **authority granting approval and reference number** for the regulatory approval provided in the manuscript?	Not Applicable	

Reporting

The MDAR framework recommends adoption of discipline-specific guidelines, established and endorsed through community initiatives. Journals have their own policy about requiring specific guidelines and recommendations to complement MDAR.

Adherence to community standards	Information included in the manuscript?	In which section is the information available? (Reagents and Tools Table, Materials and Methods, Figures, Data Availability Section)
State if relevant guidelines or checklists (e.g., **ICMJE, MIBBI, ARRIVE, PRISMA**) have been followed or provided.	Not Applicable	
For **tumor marker prognostic studies,** we recommend that you follow the **REMARK** reporting guidelines (see link list at top right). See author guidelines, under 'Reporting Guidelines'. Please confirm you have followed these guidelines.	Not Applicable	
For **phase II and III randomized controlled trials**, please refer to the **CONSORT** flow diagram (see link list at top right) and submit the CONSORT checklist (see link list at top right) with your submission. See author guidelines, under 'Reporting Guidelines'. Please confirm you have submitted this list.	Not Applicable	

Data Availability

Data availability	Information included in the manuscript?	In which section is the information available? (Reagents and Tools Table, Materials and Methods, Figures, Data Availability Section)
Have **primary datasets** been deposited according to the journal's guidelines (see 'Data Deposition' section) and the respective accession numbers provided in the Data Availability Section?	Yes	Data Availability Section
Were **human clinical and genomic datasets** deposited in a public access-controlled repository in accordance to ethical obligations to the patients and to the applicable consent agreement?	Not Applicable	
Are **computational models** that are central and integral to a study available without restrictions in a machine-readable form? Were the relevant accession numbers or links provided?	Not Applicable	
If publicly available data were reused, provide the respective **data citations in the reference list**.	Yes	Results and Reference List
